# Synthesis and Antitumor Evaluation of Menthone-Derived Pyrimidine-Urea Compounds as Potential PI3K/Akt/mTOR Signaling Pathway Inhibitor

**DOI:** 10.3389/fchem.2021.815531

**Published:** 2022-02-03

**Authors:** Mei Huang, Wengui Duan, Naiyuan Chen, Guishan Lin, Xiu Wang

**Affiliations:** ^1^ School of Chemistry and Chemical Engineering, Guangxi University, Nanning, China; ^2^ Guangxi Research Institute of Chemical Industry Co., Ltd., Nanning, China; ^3^ School of Public Health, Guangxi Medical University, Nanning, China; ^4^ Guangxi Colleges and Universities Key Laboratory of Prevention and Control of Highly Prevalent Diseases, Guangxi Medical University, Nanning, China

**Keywords:** menthone, pyrimidine, urea, antitumor activity, synthesis, PI3K/AKT/mTOR

## Abstract

A series of novel menthone derivatives bearing pyrimidine and urea moieties was designed and synthesized to explore more potent natural product-derived antitumor agents. The structures of the target compounds were confirmed by FTIR, NMR, and HRMS. The *in vitro* antitumor activity was tested by standard methyl thiazolytetrazolium assay and showed that **4i**, **4g**, **4s**, and **4m** are the best compounds with IC_50_ values of 6.04 ± 0.62µM, 3.21 ± 0.67µM, 19.09 ± 0.49µM, and 18.68 ± 1.53µM, against Hela, MGC-803, MCF-7, and A549, respectively. The results of the preliminary action mechanism studies showed that compound **4i**, the representative compound, could induce cell apoptosis in Hela cells in a dose-dependent manner and might arrest the cell cycle in the G2/M phase. Furthermore, the results of network pharmacology prediction and Western blot experiments indicated that compound **4i** might inhibit Hela cells through inhibit PI3K/Akt/mTOR signaling pathway. The binding modes and the binding sites interactions between compound **4i** and the target proteins were predicted preliminarily by the molecular docking method.

## Introduction

*Cancer* is a life-threatening disease with a high mortality rate and a major public health problem worldwide (Siegel et al., 2018). Although many new significant therapeutic methods were developed during the past decades, chemotherapy remains the main method for cancer treatment (Bukowski et al., 2020). However, the drug resistance of tumors restricted the applications of many chemotherapeutic drugs in treatments (Kumar et al., 2015; Gao et al., 2019). The tumors could be resistant to some chemotherapeutic drugs intrinsically, and show acquired drug resistance after chemotherapy, which makes the tumors become insensitive to similar drugs later. Therefore, the development of new drugs to overcome the resistance is still an important mission for medicinal chemists.

PI3K/Akt/mTOR pathway has been reported as an important cell growth signaling pathway. This pathway is excessively activated in many tumor cells and facilitates the cancer cells resistance to chemotherapy (Chen et al., 2010; Zhang and Yang, 2020). In addition, there is evidence that the sensitivity of tumor cells to therapeutic drugs could be restored by inhibiting PI3K/Akt/mTOR pathway, which also prompts the apoptosis of tumor cells (Papadimitrakopoulo, 2012). Thus, many small molecule inhibitors against PI3K/Akt/mTOR signaling pathway have been studied for antitumor use (Alzahrani, 2019).

Natural product is a prolific source with diverse structures and biological properties (Seca and Pinto 2018). Many plants have been used as traditional medicine and the leading compounds for the development of antitumor agents (Agarwal et al., 2019). *L* *-*menthone is a naturally occurring monocyclic monoterpenone found in many essential oils (Tahghighi et al., 2019; Kikowska et al., 2020; Montenegro et al., 2020), especially in peppermint essential oil. It can also be conveniently prepared by oxidation of *L-*menthol, which is the dominant component of peppermint essential oil. *L* *-*menthone and its derivatives have been reported to have a wide range of biological activities, such as antiviral (He et al., 2013), anti-inflammatory (Sun et al., 2014), antidepressant (Xue et al., 2015), antifungal (Boni et al., 2016), and anticancer activities (Bhalla et al., 2013; Chen et al., 2014; Mosbah et al., 2018; Qi et al., 2018). Herein, *L* *-*menthone has received growing interest from medicinal chemists due to their wide range of biological activities.

Many pyrimidine derivatives showed various biological activities such as antibacterial (Xiong et al., 2012), antifungal (Zhang et al., 2019), and anti-inflammatory (Horishny et al., 2021) activities. Especially, pyrimidine derivatives have been reported to show antitumor effect against different human cancers with various mechanisms (Chen N.-Y. et al., 2020; Wang et al., 2020). Meanwhile, many substituted pyrimidines displayed enzymes inhibitory activity (Heffron et al., 2010; Ni et al., 2011), such as PI3K inhibitor (Han et al., 2015), Akt inhibitor (Liu et al., 2017), PI3K and mTOR dual inhibitor (Han et al., 2017), and others. These reports inspire the development of novel pyrimidine-containing antitumor compounds.

On the other hand, urea derivatives are widely applied in medicinal synthesis due to their various biological activities, such as anti-inflammatory (Drexel et al., 2019), antitumor activities (Kang et al., 2019; Kilic-Kurta et al., 2020), and antimicrobial (Lafzi et al., 2021). Recent reports showed that urea derivatives could inhibit cell signaling transduction, such as RAS-RAFMEK-ERK signaling pathway and PI3K/Akt/mTOR pathway (Li et al., 2019), and inhibit protein activity, such as mTORC1 and mTORC2 (Gao et al., 2018). Thus, to design new compounds with urea moiety to explore chemotherapy agents is a feasible strategy.

**FIGURE 1:**
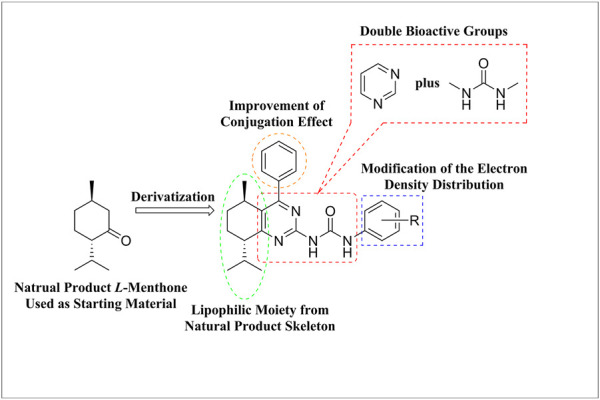
The design strategy of the title compounds.

In addition, it is an effective strategy in drug development to integrate various pharmacophores to obtain novel molecules with synergistic effect. In continuation of our interest in natural product-based antitumor compounds (Li et al., 2017; Wang et al., 2018; Chen Y. et al., 2020; Li et al., 2020; Zhu et al., 2020; Wang et al., 2021), a series of novel menthone-derived compounds containing pyrimidine and urea moieties were designed and synthesized according to the strategy, which was shown in Figure 1. The *in vitro* antitumor activities of the menthone derivatives against the four tumor cell lines were evaluated. The action mechanisms of the target compounds against tumor cell line were studied using a representative compound against Hela cell line by the tests of cell cycle and cell apoptosis, and further predicted by network pharmacology method and molecular docking. Western blot experiments were chosen to confirm the predicted result of the key signaling pathway regulated by this representative compound.

## Materials and Methods

### General

The melting points were recorded on an MP420 automatic melting point apparatus (Hanon Instruments Co., Ltd., Jinan, China) without calibration. Optical rotations were recorded at 20°C on precision scientific WZZ-1 digital automatic polarimeter (Shanghai Precision Technology Instrument Co., Ltd., Shanghai, China). FT-IR spectra were carried out using a Nicolet iS50 FT-IR spectrometer (Thermo Scientific Co., Ltd., United States) by KBr pellet method. NMR spectra were recorded on Bruker Avance III HD 500MHz/600MHz spectrometer (Bruker Co., Ltd., Switzerland) with TMS (tetramethylsilane) as an internal standard in CDCl_3_. High resolution mass spectrometry was determined on ESQUIRE HCT (Bruker Daltonics Co., Ltd., United States) equipped with a TOF/TOF/Ultimate 3000 Nano HPLC. GC analysis was conducted on an Agilent 6890GC (Agilent Technologies Inc., United States) equipped with column HP-1 (30m, 0.530 mm, 0.88μm) and FID. HPLC analysis was conducted on Waters 1525 HPLC instrument (Waters Corp., United States) equipped with Waters 2998 PDA detector and column C18 5μm (4.6mm × 150mm). *L* *-*Menthone (GC purity 98%) was provided by Shanghai Macklin Biochemical Co., Ltd., China. Routine thin-layer chromatography (TLC) was performed on silica gel plates (silica gel GF254 from Qingdao Haiyang Chemical Co., Ltd., Qingdao, China). Preparative flash column chromatography was performed on the 200–300 mesh silica gel (Qingdao Haiyang Chemical Co. Ltd.). Other reagents were purchased from commercial suppliers and used as received.

### General Procedure for Compound 2

*L* *-*Menthone (3.50ml, 20mmol), KOH (1.40g, 25mmol), and anhydrous DMSO (15ml) were mixed and stirred at 20°C for 30min. Then, the mixed solution of benzaldehyde (2.50g, 22mmol) and anhydrous DMSO (10ml) was added dropwise to the reaction system, and the reaction temperature was controlled not to exceed 20°C. After continuous stirring for 4h, the reaction solution was poured into 250ml 4% acetic acid ice-water mixture and then extracted by EtOAc three times. The extracts were combined and washed with saturated sodium chloride and deionized water three times, respectively. Then, the resulting solution was dried with anhydrous Na_2_SO_4_ and concentrated in *vacuum.* The crude product was purified by silica gel column chromatography (EtOAc-petroleum ether = 1:60, v/v) to obtain *L* *-*menthone-derived *α*,*β*-unsaturated ketone, (3R,6S)-2-((*E*)-benzylidene)-6-isopropyl-3-methylcyclohexan-1-one, compound **2**, as a pale yellow liquid in the yield of 93% and HPLC purity of 98.8%. [α]_D_^20^ = - 68.8°(c = 0.20, CH_2_Cl_2_); ^1^H NMR (600MHz, CDCl_3_) *δ* (ppm): 7.38 (d, *J* = 7.2 Hz, 4H), 7.32 (t, *J* = 6.8 Hz, 1H), 7.13 (s, 1H), 3.43 (dt, *J* = 10.8, 6.9 Hz, 1H), 2.58 (m, 1H), 2.26 (ddd, *J* = 11.5, 7.2, 3.5 Hz, 1H), 1.90 (td, *J* = 13.2, 7.6 Hz, 3H), 1.80 (m, 1H), 1.21 (d, *J* = 7.2 Hz, 3H), 0.99 (d, *J* = 7.1 Hz, 3H), 0.93 (d, *J* = 6.8 Hz, 3H); ^13^C NMR (151MHz, CDCl_3_) *δ* (ppm): 205.18, 145.00, 136.07, 132.66, 129.61, 128.55, 128.21, 56.07, 31.79, 30.66, 26.74, 20.54, 19.98, 18.85, 17.97; IR (KBr) *v*: 3438, 3199, 2959, 2939, 1691, 1614, 1488, 1458cm^−1^.

### General Procedure for Compound 3

The mixed solution of compound **2** (1.20 g, 5mmol), guanidine hydrochloride (0.95g, 10mmol), K_2_CO_3_ (4.15g, 30mmol), distilled water (1.20g), and ethanol (30ml) were stirred and refluxed for 9h. After the reaction was completed, the reaction solution was diluted with water and then extracted by EtOAc three times. The extracts were combined and washed with saturated brine to neutrality. The resulting solution was dried over Na_2_SO_4_ and concentrated in *vacuum.* The crude product was purified by silica gel column chromatography (EtOAc-petroleum ether = 1:6, v/v) to obtain *L* *-*menthone-derived pyrimidine, (5R,8S)-8-isopropyl*-*5-methyl*-*4-phenyl*-*5,6,7,8-tetrahydroquinazolin-2-amine, compound **3**, as a white solid in the yield of 28.6% and HPLC purity of 98.2%; [α]_D_^20^ = + 31.3°(c = 0.20, CH_2_Cl_2_); m.p. 144.5–145.6°C; ^1^H NMR (500MHz, CDCl_3_) *δ* (ppm): 7.47–7.38 (m, 5H), 4.87 (s, 2H), 3.04 (td, *J* = 7.0, 3.9 Hz, 1H), 2.92 (m, 1H), 2.70 (td, *J* = 8.8, 3.9 Hz, 1H), 1.76 (d, *J* = 8.0 Hz, 2H), 1.70–1.65 (m, 2H), 1.08 (d, *J* = 7.1 Hz, 3H), 0.85 (d, *J* = 7.0 Hz, 3H), 0.75 (d, *J* = 6.8 Hz, 3H); ^13^C NMR (126MHz, CDCl_3_) *δ* (ppm): 170.04, 167.30, 160.58, 139.35, 128.36, 128.30, 127.99, 123.97, 46.41, 29.59, 28.96, 27.56, 21.45, 20.78, 16.71, 16.44; IR (KBr) *v*: 3438, 3199, 2959, 2939, 1691, 1614, 1488, 1458cm^−1^; HRMS *m/z*: Calcd for C_18_H_23_N_3_ [M+H]^+^: 282.1965, found: 282.1954.

### General Procedure for the Title Compounds 4a-4s

The mixture of compound **3** (0.28 g, 1mmol), substituted phenyl isocyanate (1.3mmol), and toluene (10ml) were stirred at room temperature for 8–12h. After compound **3** was fully reacted, the precipitate was filtered, and dissolved in EtOAc. The resulting solution was concentrated in *vacuum.* The crude product was purified by silica gel column chromatography (EtOAc*-*petroleum ether = 1:10, *v*/*v*) to afford *L* *-*menthone-derived pyrimidine-urea, compounds **4a-4s**, as a white solid.

**1-(2′-Methylphenyl)-3-((5R,8S)-8-isopropyl*-*5-methyl*-*4-phenyl*-*5,6,7,8-tetrahydroquinazolin-2-yl)urea (4a).** White solid, Yield: 80%; HPLC purity: 95.3%; [α]_D_^20^ = + 10.2°(c = 0.11, CHCl_3_); m.p. 191.2–193.3°C; ^1^H NMR (500MHz, CDCl_3_) *δ* (ppm): 11.08 (s, 1H), 8.08 (d, *J* = 7.9 Hz, 1H), 7.57–7.46 (m, 4H), 7.45–7.40 (m, 2H), 7.21 (t, *J* = 7.8 Hz, 1H), 7.07 (d, *J* = 7.5 Hz, 1H), 6.99 (t, *J* = 7.4 Hz, 1H), 3.12 (m, 1H), 2.93 (ddd, *J* = 13.9, 8.9, 5.4 Hz, 1H), 2.83 (tt, *J* = 6.0, 3.2 Hz, 1H), 1.89 (s, 1H), 1.84–1.80 (m, 3H), 1.77 (s, 1H), 1.77–1.73 (m, 1H), 1.68 (d, *J* = 8.5 Hz, 1H), 1.10 (d, *J* = 7.1 Hz, 3H), 0.92 (d, *J* = 7.0 Hz, 3H), 0.77 (d, *J* = 6.8 Hz, 3H); ^13^C NMR (126MHz, CDCl_3_) *δ* (ppm): 155.06, 152.21, 138.18, 136.92, 131.04, 130.18, 128.97, 128.89, 128.62, 128.46, 128.25, 128.04, 127.77, 126.63, 123.70, 46.62, 29.84, 28.66, 27.80, 21.30, 20.67, 18.03, 16.80, 16.33; IR (KBr) *v*: 3203, 3122, 3045, 2960, 2941, 2866, 1692, 1614, 1590, 1561, 1543, 1489, 1458, 1376cm^−1^; HRMS *m/z*: Calcd for C_26_H_30_N_4_O [M+H]^+^: 415.2492, found: 415.2492.

**1-(3′-Methylphenyl)-3-((5R,8S)-8-isopropyl*-*5-methyl*-*4-phenyl*-*5,6,7,8-tetrahydroquinazolin-2-yl)urea (4b).** White solid, Yield: 83%; HPLC purity: 97.2%; [α]_D_^20^ = + 11.0°(c = 0.10, CHCl_3_); m.p. 170.8–173.7°C; ^1^H NMR (500MHz, CDCl_3_) *δ* (ppm): 11.52 (s, 1H), 7.48 (d, *J* = 3.5Hz, 5H), 7.37–7.28 (m, 3H), 7.19 (t, *J* = 7.8Hz, 1H), 6.88 (d, *J* = 7.5Hz, 1H), 3.25 (m, 1H), 2.99 (tt, *J* = 10.6, 5.4Hz, 1H), 2.85 (s, 1H), 2.33 (s, 3H), 1.90–1.80 (m, 3H), 1.79–1.75 (m, 1H), 1.15 (d, *J* = 7.0Hz, 3H), 0.89 (d, *J* = 7.0Hz, 3H), 0.80 (d, *J* = 6.8Hz, 3H); ^13^C NMR (126MHz, CDCl_3_) *δ* (ppm): 154.98, 151.94, 138.91, 138.57, 138.31, 129.15, 128.97, 128.59, 128.12, 127.76, 124.22, 120.39, 116.68, 46.82, 29.85, 28.66, 27.69, 21.69, 21.46, 20.75, 16.73, 16.24; IR (KBr) *v*: 3203, 3116, 3054, 2958, 2923, 2869, 1687, 1614, 1566, 1543, 1486, 1379cm^−1^; HRMS *m/z*: Calcd for C_26_H_30_N_4_O [M+H]^+^: 415.2492, found: 415.2487.

**1-(4′-Methylphenyl)-3-((5R,8S)-8-isopropyl*-*5-methyl*-*4-phenyl*-*5,6,7,8-tetrahydroquinazolin-2-yl)urea (4c).** White solid, Yield: 88%; HPLC purity: 96.8%; [α]_D_^20^ = + 12.6°(c = 0.11, CHCl_3_); m.p. 179.8–181.2°C; ^1^H NMR (500MHz, CDCl_3_) *δ* (ppm): 11.47 (s, 1H), 7.50 (d, *J* = 3.9Hz, 5H), 7.41 (d, *J* = 8.4Hz, 3H), 7.13 (d, *J* = 8.0Hz, 2H), 3.27 (q, *J* = 6.3, 5.5Hz, 1H), 3.04–2.94 (m, 1H), 2.86 (td, *J* = 8.6, 8.0, 4.0Hz, 1H), 2.33 (s, 3H), 1.92–1.81 (m, 3H), 1.28 (s, 1H), 1.16 (d, *J* = 7.0Hz, 3H), 0.91 (d, *J* = 7.0Hz, 3H), 0.81 (d, *J* = 6.9Hz, 3H); ^13^C NMR (126MHz, CDCl_3_) *δ* (ppm): 155.00, 152.01, 138.32, 136.04, 132.93, 129.61, 129.11, 128.58, 128.09, 127.68, 119.75, 46.78, 29.83, 28.65, 27.68, 21.45, 20.93, 20.75, 16.73, 16.27; IR (KBr) *v*: 3197, 3119, 3033, 2956, 2921, 1694, 1610, 1562, 1544, 1514, 1499, 1371cm^−1^; HRMS *m/z*: Calcd for C_26_H_30_N_4_O [M+H]^+^: 415.2492, found: 415.2485.

**1-(4′-Fluorophenyl)-3-((5R,8S)-8-isopropyl*-*5-methyl*-*4-phenyl*-*5,6,7,8-tetrahydroquinazolin-2-yl)urea (4d).** White solid, Yield: 86%; HPLC purity: 98.0%; [α]_D_^20^ = + 5.7°(c = 0.11, CHCl_3_); m.p. 191.5–192.9°C; ^1^H NMR (500MHz, CDCl_3_) *δ* (ppm): 11.56 (s, 1H), 7.54–7.43 (m, 8H), 7.01 (t, *J* = 8.5Hz, 2H), 3.27 (m, 1H), 2.96 (m, 1H), 2.86 (td, *J* = 8.5, 7.5, 4.1Hz, 1H), 1.85 (td, *J* = 12.8, 11.7, 3.8Hz, 3H), 1.80 (s, 1H), 1.16 (d, *J* = 7.0Hz, 3H), 0.90 (d, *J* = 7.0Hz, 3H), 0.81 (d, *J* = 6.8Hz, 3H); ^13^C NMR (126MHz, CDCl_3_) *δ* (ppm): 159.94, 158.02, 154.91, 152.12, 138.27, 134.68, 129.19, 128.62, 128.07, 127.85, 121.23, 121.17, 115.77, 115.59, 46.80, 29.85, 28.63, 27.68, 21.44, 20.76, 16.76, 16.28; IR (KBr) *v*): 3197, 3116, 3042, 2960, 2929, 2870, 1689, 1614, 1565, 1542, 1510, 1376cm^−1^; HRMS *m/z*: Calcd for C_25_H_27_FN_4_O [M+H]^+^: 419.2242, found: 419.2235.

**1-(2′-Chlorophenyl)-3-((5R,8S)-8-isopropyl*-*5-methyl*-*4-phenyl*-*5,6,7,8-tetrahydroquinazolin-2-yl)urea (4e).** White solid, Yield: 78%; HPLC purity: 95.6%; [α]_D_^20^ = + 50.2°(c = 0.11, CHCl_3_); m.p. 177.5–180.5°C; ^1^H NMR (500MHz, CDCl_3_) *δ* (ppm): 11.66 (s, 1H), 8.39 (d, *J* = 8.2Hz, 1H), 7.56–7.46 (m, 4H), 7.43 (dd, *J* = 6.8, 2.9Hz, 2H), 7.29–7.23 (m, 2H), 6.98 (t, *J* = 7.6Hz, 1H), 3.13 (m, 1H), 2.98 (m, 1H), 2.87 (dt, *J* = 9.4, 4.7Hz, 1H), 1.81 (t, *J* = 9.2Hz, 3H), 1.77–1.72 (m, 1H), 1.10 (d, *J* = 7.0Hz, 3H), 0.93 (d, *J* = 7.0Hz, 3H), 0.75 (d, *J* = 6.8Hz, 3H); ^13^C NMR (126MHz, CDCl_3_) *δ* (ppm): 154.78, 152.01, 138.03, 136.01, 129.12, 128.87, 128.39, 128.18, 128.15, 127.44, 123.85, 123.49, 122.14, 46.48, 30.00, 28.63, 27.84, 21.20, 20.70, 16.76, 16.33; IR (KBr) *v*: 3203, 3140, 3036, 2958, 2876, 1689, 1592, 1557, 1538, 1498, 1442, 1381cm^−1^; HRMS *m/z*: Calcd for C_25_H_27_ClN_4_O [M+H]^+^: 435.1946, found: 435.1945.

**1-(3′-Chlorophenyl)-3-((5R,8S)-8-isopropyl*-*5-methyl*-*4-phenyl*-*5,6,7,8-tetrahydroquinazolin-2-yl)urea (4f).** White solid, Yield: 80%; HPLC purity: 98.2%; [α]_D_^20^ = + 25.2°(c = 0.12, CHCl_3_); m.p. 170.8–173.7°C; ^1^H NMR (500MHz, CDCl_3_) *δ* (ppm): 11.74 (s, 1H), 7.61 (s, 1H), 7.51 (q, *J* = 8.1, 7.0Hz, 6H), 7.43–7.31 (m, 1H), 7.22 (t, *J* = 8.0Hz, 1H), 7.04 (d, *J* = 7.9Hz, 1H), 3.33–3.24 (m, 1H), 2.98 (m, 1H), 2.88 (dd, *J* = 9.9, 4.4Hz, 1H), 1.92–1.82 (m, 3H), 1.79 (dd, *J* = 12.2, 4.1Hz, 1H), 1.18 (d, *J* = 7.1Hz, 3H), 0.91 (d, *J* = 7.0Hz, 3H), 0.82 (d, *J* = 6.8Hz, 3H); ^13^C NMR (126MHz, CDCl_3_) *δ* (ppm): 154.80, 151.85, 139.91, 138.20, 134.71, 130.08, 129.26, 128.65, 128.08, 128.01, 123.34, 119.68, 117.45, 46.83, 29.91, 28.62, 27.69, 21.45, 20.72, 16.74, 16.24; IR (KBr) *v*: 3215, 3134, 3057, 2959, 2870, 1694, 1595, 1560, 1541, 1480, 1421, 1379cm^−1^; HRMS *m/z*: Calcd for C_25_H_27_ClN_4_O [M+H]^+^: 435.1946, found: 435.1942.

**1-(4′-Chlorophenyl)-3-((5R,8S)-8-isopropyl*-*5-methyl*-*4-phenyl*-*5,6,7,8-tetrahydroquinazolin-2-yl)urea (4g).** White solid, Yield: 70%; HPLC purity: 96.1%; [α]_D_^20^ = + 18.8°(c = 0.11, CHCl_3_); m.p. 183.6–185.5°C; ^1^H NMR (500MHz, CDCl_3_) *δ* (ppm): 11.67 (s, 1H), 7.61 (s, 1H), 7.54–7.44 (m, 7H), 7.27 (d, *J* = 8.5Hz, 2H), 3.28 (q, *J* = 6.3, 5.7Hz, 1H), 3.00–2.92 (m, 1H), 2.87 (m, 1H), 1.85 (td, *J* = 12.9, 12.0, 3.9Hz, 3H), 1.75 (dd, *J* = 14.0, 6.6Hz, 1H), 1.16 (d, *J* = 7.0Hz, 3H), 0.91 (d, *J* = 7.0Hz, 3H), 0.81 (d, *J* = 6.8Hz, 3H); ^13^C NMR (126MHz, CDCl_3_) *δ* (ppm): 154.84, 151.99, 138.23, 137.30, 129.21, 129.06, 128.60, 128.24, 128.19, 128.06, 127.92, 120.77, 46.77, 29.87, 28.60, 27.66, 21.42, 20.74, 16.75, 16.26; IR (KBr) *v*: 3203, 3108, 3030, 2957, 2932, 2864, 1697, 1611, 1602, 1583, 1560, 1542, 1493, 1371cm^−1^; HRMS *m/z*: Calcd for C_25_H_27_ClN_4_O [M+H]^+^: 435.1946, found: 435.1940.

**1-(2′-Bromophenyl)-3-((5R,8S)-8-isopropyl*-*5-methyl*-*4-phenyl*-*5,6,7,8-tetrahydroquinazolin-2-yl)urea (4h).** White solid, Yield: 76%; HPLC purity: 96.7%; [α]_D_^20^ = + 19.2°(c = 0.11, CHCl_3_); m.p. 184.2–187.1°C; ^1^H NMR (500MHz, CDCl_3_) *δ* (ppm): 11.50 (s, 1H), 8.32 (d, *J* = 8.2Hz, 1H), 7.52 (s, 1H), 7.50–7.44 (m, 4H), 7.42 (dd, *J* = 6.7, 3.1Hz, 2H), 7.30 (d, *J* = 8.0Hz, 1H), 6.92 (q, *J* = 7.3Hz, 1H), 3.15–3.06 (m, 1H), 2.98 (m, 1H), 2.87 (dt, *J* = 9.6, 4.6Hz, 1H), 1.82–1.73 (m, 3H), 1.65 (s, 1H), 1.10 (d, *J* = 7.1Hz, 3H), 0.93 (d, *J* = 7.0Hz, 3H), 0.75 (d, *J* = 6.8Hz, 3H); ^13^C NMR (126MHz, CDCl_3_) *δ* (ppm): 154.74, 152.09, 138.07, 137.24, 132.69, 132.46, 128.86, 128.56, 128.39, 128.26, 128.18, 127.97, 124.51, 122.91, 46.46, 29.99, 28.64, 27.85, 21.22, 20.68, 16.74, 16.30; IR (KBr) *v*: 3212, 3137, 3033, 2960, 2923, 2870, 1690, 1581, 1569, 1556, 1536, 1498, 1438, 1421, 1380cm^−1^; HRMS *m/z*: Calcd for C_25_H_27_BrN_4_O [M+H]^+^: 479.1441, found: 479.1462.

**1-(4′-Bromophenyl)-3-((5R,8S)-8-isopropyl*-*5-methyl*-*4-phenyl*-*5,6,7,8-tetrahydroquinazolin-2-yl)urea (4i).** White solid, Yield: 82%; HPLC purity: 95.8%; [α]_D_^20^ = + 20.2°(c = 0.11, CHCl_3_); m.p. 188.8–190.1°C; ^1^H NMR (500MHz, CDCl_3_) *δ* (ppm): 11.66 (s, 1H), 7.53 (s, 1H), 7.52–7.47 (m, 4H), 7.42 (d, *J* = 9.5 Hz, 5H), 3.32–3.23 (m, 1H), 2.96 (m, 1H), 2.86 (m, 1H), 1.86–1.78 (m, 3H), 1.67–1.61 (m, 1H), 1.16 (d, *J* = 7.0Hz, 3H), 0.90 (d, *J* = 7.0Hz, 3H), 0.81 (d, *J* = 6.8Hz, 3H); ^13^C NMR (126MHz, CDCl_3_) *δ* (ppm): 154.81, 151.90, 138.22, 137.80, 132.04, 129.24, 128.64, 128.07, 127.98, 121.16, 115.85, 46.82, 29.89, 28.62, 27.69, 21.44, 20.75, 16.76, 16.27; IR (KBr) *v*: 3209, 3114, 3030, 2958, 2926, 2870, 1696, 1607, 1591, 1583, 1559, 1542, 1488, 1371cm^−1^; HRMS *m/z*: Calcd for C_25_H_27_BrN_4_O [M+H]^+^: 479.1441, found: 479.1446.

**1-(2′-(Trifluoromethyl)phenyl)-3-((5R,8S)-8-isopropyl*-*5-methyl*-*4-phenyl*-*5,6,7,8-tetrahydroquinazolin-2-yl)urea (4j).** White solid, Yield: 80%; HPLC purity: 95.2%; [α]_D_^20^ = + 54.7°(c = 0.14, CHCl_3_); m.p. 171.2–174.2°C; ^1^H NMR (500 MHz, CDCl_3_) *δ* (ppm): 11.29 (s, 1H), 8.04 (d, *J* = 8.2Hz, 1H), 7.57 (h, *J* = 9.6, 8.7Hz, 3H), 7.47 (dd, *J* = 5.3, 1.9Hz, 3H), 7.41 (dd, *J* = 6.8, 2.9Hz, 2H), 7.22 (t, *J* = 7.7Hz, 1H), 3.13 (m, 1H), 2.96–2.75 (m, 2H), 1.81–1.72 (m, 3H), 1.69–1.61 (m, 1H), 1.07 (d, *J* = 6.8Hz, 3H), 0.91 (d, *J* = 7.0Hz, 3H), 0.74 (d, *J* = 6.7 Hz, 3H); ^13^C NMR (126MHz, CDCl_3_) *δ* (ppm): 154.81, 152.58, 137.94, 135.65, 132.49, 128.81, 128.34, 128.26, 128.05, 126.77, 126.09, 126.04, 126.00, 124.40, 122.45, 46.26, 30.03, 28.60, 27.82, 21.14, 20.63, 16.76, 16.32; IR (KBr) *v*: 3197, 3143, 3066, 3042, 2961, 2938, 2876, 1684, 1620, 1588, 1566, 1535, 1481, 1455, 1371cm^−1^; HRMS *m/z*: Calcd for C_26_H_27_F_3_N_4_O [M+H]^+^: 469.2210, found: 469.2211.

**1-(3′-(Trifluoromethyl)phenyl)-3-((5R,8S)-8-isopropyl*-*5-methyl*-*4-phenyl*-*5,6,7,8-tetrahydroquinazolin-2-yl)urea (4k).** White solid, Yield: 76%; HPLC purity: 97.4%; [α]_D_^20^ = + 16.7°(c = 0.11, CHCl_3_); m.p. 175.4–176.3°C; ^1^H NMR (500MHz, CDCl_3_) *δ* (ppm): 11.92 (s, 1H), 7.87 (s, 1H), 7.66 (s, 1H), 7.52 (d, *J* = 6.8Hz, 5H), 7.43 (dd, *J* = 9.8, 6.3Hz, 2H), 7.32 (d, *J* = 7.8Hz, 1H), 3.30 (d, *J* = 8.4Hz, 1H), 2.99 (m, 1H), 2.88 (q, *J* = 7.2, 4.9Hz, 1H), 1.85 (td, *J* = 13.3, 9.6Hz, 3H), 1.66–1.59 (m, 1H), 1.18 (d, *J* = 7.1Hz, 3H), 0.92 (d, *J* = 7.0Hz, 3H), 0.83 (d, *J* = 6.8Hz, 3H); ^13^C NMR (126MHz, CDCl_3_) *δ* (ppm): 154.78, 151.92, 139.29, 138.18, 131.28, 129.76, 129.31, 128.84, 128.68, 128.12, 128.07, 125.18, 123.01, 122.49, 119.83, 116.11, 116.08, 46.88, 29.85, 28.62, 27.70, 21.48, 20.64, 16.69, 16.21; IR (KBr) *v*: 3206, 3120, 3063, 2963, 2926, 2876, 1696, 1614, 1566, 1541, 1486, 1373cm^−1^; HRMS *m/z*: Calcd for C_26_H_27_F_3_N_4_O [M+H]^+^: 469.2210, found: 469.2211.

**1-(4′-(Trifluoromethyl)phenyl)-3-((5R,8S)-8-isopropyl*-*5-methyl*-*4-phenyl*-*5,6,7,8-tetrahydroquinazolin-2-yl)urea (4l).** White solid, Yield: 70%; HPLC purity: 98.3%; [α]_D_^20^ = + 11.2°(c = 0.10, CHCl_3_); m.p. 204.7–206.2°C; ^1^H NMR (500MHz, CDCl_3_) *δ* (ppm): 11.89 (s, 1H), 7.64–7.43 (m, 10H), 3.33–3.22 (m, 1H), 2.97 (m, 1H), 2.87 (s, 1H), 1.87–1.80 (m, 3H), 1.61 (d, *J* = 3.2Hz, 1H), 1.17 (d, *J* = 7.0Hz, 3H), 0.91 (d, *J* = 7.0Hz, 3H), 0.81 (d, *J* = 6.9Hz, 3H); ^13^C NMR (126MHz, CDCl_3_) *δ* (ppm): 154.74, 151.89, 141.85, 138.18, 129.32, 128.68, 128.18, 128.07, 126.40, 123.33, 119.14, 46.86, 29.85, 28.61, 27.71, 21.44, 20.76, 16.77, 16.27; IR (KBr) *v*: 3206, 3137, 3111, 3039, 2960, 2929, 2870, 1697, 1603, 1564, 1541, 1504, 1488, 1376cm^−1^; HRMS *m/z*: Calcd for C_26_H_27_F_3_N_4_O [M+H]^+^: 469.2210, found: 469.2202.

**1-(3′,5′-Dimethylphenyl)-3-((5R,8S)-8-isopropyl*-*5-methyl*-*4-phenyl*-*5,6,7,8-tetrahydroquinazolin-2-yl)urea (4m).** White solid, Yield: 80%; HPLC purity: 95.9%; [α]_D_^20^ = + 17.2°(c = 0.11, CHCl_3_); m.p. 166.9–169.1°C; ^1^H NMR (500MHz, CDCl_3_) *δ* (ppm): 11.54 (s, 1H), 7.51 (s, 5H), 7.36 (s, 1H), 7.18 (s, 2H), 6.74 (s, 1H), 3.28 (d, *J* = 7.3Hz, 1H), 3.03 (m, 1H), 2.87 (q, *J* = 7.3, 5.1Hz, 1H), 2.31 (s, 6H), 1.91–1.82 (m, 3H), 1.69 (s, 1H), 1.18 (d, *J* = 7.1Hz, 3H), 0.91 (d, *J* = 7.0Hz, 3H), 0.83 (d, *J* = 6.8Hz, 3H); ^13^C NMR (126MHz, CDCl_3_) *δ* (ppm): 154.99, 151.89, 138.73, 138.49, 138.32, 129.12, 128.96, 128.56, 128.13, 127.70, 125.10, 117.38, 46.80, 29.84, 28.66, 27.67, 21.57, 21.46, 20.72, 16.71, 16.19; IR (KBr) *v*: 3206, 3131, 3021, 2957, 2920, 2867, 1692, 1617, 1566, 1548, 1485, 1388cm^−1^; HRMS *m/z*: Calcd for C_27_H_32_N_4_O [M+H]^+^: 429.2649, found: 429.2647.

**1-(2′,4′-Difluorophenyl)-3-((5R,8S)-8-isopropyl*-*5-methyl*-*4-phenyl*-*5,6,7,8-tetrahydroquinazolin-2-yl)urea (4n).** White solid, Yield 75%; Purity 97.0%; [α]_D_^20^ = + 25.2°(c = 0.11, CHCl_3_); m.p. 179.7–182.2°C; ^1^H NMR (500MHz, CDCl_3_) *δ* (ppm): 11.61 (s, 1H), 8.31 (td, *J* = 9.0, 5.9 Hz, 1H), 7.54–7.45 (m, 6H), 6.86 (td, *J* = 13.3, 11.2, 7.1Hz, 2H), 3.23 (m, 1H), 3.00–2.92 (m, 1H), 2.88 (dt, *J* = 8.9, 4.4Hz, 1H), 1.86–1.80 (m, 3H), 1.68–1.64 (m, 1H), 1.12 (d, *J* = 7.0Hz, 3H), 0.91 (d, *J* = 7.0Hz, 3H), 0.77 (d, *J* = 6.9Hz, 3H); ^13^C NMR (126MHz, CDCl_3_) *δ* (ppm): 159.10 (d, *J* = 11.1Hz), 157.15 (d, *J* = 11.3Hz), 154.81, 153.79 (d, *J* = 11.7Hz), 151.99, 138.04, 129.05, 128.51, 128.12, 123.44 (dd, *J* = 10.8, 4.0Hz), 122.70 (d, *J* = 9.2Hz), 111.37–110.73 (m), 104.02–102.92 (m), 46.59, 29.86 (d, *J* = 5.0Hz), 28.58, 27.75, 21.23, 20.75, 16.77, 16.34; IR (KBr) *v*: 3209, 3152, 3117, 3027, 2960, 2926, 2870, 1694, 1611, 1565, 1542, 1503, 1371cm^−1^; HRMS *m/z*: Calcd for C_25_H_26_F_2_N_4_O [M+H]^+^: 437.2147, found: 437.2149.

**1-(3′,4′-Dichlorophenyl)-3-((5R,8S)-8-isopropyl*-*5-methyl*-*4-phenyl*-*5,6,7,8-tetrahydroquinazolin-2-yl)urea (4o).** White solid, Yield: 72%; HPLC purity: 95%; [α]_D_^20^ = + 10.2°(c = 0.12, CHCl_3_); m.p. 178.7–180.8°C; ^1^H NMR (500MHz, CDCl_3_) *δ* (ppm): 11.81 (s, 1H), 7.71 (s, 1H), 7.55–7.43 (m, 6H), 7.35 (d, *J* = 6.6 Hz, 2H), 3.29 (dq, *J* = 10.1, 6.0 Hz, 1H), 2.95 (m, 1H), 2.87 (m, 1H), 1.84 (dt, *J* = 15.8, 6.3Hz, 3H), 1.64–1.61 (m, 1H), 1.17 (d, *J* = 7.1Hz, 3H), 0.91 (d, *J* = 7.0Hz, 3H), 0.81 (d, *J* = 6.8Hz, 3H); ^13^C NMR (126MHz, CDCl_3_) *δ* (ppm): 154.69, 151.78, 138.29, 138.16, 132.78, 130.61, 129.34, 128.69, 128.17, 128.06, 126.36, 121.17, 118.68, 46.86, 29.92, 28.60, 27.69, 21.46, 20.72, 16.75, 16.24; IR (KBr) *v*: 3215, 3146, 3030, 2959, 2932, 2870, 1694, 1592, 1566, 1538, 1476, 1371cm^−1^; HRMS *m/z*: Calcd for C_25_H_26_Cl_2_N_4_O [M+H]^+^: 469.1556, found: 469.1556.

**1-(3′,5′-Bis(trifluoromethyl)phenyl)-3-((5R,8S)-8-isopropyl*-*5-methyl*-*4-phenyl*-*5,6,7,8-tetrahydroquinazolin-2-yl)urea (4p).** White solid, Yield: 80%; HPLC purity: 96.5%; [α]_D_^20^ = + 26.2°(c = 0.10, CHCl_3_); m.p. 160.5–162.0°C; ^1^H NMR (500MHz, CDCl_3_): *δ* (ppm) 12.30 (s, 1H), 8.02 (s, 2H), 7.58–7.49 (m, 7H), 3.33 (s, 1H), 3.04–2.95 (m, 1H), 2.91 (d, *J* = 10.8Hz, 1H), 1.86 (dt, *J* = 14.0, 6.0Hz, 3H), 1.62 (d, *J* = 3.4Hz, 1H), 1.19 (d, *J* = 7.1Hz, 3H), 0.93 (d, *J* = 7.0Hz, 3H), 0.83 (d, *J* = 6.8Hz, 3H); ^13^C NMR (126MHz, CDCl_3_) *δ* (ppm): 154.58, 151.81, 140.28, 138.06, 132.57, 132.30, 132.03, 129.47, 128.75, 128.46, 128.03, 126.61, 124.44, 122.27, 120.10, 118.97, 116.47, 46.93, 30.05, 28.58, 27.71, 21.50, 20.57, 16.67, 16.15; IR (KBr) *v*: 3224, 3164, 3108, 2962, 2873, 1706, 1625, 1583, 1565, 1545, 1473, 1450, 1389cm^−1^; HRMS *m/z*: Calcd for C_27_H_26_F_6_N_4_O [M+H]^+^: 537.2085, found: 537.2084.

**1-(4′-Chloro-3′-(trifluoromethyl)phenyl)-3-((5R,8S)-8-isopropyl*-*5-methyl*-*4-phenyl*-*5,6,7,8-tetrahydroquinazolin-2-yl)urea (4q).** White solid, Yield: 76%; HPLC purity: 95.2%; [α]_D_^20^ = + 19.2°(c = 0.11, CHCl_3_); m.p. 192.1–193.5°C; ^1^H NMR (500MHz, CDCl_3_) *δ* (ppm): 12.00 (s, 1H), 7.92 (s, 1H), 7.63 (s, 1H), 7.55–7.47 (m, 6H), 7.44 (d, *J* = 8.8Hz, 1H), 3.31 (d, *J* = 7.2Hz, 1H), 2.97 (m, 1H), 2.88 (m, 1H), 1.84 (dt, *J* = 13.3, 6.1Hz, 3H), 1.65 (s, 1H), 1.17 (d, *J* = 7.0Hz, 3H), 0.92 (d, *J* = 7.0Hz, 3H), 0.82 (d, *J* = 6.8Hz, 3H); ^13^C NMR (126MHz, CDCl_3_) *δ* (ppm): 154.66, 151.89, 138.11, 137.71, 132.20, 129.36, 128.83, 128.71, 128.58, 128.26, 128.02, 125.61, 123.85, 123.31, 121.68, 119.51, 118.21 (d, *J* = 5.5Hz), 46.87, 29.99, 28.59, 27.70, 21.47, 20.63, 16.68, 16.20; IR (KBr) *v*: 3206, 3137, 3102, 3042, 2961, 2935, 2873, 1695, 1603, 1560, 1540, 1482, 1371cm^−1^; HRMS *m/z*: Calcd for C_26_H_26_ClF_3_N_4_O [M+H]^+^: 503.1820, found: 503.1824.

**1-(4′-Cyanophenyl)-3-((5R,8S)-8-isopropyl*-*5-methyl*-*4-phenyl*-*5,6,7,8-tetrahydroquinazolin-2-yl)urea (4r).** White solid, Yield: 80%; HPLC purity: 95.5%; [α]_D_^20^ = + 16.2°(c = 0.11, CHCl_3_); m.p. 216.4–218.4°C; ^1^H NMR (500MHz, CDCl_3_) *δ* (ppm): 12.03 (s, 1H), 7.60 (d, *J* = 15.8Hz, 5H), 7.55–7.48 (m, 5H), 3.28 (t, *J* = 8.1 Hz, 1H), 3.01–2.92 (m, 1H), 2.87 (t, *J* = 6.8 Hz, 1H), 1.89–1.81 (m, 3H), 1.70–1.63 (m, 1H), 1.16 (d, *J* = 7.0Hz, 3H), 0.91 (d, *J* = 7.1Hz, 3H), 0.81 (d, *J* = 6.7Hz, 3H); ^13^C NMR (126MHz, CDCl_3_) *δ* (ppm): 154.60, 151.79, 142.90, 138.09, 133.39, 129.38, 128.69, 128.34, 128.03, 119.37, 119.25, 106.11, 46.84, 29.83, 28.55, 27.69, 21.41, 20.74, 16.78, 16.26; IR (KBr) *v*: 3209, 3155, 3036, 2960, 2929, 2873, 2227, 1698, 1593, 1568, 1538, 1510, 1500, 1489, 1372cm^−1^; HRMS *m/z*: Calcd for C_26_H_26_ClF_3_N_4_O [M+H]^+^: 426.2288, found: 426.2288.

**1-(4′-Nitrophenyl)-3-((5R,8S)-8-isopropyl*-*5-methyl*-*4-phenyl*-*5,6,7,8-tetrahydroquinazolin-2-yl)urea (4s).** White solid, Yield: 82%; HPLC purity: 97.3%; [α]_D_^20^ = + 19.2°(c = 0.10, CHCl_3_); m.p. 221.4–224.0°C; ^1^H NMR (500MHz, CDCl_3_) *δ* (ppm): 12.20 (s, 1H), 8.19 (d, *J* = 8.6Hz, 2H), 7.64 (d, *J* = 12.8Hz, 3H), 7.56–7.46 (m, 5H), 3.30 (m, 1H), 2.97 (m, 1H), 2.92–2.82 (m, 1H), 1.92–1.83 (m, 3H, C_3_-H), 1.82–1.77 (m, 1H), 1.17 (d, *J* = 7.0Hz, 3H), 0.92 (d, *J* = 7.0Hz, 3H), 0.81 (d, *J* = 6.8Hz, 3H); ^13^C NMR (126MHz, CDCl_3_) *δ* (ppm): 154.55, 151.75, 144.83, 142.96, 138.06, 129.44, 128.73, 128.47, 128.17, 128.04, 125.28, 118.79, 46.87, 29.98, 28.55, 27.71, 21.41, 20.75, 16.80, 16.26; IR (KBr) *v*: 3206, 3120, 3039, 2958, 2923, 2870, 1701, 1614, 1600, 1561, 1543, 1506, 1371cm^−1^; HRMS *m/z*: Calcd for C_26_H_26_ClF_3_N_4_O [M+H]^+^: 446.2187, found: 446.2188.

### Cytotoxicity Assay

The antitumor activity of the target compounds and the positive control, 5-FU, were evaluated against the human cancer cell lines A549, Hela, MCF-7, and MGC-803 by MTT assay. The cell lines were grown on 96-well micro-plates at a density of 5 × 10^3^ cells per well in DMEM (Dulbecco’s modified eagle medium) with 10% FBS (fetal bovine serum). The plates were incubated and maintained at 37°C with 5% CO_2_. The cells were then exposed to different concentrations of the synthesized compounds and 5-FU and incubated for another 48h. Control wells were formed by culture media with the maximum concentration of DMSO used in each assay (1‰) (Ruvinsky and Meyuhas, 2006). A total of 20µL of MTT was added and incubated for about 4h. The medium was thrown away and replaced by 150ml DMSO. The absorbance (OD) value was measured at 490nm using an enzyme labeling instrument. Each experiment was repeated at least three times to obtain the mean values (Hafez and EL-Gazzar, 2017).

### Cell Cycle Analysis

The Hela cell line was treated with different concentrations of compound **4i**. After incubation for 48h, the treated cells were washed twice with ice-cold phosphate buffer saline (PBS), fixed, and permeabilized with ice-cold 70% ethanol (−20°C) overnight. The cells were treated with 100µg/ml rnase A at 37°C for 30min, then washed with ice-cold PBS, and finally stained with 1mg/ml propidium iodide (PI) (BD, Pharmingen) in the dark at 4°C for 30min. The data were analyzed by the system software (Cell Quest; BD Biosciences, Becton Dickinson FACSAriaIII, New York, United States).

### Apoptosis Analysis

Hela cells were seeded at a concentration of 2 × 10^6^ cells/mL of the DMEM medium with 10% FBS on 6-well plates to the final volume of 2ml, and then treated with compound **4i** at different concentrations for 48h. After 48h, the cells were collected and washed twice with PBS, then resuspended in 100µL 1 × binding buffer. The cells were subjected to 5µL of FITC Annexin V and 5µL propidium iodide (PI) staining using an annexin-V FITC apoptosis kit (BD, Pharmingen) and incubated for 30min at RT (25°C) in the dark. Then, 100µL 1× binding buffer was added. The apoptosis ratio was quantified by system software (Cell Quest; BD Biosciences, Becton Dickinson FACSAriaIII, New York, United States).

### Network Pharmacology Analysis

#### Targets Prediction of 4i

The structure of **4i** was imported into the database of PharmMapper Server (http://www.lilab-ecust.cn/pharmmapper/) for prediction (Yi et al., 2017). The results, whose scores of Norm Fit were greater than zero, were chosen as the predicted targets of **4i**. The targets, including the gene names and gene ID, were further extracted using UniProtKB (http://www.uniprot.org) (Wang et al., 2017).

#### Collection of Potential Targets for Cervical Cancer Hela Cell Line

“Hela human cervical cancer Hela cell line” were used as keywords to retrieve cervical cancer Hela cell line associated targets in Online Mendelian Inheritance in Man database (OMIM, https://omim.org) (Bateman et al., 2021) and Genecards database (https://www.genecards.org) (Hamosh et al., 2005). On mapping cervical cancer Hela cell line associated targets and predicted targets of **4i**, the potential targets of **4i** for treatment of cervical cancer Hela cell line were finally obtained.

#### Construction and Analysis of Protein-Protein Interaction Network

The potential targets gene list was imported into the STRING database (https://string-db.org) (Stelzer et al., 2016), with the species as ‘‘Homo sapiens” and a confidence score greater than 0.4, to obtain the data of PPI and the visualized PPI network. The topological parameters of the PPI network were analyzed with the Network Analyzer tool of Cytoscape. The targets whose degrees were greater than 1.9 times median were selected as the core targets.

### Gene Ontology and Kyoto Encyclopedia of Genes and Genomes Enrichment Analysis

The biological functions of the target genes were assessed through gene ontology (GO) and Kyoto Encyclopedia of Genes and Genomes (KEGG) pathways enrichment analysis. The R package, “DOSE”, “clusterProfiler”, “enrichplot”, and “org.Hs.eg.db”, were used to perform the enrichment analyses. The bar graphs of results were drawn by the R package “ggplot2”.

### Molecular Docking Analysis

Molecular docking was performed using AutoDock 4.2.6 software (ADT) according to the report (Szklarczyk et al., 2019). The structures of the key targets were downloaded from RCSB PDB database (http://www.rcsb.org/). All the PDB files of the targets were cleaned by removing small molecules, ions, and native ligand in the crystal using PyMOL software. The resulting PDB files were prepared by ADT to adding hydrogen atoms, calculating and adding Gasteigar charges, merging non-polar hydrogen atoms, and setting rotatable torsion bonds to afford protein files for docking. The structure of **4i** was drawn and minimized energy using Chem3D 19.1. The torsional bonds of **4i** were automatically set by the AUTOTORS module in ADT. The docked sites of the protein were set in the place of the native ligand. The binding energy between **4i** and the targets was calculated using the AutoGrid program with a grid spacing of 0.375Å by the Lamarckian genetic algorithm as a searching method. The interaction between **4i** and the amino acid residues of the targets was found by LigPlot+ 2.1 and visualized by PyMOL software. The reliability of the docking method was confirmed when the RMSD value, which is obtained from the docking result between native ligand and the target, is less than 2Å. The accessible surface area (ASA) was calculated from Accessible Surface Area and Accessibility Calculation for Protein (ver. 1.2) (http://cib.cf.ocha.ac.jp/bitool/ASA/). The loss in ASA (ΔASA) was calculated as follows: ΔASA = ASA_Unbound protein_-ASA _Protein−ligand complex_. If the ΔASA loses more than 10Å^2^, the residue is involved in binding (Miao et al., 2019).

### Western Blot Analysis of PI3K/Akt Signaling Pathway Inhibitory Activity of 4i

Hela cells were seeded at a density of 3 × 10^6^ cells per dish and attached for 8h, and then treated with compounds in concentrations of 0, 5, 10, and 20μM for 24h. After that, cells were collected, washed with cold PBS, and lysed with lysis buffer (100mM Tris-HCl, pH 6.8, 4% SDS, 20% glycerol) on ice for 30min. Protein concentrations were detected using the bicinchoninc acid procedure (BCA) method (Beyotime Institute of Biotechnology, Haimen, China). Proteins were electrophoresed using sodium dodecyl sulfate/polyacrylamide gel electrophoresis (SDS-PAGE Bio-Rad, California, United States) and transferred electrophoretically to membranes. The membranes were blocked with 5% non-fat milk at room temperature for 1h and were incubated with primary antibodies overnight at 4°C. The next day, membranes were washed and incubated with the appropriate fluorescence-conjugated secondary antibodies at room temperature for 1h. Finally, membranes were washed and developed by the addition of enhanced chemiluminescence (ECL) substrate (Thermo Fisher Scientific, Rockford, United States). Proteins were visualized using the intelligent gel imaging system IBright FL1000 (Thermofisher, Rockford, United States). Primary antibodies were as follows: anti-phospho-4EBP1, anti-4EBP1, anti-mTOR, anti-phospho-mTOR, anti-Akt, anti-phospho-Akt, anti-PI3K(p85), anti-phospho-PI3K(p85), and anti-α-tubulin, which were purchased from Cell Signaling Technology (Cell Signaling Technology, Boston, MA, United States).

### Statistical Analyses

Statistical analysis was conducted on SPSS 22.0 (SPSS, Chicago, IL, United States), with a data-processing method of independent-sample *t* test. Comparisons between groups were statistically analyzed, and *p* < 0.05 was considered to be statistically significant.

## Results and Discussion

### Chemistry

**SCHEME 1:**
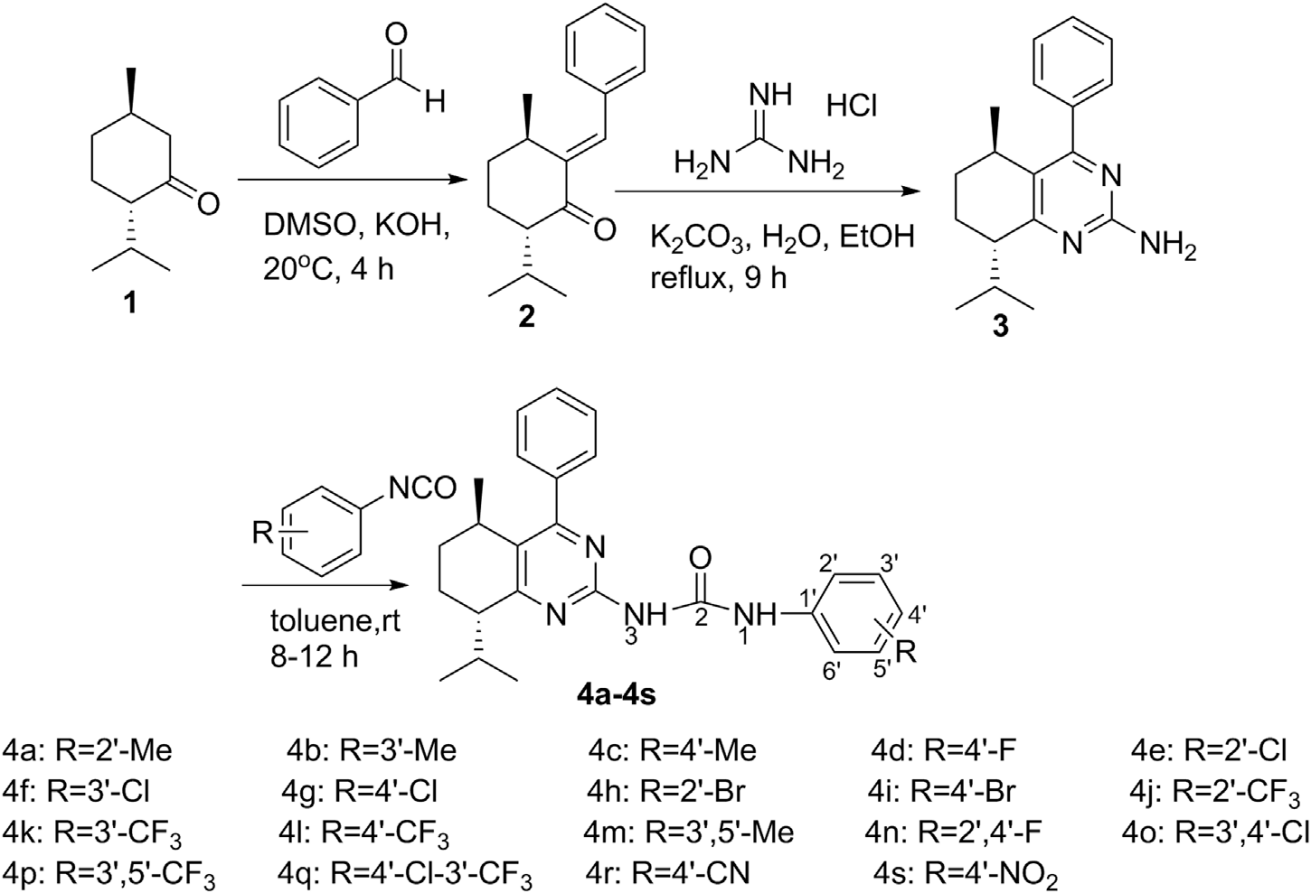
Synthesis of menthone-derived pyrimidine-urea compounds **4a-4s**.

The synthetic approaches adopted to afford the target compounds are outlined in Scheme 1, *L* *-*menthone-derived *α*, *β*-unsaturated ketone **2** was prepared by Claisen-Schmidt condensation reaction of *L* *-*menthone **1** with benzaldehyde. Then, *L* *-*menthone-derived pyrimidine **3** was prepared by cyclization of compound **2** with guanidine hydrochloride in the presence of K_2_CO_3_ in ethanol. Finally, a series of *L* *-*menthone-derived pyrimidine-urea compounds **4a-4s** were synthesized by nucleophilic addition reaction of compound **3** with substituted phenyl isocyanates.

The structures of all the target compounds were characterized by FT-IR, ^1^H NMR, ^13^C NMR, and HRMS. The spectra can be found in the Supplementary Material. In the FT-IR spectra of the target compounds, the absorption bands at about 3200cm^−1^ were attributed to the stretching vibrations of N-H. The characteristic absorption bands at about 1690cm^−1^ were assigned to the stretching vibrations of C=O. The characteristic absorption bands at about 1581–1620cm^−1^ were assigned to C=N in the pyrimidine moiety. In the ^1^H-NMR spectra, the protons of a benzene ring showed signals at 6.88–8.19ppm. The N–H protons exhibited signals at 11.08–12.30ppm and 7.51–8.13ppm, respectively. The protons of the menthone moiety displayed signals in the range of 0.77–3.12ppm. The ^13^C NMR spectra of the target compounds showed peaks for the carbon atom of C=O at about 155.00 ppm, carbon atoms of the benzene ring, and the unsaturated carbons at 102.92–158.02ppm. The other saturated carbons of menthone displayed signals in the region of 16.15–46.93ppm. Their molecular weights were confirmed by HRMS.

### Cytotoxicity Measurement

The *in vitro* antitumor activity of *L* *-*menthone-derived pyrimidine-urea compounds **4a-4s** against four human cancer cell lines (A549 human lung adenocarcinoma cell, Hela human cervical cancer cell line, MCF-7 human breast cancer cell line, andMGC-803 human gastric cancer cell line) was evaluated by MTT (methyl thiazolytetrazolium) assay and compared with the positive control 5-fluorouracil (5-FU) in each panel. The results are shown in Table 1.

**TABLE 1:** Anti-proliferative activities of the target compounds **4a-4s** against cancer cell lines.

Compounds	IC_50_ (μM)			
	A549	Hela	MCF-7	MGC-803
**4a** (R = *o*-CH_3_)	26.56 ± 0.14	24.57 ± 0.50	28.27 ± 1.44	21.98 ± 0.49
**4b** (R = *m*-CH_3_)	35.84 ± 0.76	28.29 ± 0.83	41.11 ± 4.09	20.19 ± 0.16
**4c** (R = *p*-CH_3_)	35.62 ± 1.63	13.54 ± 1.05	33.85 ± 1.73	6.37 ± 0.57
**4d** (R = *p*-F)	44.82 ± 0.79	18.74 ± 0.23	22.11 ± 1.46	7.86 ± 0.31
**4e** (R = *o*-Cl)	49.6 ± 0.42	70.17 ± 3.26	59.98 ± 2.72	21.92 ± 1.58
**l4f** (R = *m*-Cl)	38.03 ± 1.29	21.41 ± 0.42	54.42 ± 0.91	7.91 ± 0.72
**l4g** (R = *p*-Cl)	39.40 ± 3.09	7.95 ± 1.02	47.54 ± 3.19	3.21 ± 0.67
**4h** (R = *o*-Br)	>100	38.87 ± 0.69	>100	32.28 ± 3.18
**4i** (R = *p*-Br)	38.17 ± 0.93	6.04 ± 0.62	35.71 ± 0.28	3.99 ± 0.78
**4j** (R = *o*-CF_3_)	57.95 ± 1.39	55.53 ± 0.34	40.36 ± 0.87	20.61 ± 0.82
**4k** (R = *m*-CF_3_)	91.58 ± 3.95	13.66 ± 0.01	>100	>100
**4l** (R = *p*-CF_3_)	22.86 ± 0.52	38.04 ± 0.54	32.86 ± 1.90	17.74 ± 0.21
**4m** (R = *m,m*-CH_3_)	18.68 ± 1.53	33.80 ± 0.21	49.98 ± 5.88	15.88 ± 2.15
**4n** (R = *m, p*-F)	26.74 ± 0.15	10.91 ± 0.03	20.18 ± 2.38	6.08 ± 1.14
**4o** (R = *m, p*-Cl)	27.46 ± 0.55	22.29 ± 0.20	27.82 ± 0.76	18.12 ± 0.05
**4p** (R = *m,m*-CF_3_)	36.91 ± 0.95	61.46 ± 4.24	42.12 ± 1.72	16.02 ± 3.22
**4q** (R = *p-Cl-m*-CF_3_)	21.42 ± 2.35	42.13 ± 0.70	66.15 ± 0.50	9.36 ± 0.33
**4r** (R = *p*-CN)	21.69 ± 0.95	40.32 ± 2.78	24.86 ± 1.97	14.44 ± 0.04
**4s** (R = *p*-NO_2_)	95.63 ± 0.04	26.37 ± 0.10	19.09 ± 0.49	14.2 ± 1.07
5-FU	31.41 ± 0.41	>50	>50	14.55 ± 1.24

IC_50_ values are expressed as the mean ± SD (standard deviation) from three independent experiments.

It was found that 7, 16, 14, and 9 compounds exhibited better antitumor activity than the positive control, 5-FU, against A549, Hela, MCF-7, and MGC-803, respectively, while the target compounds showed more effective inhibitory activity against MGC-803 than other tested cell lines, which were inhibited by the target compounds in a similar level. Compounds **4m, 4i**, **4s**, and **4g** showed the best IC_50_ values of 18.68 ± 1.53µM, 6.04 ± 0.62µM, 19.09 ± 0.49µM, and 3.21 ± 0.67µM against A549, Hela, MCF-7, and MGC-803, respectively. Compounds **4i** and **4g** showed good dual antitumor activity against Hela and MGC-803.

In addition, the compounds with para substituent showed good activity in the greater probability, especially the halogen substituted compounds. It is indicated that the lone electron pair on the para substituent might act as an important role in tumor inhibition. For the further action mechanism study, compound **4i** was employed as a representative.

### Cell Cycle Analysis

**FIGURE 2:**
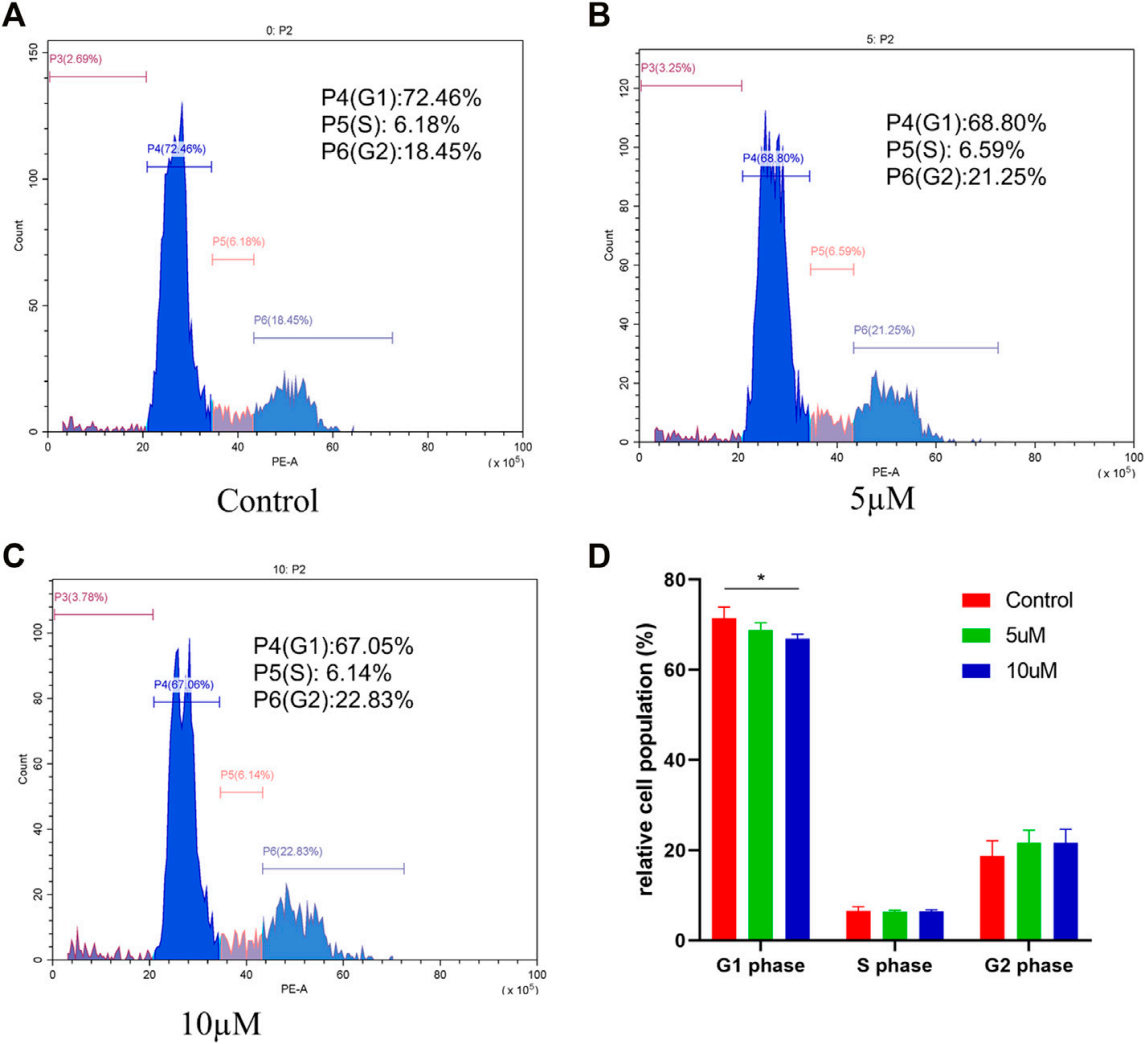
Effect of compound **4i** (0°μM **(A)**, 5°μM **(B)**, and 10°μM **(C)**) on the cell cycle of human cervical cancer Hela cell. The graph **(D)** presents cells in the G0/G1, S, and G2/M phases in the Hela cell line using PI staining after exposure to compound **4i** at (0°μM, 5°μM, and 10°μM) for 48 h. Data are expressed as the mean and SD from three independent experiments; *p < 0.05.

To study the possible role of compound **4i** in the tumor growth inhibition, different concentrations of compound **4i** were treated with Hela cells for 48h. The cell cycle distribution was investigated by flow cytometric analysis following the staining of DNA with propidium iodide (PI). It was observed that the G0/G1 phase cells were gradually decreased from 72.46% (0μM) to 67.05% (10μM, *p* = 0.043), and the S phase cells changed unobviously, while the G2/M phase cells gradually increased from 18.45% (0μM) to 22.83% (10μM) (Figure 2). Thus, these results suggested that the target compound **4i** might arrest the Hela cells in the G2/M phase.

### Compound 4i Induces Apoptosis in Hela Cells

**FIGURE 3:**
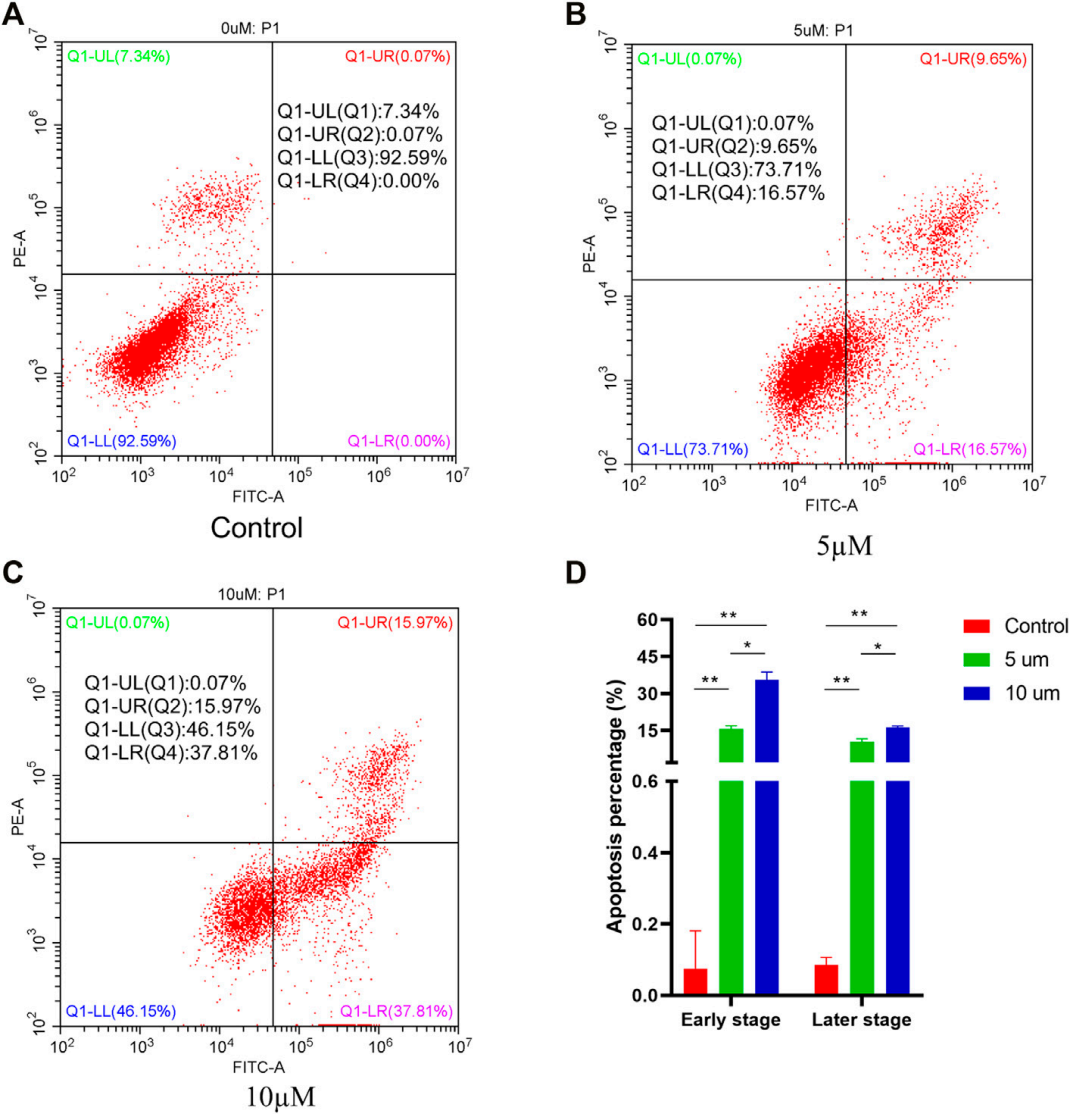
Apoptosis ratio detection of compound **4i** (0°μM **(A)**, 5°μM **(B)**, and 10 **(C)** μM) by flow cytometry. The graph **(D)** presents cell apoptosis in an early stage and a later stage in the Hela cell line using PI staining after exposure to compound **4i** at (0°μM, 5°μM, and 10°μM) for 48 h. Data are expressed as the mean and SD from three independents experiments; *P < 0.05, **P < 0.01.

To investigate whether compound **4i** could induce cell apoptosis, Hela cells were treated with compound **4i** in different concentrations for 48h. The Annexin V-FITC/PI dual staining assay was carried out by flow cytometry. The results are illustrated in Figure 3. The percentage of all apoptotic cells (early and late apoptotic cells, lower right quadrant and upper right quadrant, AV+/PI, respectively) significantly increased from 0.07% (0μM, early, 0.00%; later, 0.07%) to 26.22% (5μM, early, 16.57%, *p* = 0.003; later 9.65%, *p* = 0.007) and 53.78% (10μM, early, 37.81%, *p* = 0.004; later 15.97%, *p* = 0.001), while the increase in the 10µM treatment group was much greater than that in the 5µM treatment group (early, *p* = 0.015; later, *p* = 0.024). These results clearly confirmed that compound **4i** effectively induced apoptosis in Hela cells in a dose-dependent manner.

### Preliminary Exploration of the Mechanism of Compound 4i Against Hela Cells Based on Network Pharmacology and Molecular Docking Technology

#### Potential Targets of 4i Against Hela Cell Line

A total of 293 targets of compound **4i** (Supplementary Table S1) were obtained by PharmMapper Server. A total of 834 Hela cell line associated targets (Supplementary Table S2) were collected by OMIM database and Genecards database after eliminating duplicates. Finally, 59 potential targets were obtained by overlapping the two sets (Supplementary Table S3).

#### Construction and Analysis of Protein-Protein Interaction Network

**FIGURE 4:**
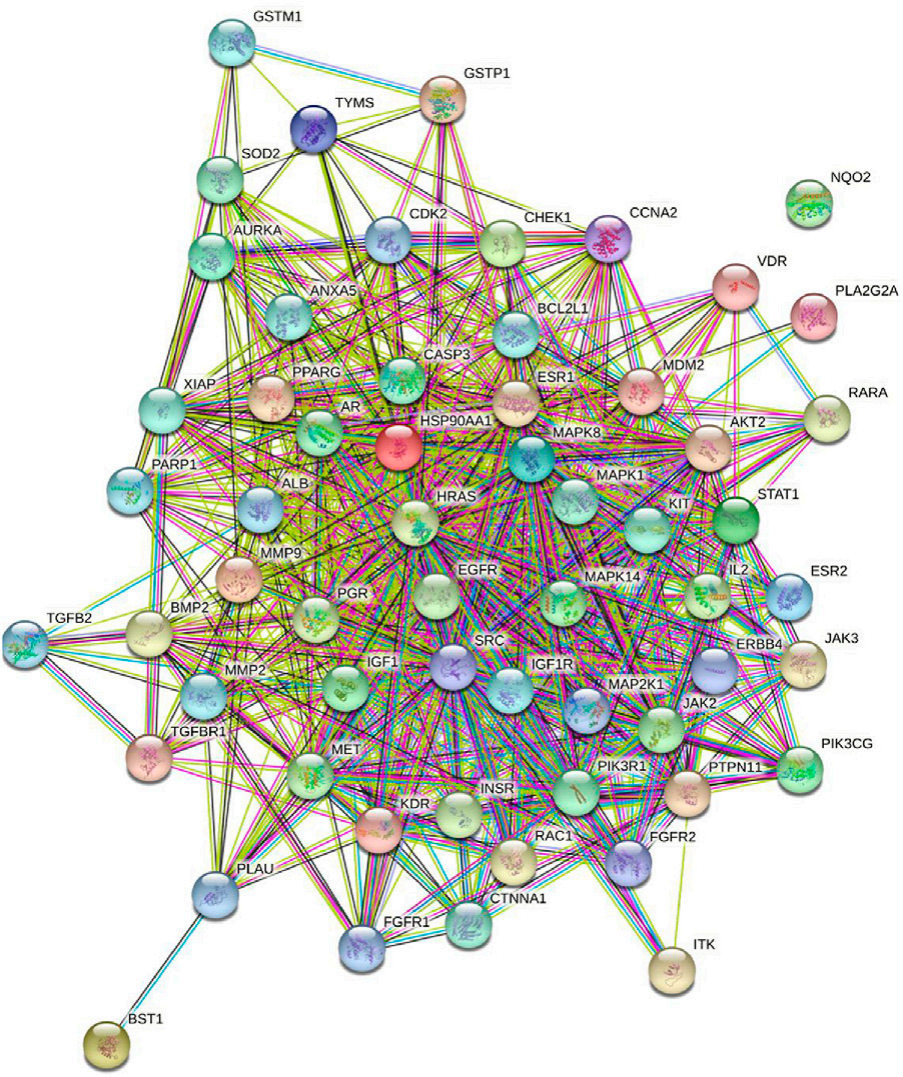
Network of potential targets of **4i** in the Hela cell line analyzed by STRING.

A total of 59 targets was imported into the STRING database to obtain the data of PPI and the visualized PPI network. As shown in Figure 4, the nodes in the network represent the potential targets, and the edges represent the interaction between the targets. The network was comprised of 59 nodes and 708 edges, and the average node degree was 24.4. The targets with the degree greater than 1.9 times median, EGFR, HRAS, and MAPK1, were selected as the core targets (Table 2).

**TABLE 2:** The predicted core targets of **4i** in the Hela cell line.

No.	Gene symbol	Protein name	Degree in PPI network
1	EGFR	Epidermal growth factor receptor (EGFR)	50
2	HRAS	HRas proto-oncogene, gtpase(HRAS)	47
3	MAPK1	Mitogen-activated protein kinase 1 (MAPK1)	47

#### Results of GO and KEGG Pathway Enrichment Analysis

**FIGURE 5:**
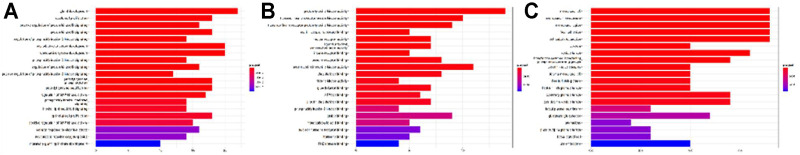
GO enrichment of **4i** targets. **(A)** Biological process. **(B)** Molecular function. **(C)** Cellular components.

**FIGURE 6:**
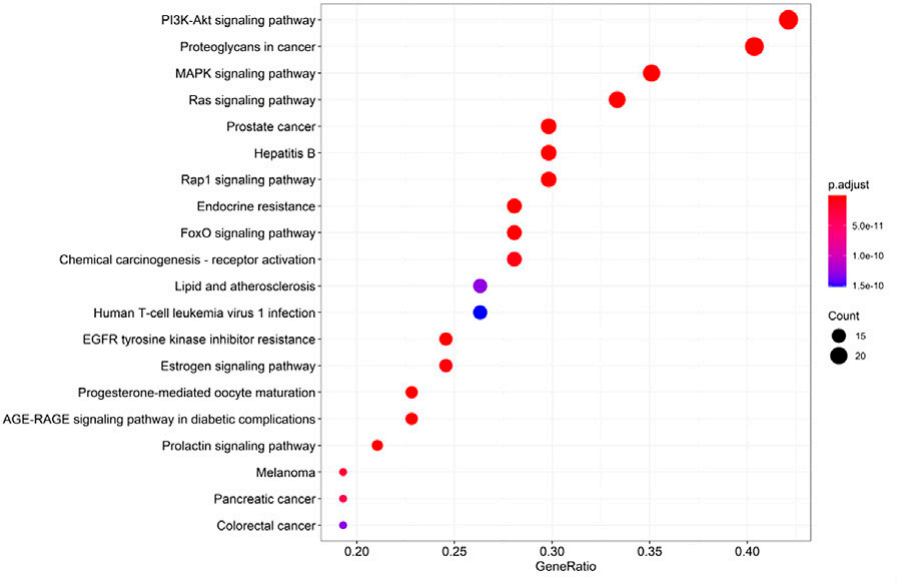
KEGG pathway enrichment of **4i** targets.

GO and KEGG pathway enrichment analysis were performed using the 59 potential targets in R packages, and the GO terms and KEGG pathways with *p*-value < 0.05 were significantly enriched (Supplementary Tables S4 and S5). The top 20 GO terms of biological process, molecular function, and cellular components were shown in Figure 5. The KEGG pathway enrichment analysis indicated that 54 of the 59 targets (91.52%) were enriched in 141 pathways. The top 20 pathways were shown in Figure 6. There are 6 cancer pathways, 5 signal transduction pathways, 3 endocrine system pathways, 2 antineoplastic drug resistance pathways, 2 viral infectious pathways, 1 endocrine and metabolic disease pathway, and 1 cardiovascular disease pathway (Supplementary Table S6). This result indicated that compound **4i** against cancer may through regulating these pathways.

Furthermore, there are 12 proteins involved in the top 20 pathways more than 15 times, including MAP2K1(19), KDR (19), IGF1R (19), HRAS (19), IGF1 (19), FGFR1 (19), Akt2 (19), ESR1 (19), ERBB4(19), EGFR (19), CASP3(19), and MAPK1(16), suggesting that these targets may be the key proteins in the enriched pathways.

### Molecular Docking Analysis

Proteins, EGFR, HRAS, and MAPK1, are the overlapping targets of the core targets in PPI network analysis and the key proteins in the top 20 pathways. Activated EGFR, HRAS, and MAPK1 can stimulate the activation of PI3K/Akt signaling pathway. Akt2 is a member protein in the pathway of PI3K-Akt signaling pathway which is the first pathway in the KEGG pathway enrichment. Thus, these four proteins, EGFR, HRAS, MAPK1, and Akt2, were studied in molecular docking. All the RMSD value in the docking resulted in less than 2Å, which is considered a good prediction for computed ligand-protein conformation and indicated the docking method is believable (García-Godoy et al., 2016).

Many reports show that the overexpression or the mutational activation of these four targets was found in cancer cells and provides strong growth and survival signals to cancer cells (Vajdos et al., 2001; Yun et al., 2007; Zhu et al., 2011; Rampias et al., 2014; Sharma et al., 2019; Jiang et al., 2021). For regulating the expression of the targets, some small molecules which could bind with the specific region at the target proteins were found as inhibitors for cancer therapy (Roskoski, 2018; Alzahrani, 2019). Thus, in this work, the ATP-binding sites, which contain the native ligand as inhibitor of the targets, were chosen as docking sites.

In the results, the predicted minimum binding energies of compound **4i** with the target proteins are less than or equal to −7.48kcal/mol. Particularly, the minimum binding energy of compound **4i** with MAPK1 is lower than that of the native ligand. These low binding energies indicated that compound **4i** may form stable binding with the docking sites and might act as a well inhibitor against EGFR, HRAS, MAPK1, and Akt2, respectively (Table 3).

**TABLE 3:** The basic information of the protein and the minimum binding energy predicted by docking.

Name of proteins	PDB ID	RMSD value (in Å)^a^	Minimum binding energy (kcal/mol)	
			Native ligand^b^	4i
EGFR	2j6m	0.94	−9.92	−7.48
HRAS	1wq1	0.37	−12.57	−8.91
MAPK1	2oji	0.81	−8.32	−8.8
AKT2	2uw9	0.43	−10.39	−9.41

a Note: The RMSD value is obtained from the docking result between the native ligand and the target.

b Note: The native ligand in the PDB, files. ^#^The ID and the formula of the native ligand in the corresponding PDB files.

**FIGURE 7:**
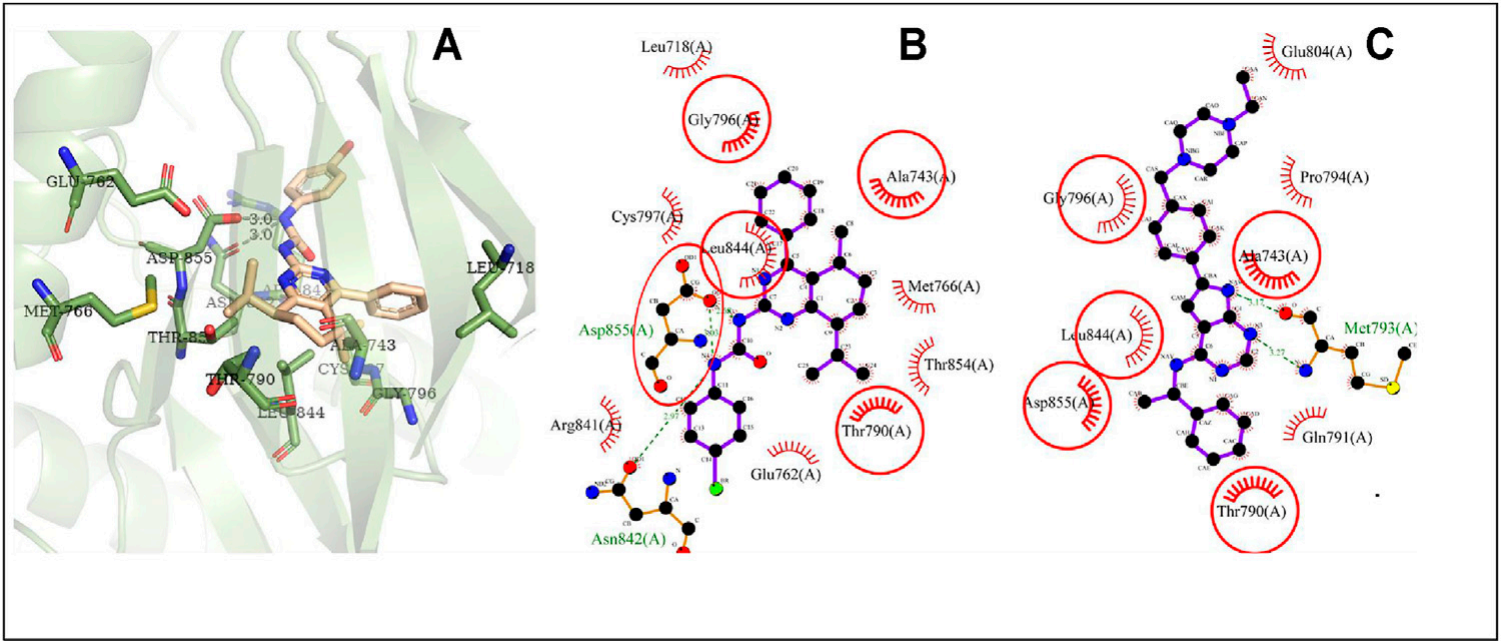
The conformation of **4i** docked in EGFR. Note **(A)** 3D docking conformation; **(B)** 2D docking conformation; **(C)** 2D conformation of native ligand and residues, the same as below.

The possible interaction mode between the residues of EGFR and compound **4i** were shown in Figure 7. Five of 12 residues (41.67%) are overlapping with the interacting residues of the native ligand, including an overlapping residue, Asp855, forming a hydrogen bond with urea moiety at N atom, which is also forming a hydrogen bond with binding site residue Asp855. Arg841 with the maximum ΔASA (48.158Å^2^) is seen as the key interacting residue (Supplementary Table S7).

**FIGURE 8:**
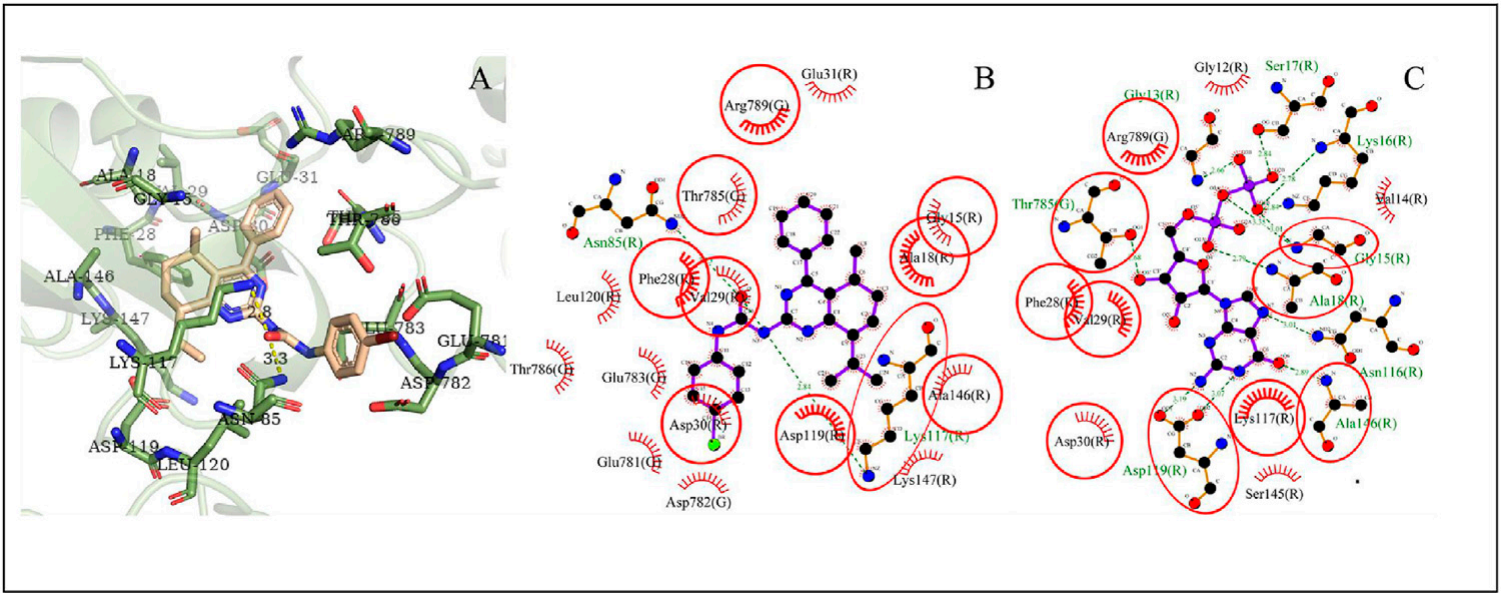
The conformation of **4i** docked in HRAS.

The possible interaction mode between the residues of HRAS and compound **4i** were shown in Figure 8. Ten of 18 residues (55.56%) are overlapping with the interacting residues of the native ligand, including an overlapping residue, Lys117, forming a hydrogen bond with urea moiety at O atom, which is also forming a hydrogen bond with binding site residue Asn85. Lys117 with the maximum ΔASA (48.206Å^2^) is seen as the key interacting residue.

**FIGURE 9:**
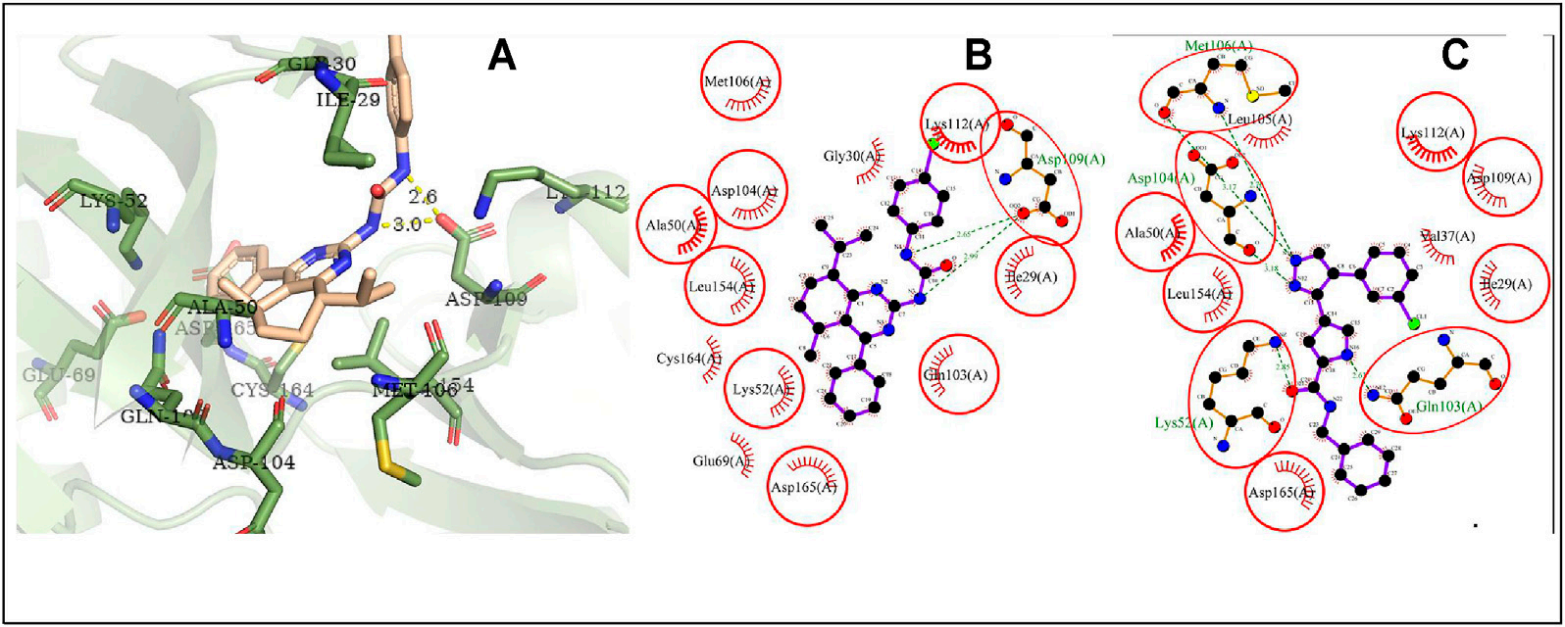
The conformation of **4i** docked in MAPK1.

The possible interaction mode between the residues of MAPK1 and compound **4i** were shown in Figure 9. Ten of 13 residues (76.92%) are overlapping with the interacting residues of the native ligand, including an overlapping residue, Asp109, forming two hydrogen bonds with the 2N atoms of urea moiety. Ile29 with the maximum ΔASA (46.449Å^2^) is seen as the key interacting residue.

**FIGURE 10:**
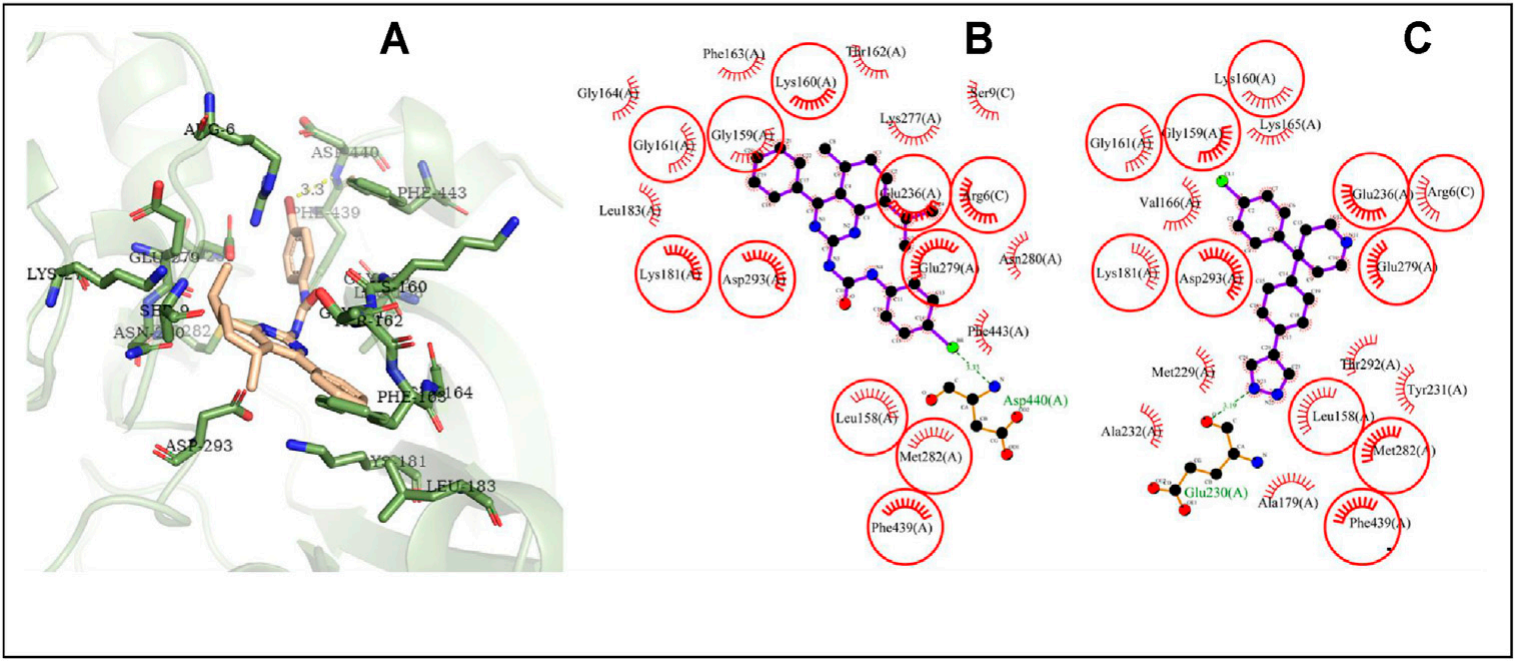
The conformation of **4i** docked in Akt2.

The possible interaction mode between the residues of Akt2 and compound **4i** were shown in Figure 10. Eleven of 20 residues (55.00%) are overlapping with the interacting residues of the native ligand. The binding site residue Asp440 formed a hydrogen bond with the Br atom. Asp293 with the maximum ΔASA (48.293Å^2^) is seen as the key interacting residue.

The interaction mode study showed that the higher overlapping ratio of compound **4i** to the native ligand interacting residues and the higher average ΔASA of compound **4i** might relate to low minimum binding energy. A hydrogen bond is also a positive factor for ligand-protein accommodation. In the docking results, the hydrogen bonds’ interaction almost associates to urea moiety, indicating that introduction of urea moiety is favorable to form new compounds with good protein-binding ability. In addition, the Br atom of compound **4i** could form a hydrogen bond with the Akt2 binding site residue that might be a factor of the para halogen substituted compounds showing good antitumor activity.

### PI3K/Akt/mTOR Signaling Pathway Inhibitory Activity of 4i

**FIGURE 11:**
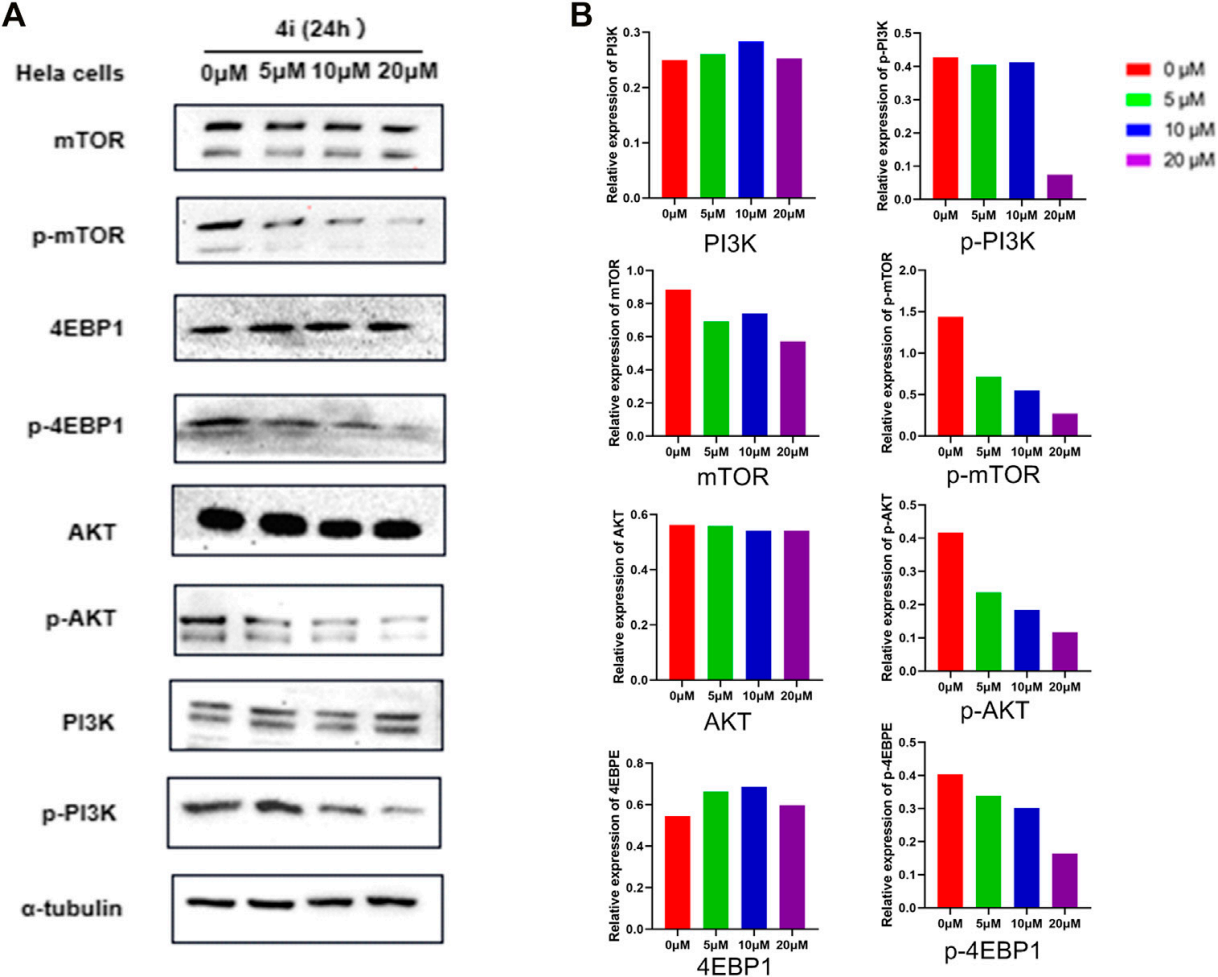
Inhibitory effects of compound **4i** in the Hela cell line according to Western blot analysis. **(A)** The expression levels of the PI3K,p-PI3K, mTOP, p-mTOR, AKT, p-AKT, 4EBPE, and p-4EBPE in the HeLa cell which were treated with (0°μM, 5°μM, 10°μM, and 20°μM of **4i**) for 24 h,were examined by Western Blot **(B)**.

The inhibitory activity of compound **4i** against PI3K/Akt/mTOR signaling pathway, the first pathway in the KEGG pathway enrichment analysis and its downstream protein, was investigated. The proteins’ expressions of PI3K(p85), p-PI3K(p85), AKT, *p*-AKT, mTOR, *p*-mTOR, 4EBP1, p-4EBP1, and *α*-tubulin in Hela cells were detected by Western blot method. The result is shown in Figure 11. In the Hela cells treated by compound **4i**, the total PI3K(p85), AKT, mTOR, 4EBP1, and α-tubulin were almost not affected by the treatments, while the expression of the phosphorylated proteins, p-PI3K(p85), *p*-AKT, *p*-mTOR, and p-4EBP1 suffered significant decrease.

In PI3K/Akt/mTOR signaling pathway, the phosphorylated PI3K (p-PI3K), some of which were constructed by a catalytic subunit (p110) and a regulatory subunit (p85), could phosphorylate PIP2 to convert it into PIP3. PIP3 could activate Akt through phosphorylation, and further activate the mTOR as phosphorylated mTOR. The activated mTOR could deactivate its downstream effectors 4EBP1, which could bind with eIF4E to inhibit the cells’ proliferation, growth, and protein synthesis, through phosphorylation (Condon and Sabatini, 2019; Rehan and Bajouh, 2019; Sun et al., 2019). Thus, the decreased expression of the phosphorylated PI3K, Akt, mTOR, and downstream effectors 4EBP1 indicated that compound **4i** could inhibit PI3K/Akt/mTOR signaling pathways significantly.

## Conclusion

In summary, a series of novel menthone derivatives bearing pyrimidine and urea moieties were synthesized and identified by FTIR, NMR, and HRMS. All the synthesized compounds were evaluated for their cytotoxic effects against A549, Hela, MCF-7, and MGC-803 cell lines by standard MTT assay. The results revealed that most of the target compounds exhibited better antitumor activity than the positive control, 5-FU. Compounds **4m, 4i**, **4s**, and **4g** showed the best IC_50_ values against A549, Hela, MCF-7, and MGC-803, respectively. In particular, compounds **4i** and **4g** showed good dual antitumor activity against Hela and MGC-803. The action mechanistic studies, using compound **4i** as a representative, revealed that compound **4i** could markedly induce Hela cell apoptosis in a dose-dependent manner and arrested them in the G2/M phase. Network pharmacology prediction showed that compound **4i** might against Hela cells through regulating the expression and activation of the core targets, EGFR, HRAS, MAPK1, and Akt2. In KEGG pathway enrichment, PI3K/Akt signaling pathway was evaluated as the first pathway for compound **4i** against Hela cells. The Western blot assay confirmed that compound **4i** could inhibit PI3K/Akt signaling pathway, including the downstream protein mTOR and downstream effectors 4EBP1, significantly. Molecular docking shows that compound **4i** was able to interact efficiently with the ATP-binding site of EGFR, HRAS, MAPK1, and Akt2. The urea moiety and para substituent halogen acted as important roles for ligand-protein stabling. These results indicated that the synthetic strategy in this study is feasible. The compound **4i** might serve as a leading compound for potent antitumor agents such as PI3K/Akt/mTOR signaling pathway inhibitor.

## Data Availability

The original contributions presented in the study are included in the article/Supplementary Material, further inquiries can be directed to the corresponding authors.
