# Supplementary material for: Synthesis and Antitumor Evaluation of Menthone-Derived Pyrimidine-Urea Compounds as Potential PI3K/Akt/mTOR Signaling Pathway Inhibitor
Source: Front Chem. 2022 Feb 3;9:815531. doi: 10.3389/fchem.2021.815531 (PMC8852737; doi:10.3389/fchem.2021.815531)
Supplement: Supplementary file 1 [file DataSheet1.docx]

Supplementary Material

**NMR-Spectra**


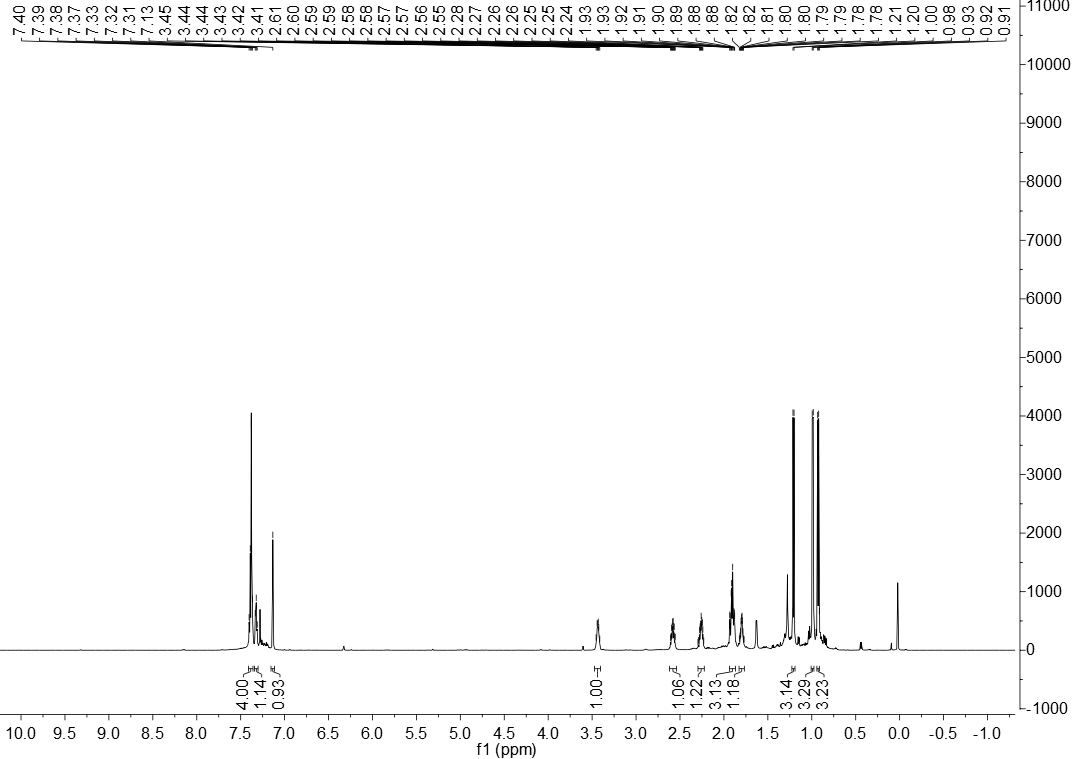


**FigureS1.** ^1^H NMR (600 MHz, CDCl_3_) of compound **2**


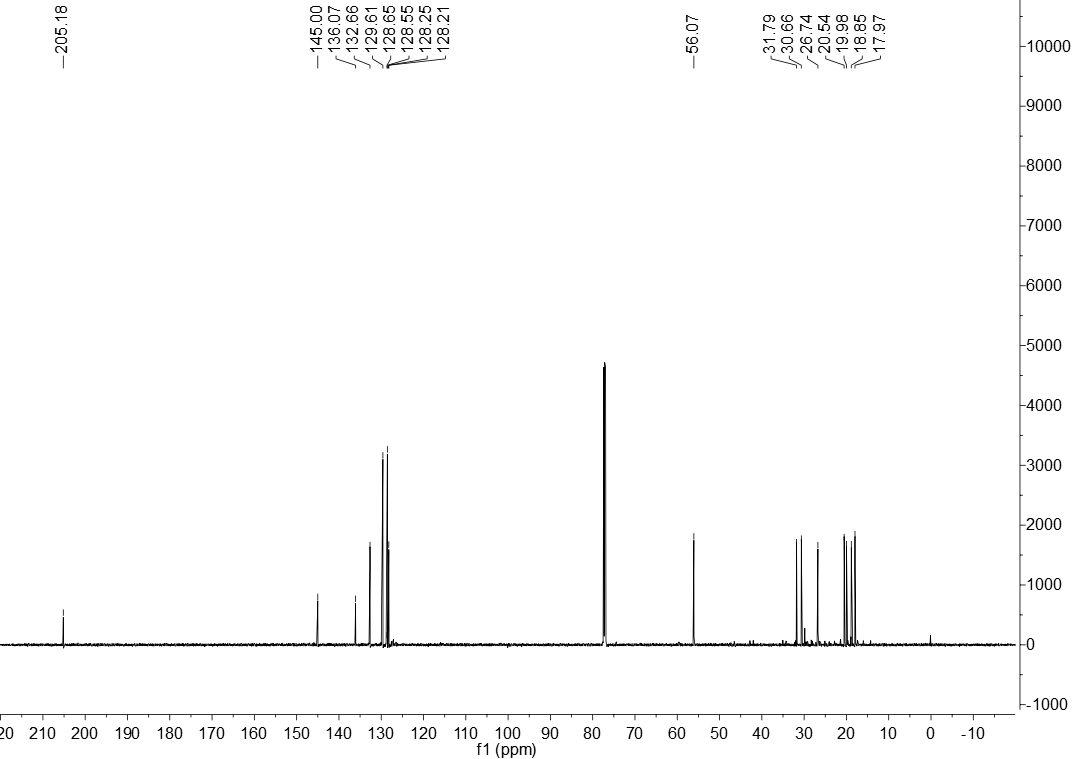


**FigureS2.** ^13^C NMR (150 MHz, CDCl_3_) of compound **2**


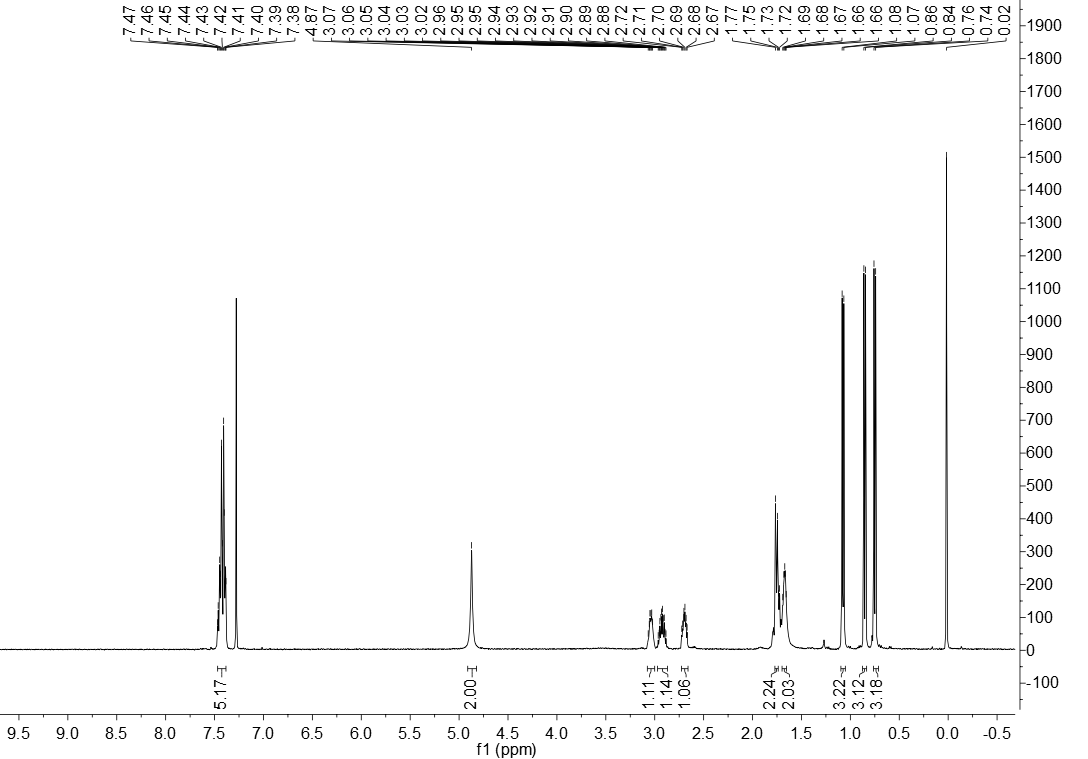


**FigureS3.** ^1^H NMR (500 MHz, CDCl_3_) of compound **3**


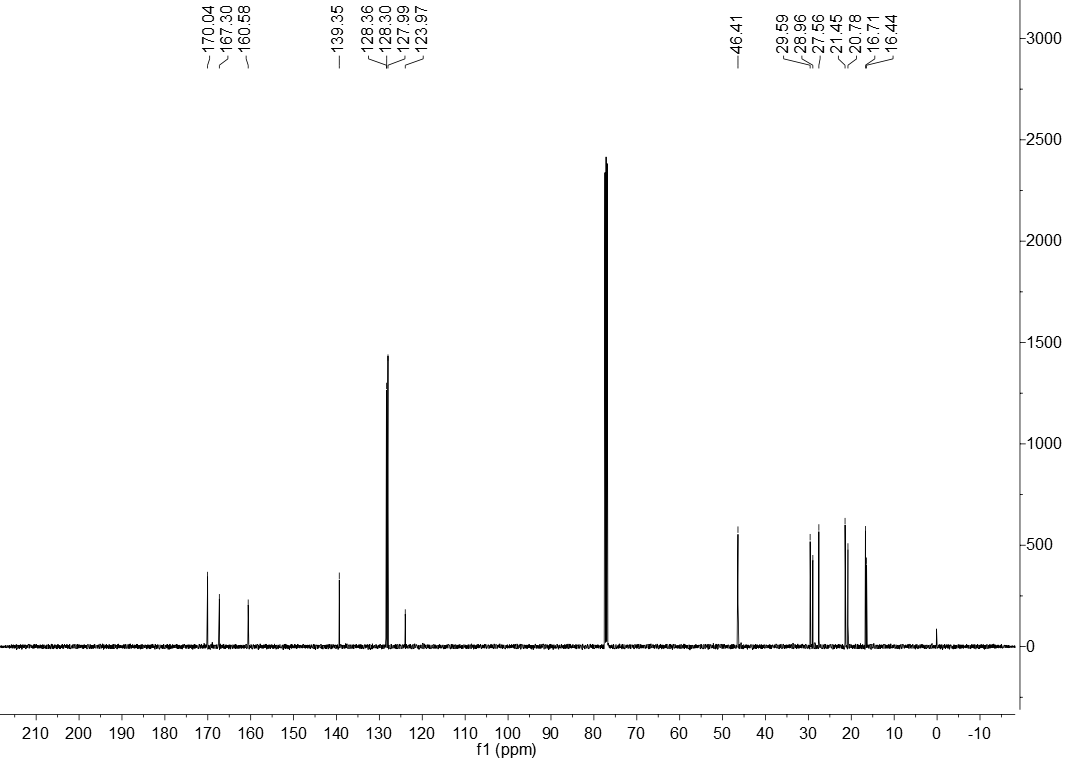


**FigureS4.** ^13^C NMR (126 MHz, CDCl_3_) of compound **3**


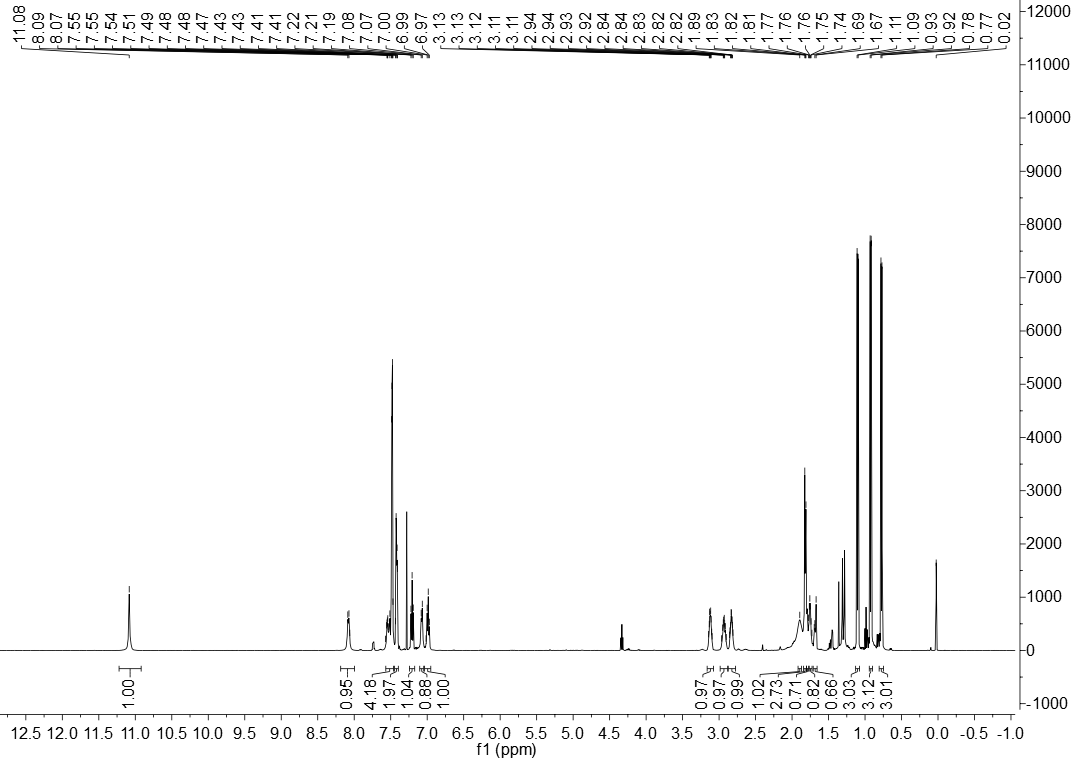


**FigureS5.** ^1^H NMR (500 MHz, CDCl_3_) of compound **4a**


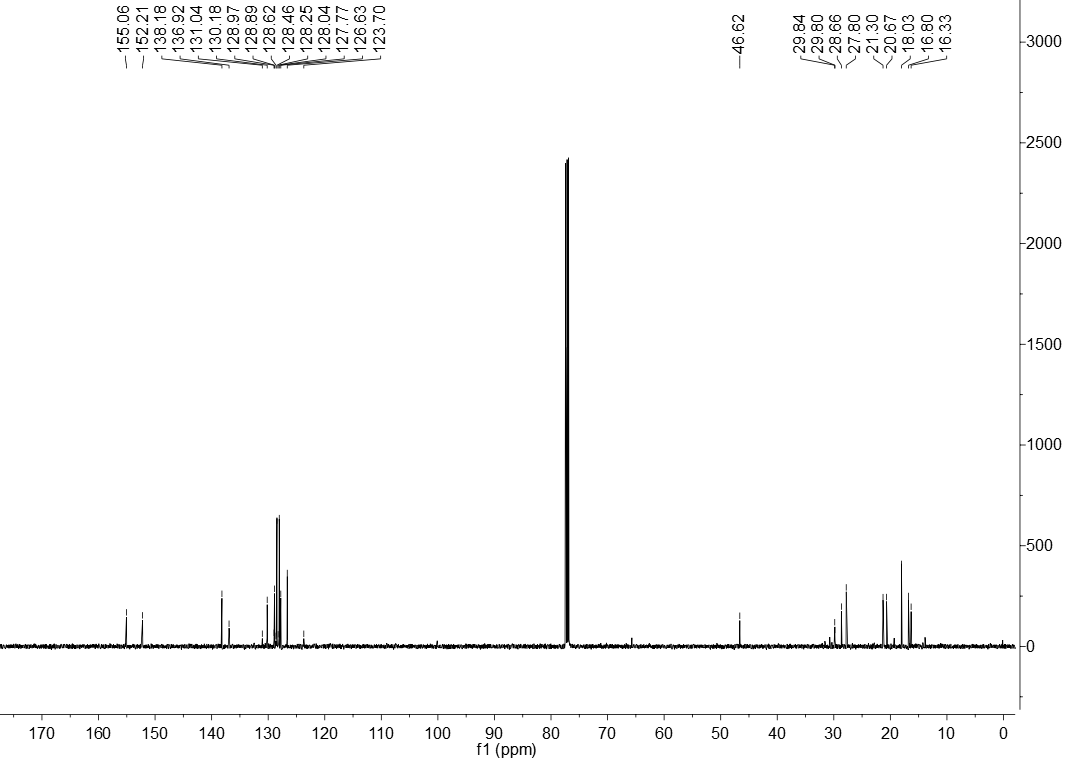


**FigureS6.** ^13^C NMR (126 MHz, CDCl_3_) of compound **4a**


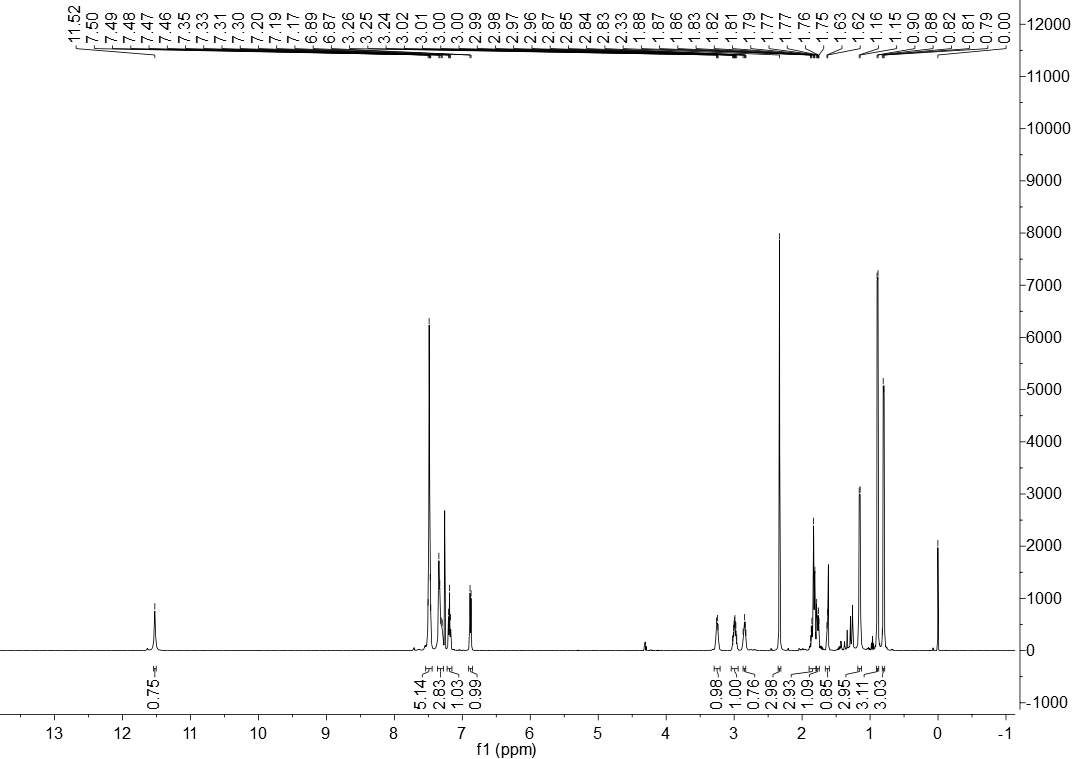


**FigureS7.** ^1^H NMR (500 MHz, CDCl_3_) of compound **4b**


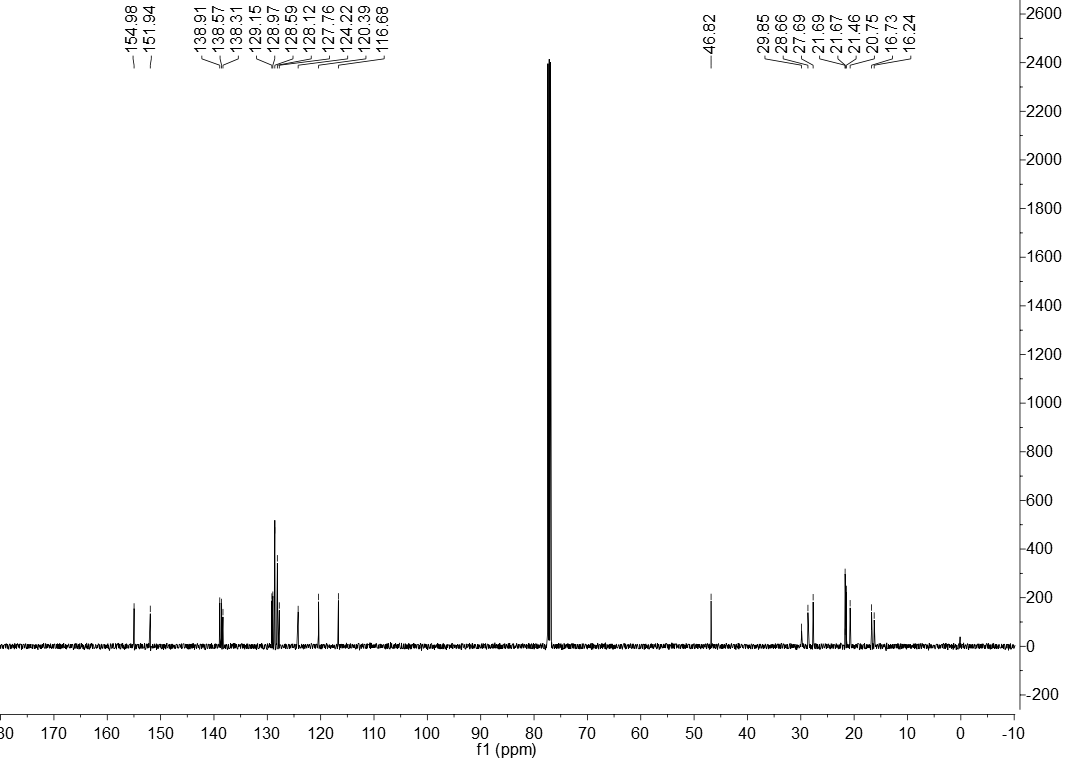


**FigureS8.** ^13^C NMR (126 MHz, CDCl_3_) of compound **4b**


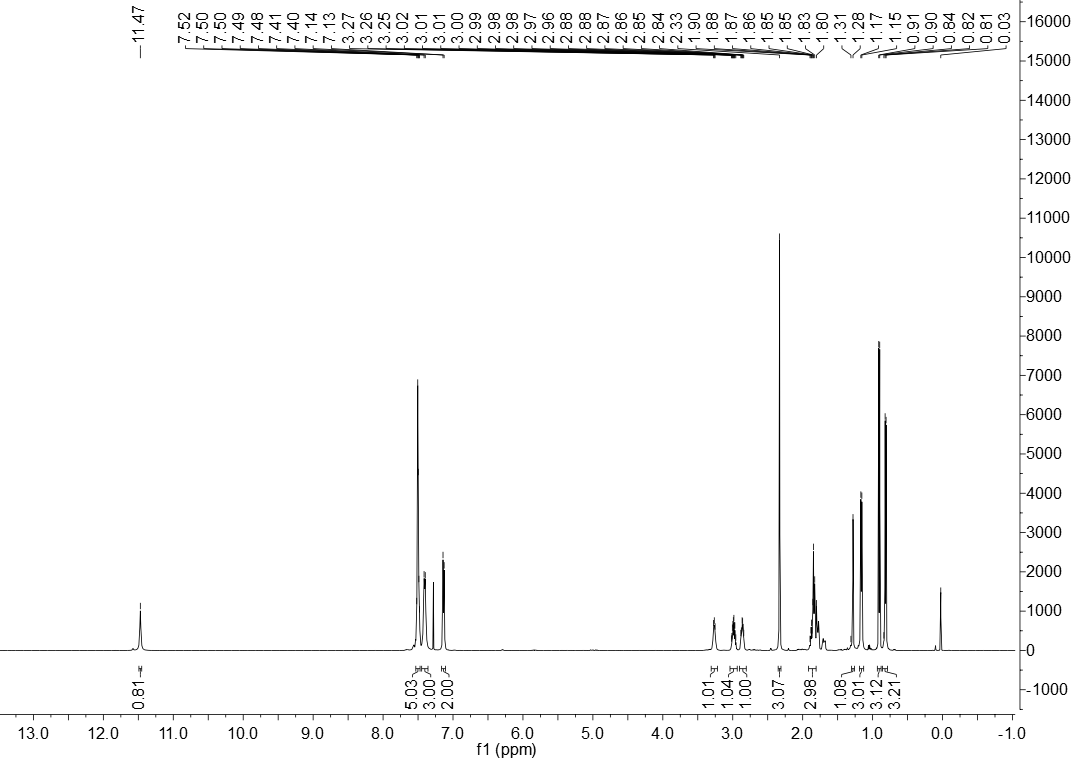


**FigureS9.** ^1^H NMR (500 MHz, CDCl_3_) of compound **4c**


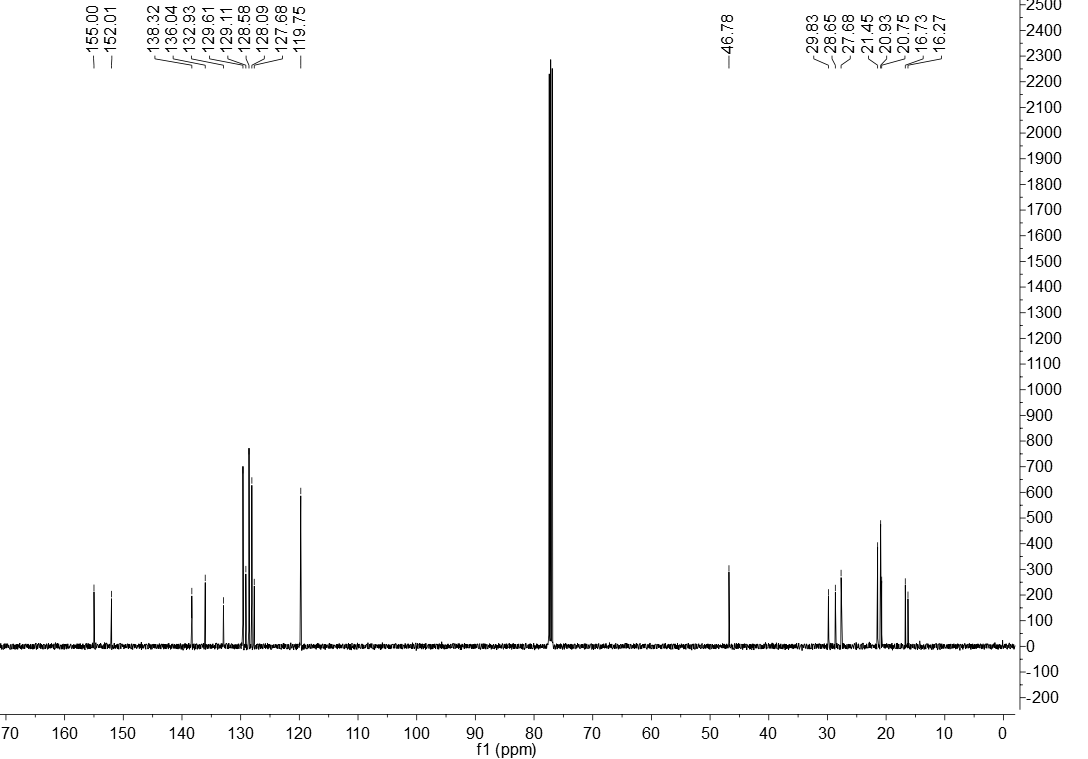


**FigureS10.** ^13^C NMR (126 MHz, CDCl_3_) of compound **4c**


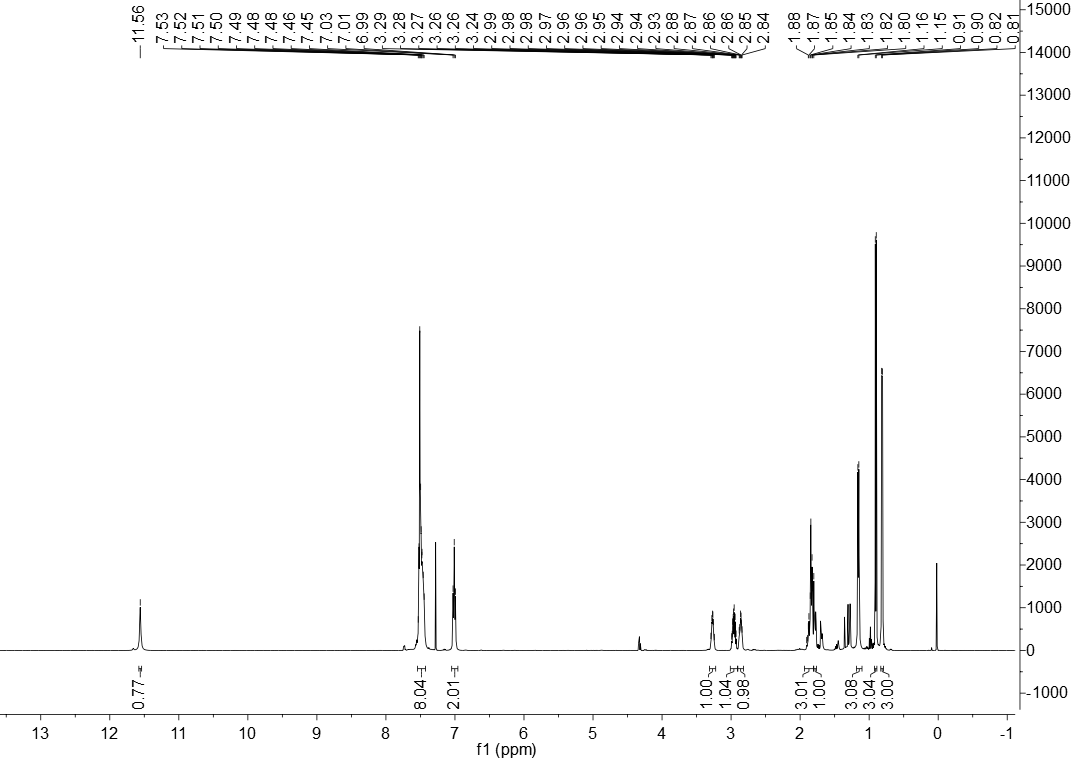


**FigureS11.** ^1^H NMR (500 MHz, CDCl_3_) of compound **4d**


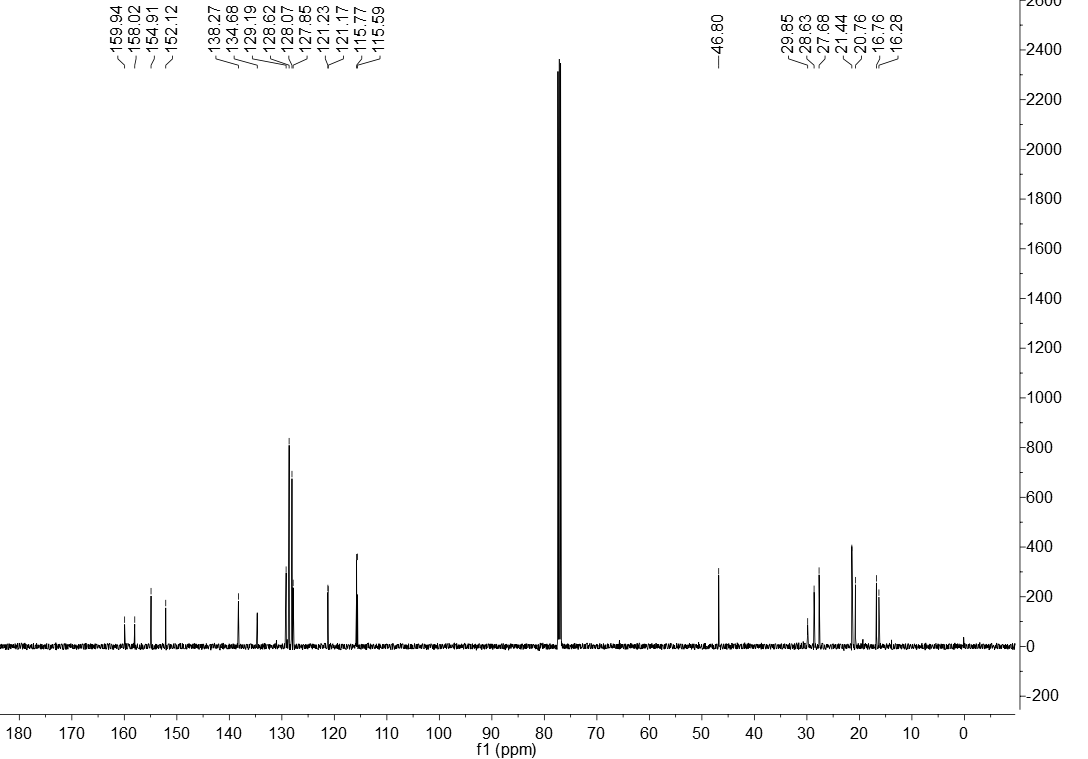


**FigureS12.** ^13^C NMR (126 MHz, CDCl_3_) of compound **4d**


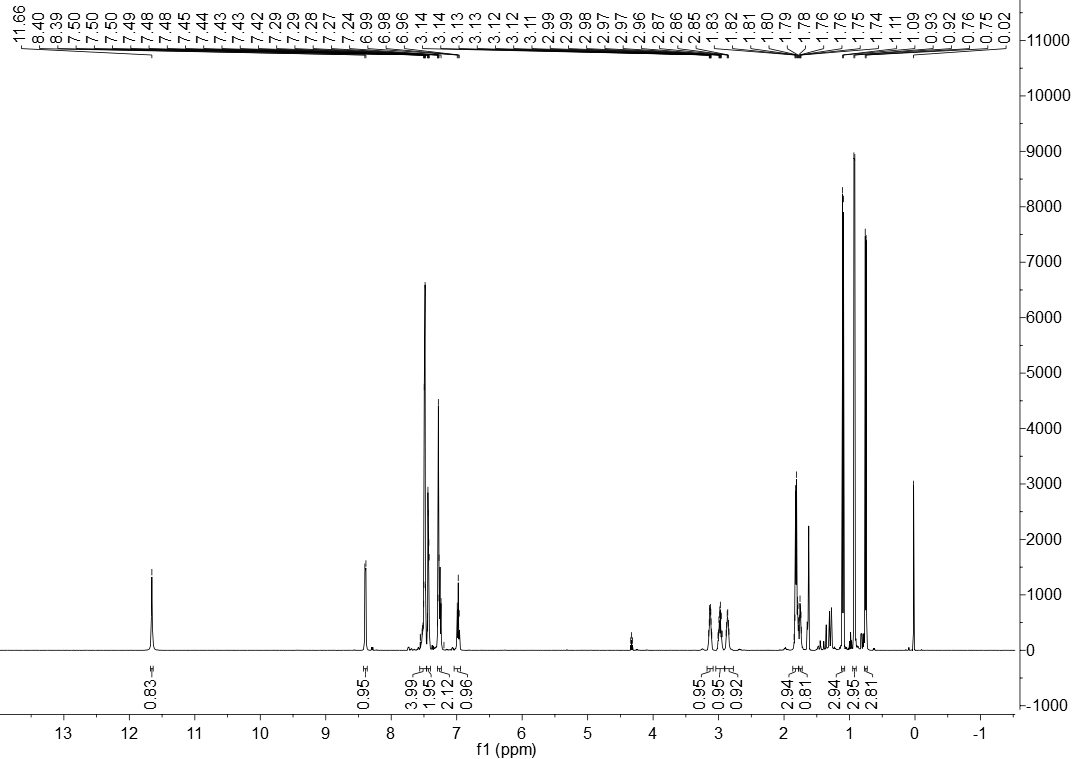


**FigureS13.** ^1^H NMR (500 MHz, CDCl_3_) of compound **4e**


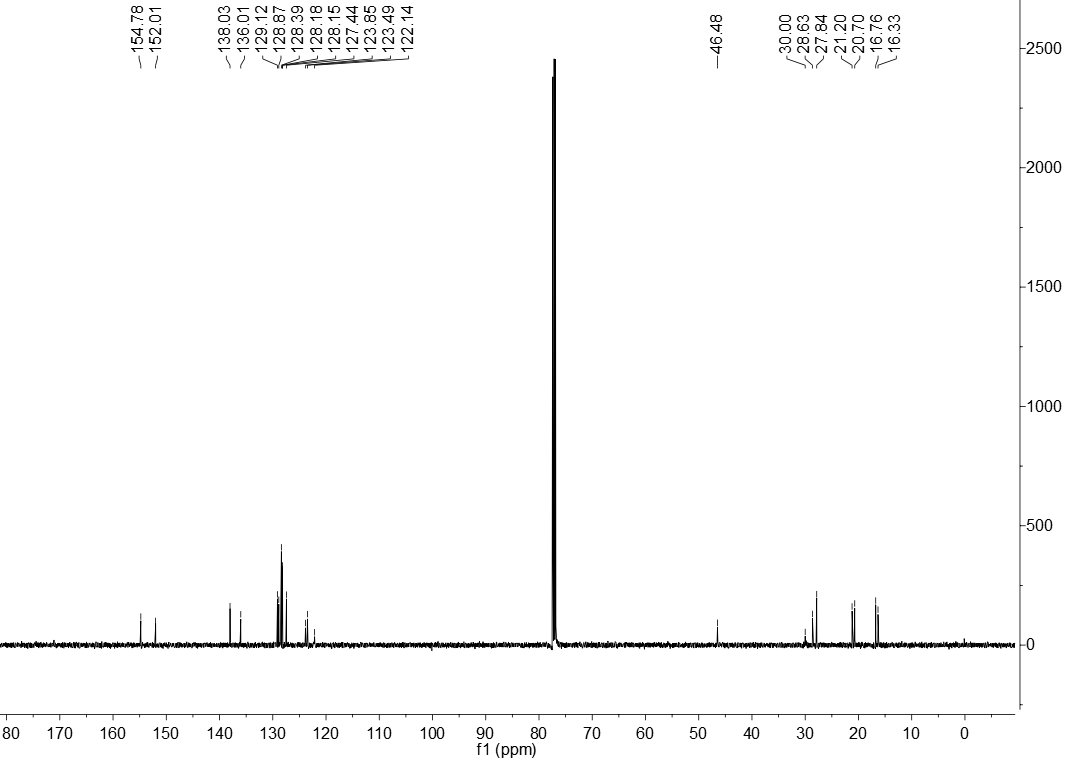


**FigureS14.** ^13^C NMR (126 MHz, CDCl_3_) of compound **4e**


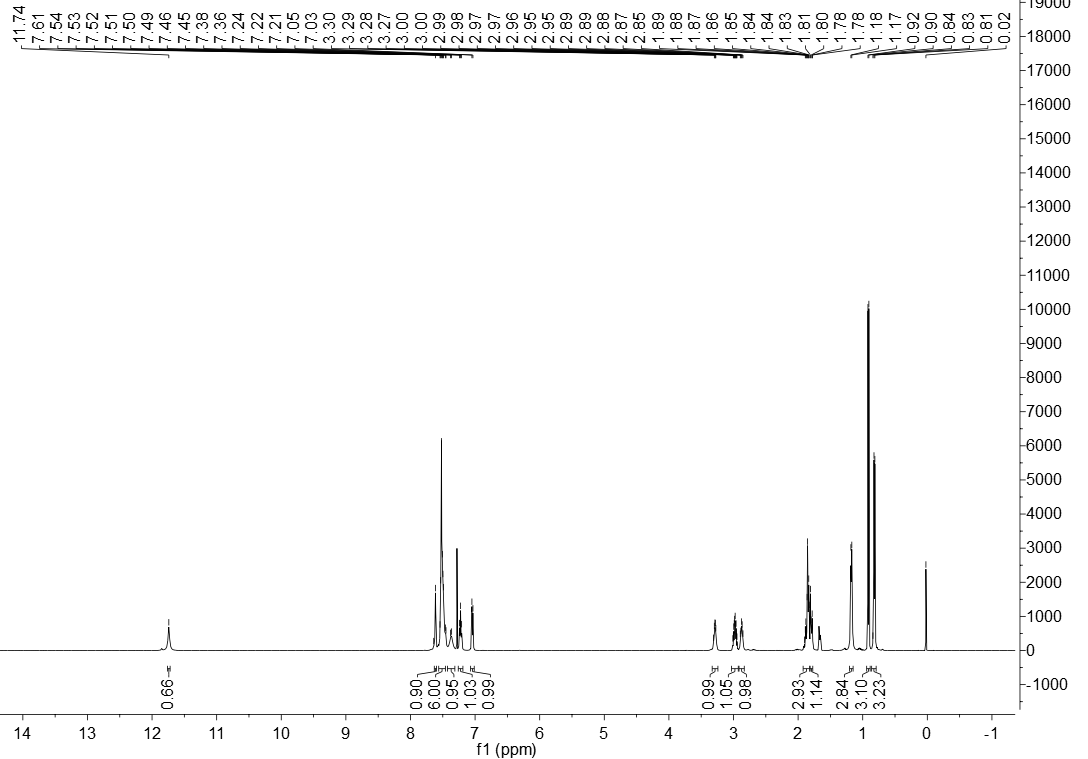


**FigureS15.** ^1^H NMR (500 MHz, CDCl_3_) of compound **4f**


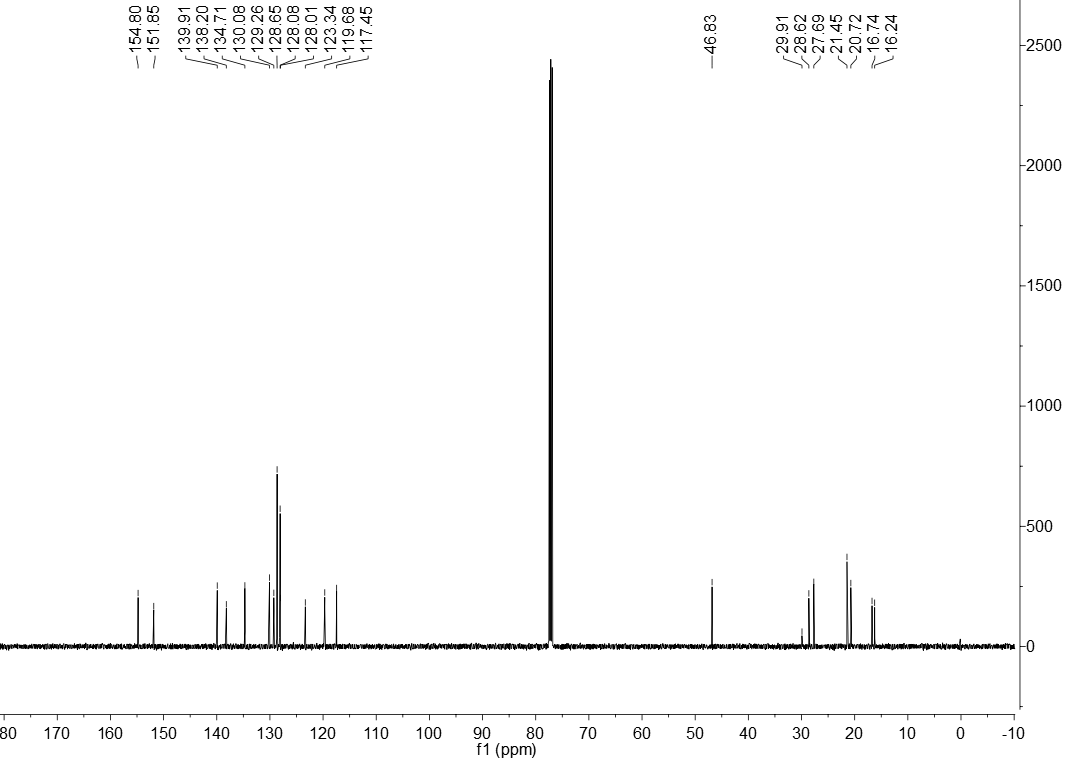


**FigureS16.** ^13^C NMR (126 MHz, CDCl_3_) of compound **4f**


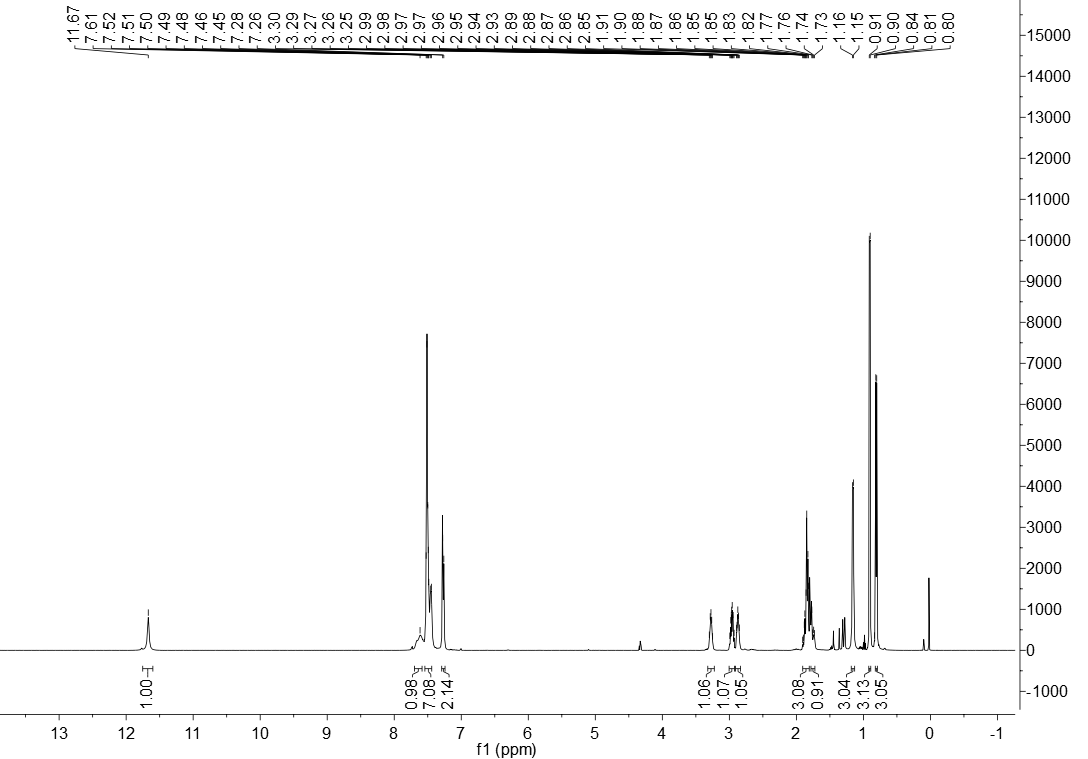


**FigureS17.** ^1^H NMR (500 MHz, CDCl_3_) of compound **4g**


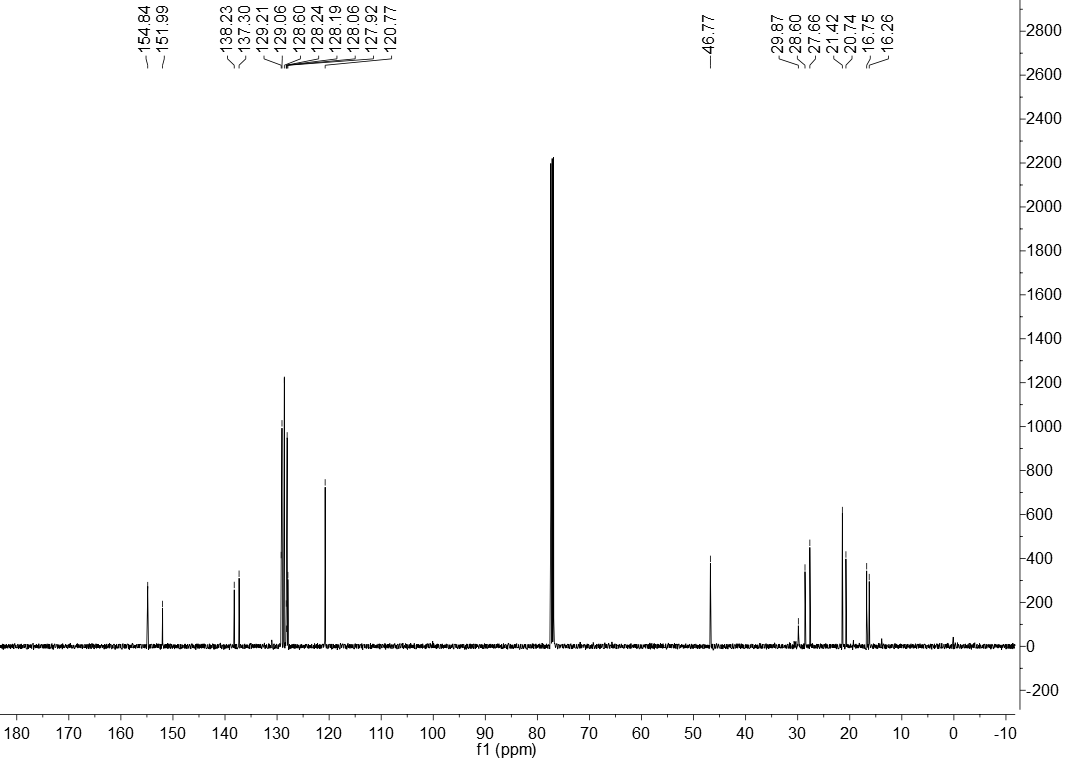


**FigureS18.** ^13^C NMR (126 MHz, CDCl_3_) of compound **4g**


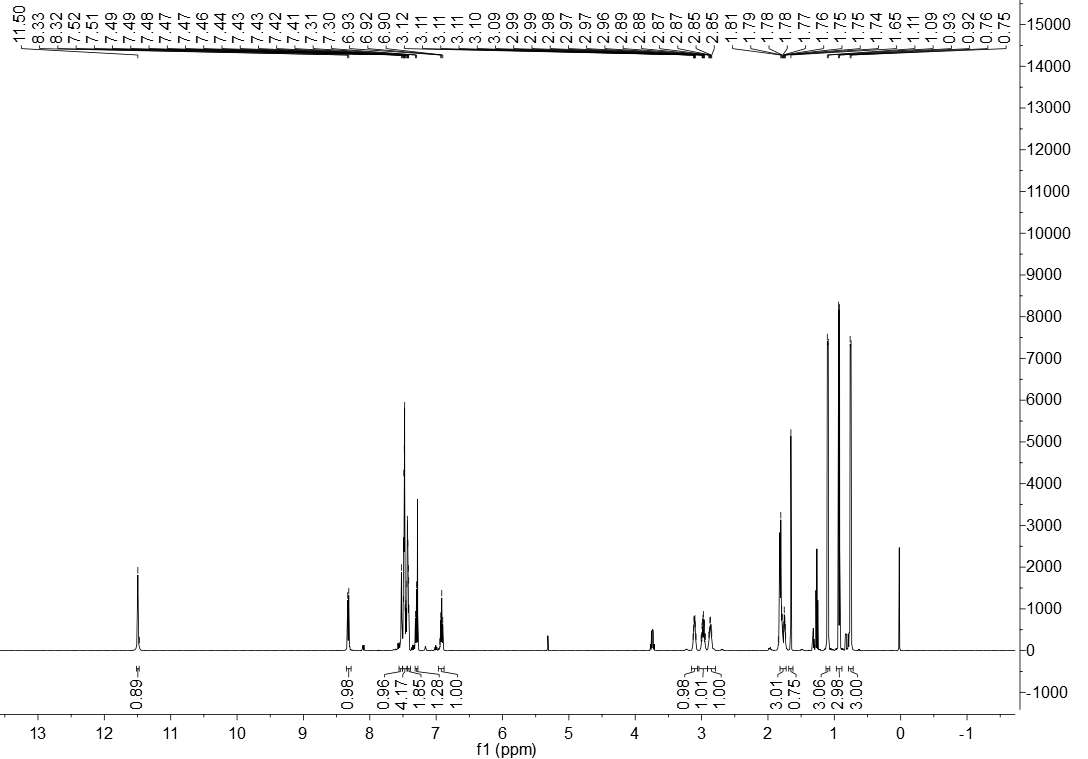


**FigureS19.** ^1^H NMR (500 MHz, CDCl_3_) of compound **4h**


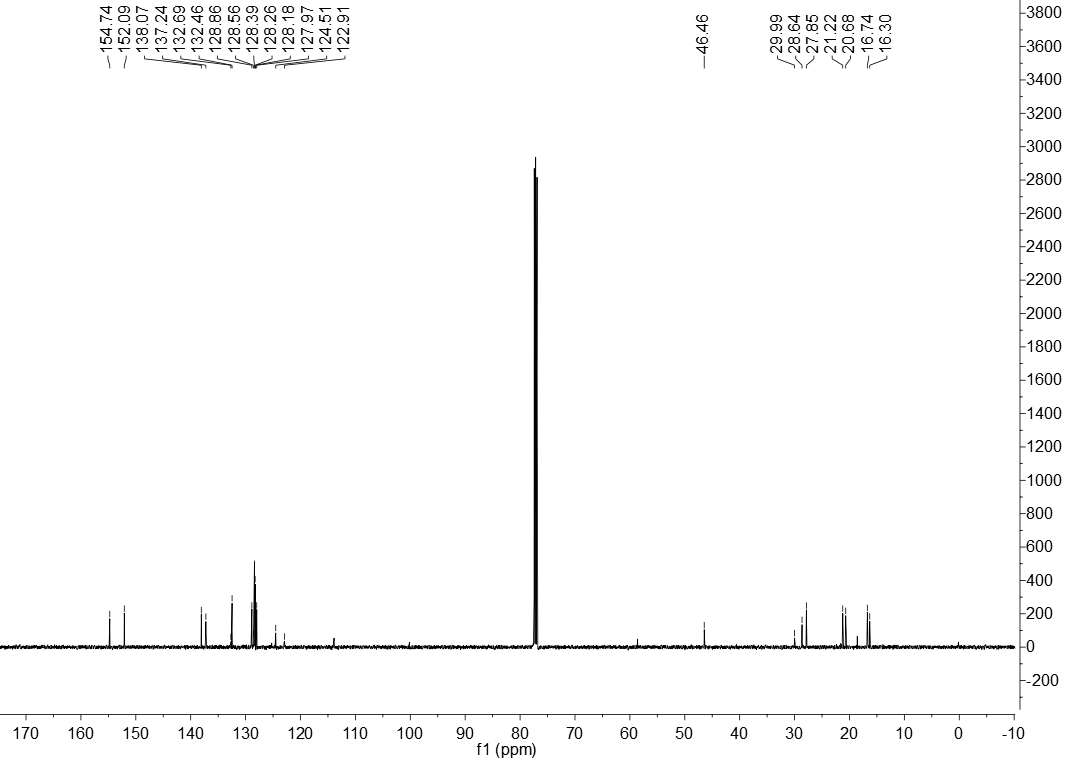


**FigureS20.** ^13^C NMR (126 MHz, CDCl_3_) of compound **4h**


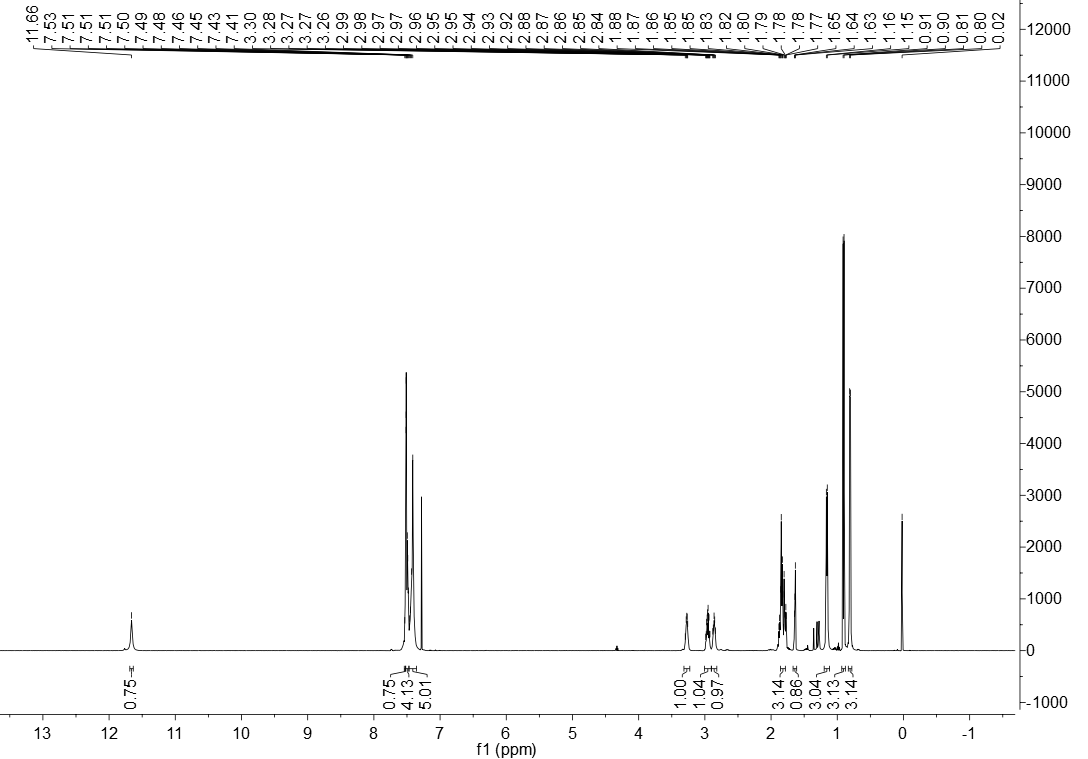


**FigureS21.** ^1^H NMR (500 MHz, CDCl_3_) of compound **4i**


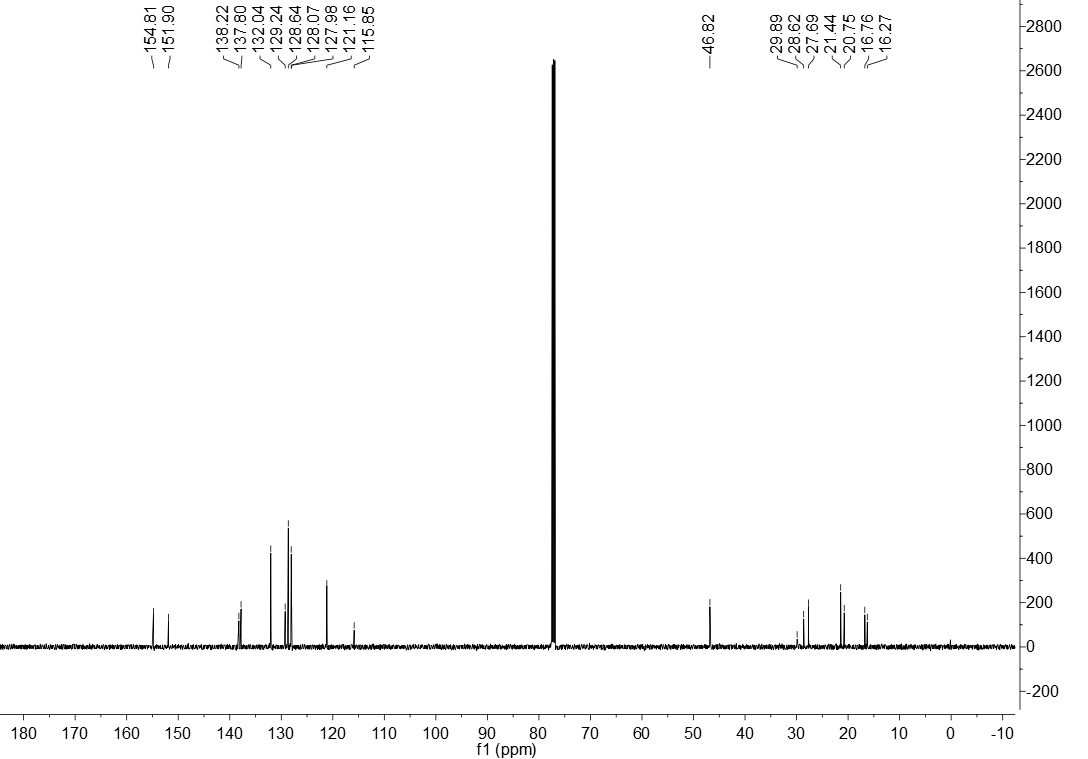


**FigureS22.** ^13^C NMR (126 MHz, CDCl_3_) of compound **4i**


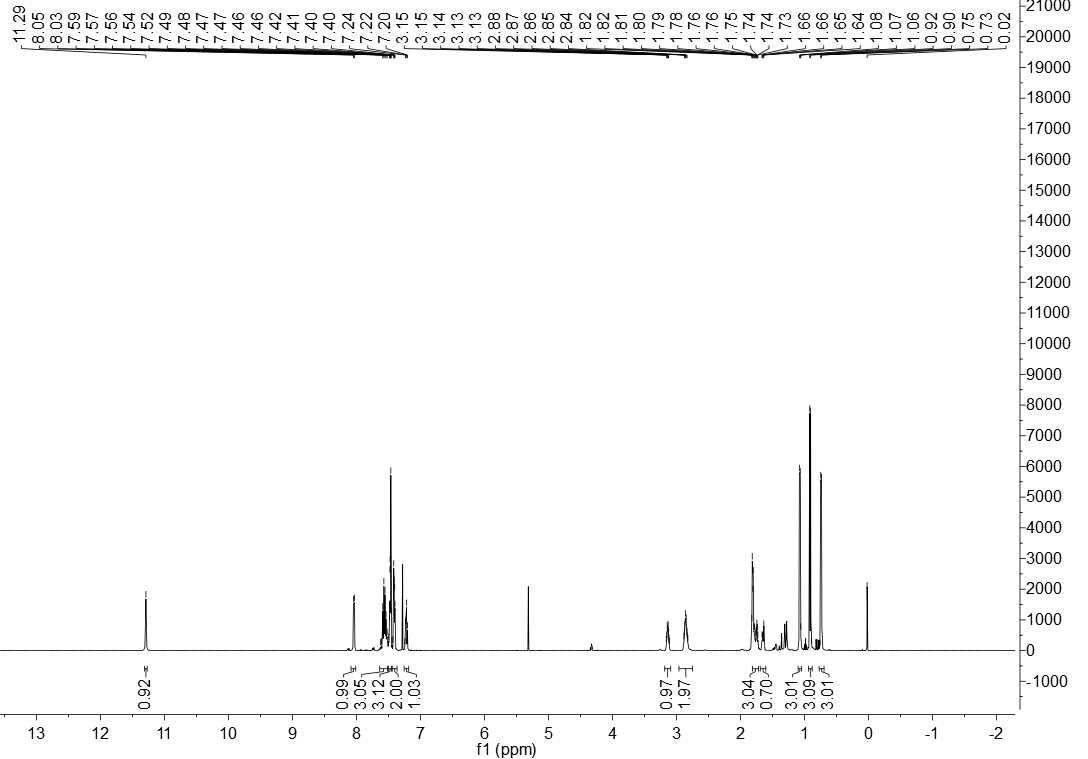


**FigureS23.** ^1^H NMR (500 MHz, CDCl_3_) of compound **4j**


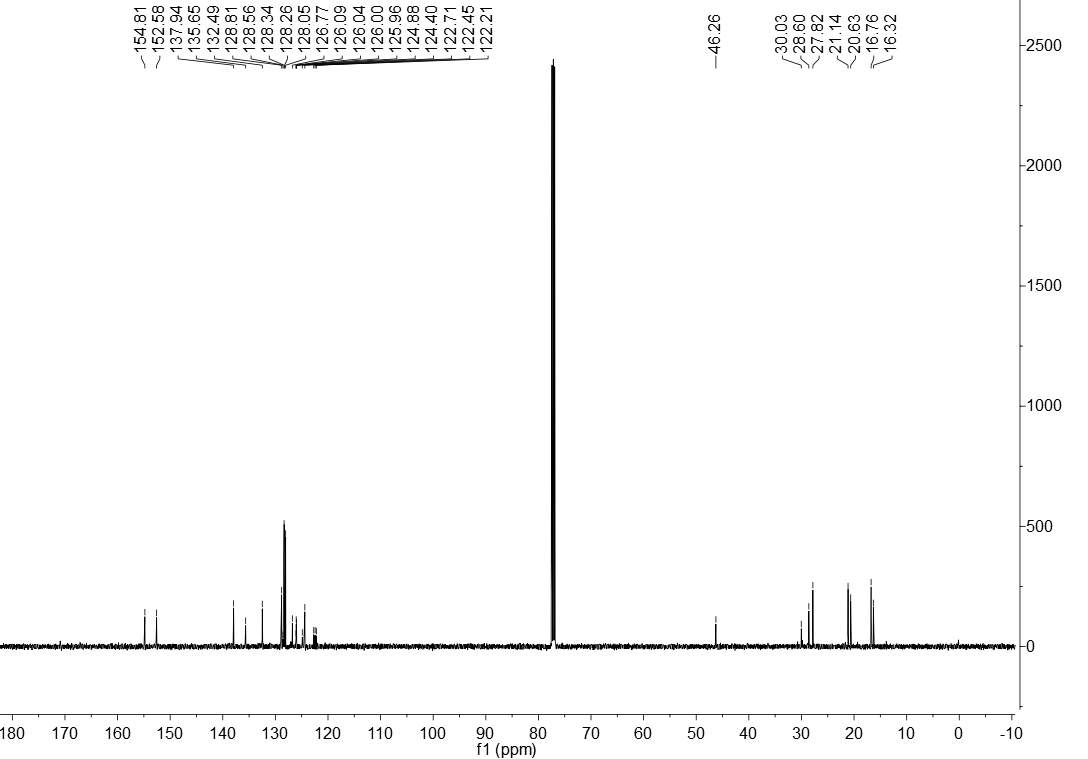


**FigureS24.** ^13^C NMR (126 MHz, CDCl_3_) of compound **4j**


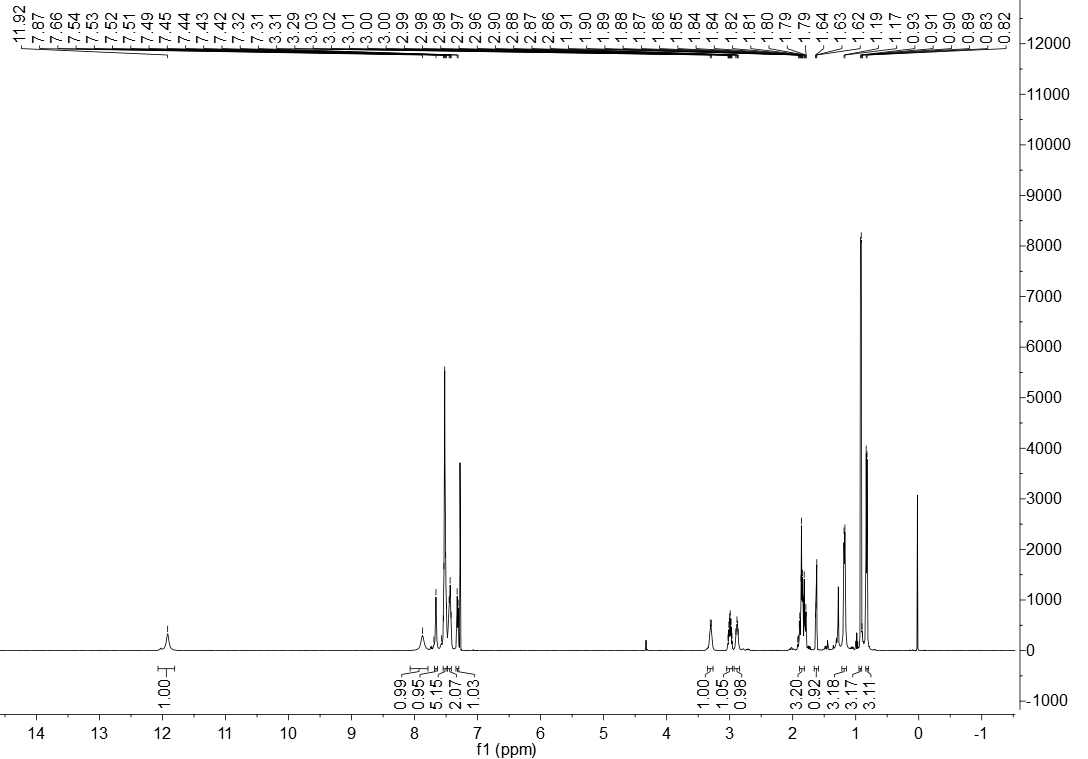


**FigureS25.** ^1^H NMR (500 MHz, CDCl_3_) of compound **4k**


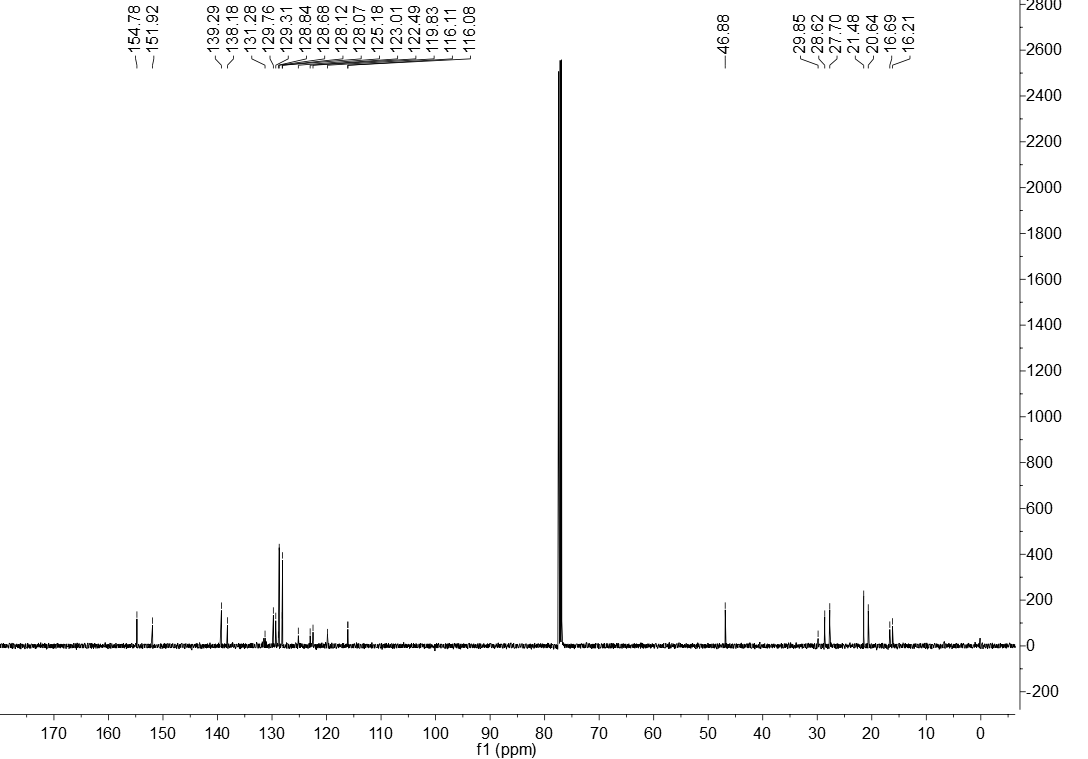


**FigureS26.** ^13^C NMR (126 MHz, CDCl_3_) of compound **4k**


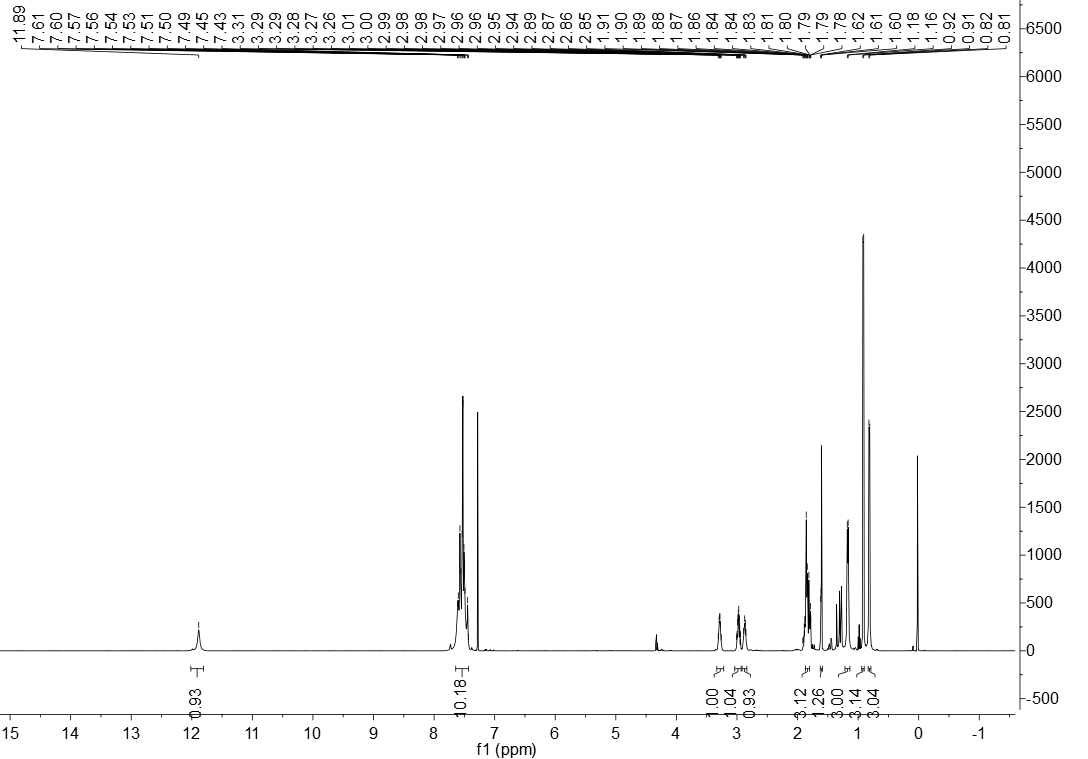


**FigureS27.** ^1^H NMR (500 MHz, CDCl_3_) of compound **4l**


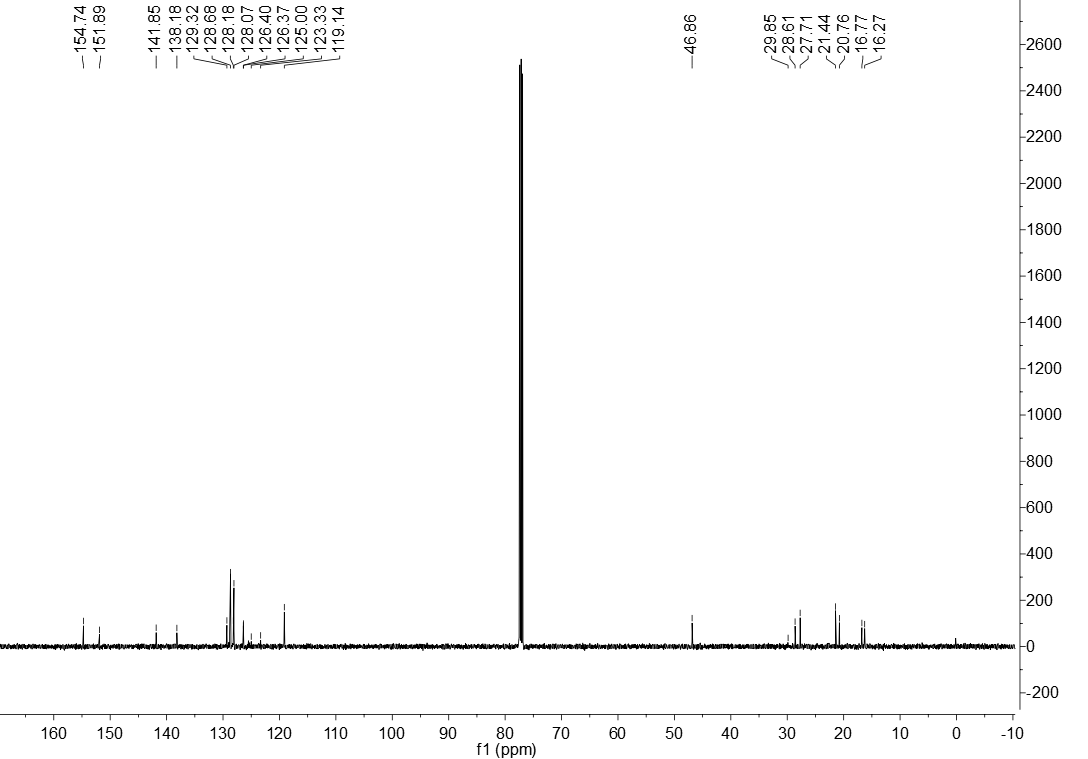


**FigureS28.** ^13^C NMR (126 MHz, CDCl_3_) of compound **4l**


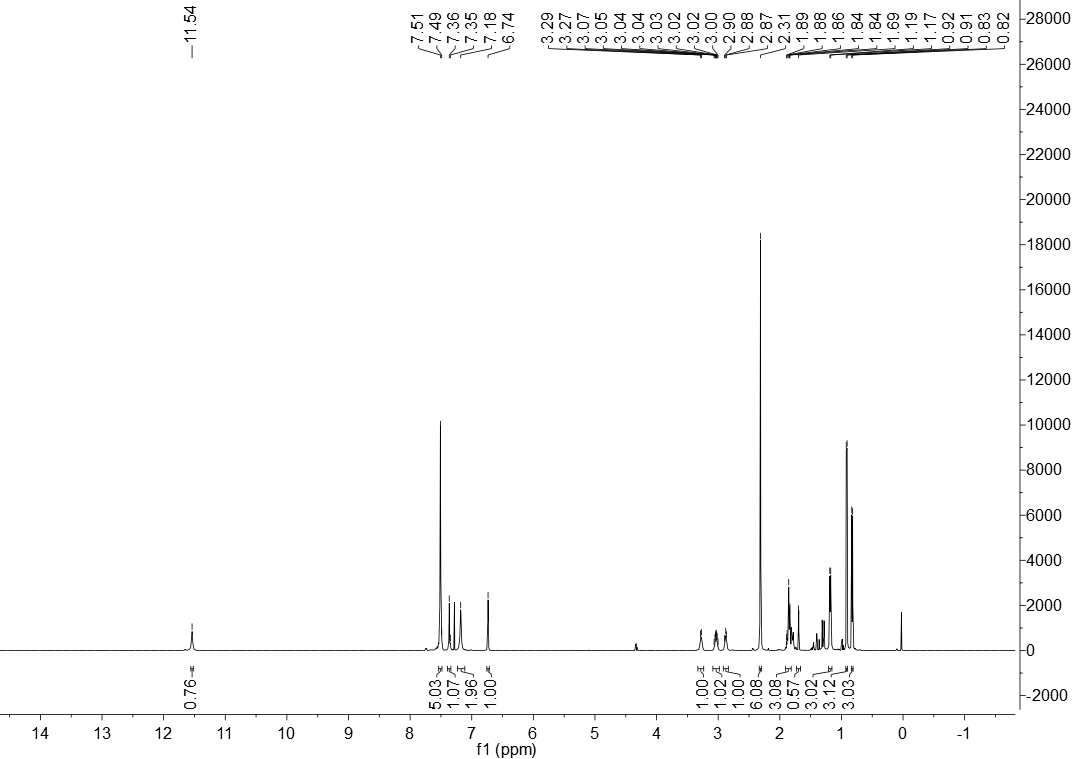


**FigureS29.** ^1^H NMR (500 MHz, CDCl_3_) of compound **4m**


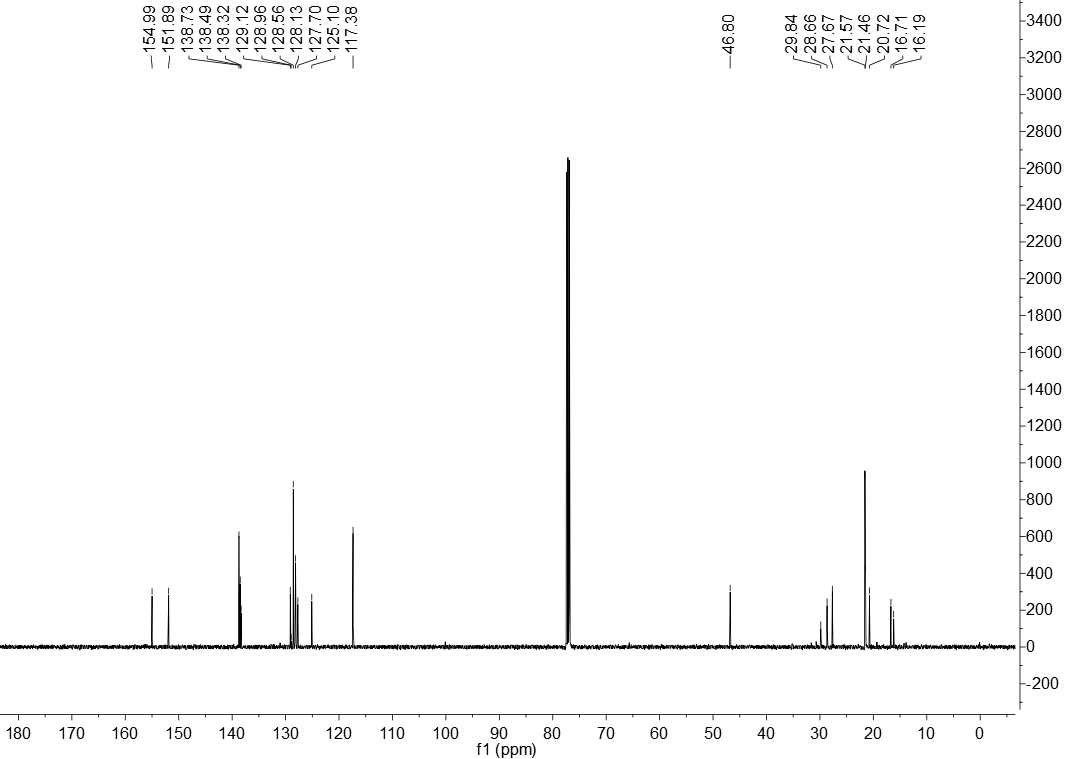


**FigureS30.** ^13^C NMR (126 MHz, CDCl_3_) of compound **4m**


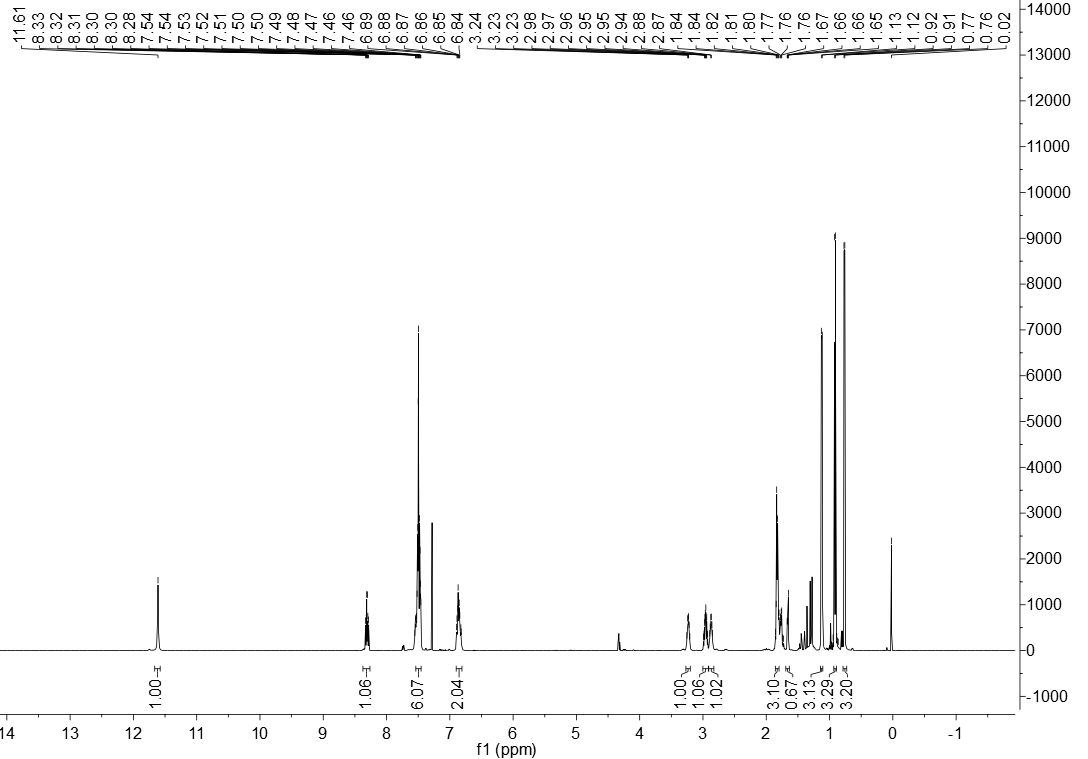


**FigureS31.** ^1^H NMR (500 MHz, CDCl_3_) of compound **4n**


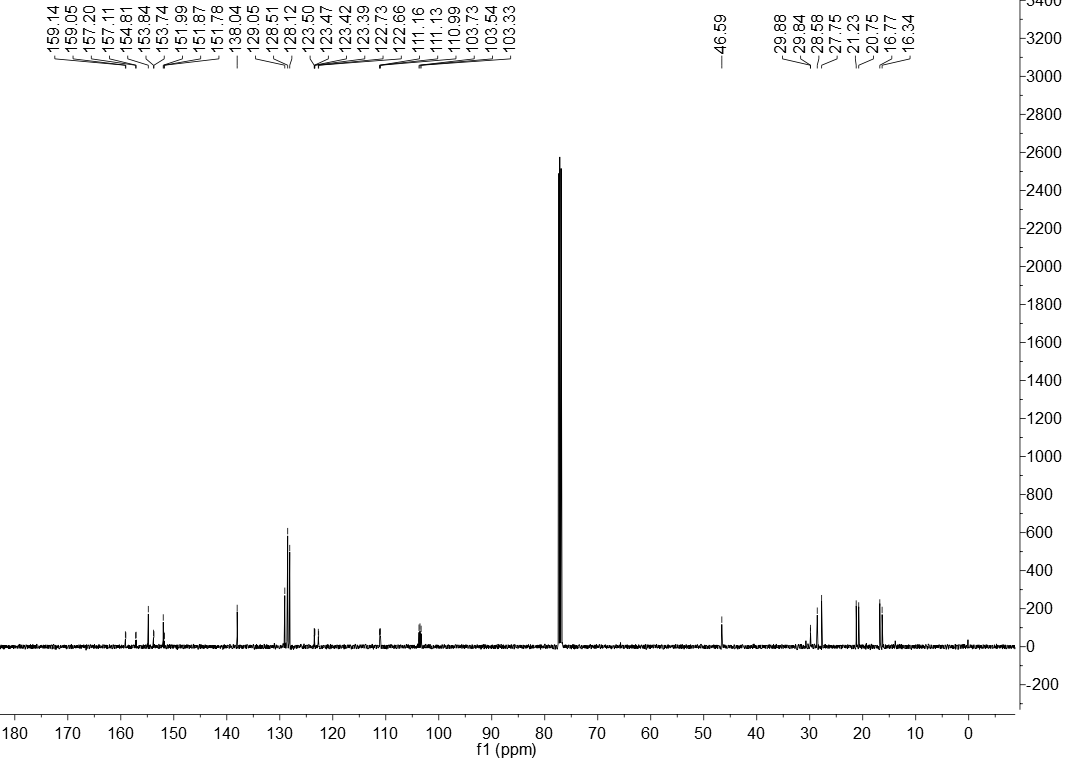


**FigureS32.** ^13^C NMR (126 MHz, CDCl_3_) of compound **4n**


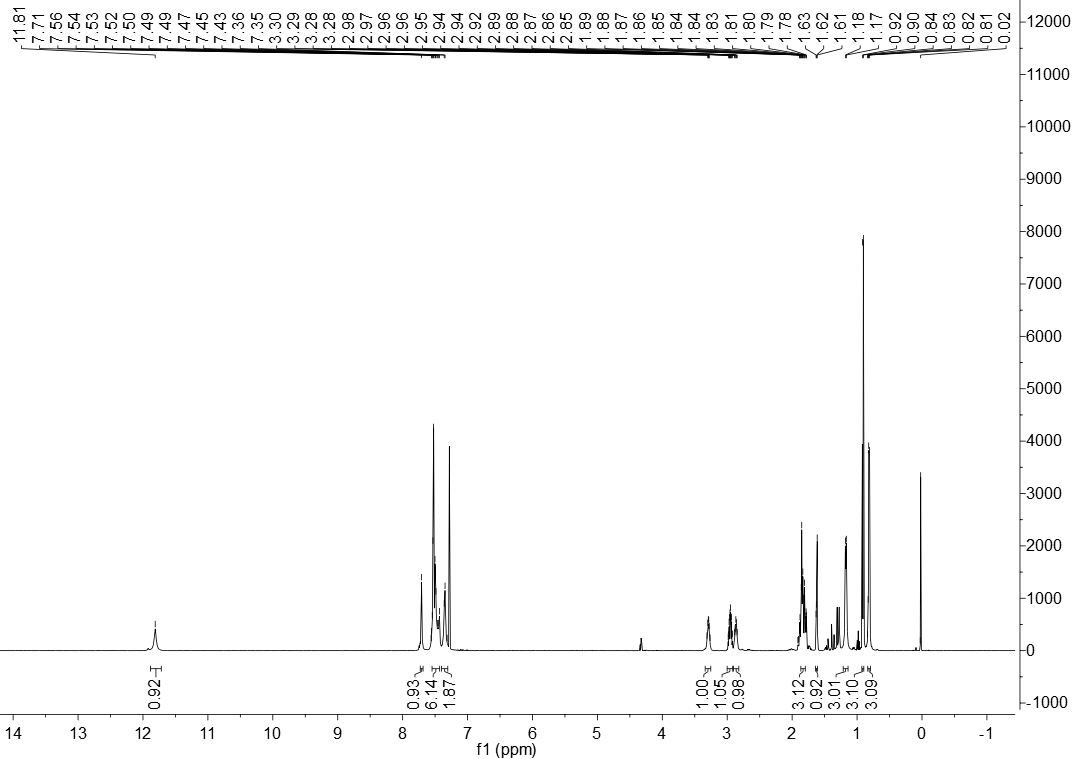


**FigureS33.** ^1^H NMR (500 MHz, CDCl_3_) of compound **4o**


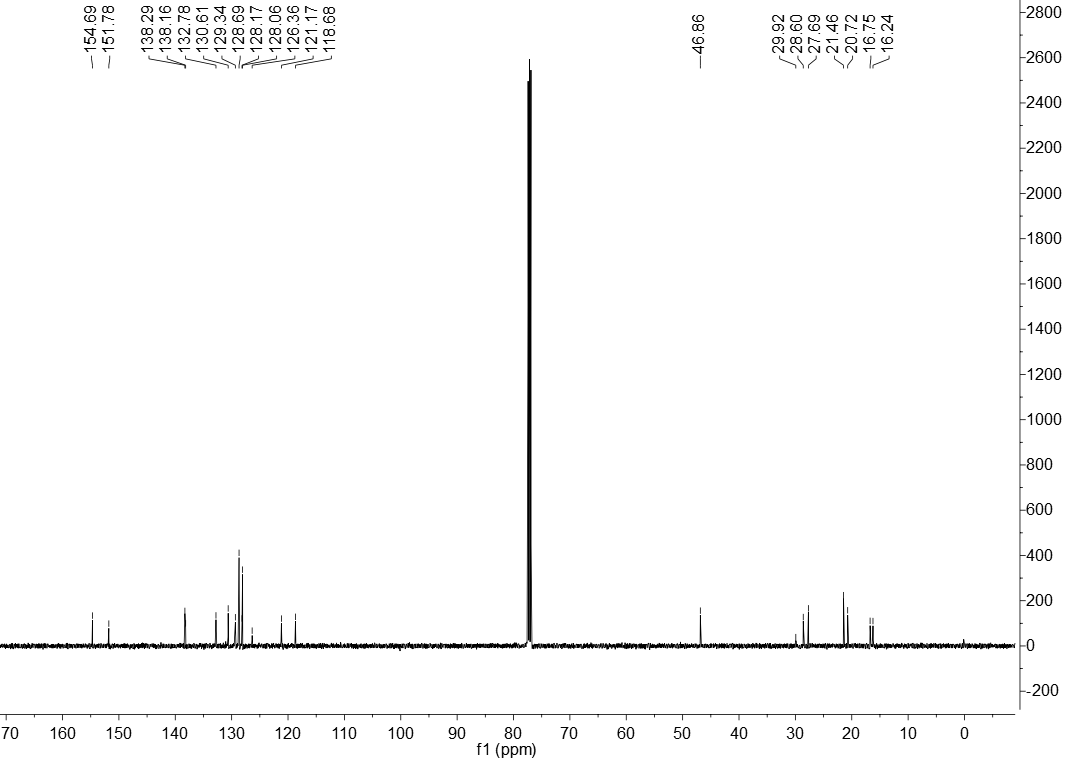


**FigureS34.** ^13^C NMR (126 MHz, CDCl_3_) of compound **4o**


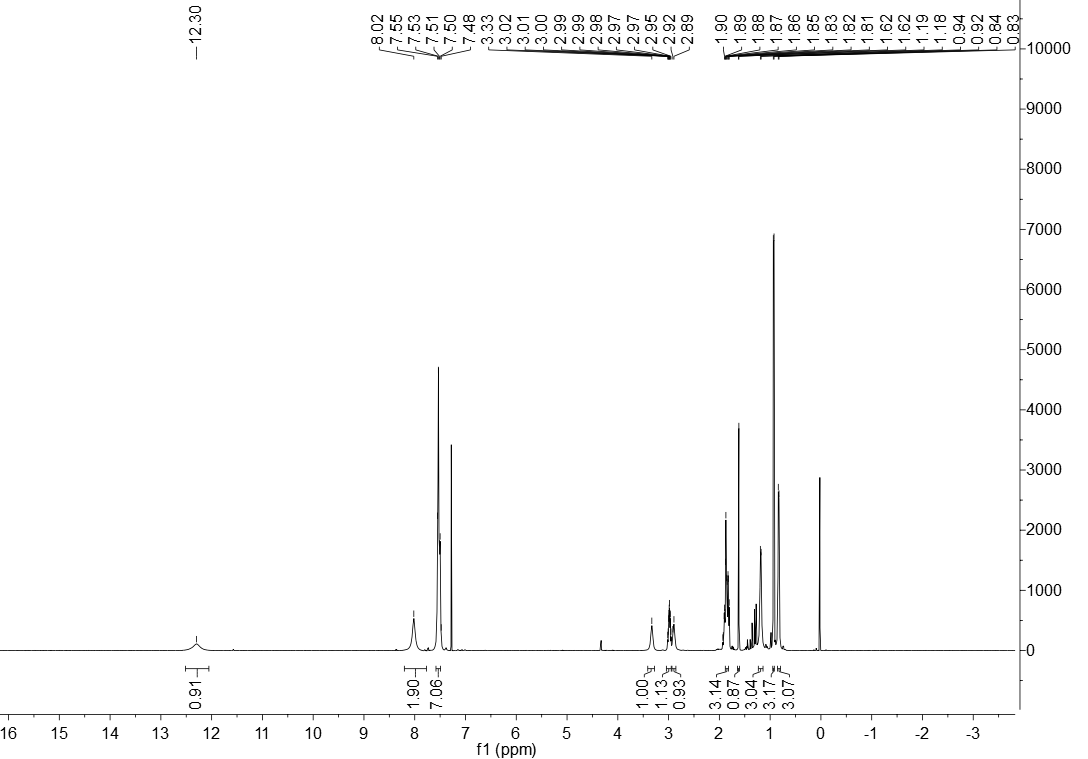


**FigureS35.** ^1^H NMR (500 MHz, CDCl_3_) of compound **4p**


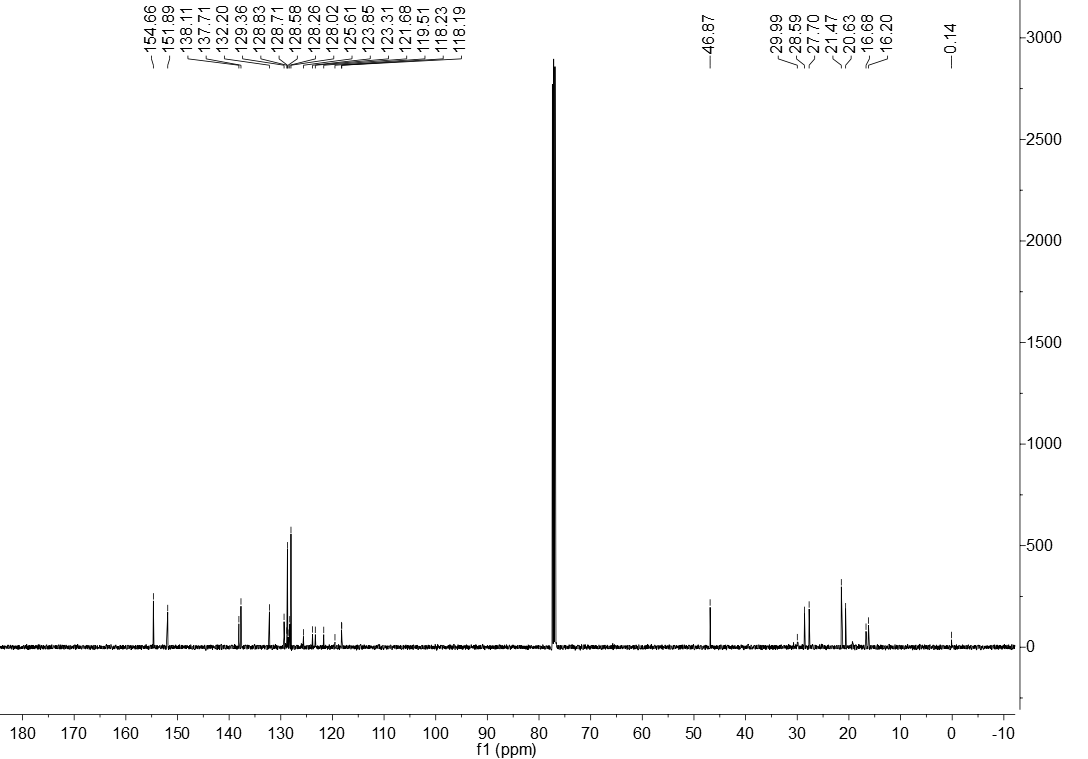


**FigureS36.** ^13^C NMR (125 MHz, CDCl_3_) of compound **4p**


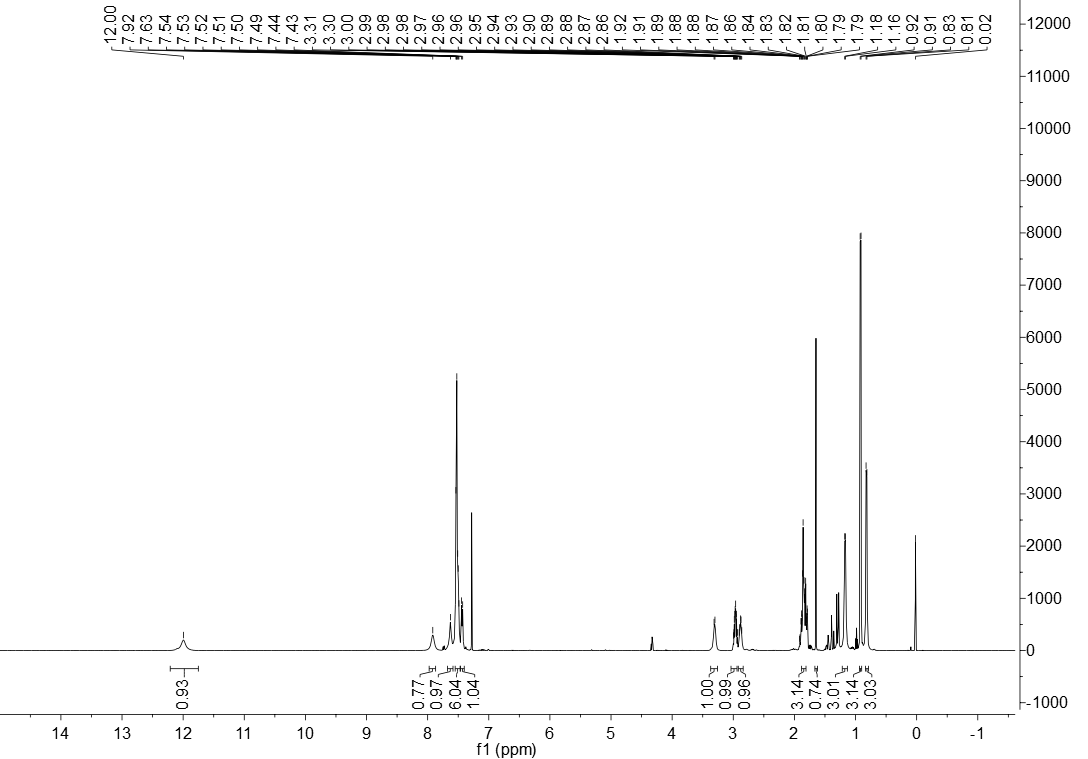


**FigureS37.** ^1^H NMR (500 MHz, CDCl_3_) of compound **4q**


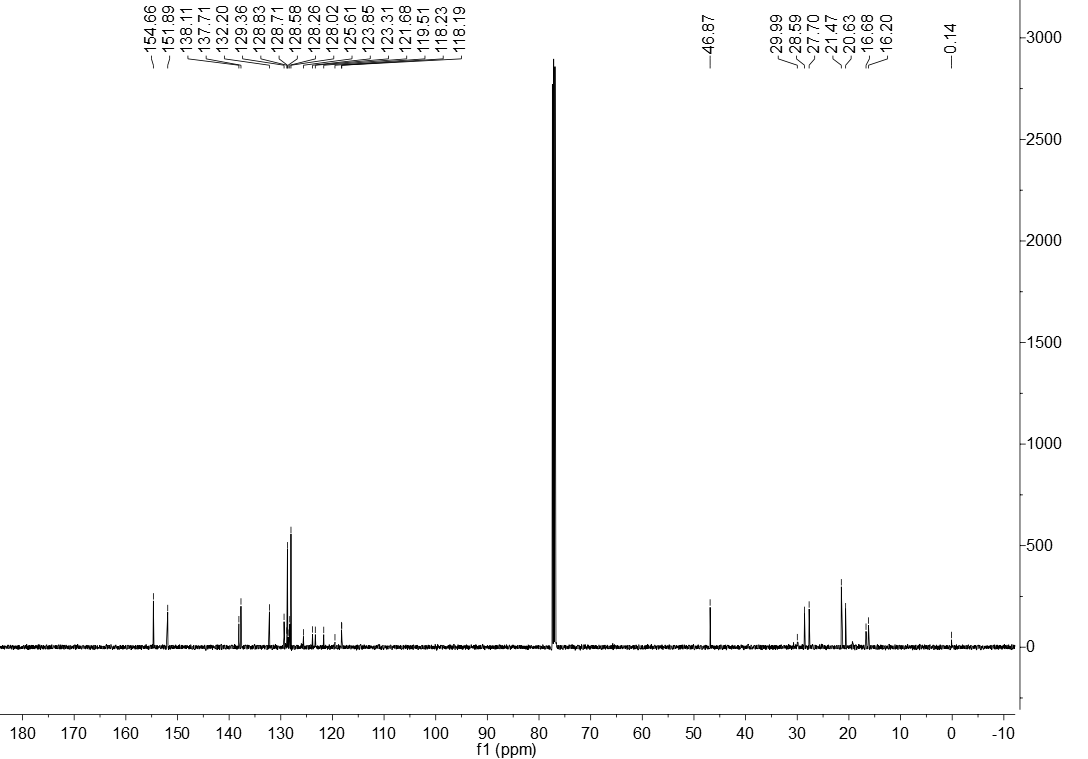


**FigureS38.** ^13^C NMR (126 MHz, CDCl_3_) of compound **4q**


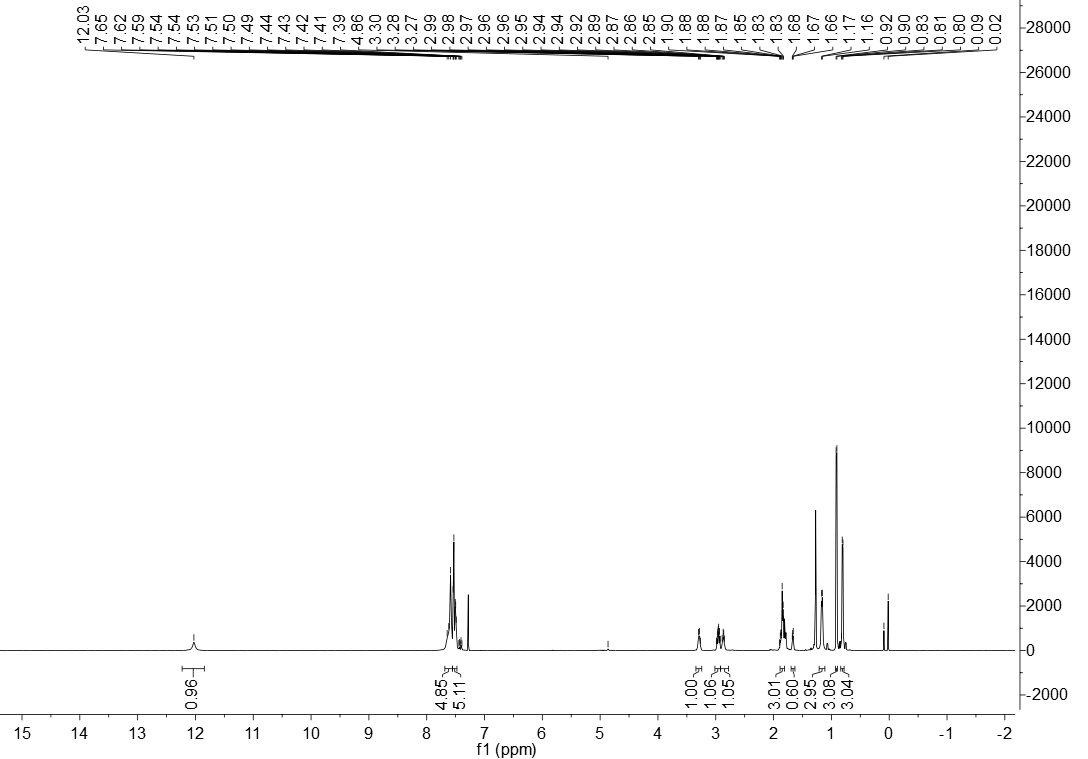


**FigureS39.** ^1^H NMR (500 MHz, CDCl_3_) of compound **4r**


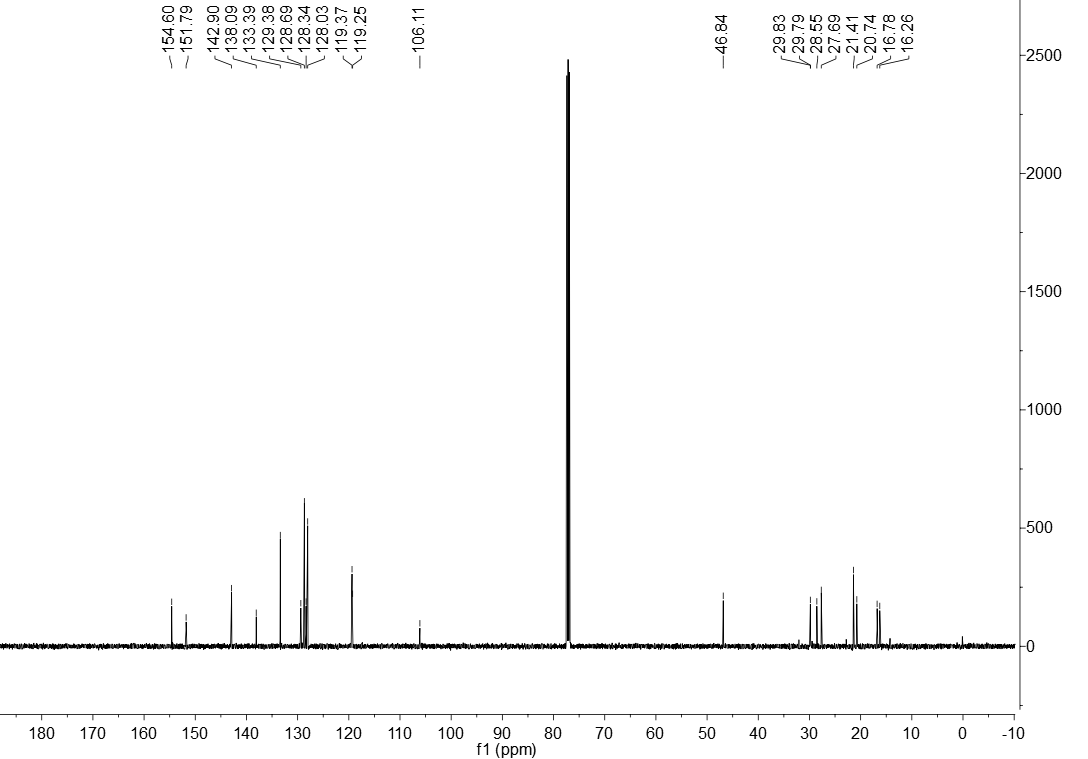


**FigureS40.** ^13^C NMR (126 MHz, CDCl_3_) of compound **4r**


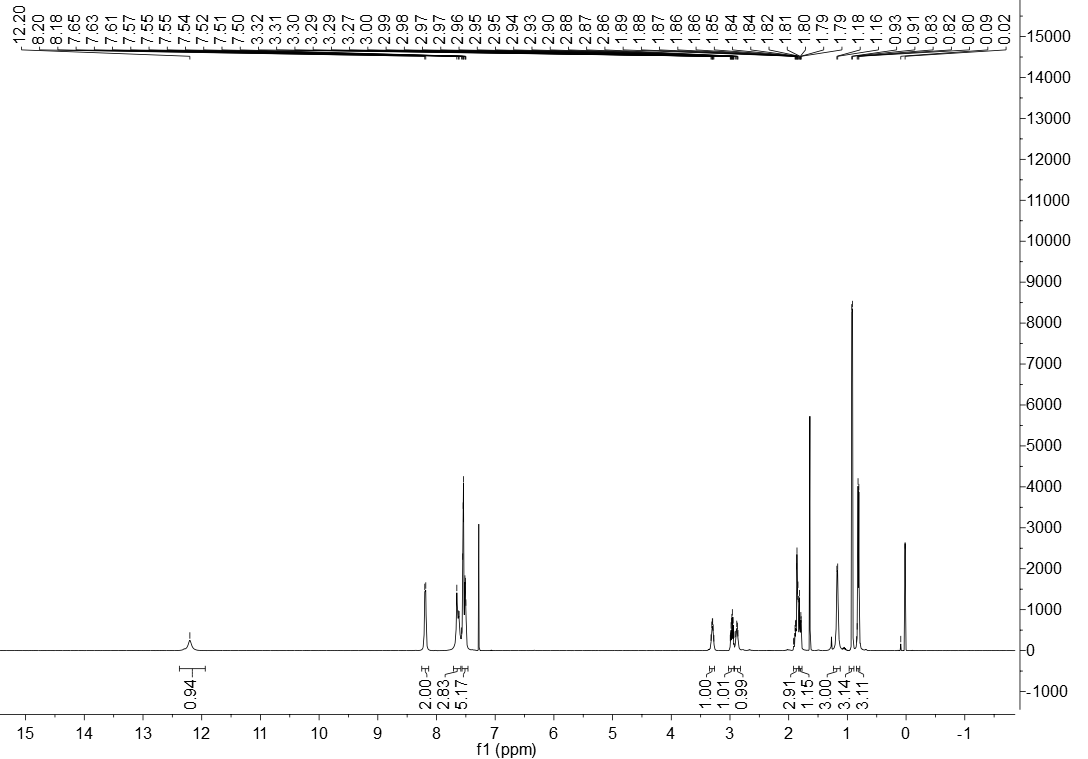


**FigureS41.** ^1^H NMR (500 MHz, CDCl_3_) of compound **4s**


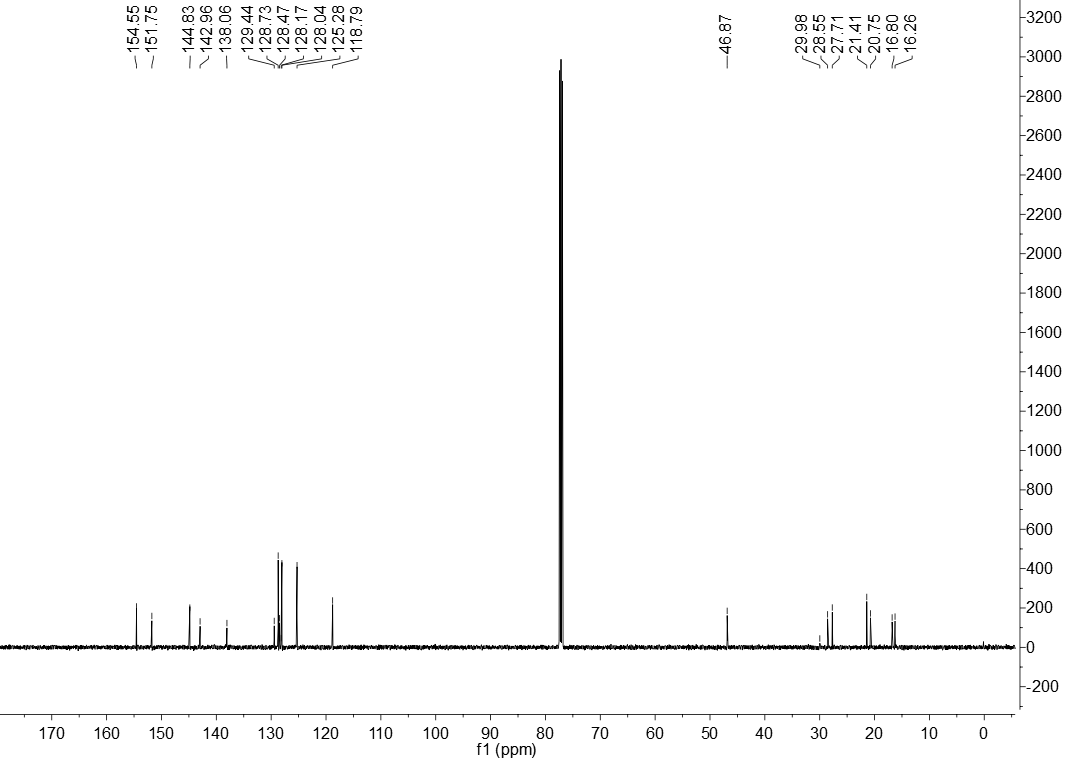


**FigureS42.** ^13^C NMR (126 MHz, CDCl_3_) of compound **4s**

**FT-IR-Spectra**

**FigureS43.** FT-IR spectra of compound **3**

**FigureS44.** FT-IR spectra of compound **4a**

**FigureS45.** FT-IR spectra of compound **4b**

**FigureS46.** FT-IR spectra of compound **4c**

**FigureS47.** FT-IR spectra of compound **4d**

**FigureS48.** FT-IR spectra of compound **4e**

**FigureS49.** FT-IR spectra of compound **4f**

**FigureS50.** FT-IR spectra of compound **4g**

**FigureS51.** FT-IR spectra of compound **4h**

**FigureS52.** FT-IR spectra of compound **4i**

**FigureS53.** FT-IR spectra of compound **4j**

**FigureS54.** FT-IR spectra of compound **4k**

**FigureS55.** FT-IR spectra of compound **4l**

**FigureS56.** FT-IR spectra of compound **4m**

**FigureS57.** FT-IR spectra of compound **4n**

**FigureS58.** FT-IR spectra of compound **4o**

**FigureS59.** FT-IR spectra of compound **4p**

**FigureS60.** FT-IR spectra of compound **4q**

**FigureS61.** FT-IR spectra of compound **4r**

**FigureS62.** FT-IR spectra of compound **4s**

**HRMS-Spectra**

**FigureS63. HR**MS spectra of compound **3**

**
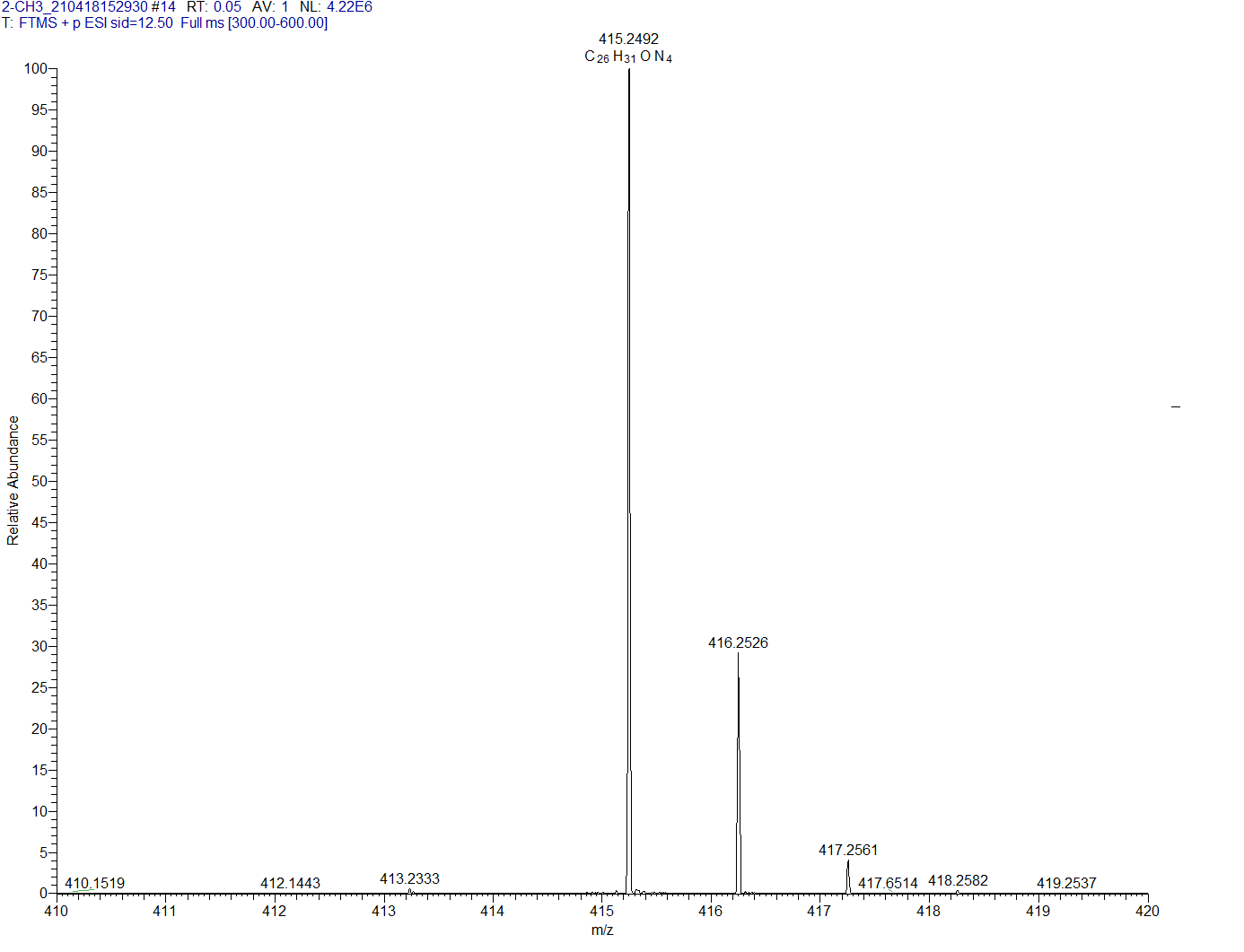
**

**FigureS64. HR**MS spectra of compound **4a**

**
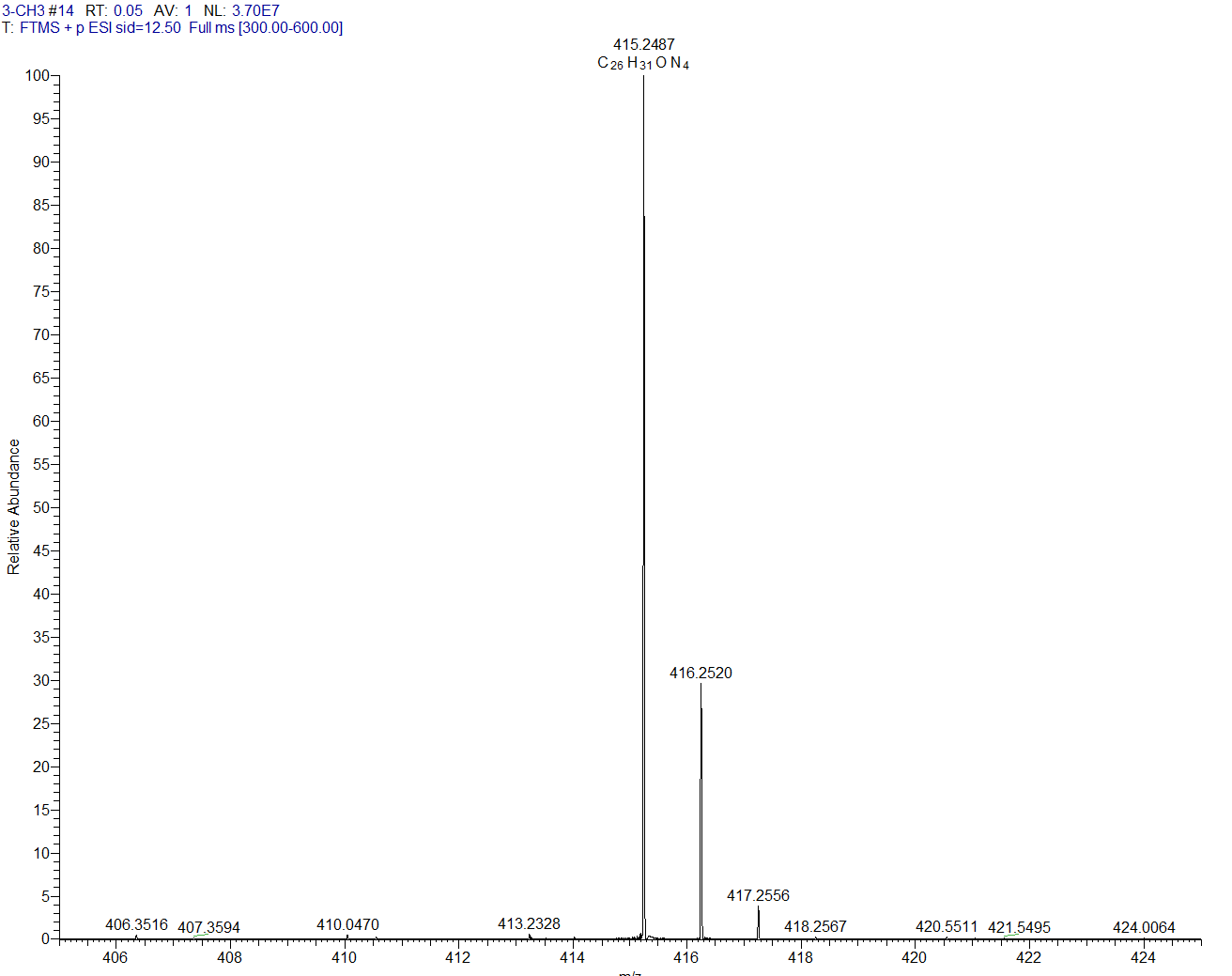
**

**FigureS65. HR**MS spectra of compound **4b**

**
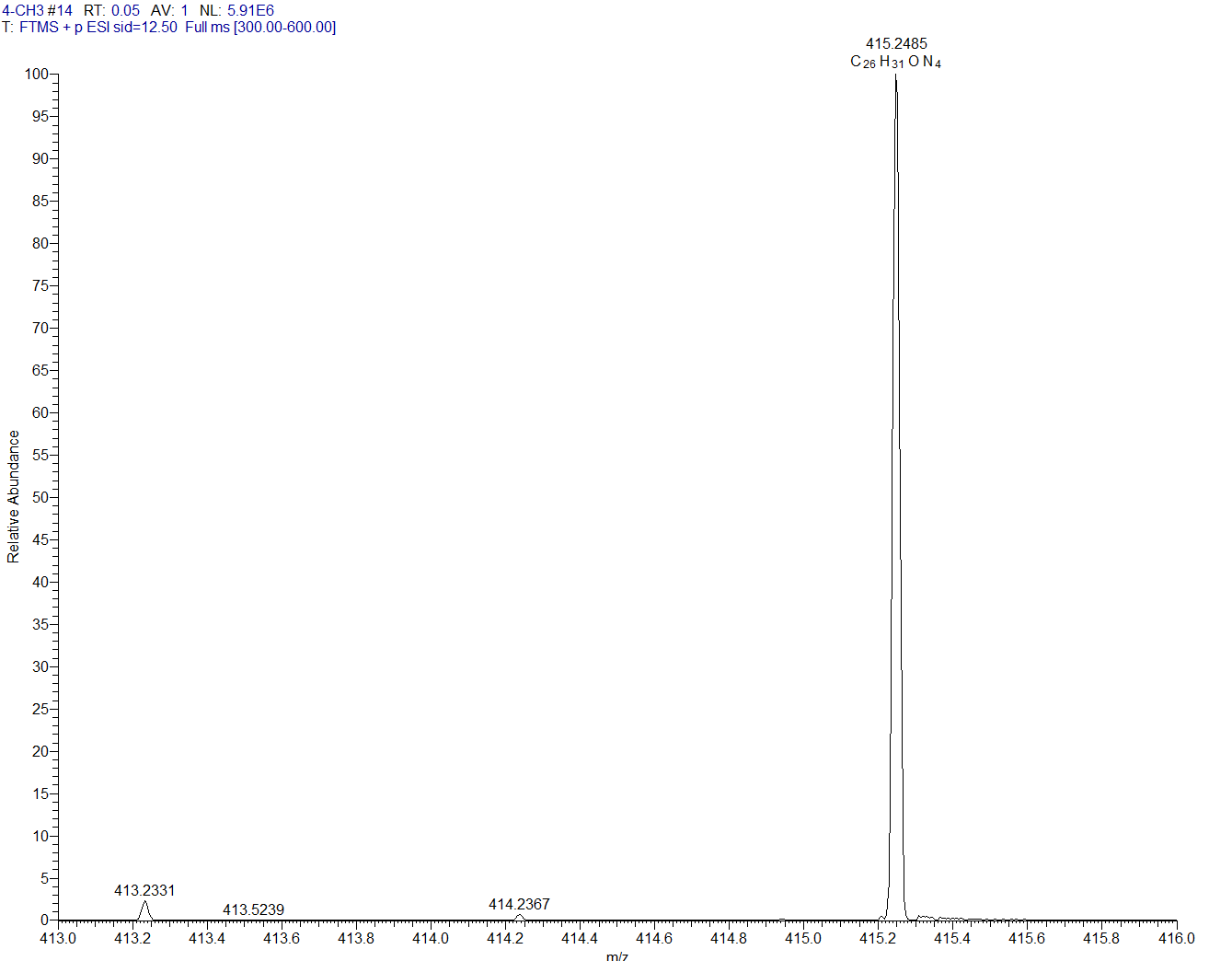
**

**FigureS66. HR**MS spectra of compound **4c**


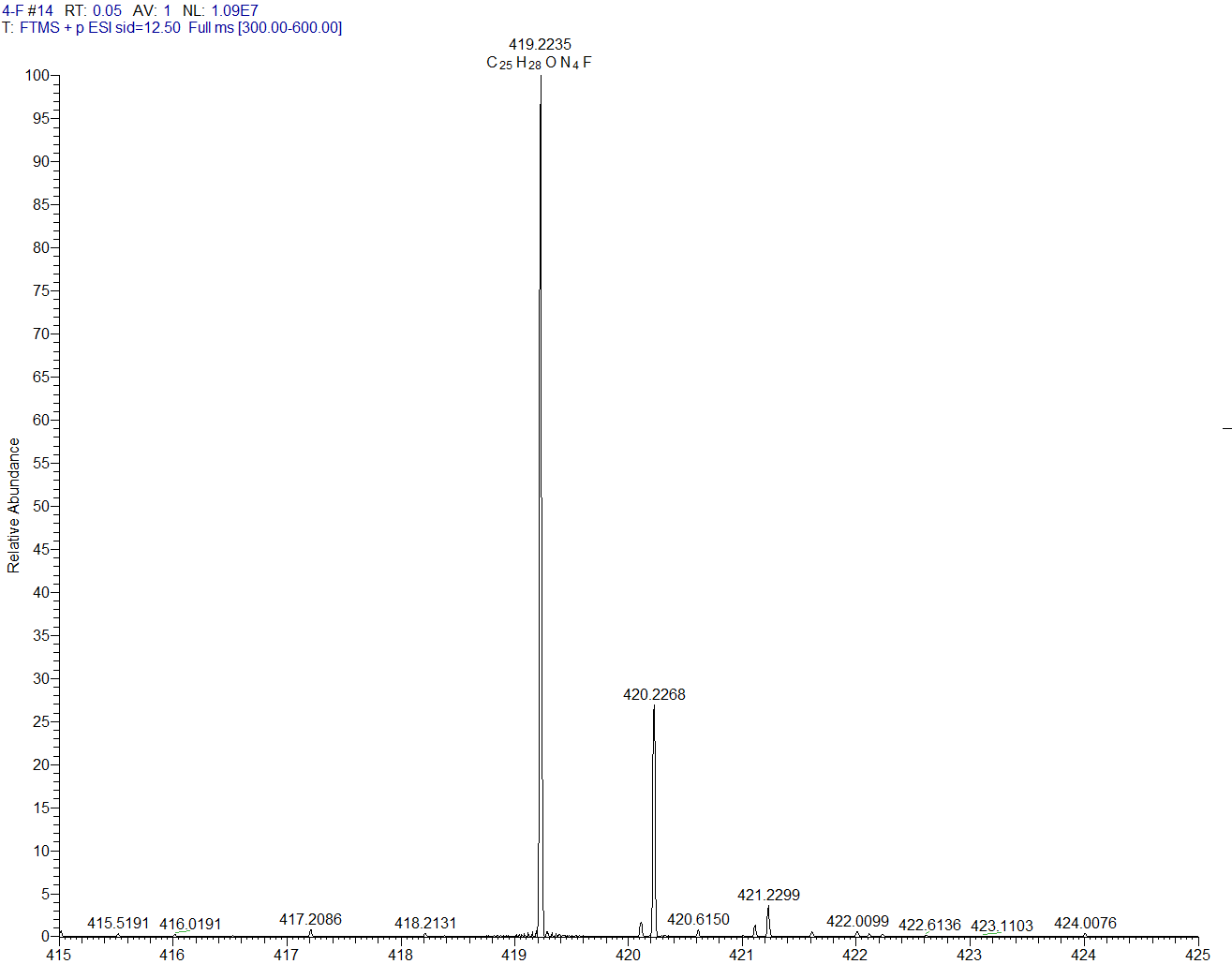


**FigureS67. HR**MS spectra of compound **4d**

**
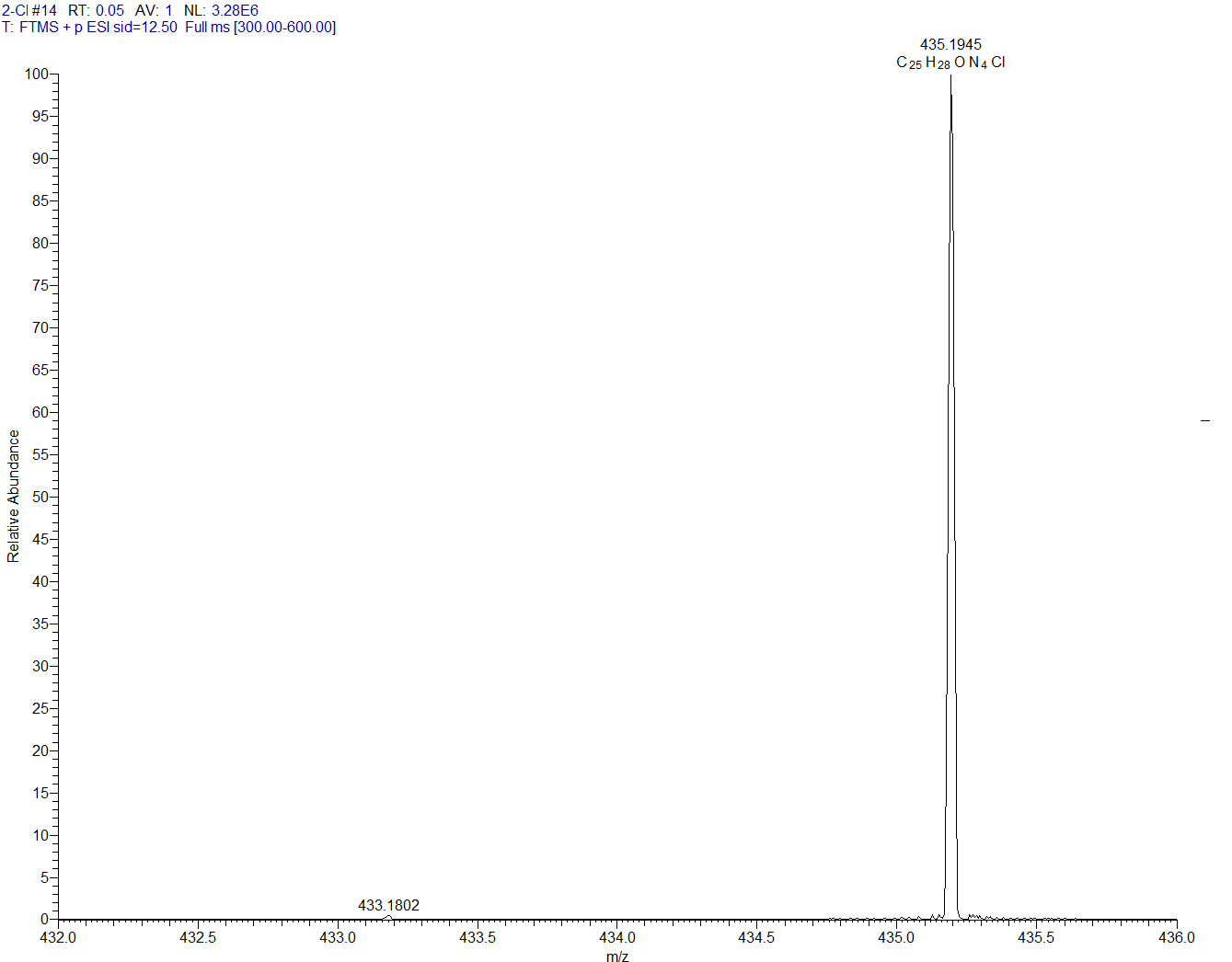
**

**FigureS68. HR**MS spectra of compound **4e**

**
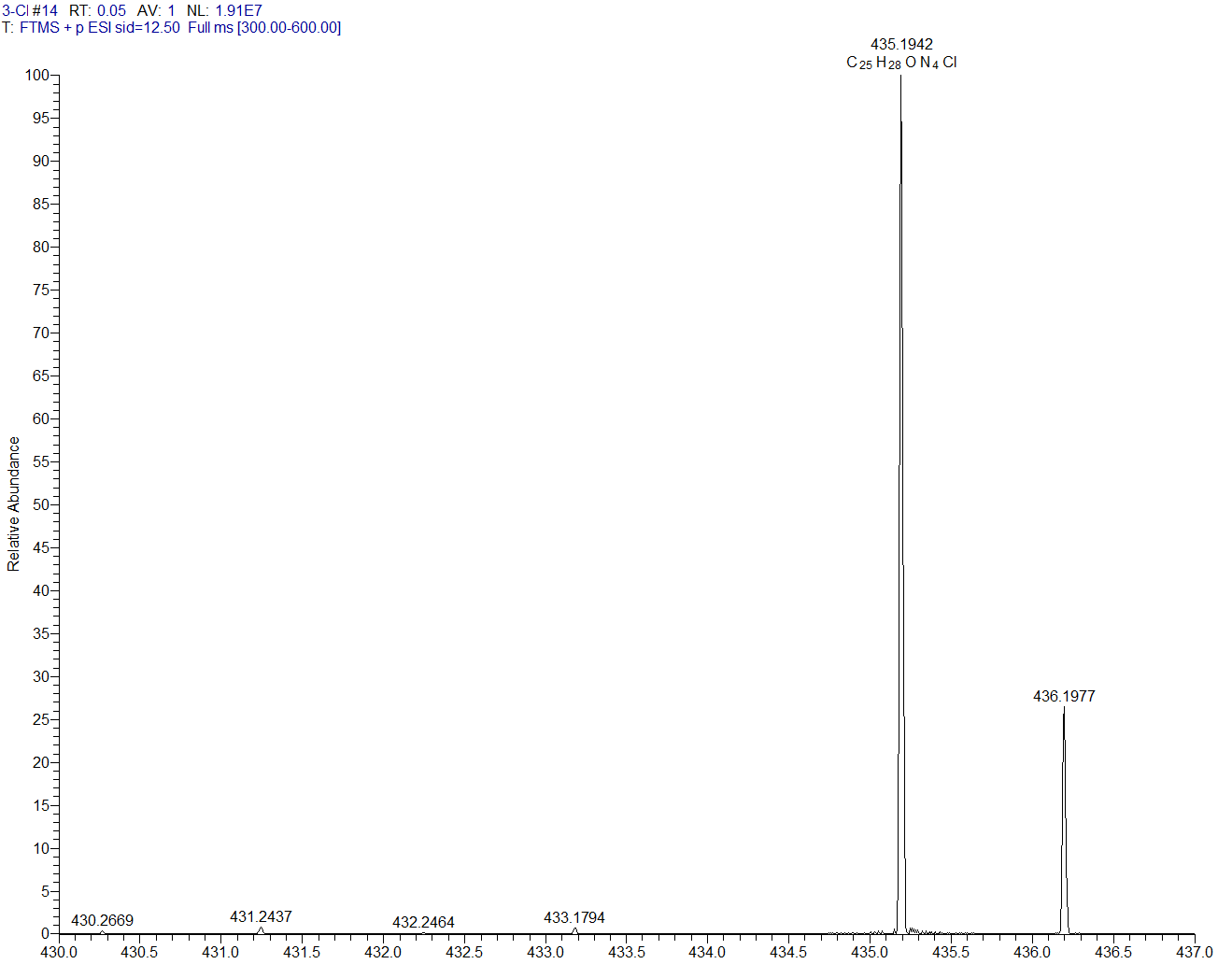
**

**FigureS69. HR**MS spectra of compound **4f**

**
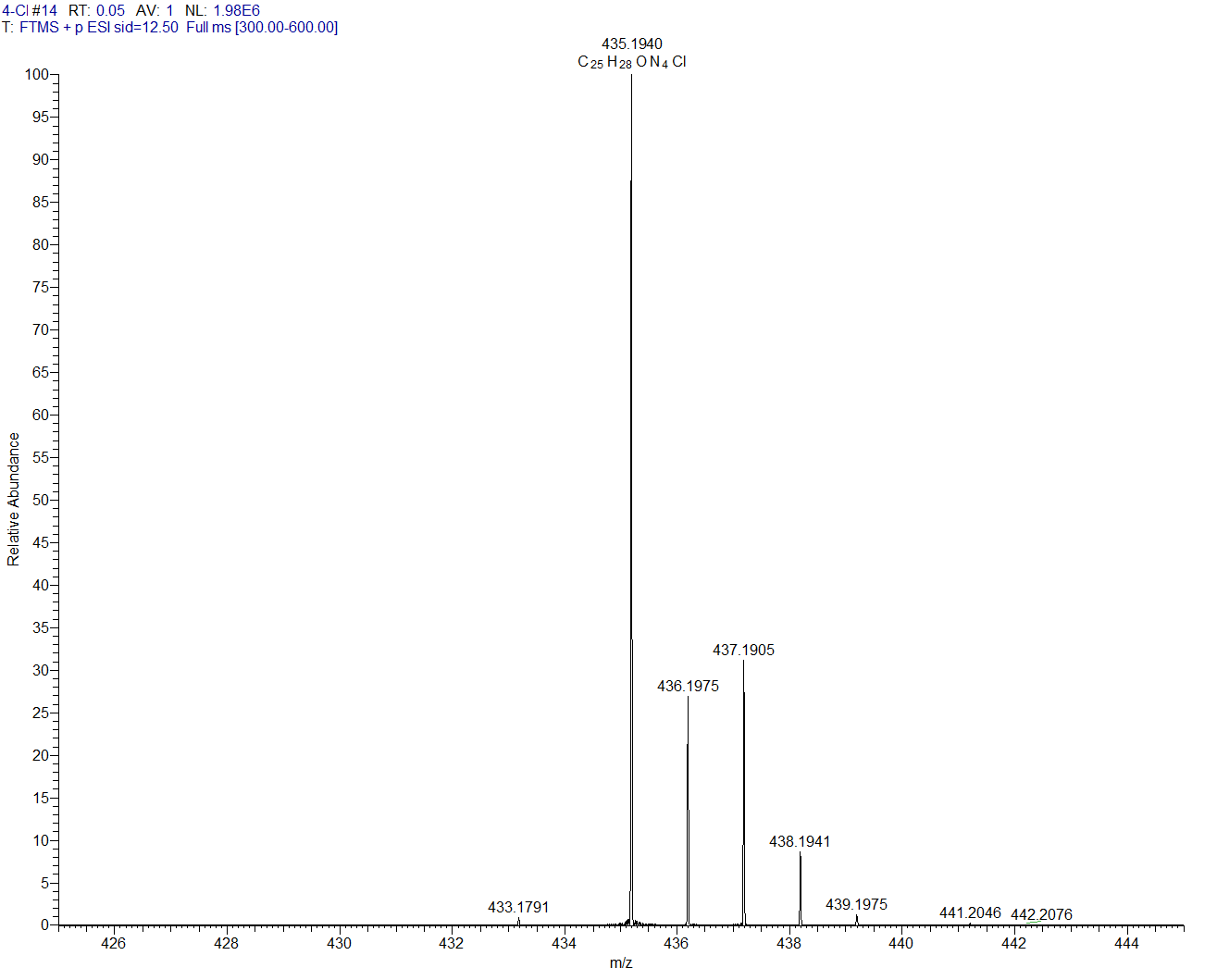
**

**FigureS70. HR**MS spectra of compound **4g**

**
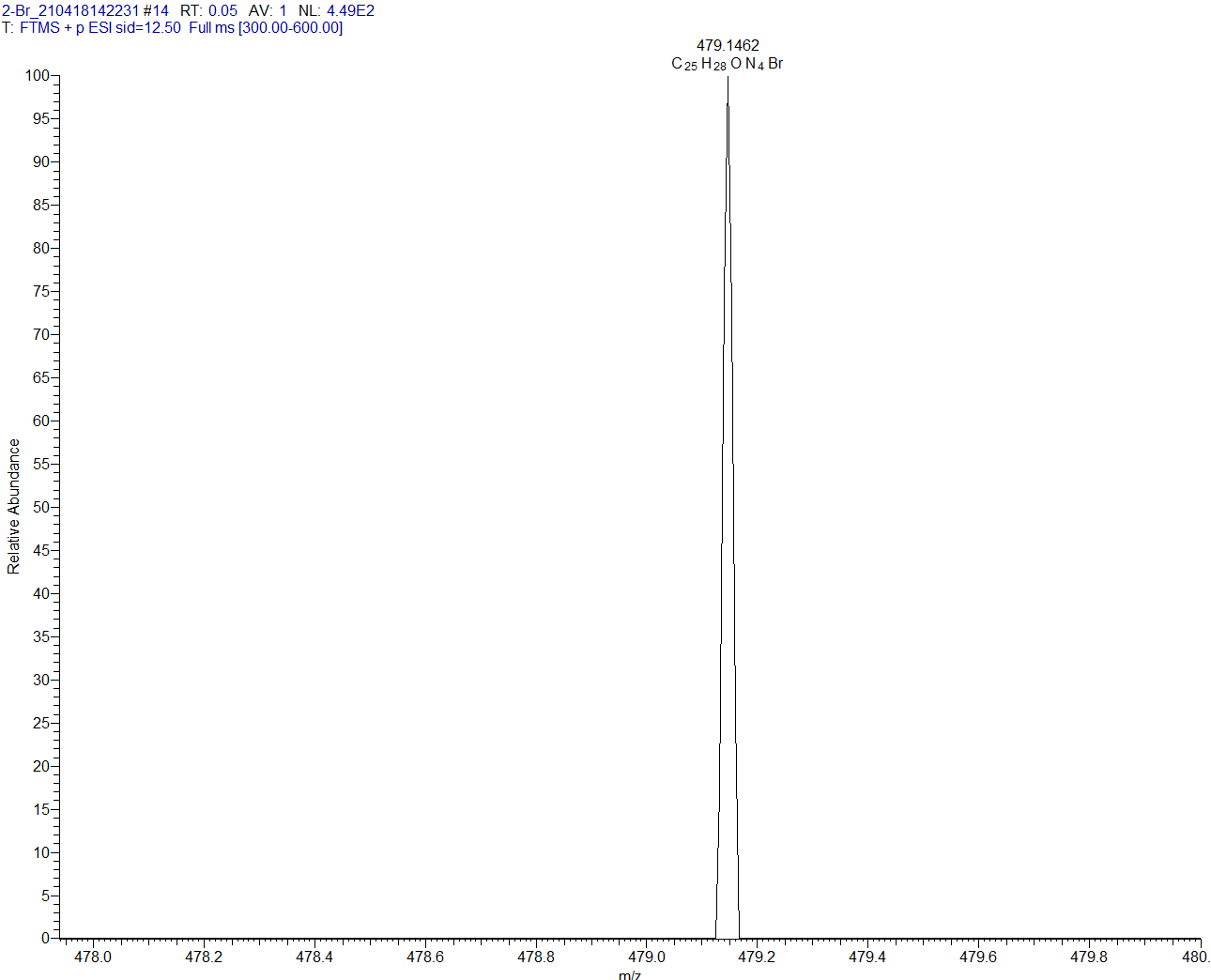
**

**FigureS71. HR**MS spectra of compound **4h**

**
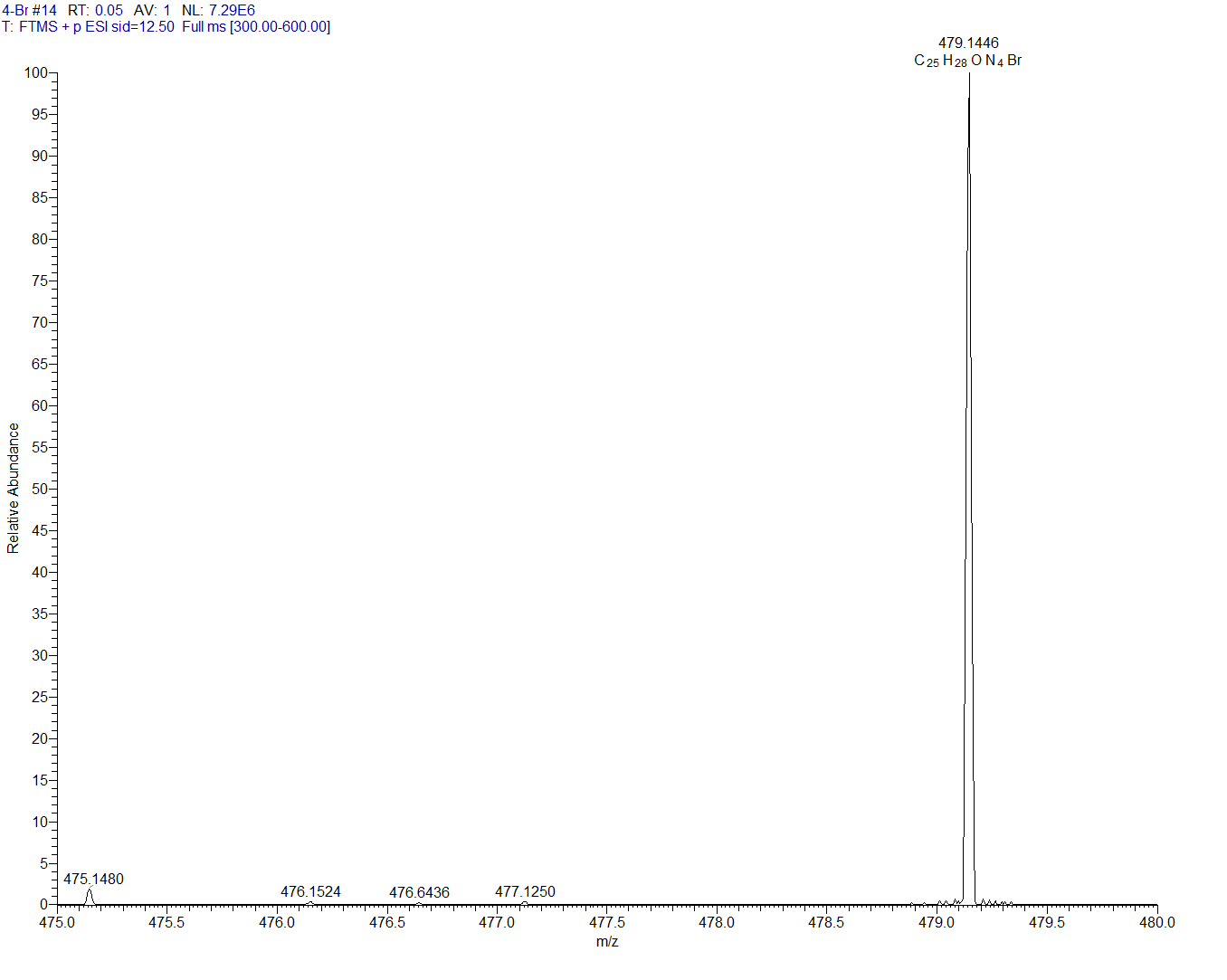
**

**FigureS72. HR**MS spectra of compound **4i**

**
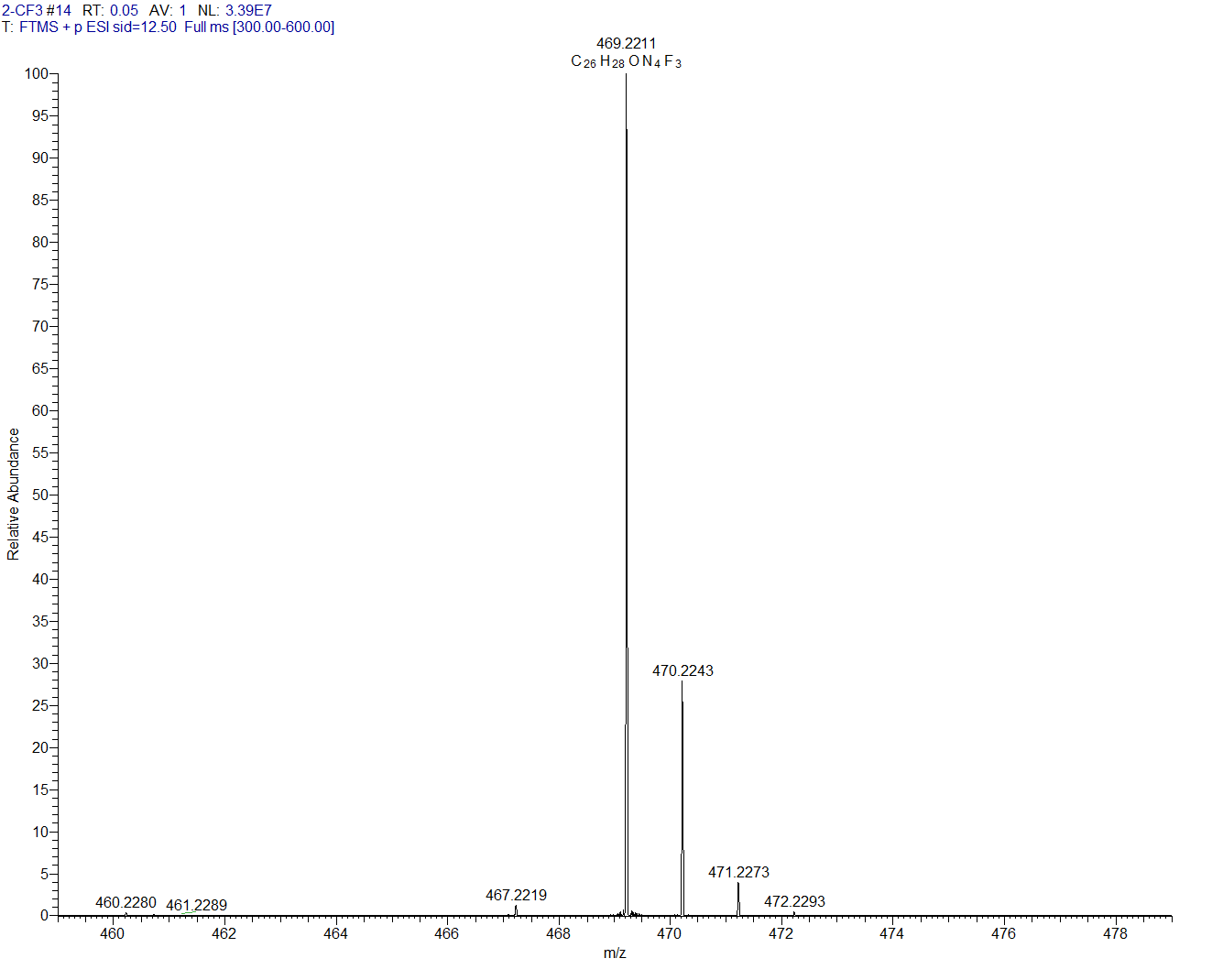
**

**FigureS73. HR**MS spectra of compound **4j**

**
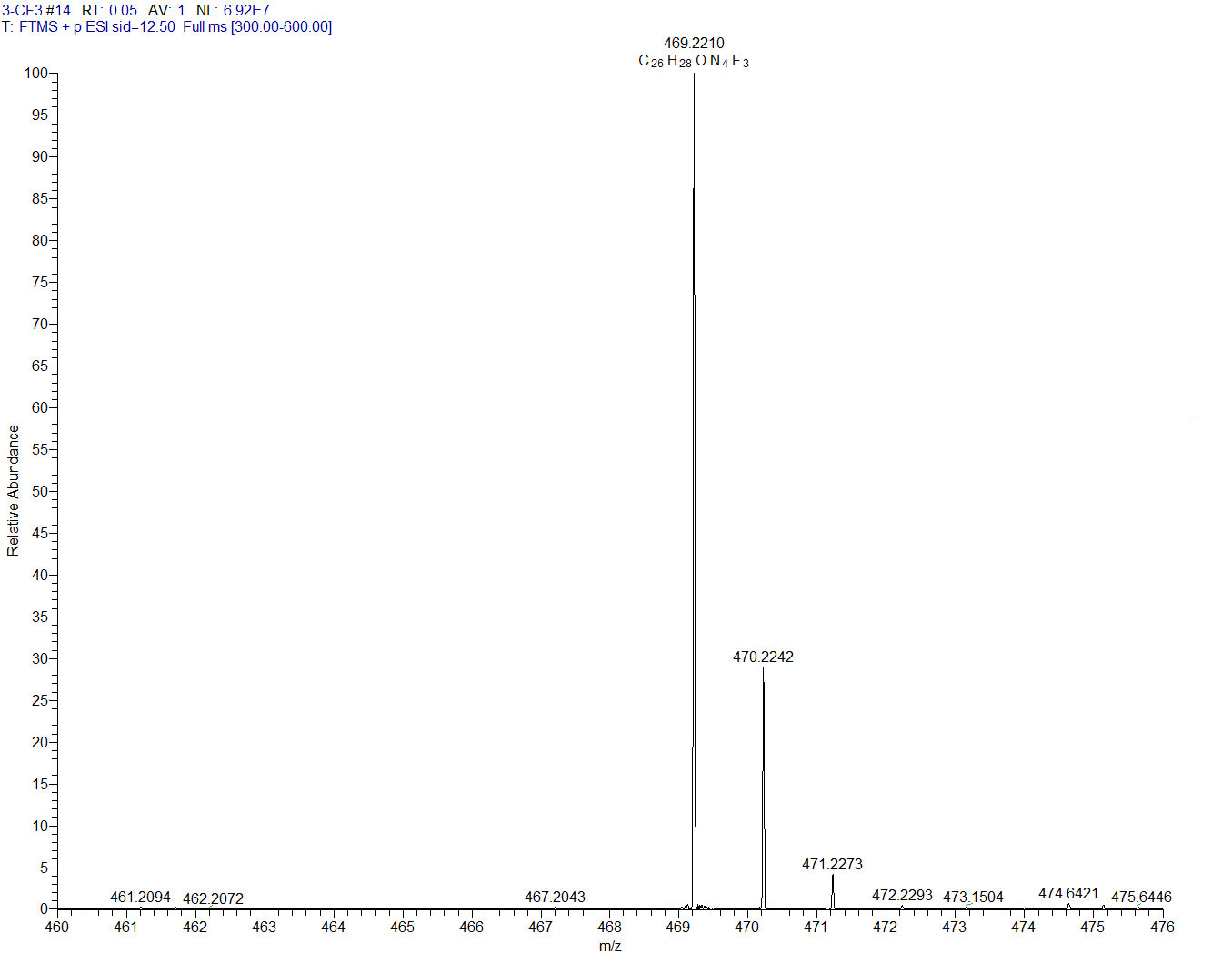
**

**FigureS74. HR**MS spectra of compound **4k**

**
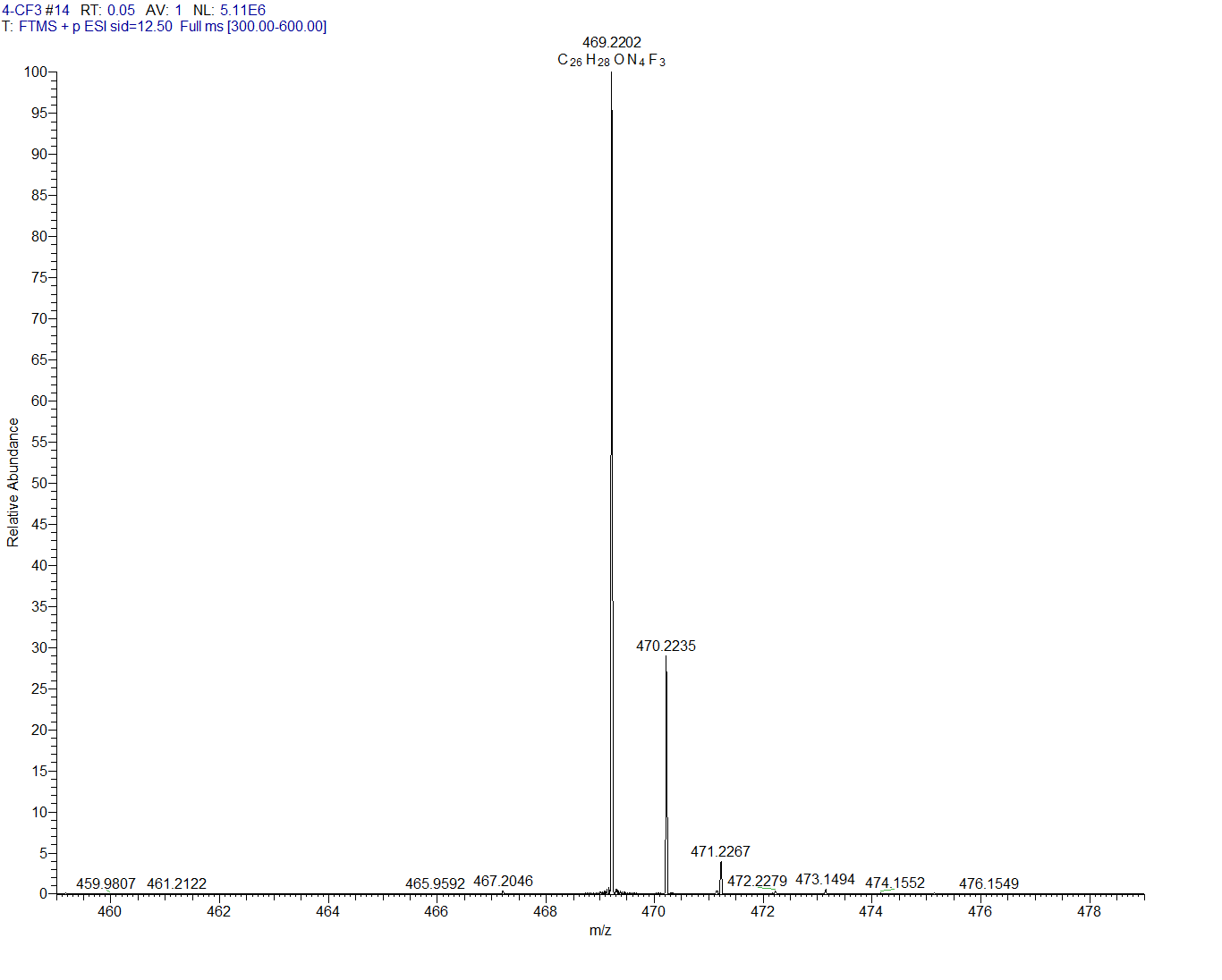
**

**FigureS75. HR**MS spectra of compound **4l**

**
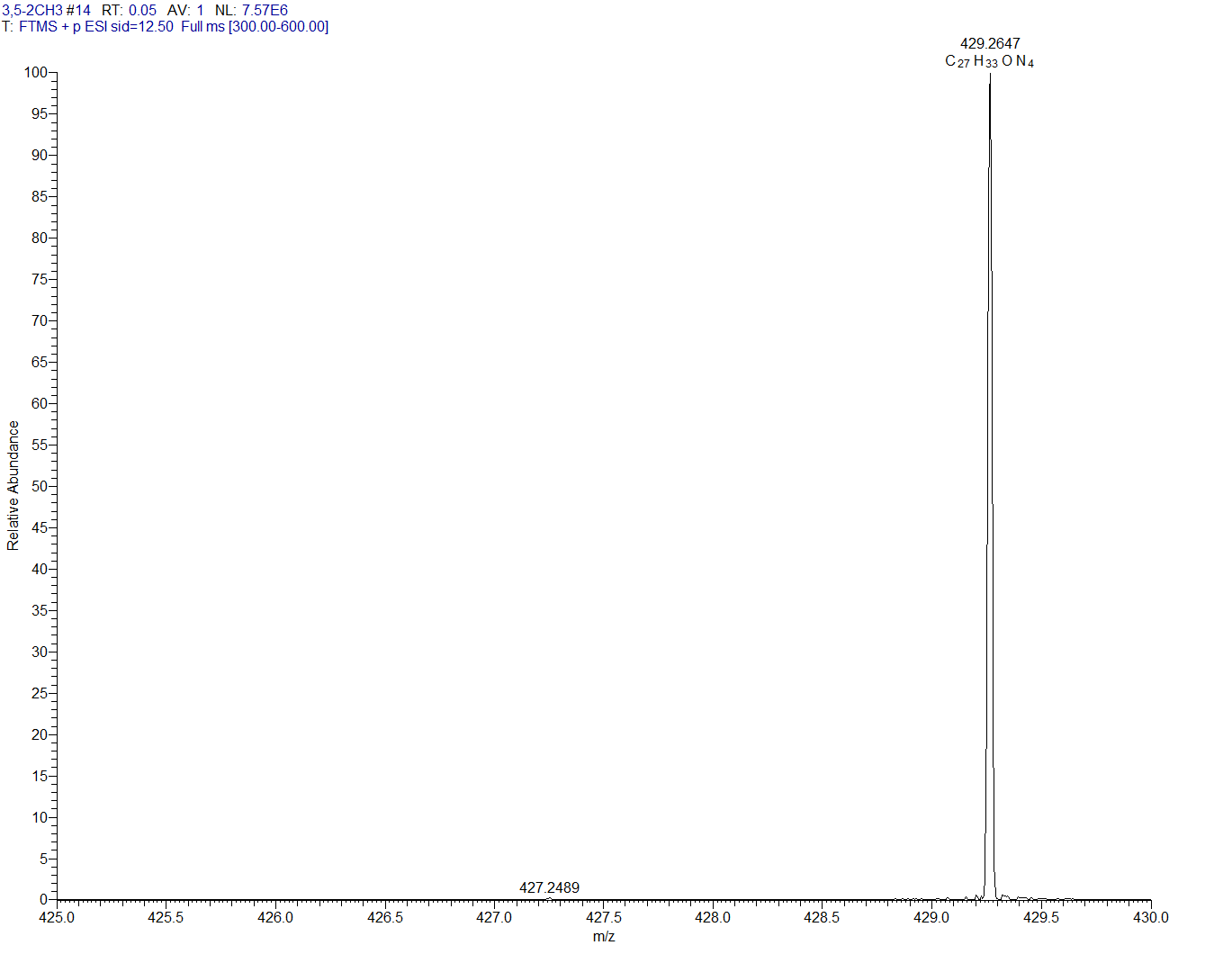
**

**FigureS76. HR**MS spectra of compound **4m**

**
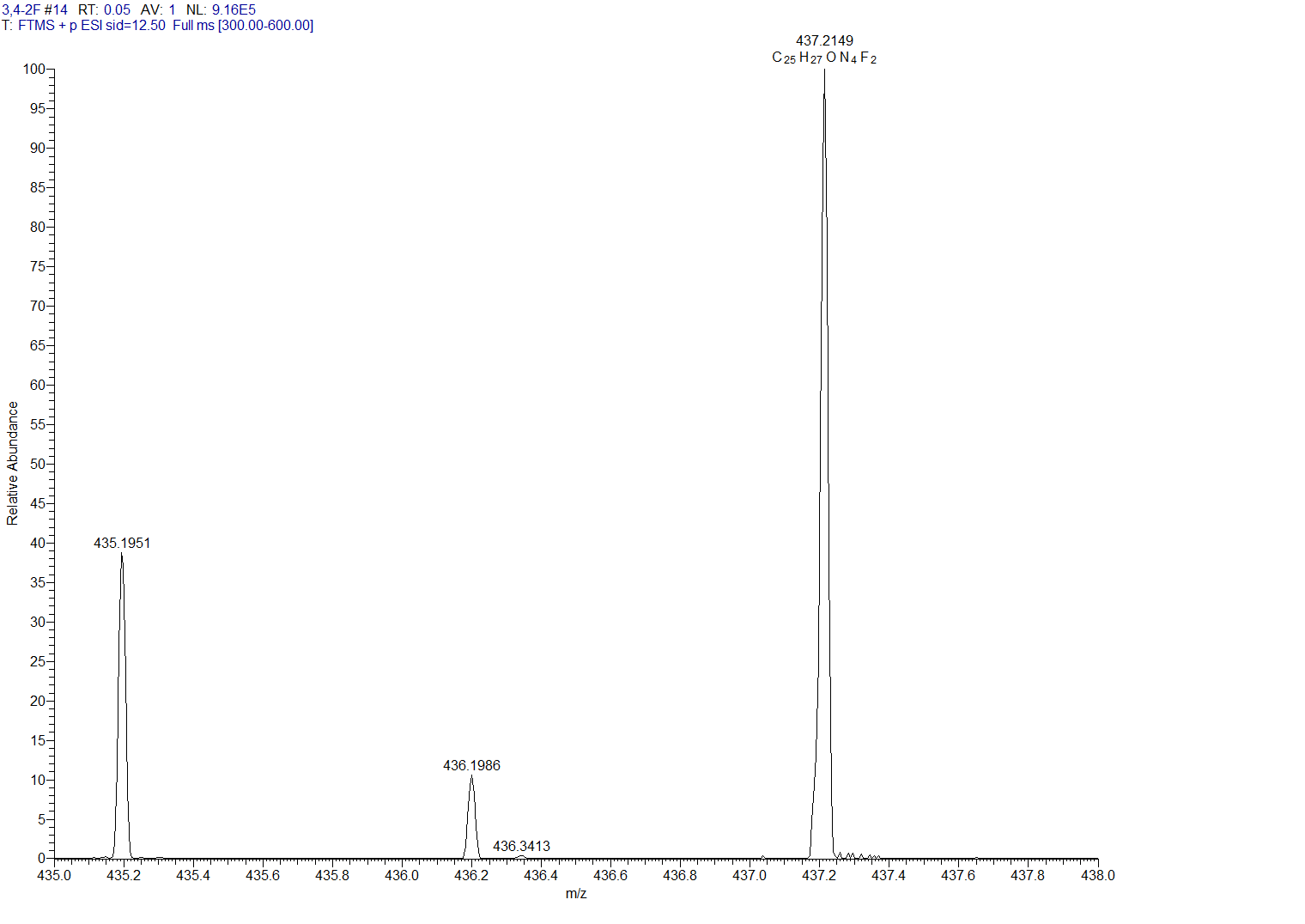
**

**FigureS77. HR**MS spectra of compound **4n**

**
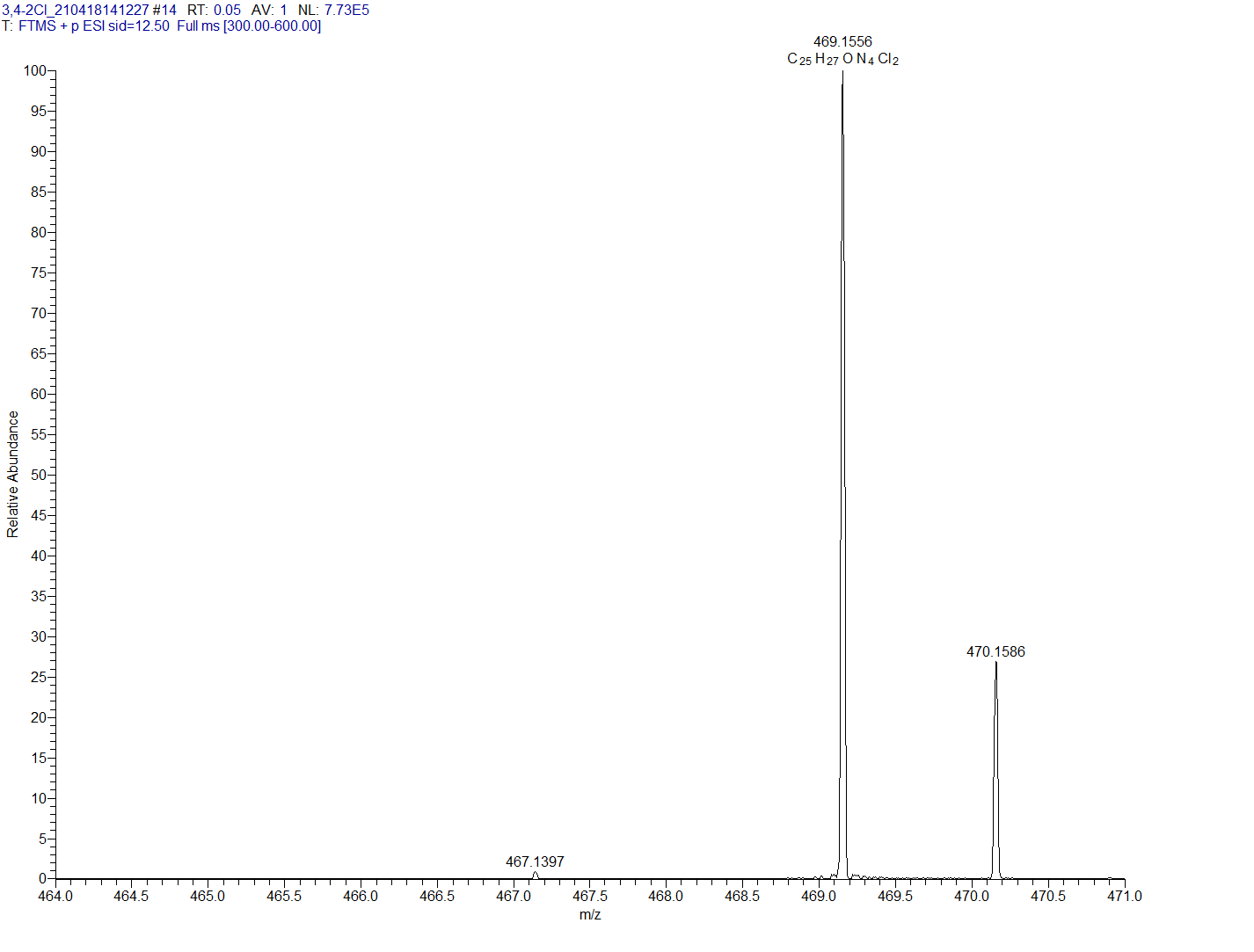
**

**FigureS78. HR**MS spectra of compound **4o**

**
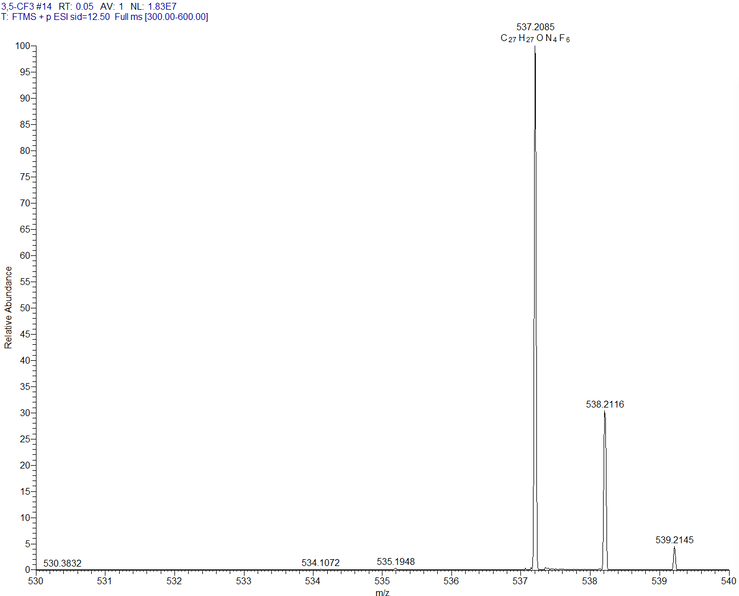
**

**FigureS79. HR**MS spectra of compound **4p**

**
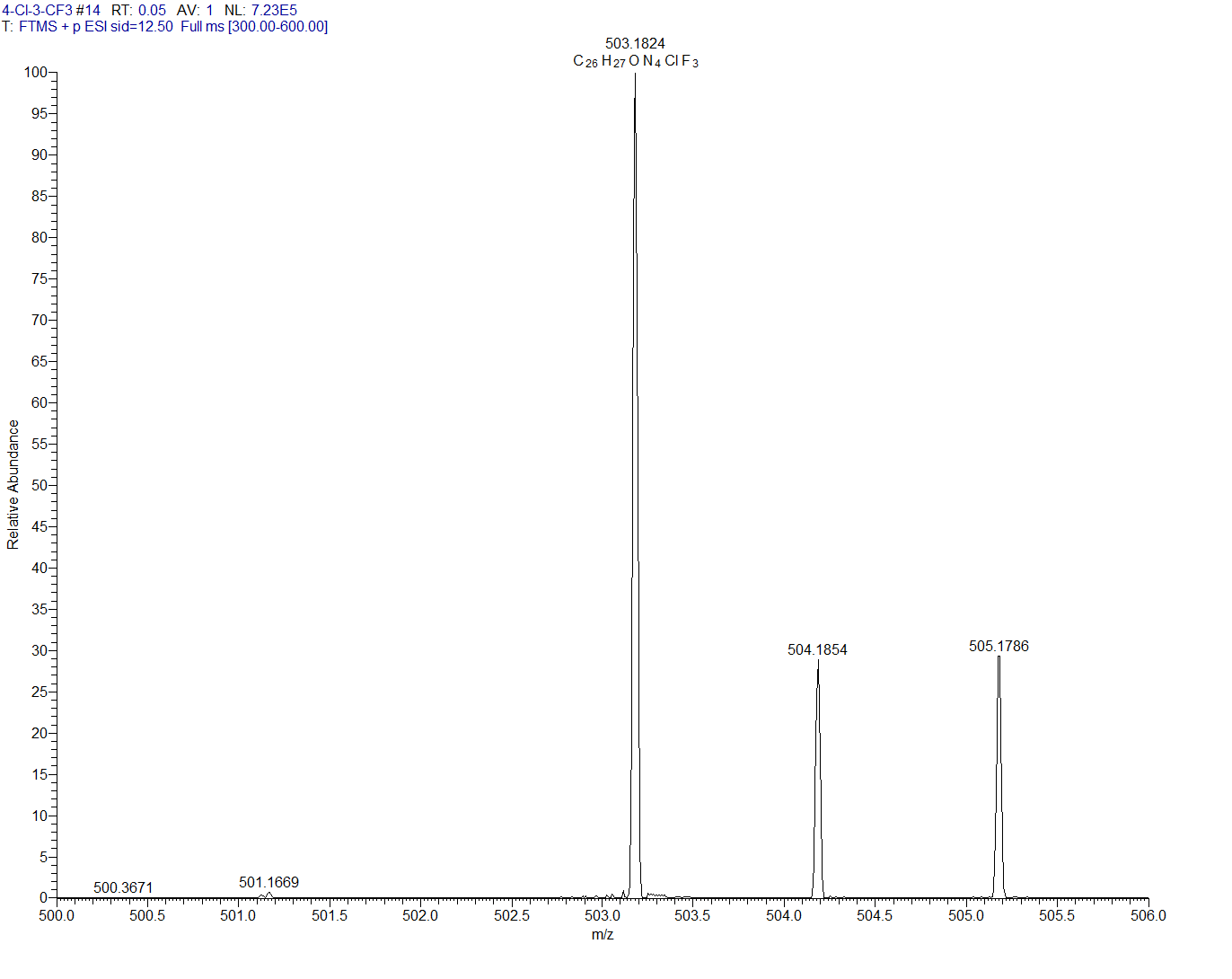
**

**FigureS80. HR**MS spectra of compound **4q**

**
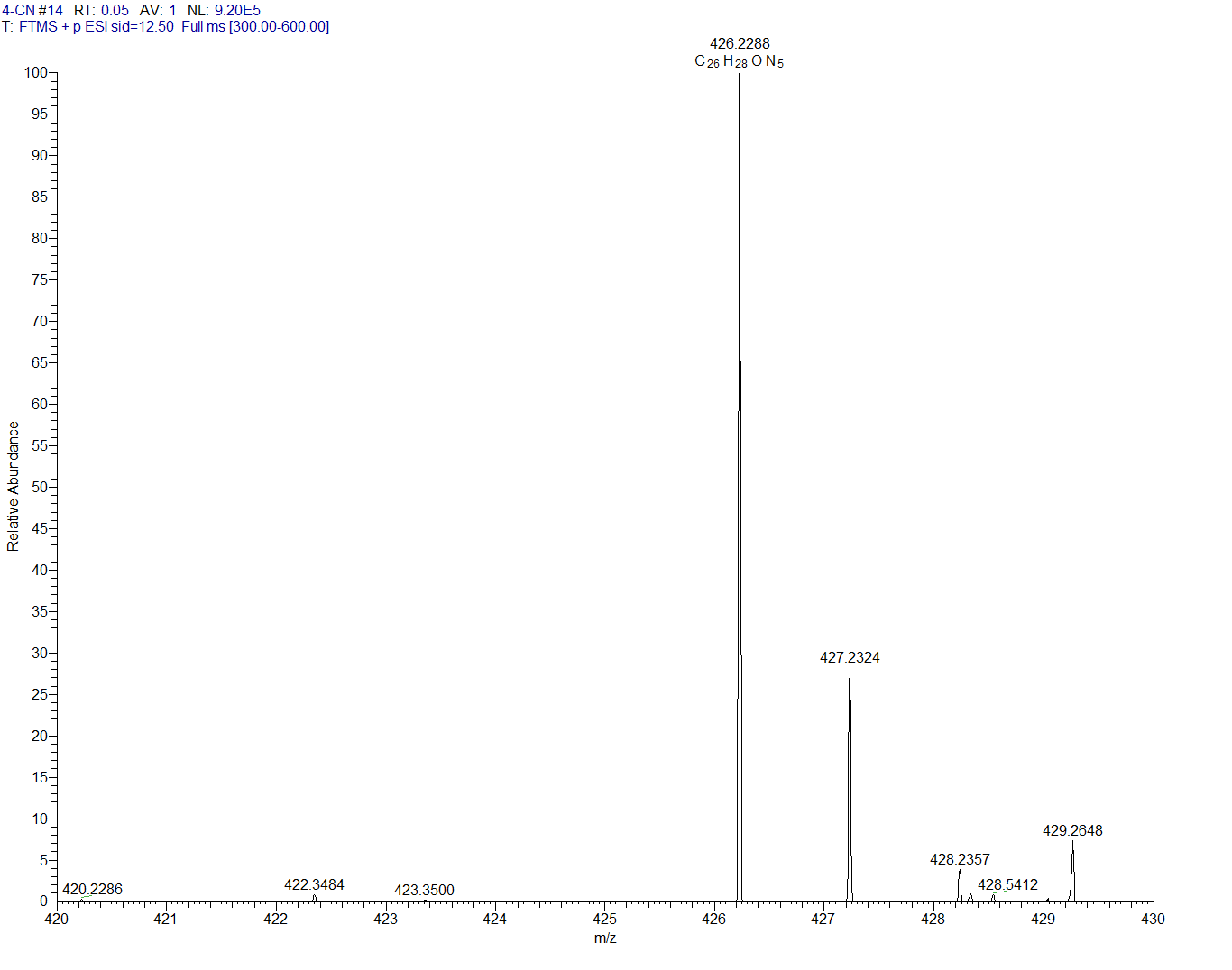
**

**FigureS81. HR**MS spectra of compound **4r**

**
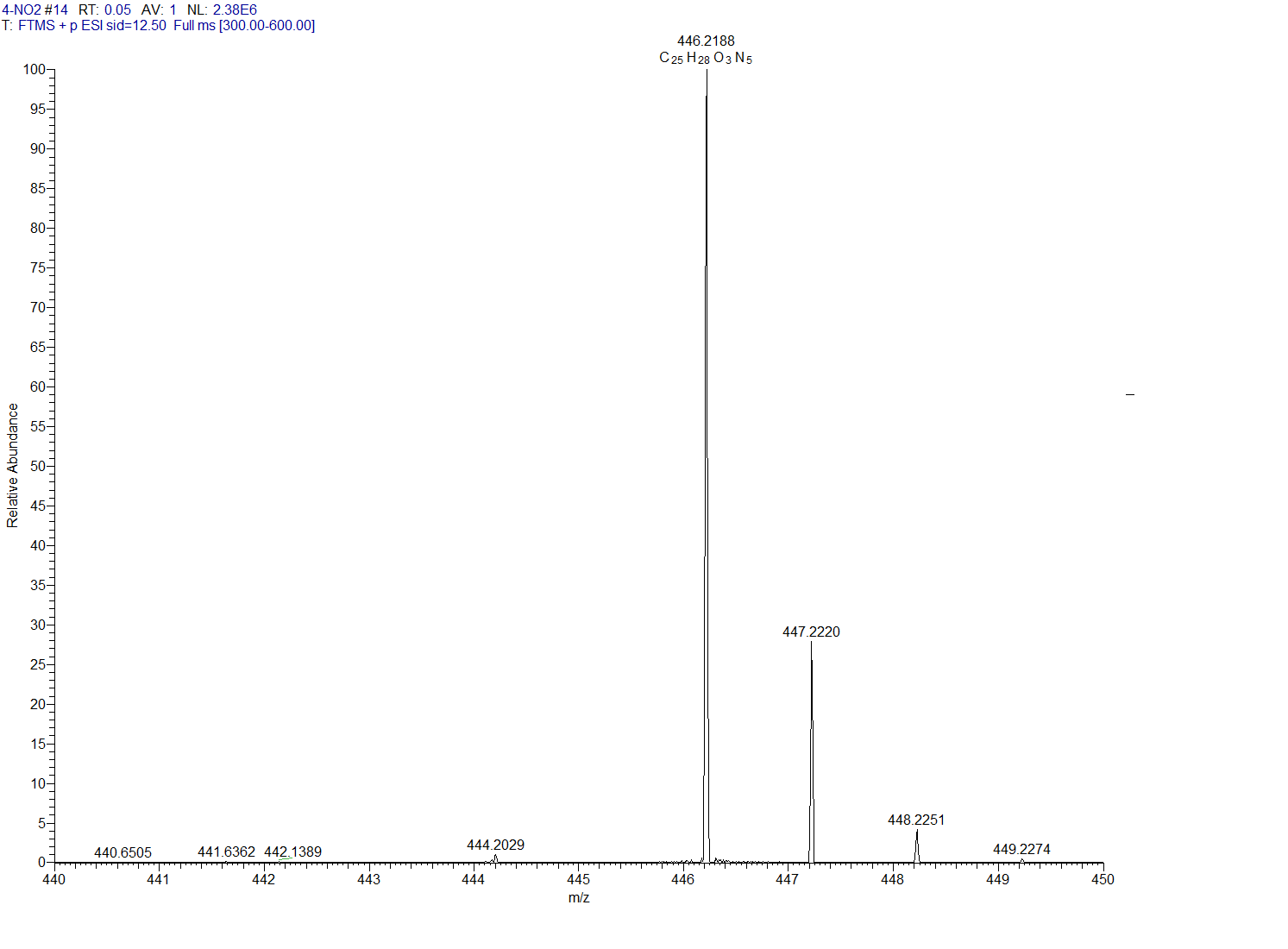
**

**FigureS82. HR**MS spectra of compound **4s**

**Table S1 Predicted targets of 4i**

| **Table S1** Predicted targets of **4i** | | |  |  |  |
| --- | --- | --- | --- | --- | --- |
| No. | Gene Symbol | Protein name |  |  |  |
| 1 | PNMT | phenylethanolamine N-methyltransferase(PNMT) |  |  |  |
| 2 | CDA | cytidine deaminase(CDA) |  |  |  |
| 3 | IGLV2-8 | immunoglobulin lambda variable 2-8(IGLV2-8) |  |  |  |
| 4 | LGALS3 | galectin 3(LGALS3) |  |  |  |
| 5 | STS | steroid sulfatase (microsomal), isozyme S(STS) |  |  |  |
| 6 | AKT2 | AKT serinethreonine kinase 2(AKT2) |  |  |  |
| 7 | CHEK1 | checkpoint kinase 1(CHEK1) |  |  |  |
| 8 | KDR | kinase insert domain receptor(KDR) |  |  |  |
| 9 | PRKACA | protein kinase cAMP-activated catalytic subunit alpha(PRKACA) |  |  |  |
| 10 | LGALS7 | galectin 7(LGALS7) |  |  |  |
| 11 | EPHB4 | EPH receptor B4(EPHB4) |  |  |  |
| 12 | PDK2 | pyruvate dehydrogenase kinase 2(PDK2) |  |  |  |
| 13 | SULT2A1 | sulfotransferase family 2A member 1(SULT2A1) |  |  |  |
| 14 | ARSA | arylsulfatase A(ARSA) |  |  |  |
| 15 | SDS | serine dehydratase(SDS) |  |  |  |
| 16 | DAPK1 | death associated protein kinase 1(DAPK1) |  |  |  |
| 17 | MIF | macrophage migration inhibitory factor (glycosylation-inhibiting factor)(MIF) |  |  |  |
| 18 | RNASE2 | ribonuclease A family member 2(RNASE2) |  |  |  |
| 19 | SULT2B1 | sulfotransferase family 2B member 1(SULT2B1) |  |  |  |
| 20 | CHIT1 | chitinase 1(CHIT1) |  |  |  |
| 21 | AR | androgen receptor(AR) |  |  |  |
| 22 | ACE2 | angiotensin I converting enzyme 2(ACE2) |  |  |  |
| 23 | RBP4 | retinol binding protein 4(RBP4) |  |  |  |
| 24 | MTAP | methylthioadenosine phosphorylase(MTAP) |  |  |  |
| 25 | DDX39B | DExD-box helicase 39B(DDX39B) |  |  |  |
| 26 | MTHFD1 | methylenetetrahydrofolate dehydrogenase, cyclohydrolase and  formyltetrahydrofolate synthetase 1(MTHFD1) | | |  |
| 27 | PRKCQ | protein kinase C theta(PRKCQ) |  |  |  |
| 28 | PADI4 | peptidyl arginine deiminase 4(PADI4) |  |  |  |
| 29 | CFB | complement factor B(CFB) |  |  |  |
| 30 | CES1 | carboxylesterase 1(CES1) |  |  |  |
| 31 | CFD | complement factor D(CFD) |  |  |  |
| 32 | TPH1 | tryptophan hydroxylase 1(TPH1) |  |  |  |
| 33 | CRABP2 | cellular retinoic acid binding protein 2(CRABP2) |  |  |  |
| 34 | MAOB | monoamine oxidase B(MAOB) |  |  |  |
| 35 | SHMT1 | serine hydroxymethyltransferase 1(SHMT1) |  |  |  |
| 36 | HPN | hepsin(HPN) |  |  |  |
| 37 | PIK3R1 | phosphoinositide-3-kinase regulatory subunit 1(PIK3R1) |  |  |  |
| 38 | ADH5 | alcohol dehydrogenase 5 (class III), chi polypeptide(ADH5) |  |  |  |
| 39 | ABO | ABO blood group (transferase A, alpha 1-3-N-acetylgalactosaminyltransferase;  transferase B, alpha 1-3-galactosyltransferase)(ABO) | | | |
| 40 | SEC14L2 | SEC14 like lipid binding 2(SEC14L2) |  |  |  |
| 41 | HSD11B1 | hydroxysteroid 11-beta dehydrogenase 1(HSD11B1) |  |  |  |
| 42 | TTPA | alpha tocopherol transfer protein(TTPA) |  |  |  |
| 43 | HMOX1 | heme oxygenase 1(HMOX1) |  |  |  |
| 44 | LTA4H | leukotriene A4 hydrolase(LTA4H) |  |  |  |
| 45 | PCK1 | phosphoenolpyruvate carboxykinase 1(PCK1) |  |  |  |
| 46 | GSTT2B | glutathione S-transferase theta 2B (genepseudogene)(GSTT2B) |  |  |  |
| 47 | ELANE | elastase, neutrophil expressed(ELANE) |  |  |  |
| 48 | GSTM2 | glutathione S-transferase mu 2(GSTM2) |  |  |  |
| 49 | ACE | angiotensin I converting enzyme(ACE) |  |  |  |
| 50 | GSTM1 | glutathione S-transferase mu 1(GSTM1) |  |  |  |
| 51 | INSR | insulin receptor(INSR) |  |  |  |
| 52 | IGF1 | insulin like growth factor 1(IGF1) |  |  |  |
| 53 | LYZ | lysozyme(LYZ) |  |  |  |
| 54 | DHFR | dihydrofolate reductase(DHFR) |  |  |  |
| 55 | BST1 | bone marrow stromal cell antigen 1(BST1) |  |  |  |
| 56 | CYP2C9 | cytochrome P450 family 2 subfamily C member 9(CYP2C9) |  |  |  |
| 57 | BHMT | betaine--homocysteine S-methyltransferase(BHMT) |  |  |  |
| 58 | UCK2 | uridine-cytidine kinase 2(UCK2) |  |  |  |
| 59 | CYP2C8 | cytochrome P450 family 2 subfamily C member 8(CYP2C8) |  |  |  |
| 60 | PPP5C | protein phosphatase 5 catalytic subunit(PPP5C) |  |  |  |
| 61 | PAH | phenylalanine hydroxylase(PAH) |  |  |  |
| 62 | KYAT1 | kynurenine aminotransferase 1(KYAT1) |  |  |  |
| 63 | REN | renin(REN) |  |  |  |
| 64 | GRB2 | growth factor receptor bound protein 2(GRB2) |  |  |  |
| 65 | ITK | IL2 inducible T-cell kinase(ITK) |  |  |  |
| 66 | ARF4 | ADP ribosylation factor 4(ARF4) |  |  |  |
| 67 | GPI | glucose-6-phosphate isomerase(GPI) |  |  |  |
| 68 | RARG | retinoic acid receptor gamma(RARG) |  |  |  |
| 69 | ARF1 | ADP ribosylation factor 1(ARF1) |  |  |  |
| 70 | SERPINA1 | serpin family A member 1(SERPINA1) |  |  |  |
| 71 | HEXB | hexosaminidase subunit beta(HEXB) |  |  |  |
| 72 | SETD7 | SET domain containing lysine methyltransferase 7(SETD7) |  |  |  |
| 73 | HNF4G | hepatocyte nuclear factor 4 gamma(HNF4G) |  |  |  |
| 74 | ADK | adenosine kinase(ADK) |  |  |  |
| 75 | AKR1B1 | aldo-keto reductase family 1 member B(AKR1B1) |  |  |  |
| 76 | PYGL | phosphorylase, glycogen, liver(PYGL) |  |  |  |
| 77 | KIF11 | kinesin family member 11(KIF11) |  |  |  |
| 78 | CTSS | cathepsin S(CTSS) |  |  |  |
| 79 | EEA1 | early endosome antigen 1(EEA1) |  |  |  |
| 80 | ATIC | aminoimidazole-4-carboxamide ribonucleotide formyltransferaseIMP  cyclohydrolase(ATIC) |  |  |  |
| 81 | C8G | complement C8 gamma chain(C8G) |  |  |  |
| 82 | RFK | riboflavin kinase(RFK) |  |  |  |
| 83 | CTSK | cathepsin K(CTSK) |  |  |  |
| 84 | CTSG | cathepsin G(CTSG) |  |  |  |
| 85 | RAC1 | ras-related C3 botulinum toxin substrate 1 (rho family, small GTP binding protein  Rac1)(RAC1) |  |  |  |
| 86 | ACADM | acyl-CoA dehydrogenase, C-4 to C-12 straight chain(ACADM) |  |  |  |
| 87 | CTSF | cathepsin F(CTSF) |  |  |  |
| 88 | JAK2 | Janus kinase 2(JAK2) |  |  |  |
| 89 | JAK3 | Janus kinase 3(JAK3) |  |  |  |
| 90 | HRAS | HRas proto-oncogene, GTPase(HRAS) |  |  |  |
| 91 | CTSB | cathepsin B(CTSB) |  |  |  |
| 92 | TPI1 | triosephosphate isomerase 1(TPI1) |  |  |  |
| 93 | SYK | spleen associated tyrosine kinase(SYK) |  |  |  |
| 94 | MME | membrane metalloendopeptidase(MME) |  |  |  |
| 95 | PARP1 | poly(ADP-ribose) polymerase 1(PARP1) |  |  |  |
| 96 | ARG1 | arginase 1(ARG1) |  |  |  |
| 97 | ANXA5 | annexin A5(ANXA5) |  |  |  |
| 98 | NME2 | NMENM23 nucleoside diphosphate kinase 2(NME2) |  |  |  |
| 99 | F2 | coagulation factor II, thrombin(F2) |  |  |  |
| 100 | DUSP6 | dual specificity phosphatase 6(DUSP6) |  |  |  |
| 101 | MMP12 | matrix metallopeptidase 12(MMP12) |  |  |  |
| 102 | BACE1 | beta-secretase 1(BACE1) |  |  |  |
| 103 | F7 | coagulation factor VII(F7) |  |  |  |
| 104 | KAT2B | lysine acetyltransferase 2B(KAT2B) |  |  |  |
| 105 | HCK | HCK proto-oncogene, Src family tyrosine kinase(HCK) |  |  |  |
| 106 | MMP13 | matrix metallopeptidase 13(MMP13) |  |  |  |
| 107 | KIT | KIT proto-oncogene receptor tyrosine kinase(KIT) |  |  |  |
| 108 | RARA | retinoic acid receptor alpha(RARA) |  |  |  |
| 109 | RARB | retinoic acid receptor beta(RARB) |  |  |  |
| 110 | PPARG | peroxisome proliferator activated receptor gamma(PPARG) |  |  |  |
| 111 | PPARA | peroxisome proliferator activated receptor alpha(PPARA) |  |  |  |
| 112 | GART | phosphoribosylglycinamide formyltransferase, phosphoribosylglycinamide  synthetase, phosphoribosylaminoimidazole synthetase(GART) | | | |
| 113 | PPARD | peroxisome proliferator activated receptor delta(PPARD) |  |  |  |
| 114 | APCS | amyloid P component, serum(APCS) |  |  |  |
| 115 | DTYMK | deoxythymidylate kinase(DTYMK) |  |  |  |
| 116 | OAT | ornithine aminotransferase(OAT) |  |  |  |
| 117 | ADH1C | alcohol dehydrogenase 1C (class I), gamma polypeptide(ADH1C) |  |  |  |
| 118 | NR1I3 | nuclear receptor subfamily 1 group I member 3(NR1I3) |  |  |  |
| 119 | GSTP1 | glutathione S-transferase pi 1(GSTP1) |  |  |  |
| 120 | NR1I2 | nuclear receptor subfamily 1 group I member 2(NR1I2) |  |  |  |
| 121 | WAS | Wiskott-Aldrich syndrome(WAS) |  |  |  |
| 122 | XIAP | X-linked inhibitor of apoptosis(XIAP) |  |  |  |
| 123 | TYMS | thymidylate synthetase(TYMS) |  |  |  |
| 124 | EGFR | epidermal growth factor receptor(EGFR) |  |  |  |
| 125 | PAPSS1 | 3'-phosphoadenosine 5'-phosphosulfate synthase 1(PAPSS1) |  |  |  |
| 126 | RXRB | retinoid X receptor beta(RXRB) |  |  |  |
| 127 | RIDA | reactive intermediate imine deaminase A homolog(RIDA) |  |  |  |
| 128 | RXRA | retinoid X receptor alpha(RXRA) |  |  |  |
| 129 | SULT1E1 | sulfotransferase family 1E member 1(SULT1E1) |  |  |  |
| 130 | HSD17B1 | hydroxysteroid 17-beta dehydrogenase 1(HSD17B1) |  |  |  |
| 131 | CTNNA1 | catenin alpha 1(CTNNA1) |  |  |  |
| 132 | NT5M | 5',3'-nucleotidase, mitochondrial(NT5M) |  |  |  |
| 133 | GC | GC, vitamin D binding protein(GC) |  |  |  |
| 134 | LGALS7B | galectin 7B(LGALS7B) |  |  |  |
| 135 | BCHE | butyrylcholinesterase(BCHE) |  |  |  |
| 136 | NQO1 | NAD(P)H quinone dehydrogenase 1(NQO1) |  |  |  |
| 137 | NQO2 | NAD(P)H quinone dehydrogenase 2(NQO2) |  |  |  |
| 138 | F10 | coagulation factor X(F10) |  |  |  |
| 139 | PKLR | pyruvate kinase, liver and RBC(PKLR) |  |  |  |
| 140 | STAT1 | signal transducer and activator of transcription 1(STAT1) |  |  |  |
| 141 | NR1H2 | nuclear receptor subfamily 1 group H member 2(NR1H2) |  |  |  |
| 142 | AKR1C1 | aldo-keto reductase family 1 member C1(AKR1C1) |  |  |  |
| 143 | F11 | coagulation factor XI(F11) |  |  |  |
| 144 | GSR | glutathione-disulfide reductase(GSR) |  |  |  |
| 145 | NR1H4 | nuclear receptor subfamily 1 group H member 4(NR1H4) |  |  |  |
| 146 | AKR1C3 | aldo-keto reductase family 1 member C3(AKR1C3) |  |  |  |
| 147 | ADAM33 | ADAM metallopeptidase domain 33(ADAM33) |  |  |  |
| 148 | NR1H3 | nuclear receptor subfamily 1 group H member 3(NR1H3) |  |  |  |
| 149 | AKR1C2 | aldo-keto reductase family 1 member C2(AKR1C2) |  |  |  |
| 150 | NMNAT3 | nicotinamide nucleotide adenylyltransferase 3(NMNAT3) |  |  |  |
| 151 | GCK | glucokinase(GCK) |  |  |  |
| 152 | FKBP1A | FK506 binding protein 1A(FKBP1A) |  |  |  |
| 153 | FABP3 | fatty acid binding protein 3(FABP3) |  |  |  |
| 154 | FABP4 | fatty acid binding protein 4(FABP4) |  |  |  |
| 155 | NMNAT1 | nicotinamide nucleotide adenylyltransferase 1(NMNAT1) |  |  |  |
| 156 | FABP5 | fatty acid binding protein 5(FABP5) |  |  |  |
| 157 | GSTA3 | glutathione S-transferase alpha 3(GSTA3) |  |  |  |
| 158 | FABP6 | fatty acid binding protein 6(FABP6) |  |  |  |
| 159 | FABP7 | fatty acid binding protein 7(FABP7) |  |  |  |
| 160 | FKBP1B | FK506 binding protein 1B(FKBP1B) |  |  |  |
| 161 | GSTA1 | glutathione S-transferase alpha 1(GSTA1) |  |  |  |
| 162 | OTC | ornithine carbamoyltransferase(OTC) |  |  |  |
| 163 | CDK5R1 | cyclin dependent kinase 5 regulatory subunit 1(CDK5R1) |  |  |  |
| 164 | TPSB2 | tryptase beta 2 (genepseudogene)(TPSB2) |  |  |  |
| 165 | AMY2A | amylase, alpha 2A (pancreatic)(AMY2A) |  |  |  |
| 166 | CCNT1 | cyclin T1(CCNT1) |  |  |  |
| 167 | RORA | RAR related orphan receptor A(RORA) |  |  |  |
| 168 | PLAT | plasminogen activator, tissue type(PLAT) |  |  |  |
| 169 | HNMT | histamine N-methyltransferase(HNMT) |  |  |  |
| 170 | NR3C1 | nuclear receptor subfamily 3 group C member 1(NR3C1) |  |  |  |
| 171 | IGF1R | insulin like growth factor 1 receptor(IGF1R) |  |  |  |
| 172 | NR3C2 | nuclear receptor subfamily 3 group C member 2(NR3C2) |  |  |  |
| 173 | PPP1CC | protein phosphatase 1 catalytic subunit gamma(PPP1CC) |  |  |  |
| 174 | FNTA | farnesyltransferase, CAAX box, alpha(FNTA) |  |  |  |
| 175 | PLAU | plasminogen activator, urokinase(PLAU) |  |  |  |
| 176 | PIM1 | Pim-1 proto-oncogene, serinethreonine kinase(PIM1) |  |  |  |
| 177 | HADH | hydroxyacyl-CoA dehydrogenase(HADH) |  |  |  |
| 178 | TGM3 | transglutaminase 3(TGM3) |  |  |  |
| 179 | MAP2K1 | mitogen-activated protein kinase kinase 1(MAP2K1) |  |  |  |
| 180 | AMY1A | amylase, alpha 1A (salivary)(AMY1A) |  |  |  |
| 181 | AMY1B | amylase, alpha 1B (salivary)(AMY1B) |  |  |  |
| 182 | AMY1C | amylase, alpha 1C (salivary)(AMY1C) |  |  |  |
| 183 | TAP1 | transporter 1, ATP binding cassette subfamily B member(TAP1) |  |  |  |
| 184 | PGF | placental growth factor(PGF) |  |  |  |
| 185 | PROCR | protein C receptor(PROCR) |  |  |  |
| 186 | ADAM17 | ADAM metallopeptidase domain 17(ADAM17) |  |  |  |
| 187 | FOLH1 | folate hydrolase 1(FOLH1) |  |  |  |
| 188 | PLA2G10 | phospholipase A2 group X(PLA2G10) |  |  |  |
| 189 | MAPKAPK2 | mitogen-activated protein kinase-activated protein kinase 2(MAPKAPK2) |  |  |  |
| 190 | DPEP1 | dipeptidase 1 (renal)(DPEP1) |  |  |  |
| 191 | PGR | progesterone receptor(PGR) |  |  |  |
| 192 | FKBP3 | FK506 binding protein 3(FKBP3) |  |  |  |
| 193 | PPIA | peptidylprolyl isomerase A(PPIA) |  |  |  |
| 194 | S100A9 | S100 calcium binding protein A9(S100A9) |  |  |  |
| 195 | PFKFB1 | 6-phosphofructo-2-kinasefructose-2,6-biphosphatase 1(PFKFB1) |  |  |  |
| 196 | FECH | ferrochelatase(FECH) |  |  |  |
| 197 | GLO1 | glyoxalase I(GLO1) |  |  |  |
| 198 | CYP19A1 | cytochrome P450 family 19 subfamily A member 1(CYP19A1) |  |  |  |
| 199 | DPP4 | dipeptidyl peptidase 4(DPP4) |  |  |  |
| 200 | PSAP | prosaposin(PSAP) |  |  |  |
| 201 | ABL1 | ABL proto-oncogene 1, non-receptor tyrosine kinase(ABL1) |  |  |  |
| 202 | ESRRA | estrogen related receptor alpha(ESRRA) |  |  |  |
| 203 | TRAPPC3 | trafficking protein particle complex 3(TRAPPC3) |  |  |  |
| 204 | HSPA8 | heat shock protein family A (Hsp70) member 8(HSPA8) |  |  |  |
| 205 | FDPS | farnesyl diphosphate synthase(FDPS) |  |  |  |
| 206 | TGFB2 | transforming growth factor beta 2(TGFB2) |  |  |  |
| 207 | VDR | vitamin D (1,25- dihydroxyvitamin D3) receptor(VDR) |  |  |  |
| 208 | EPHX2 | epoxide hydrolase 2(EPHX2) |  |  |  |
| 209 | ESRRG | estrogen related receptor gamma(ESRRG) |  |  |  |
| 210 | ESR1 | estrogen receptor 1(ESR1) |  |  |  |
| 211 | IL2 | interleukin 2(IL2) |  |  |  |
| 212 | ESR2 | estrogen receptor 2(ESR2) |  |  |  |
| 213 | CDK7 | cyclin dependent kinase 7(CDK7) |  |  |  |
| 214 | BMP2 | bone morphogenetic protein 2(BMP2) |  |  |  |
| 215 | CDK2 | cyclin dependent kinase 2(CDK2) |  |  |  |
| 216 | ALB | albumin(ALB) |  |  |  |
| 217 | MDM2 | MDM2 proto-oncogene(MDM2) |  |  |  |
| 218 | LCN2 | lipocalin 2(LCN2) |  |  |  |
| 219 | SHBG | sex hormone binding globulin(SHBG) |  |  |  |
| 220 | FGFR2 | fibroblast growth factor receptor 2(FGFR2) |  |  |  |
| 221 | BCL2L1 | BCL2 like 1(BCL2L1) |  |  |  |
| 222 | FGFR1 | fibroblast growth factor receptor 1(FGFR1) |  |  |  |
| 223 | GSK3B | glycogen synthase kinase 3 beta(GSK3B) |  |  |  |
| 224 | THRB | thyroid hormone receptor beta(THRB) |  |  |  |
| 225 | HSP90AB1 | heat shock protein 90 alpha family class B member 1(HSP90AB1) |  |  |  |
| 226 | THRA | thyroid hormone receptor, alpha(THRA) |  |  |  |
| 227 | AMD1 | adenosylmethionine decarboxylase 1(AMD1) |  |  |  |
| 228 | PDE3B | phosphodiesterase 3B(PDE3B) |  |  |  |
| 229 | PCTP | phosphatidylcholine transfer protein(PCTP) |  |  |  |
| 230 | ITGAL | integrin subunit alpha L(ITGAL) |  |  |  |
| 231 | RND3 | Rho family GTPase 3(RND3) |  |  |  |
| 232 | PIK3CG | phosphatidylinositol-4,5-bisphosphate 3-kinase catalytic subunit gamma(PIK3CG) |  |  |  |
| 233 | ADAMTS4 | ADAM metallopeptidase with thrombospondin type 1 motif 4(ADAMTS4) |  |  |  |
| 234 | ALAD | aminolevulinate dehydratase(ALAD) |  |  |  |
| 235 | CASP7 | caspase 7(CASP7) |  |  |  |
| 236 | GM2A | GM2 ganglioside activator(GM2A) |  |  |  |
| 237 | CA2 | carbonic anhydrase 2(CA2) |  |  |  |
| 238 | CASP3 | caspase 3(CASP3) |  |  |  |
| 239 | CASP1 | caspase 1(CASP1) |  |  |  |
| 240 | PDE4B | phosphodiesterase 4B(PDE4B) |  |  |  |
| 241 | ME2 | malic enzyme 2(ME2) |  |  |  |
| 242 | ARL5A | ADP ribosylation factor like GTPase 5A(ARL5A) |  |  |  |
| 243 | HSP90AA1 | heat shock protein 90 alpha family class A member 1(HSP90AA1) |  |  |  |
| 244 | APAF1 | apoptotic peptidase activating factor 1(APAF1) |  |  |  |
| 245 | PDPK1 | 3-phosphoinositide dependent protein kinase 1(PDPK1) |  |  |  |
| 246 | MMP2 | matrix metallopeptidase 2(MMP2) |  |  |  |
| 247 | PDE4D | phosphodiesterase 4D(PDE4D) |  |  |  |
| 248 | FGG | fibrinogen gamma chain(FGG) |  |  |  |
| 249 | MMP3 | matrix metallopeptidase 3(MMP3) |  |  |  |
| 250 | APOA2 | apolipoprotein A2(APOA2) |  |  |  |
| 251 | SORD | sorbitol dehydrogenase(SORD) |  |  |  |
| 252 | MMP8 | matrix metallopeptidase 8(MMP8) |  |  |  |
| 253 | MMP9 | matrix metallopeptidase 9(MMP9) |  |  |  |
| 254 | DCK | deoxycytidine kinase(DCK) |  |  |  |
| 255 | TGFBR1 | transforming growth factor beta receptor 1(TGFBR1) |  |  |  |
| 256 | CCNA2 | cyclin A2(CCNA2) |  |  |  |
| 257 | ZAP70 | zeta chain of T cell receptor associated protein kinase 70(ZAP70) |  |  |  |
| 258 | LCK | LCK proto-oncogene, Src family tyrosine kinase(LCK) |  |  |  |
| 259 | BLVRB | biliverdin reductase B(BLVRB) |  |  |  |
| 260 | ANG | angiogenin(ANG) |  |  |  |
| 261 | PDE5A | phosphodiesterase 5A(PDE5A) |  |  |  |
| 262 | BPI | bactericidalpermeability-increasing protein(BPI) |  |  |  |
| 263 | BIRC7 | baculoviral IAP repeat containing 7(BIRC7) |  |  |  |
| 264 | AGXT | alanine-glyoxylate aminotransferase(AGXT) |  |  |  |
| 265 | MET | MET proto-oncogene, receptor tyrosine kinase(MET) |  |  |  |
| 266 | BCAT2 | branched chain amino acid transaminase 2(BCAT2) |  |  |  |
| 267 | SRC | SRC proto-oncogene, non-receptor tyrosine kinase(SRC) |  |  |  |
| 268 | HMGCR | 3-hydroxy-3-methylglutaryl-CoA reductase(HMGCR) |  |  |  |
| 269 | HDAC8 | histone deacetylase 8(HDAC8) |  |  |  |
| 270 | AURKA | aurora kinase A(AURKA) |  |  |  |
| 271 | MAPK8 | mitogen-activated protein kinase 8(MAPK8) |  |  |  |
| 272 | TTR | transthyretin(TTR) |  |  |  |
| 273 | CBS | cystathionine-beta-synthase(CBS) |  |  |  |
| 274 | ERBB4 | erb-b2 receptor tyrosine kinase 4(ERBB4) |  |  |  |
| 275 | MAPK1 | mitogen-activated protein kinase 1(MAPK1) |  |  |  |
| 276 | CSK | c-src tyrosine kinase(CSK) |  |  |  |
| 277 | EIF4E | eukaryotic translation initiation factor 4E(EIF4E) |  |  |  |
| 278 | TRDMT1 | tRNA aspartic acid methyltransferase 1(TRDMT1) |  |  |  |
| 279 | PTPN1 | protein tyrosine phosphatase, non-receptor type 1(PTPN1) |  |  |  |
| 280 | DUT | deoxyuridine triphosphatase(DUT) |  |  |  |
| 281 | NOS3 | nitric oxide synthase 3(NOS3) |  |  |  |
| 282 | CMA1 | chymase 1(CMA1) |  |  |  |
| 283 | PLA2G2A | phospholipase A2 group IIA(PLA2G2A) |  |  |  |
| 284 | PTPN11 | protein tyrosine phosphatase, non-receptor type 11(PTPN11) |  |  |  |
| 285 | SOD2 | superoxide dismutase 2, mitochondrial(SOD2) |  |  |  |
| 286 | MAPK14 | mitogen-activated protein kinase 14(MAPK14) |  |  |  |
| 287 | DHODH | dihydroorotate dehydrogenase (quinone)(DHODH) |  |  |  |
| 288 | MAPK10 | mitogen-activated protein kinase 10(MAPK10) |  |  |  |
| 289 | IMPDH1 | inosine monophosphate dehydrogenase 1(IMPDH1) |  |  |  |
| 290 | TEK | TEK receptor tyrosine kinase(TEK) |  |  |  |
| 291 | YARS1 | Tyrosine--tRNA ligase, cytoplasmic (YARS1) |  |  |  |
| 292 | ACP3 | Prostatic acid phosphatase (ACP3) |  |  |  |
| 293 | WARS1 | Tryptophan--tRNA ligase, cytoplasmic (WARS1) |  |  |  |

**Table S2 Targets associated with Hela cell line from GeneCards database**

| **Table S2** Targets associated with Hela cell line from GeneCards database | | | | | | |
| --- | --- | --- | --- | --- | --- | --- |
| No. | Gene Symbol |  |  |  |  |  |
| 1 | TP53 |  |  |  |  |  |
| 2 | BRCA2 |  |  |  |  |  |
| 3 | BRCA1 |  |  |  |  |  |
| 4 | PTEN |  |  |  |  |  |
| 5 | EGFR |  |  |  |  |  |
| 6 | CDH1 |  |  |  |  |  |
| 7 | ATM |  |  |  |  |  |
| 8 | CDKN2A |  |  |  |  |  |
| 9 | KRAS |  |  |  |  |  |
| 10 | PIK3CA |  |  |  |  |  |
| 11 | MET |  |  |  |  |  |
| 12 | ERBB2 |  |  |  |  |  |
| 13 | AKT1 |  |  |  |  |  |
| 14 | MIR21 |  |  |  |  |  |
| 15 | MSH2 |  |  |  |  |  |
| 16 | BRAF |  |  |  |  |  |
| 17 | APC |  |  |  |  |  |
| 18 | CTNNB1 |  |  |  |  |  |
| 19 | CHEK2 |  |  |  |  |  |
| 20 | MSH6 |  |  |  |  |  |
| 21 | MLH1 |  |  |  |  |  |
| 22 | FGFR3 |  |  |  |  |  |
| 23 | PALB2 |  |  |  |  |  |
| 24 | RB1 |  |  |  |  |  |
| 25 | CCND1 |  |  |  |  |  |
| 26 | CASP8 |  |  |  |  |  |
| 27 | TNF |  |  |  |  |  |
| 28 | KIT |  |  |  |  |  |
| 29 | HRAS |  |  |  |  |  |
| 30 | IL6 |  |  |  |  |  |
| 31 | NBN |  |  |  |  |  |
| 32 | STK11 |  |  |  |  |  |
| 33 | MYC |  |  |  |  |  |
| 34 | ESR1 |  |  |  |  |  |
| 35 | BAX |  |  |  |  |  |
| 36 | RET |  |  |  |  |  |
| 37 | PMS2 |  |  |  |  |  |
| 38 | MIR34A |  |  |  |  |  |
| 39 | TERT |  |  |  |  |  |
| 40 | SMAD4 |  |  |  |  |  |
| 41 | BCL2 |  |  |  |  |  |
| 42 | STAT3 |  |  |  |  |  |
| 43 | DICER1 |  |  |  |  |  |
| 44 | BRIP1 |  |  |  |  |  |
| 45 | MTOR |  |  |  |  |  |
| 46 | NRAS |  |  |  |  |  |
| 47 | IL1B |  |  |  |  |  |
| 48 | VEGFA |  |  |  |  |  |
| 49 | TGFB1 |  |  |  |  |  |
| 50 | FGFR2 |  |  |  |  |  |
| 51 | FASLG |  |  |  |  |  |
| 52 | MIR145 |  |  |  |  |  |
| 53 | NF1 |  |  |  |  |  |
| 54 | CASP3 |  |  |  |  |  |
| 55 | CDKN1B |  |  |  |  |  |
| 56 | MIR17 |  |  |  |  |  |
| 57 | CDKN1A |  |  |  |  |  |
| 58 | MUTYH |  |  |  |  |  |
| 59 | EGF |  |  |  |  |  |
| 60 | MAP2K1 |  |  |  |  |  |
| 61 | BARD1 |  |  |  |  |  |
| 62 | IFNG |  |  |  |  |  |
| 63 | POLE |  |  |  |  |  |
| 64 | IL10 |  |  |  |  |  |
| 65 | MDM2 |  |  |  |  |  |
| 66 | CDK4 |  |  |  |  |  |
| 67 | MIR221 |  |  |  |  |  |
| 68 | MAPK1 |  |  |  |  |  |
| 69 | PTPRC |  |  |  |  |  |
| 70 | PTCH1 |  |  |  |  |  |
| 71 | AR |  |  |  |  |  |
| 72 | EP300 |  |  |  |  |  |
| 73 | VHL |  |  |  |  |  |
| 74 | ALK |  |  |  |  |  |
| 75 | C11orf65 |  |  |  |  |  |
| 76 | MIR126 |  |  |  |  |  |
| 77 | CXCR4 |  |  |  |  |  |
| 78 | FAS |  |  |  |  |  |
| 79 | MIR205 |  |  |  |  |  |
| 80 | SRC |  |  |  |  |  |
| 81 | PPARG |  |  |  |  |  |
| 82 | TSC2 |  |  |  |  |  |
| 83 | CXCL8 |  |  |  |  |  |
| 84 | SMARCA4 |  |  |  |  |  |
| 85 | MIR155 |  |  |  |  |  |
| 86 | MIR200C |  |  |  |  |  |
| 87 | MIR143 |  |  |  |  |  |
| 88 | HIF1A |  |  |  |  |  |
| 89 | MIR222 |  |  |  |  |  |
| 90 | TGFBR2 |  |  |  |  |  |
| 91 | EPCAM |  |  |  |  |  |
| 92 | MIR141 |  |  |  |  |  |
| 93 | RAD51C |  |  |  |  |  |
| 94 | JAK3 |  |  |  |  |  |
| 95 | MIR146A |  |  |  |  |  |
| 96 | MIR125A |  |  |  |  |  |
| 97 | PIK3R1 |  |  |  |  |  |
| 98 | MIR200B |  |  |  |  |  |
| 99 | FLCN |  |  |  |  |  |
| 100 | JAK2 |  |  |  |  |  |
| 101 | TSC1 |  |  |  |  |  |
| 102 | MIR20A |  |  |  |  |  |
| 103 | POLD1 |  |  |  |  |  |
| 104 | MIR214 |  |  |  |  |  |
| 105 | IL2 |  |  |  |  |  |
| 106 | PTGS2 |  |  |  |  |  |
| 107 | CD44 |  |  |  |  |  |
| 108 | MIR200A |  |  |  |  |  |
| 109 | CD4 |  |  |  |  |  |
| 110 | PTPN11 |  |  |  |  |  |
| 111 | NOTCH1 |  |  |  |  |  |
| 112 | MMP2 |  |  |  |  |  |
| 113 | RAD51 |  |  |  |  |  |
| 114 | ABCB1 |  |  |  |  |  |
| 115 | JUN |  |  |  |  |  |
| 116 | ADA |  |  |  |  |  |
| 117 | AXIN2 |  |  |  |  |  |
| 118 | NFKB1 |  |  |  |  |  |
| 119 | RAD50 |  |  |  |  |  |
| 120 | MIR15A |  |  |  |  |  |
| 121 | CXCL12 |  |  |  |  |  |
| 122 | MMP9 |  |  |  |  |  |
| 123 | CTNNA1 |  |  |  |  |  |
| 124 | STAT1 |  |  |  |  |  |
| 125 | MEN1 |  |  |  |  |  |
| 126 | RAD51D |  |  |  |  |  |
| 127 | FH |  |  |  |  |  |
| 128 | ITGB1 |  |  |  |  |  |
| 129 | BIRC5 |  |  |  |  |  |
| 130 | IGF2 |  |  |  |  |  |
| 131 | BCL10 |  |  |  |  |  |
| 132 | BLM |  |  |  |  |  |
| 133 | SDHB |  |  |  |  |  |
| 134 | MIR210 |  |  |  |  |  |
| 135 | AURKA |  |  |  |  |  |
| 136 | IRF1 |  |  |  |  |  |
| 137 | CDK2 |  |  |  |  |  |
| 138 | RAG1 |  |  |  |  |  |
| 139 | ICAM1 |  |  |  |  |  |
| 140 | TNFRSF10B | |  |  |  |  |
| 141 | PDGFRA |  |  |  |  |  |
| 142 | SOX9 |  |  |  |  |  |
| 143 | LRRC56 |  |  |  |  |  |
| 144 | FHIT |  |  |  |  |  |
| 145 | IL7R |  |  |  |  |  |
| 146 | MIR31 |  |  |  |  |  |
| 147 | FBXW7 |  |  |  |  |  |
| 148 | PRKN |  |  |  |  |  |
| 149 | MIR203A |  |  |  |  |  |
| 150 | KITLG |  |  |  |  |  |
| 151 | FGF2 |  |  |  |  |  |
| 152 | SMARCB1 |  |  |  |  |  |
| 153 | IL4 |  |  |  |  |  |
| 154 | TP63 |  |  |  |  |  |
| 155 | PARP1 |  |  |  |  |  |
| 156 | BMPR1A |  |  |  |  |  |
| 157 | TLR2 |  |  |  |  |  |
| 158 | MIR34C |  |  |  |  |  |
| 159 | CDK1 |  |  |  |  |  |
| 160 | NFKBIA |  |  |  |  |  |
| 161 | MAPK8 |  |  |  |  |  |
| 162 | MIR29A |  |  |  |  |  |
| 163 | MRE11 |  |  |  |  |  |
| 164 | MIR93 |  |  |  |  |  |
| 165 | MUC1 |  |  |  |  |  |
| 166 | MIR127 |  |  |  |  |  |
| 167 | WT1 |  |  |  |  |  |
| 168 | MIRLET7C |  |  |  |  |  |
| 169 | FGFR1 |  |  |  |  |  |
| 170 | HNF1B |  |  |  |  |  |
| 171 | RAF1 |  |  |  |  |  |
| 172 | BAP1 |  |  |  |  |  |
| 173 | CTLA4 |  |  |  |  |  |
| 174 | TP73 |  |  |  |  |  |
| 175 | CASP9 |  |  |  |  |  |
| 176 | CCL2 |  |  |  |  |  |
| 177 | ERCC6 |  |  |  |  |  |
| 178 | GATA2 |  |  |  |  |  |
| 179 | IGF1 |  |  |  |  |  |
| 180 | CD40 |  |  |  |  |  |
| 181 | BCL2L1 |  |  |  |  |  |
| 182 | XRCC2 |  |  |  |  |  |
| 183 | IGF1R |  |  |  |  |  |
| 184 | MIR34B |  |  |  |  |  |
| 185 | KDR |  |  |  |  |  |
| 186 | CYCS |  |  |  |  |  |
| 187 | HLA-DRB1 | |  |  |  |  |
| 188 | CREBBP |  |  |  |  |  |
| 189 | RAD51L3-RFFL | |  |  |  |  |
| 190 | INS |  |  |  |  |  |
| 191 | MIR27A |  |  |  |  |  |
| 192 | MAPK14 |  |  |  |  |  |
| 193 | RUNX1 |  |  |  |  |  |
| 194 | SETD2 |  |  |  |  |  |
| 195 | IL7 |  |  |  |  |  |
| 196 | XIAP |  |  |  |  |  |
| 197 | SP1 |  |  |  |  |  |
| 198 | CD274 |  |  |  |  |  |
| 199 | BUB1B |  |  |  |  |  |
| 200 | MIR106B |  |  |  |  |  |
| 201 | PCNA |  |  |  |  |  |
| 202 | PGR |  |  |  |  |  |
| 203 | VIM |  |  |  |  |  |
| 204 | MIR19A |  |  |  |  |  |
| 205 | MMP1 |  |  |  |  |  |
| 206 | HLA-A |  |  |  |  |  |
| 207 | MSH3 |  |  |  |  |  |
| 208 | CDC73 |  |  |  |  |  |
| 209 | MITF |  |  |  |  |  |
| 210 | MTHFR |  |  |  |  |  |
| 211 | MIR223 |  |  |  |  |  |
| 212 | IL1RN |  |  |  |  |  |
| 213 | TNFSF10 |  |  |  |  |  |
| 214 | RELA |  |  |  |  |  |
| 215 | XRCC3 |  |  |  |  |  |
| 216 | MAPK3 |  |  |  |  |  |
| 217 | ERCC2 |  |  |  |  |  |
| 218 | CCNA2 |  |  |  |  |  |
| 219 | MIR18A |  |  |  |  |  |
| 220 | MLH3 |  |  |  |  |  |
| 221 | NPM1 |  |  |  |  |  |
| 222 | MIR373 |  |  |  |  |  |
| 223 | IL2RA |  |  |  |  |  |
| 224 | PTK2 |  |  |  |  |  |
| 225 | RAG2 |  |  |  |  |  |
| 226 | FGFR4 |  |  |  |  |  |
| 227 | SMAD3 |  |  |  |  |  |
| 228 | SLC2A1 |  |  |  |  |  |
| 229 | CCR5 |  |  |  |  |  |
| 230 | TLR4 |  |  |  |  |  |
| 231 | MIRLET7B |  |  |  |  |  |
| 232 | CSF2 |  |  |  |  |  |
| 233 | OGG1 |  |  |  |  |  |
| 234 | FOS |  |  |  |  |  |
| 235 | FANCC |  |  |  |  |  |
| 236 | FN1 |  |  |  |  |  |
| 237 | PIK3CG |  |  |  |  |  |
| 238 | MIRLET7D |  |  |  |  |  |
| 239 | CAV1 |  |  |  |  |  |
| 240 | RAC1 |  |  |  |  |  |
| 241 | IFNA1 |  |  |  |  |  |
| 242 | ERCC1 |  |  |  |  |  |
| 243 | MIR29C |  |  |  |  |  |
| 244 | MCL1 |  |  |  |  |  |
| 245 | PPP2R1B |  |  |  |  |  |
| 246 | CSF3 |  |  |  |  |  |
| 247 | CDK6 |  |  |  |  |  |
| 248 | CDKN3 |  |  |  |  |  |
| 249 | NFE2L2 |  |  |  |  |  |
| 250 | CCNB1 |  |  |  |  |  |
| 251 | RHOA |  |  |  |  |  |
| 252 | SDHD |  |  |  |  |  |
| 253 | HNF1A |  |  |  |  |  |
| 254 | ERBB3 |  |  |  |  |  |
| 255 | HLA-B |  |  |  |  |  |
| 256 | KLF6 |  |  |  |  |  |
| 257 | DNMT1 |  |  |  |  |  |
| 258 | MIR23B |  |  |  |  |  |
| 259 | ESR2 |  |  |  |  |  |
| 260 | HLA-C |  |  |  |  |  |
| 261 | HLA-DQB1 | |  |  |  |  |
| 262 | MIRLET7G |  |  |  |  |  |
| 263 | SPP1 |  |  |  |  |  |
| 264 | PRKCD |  |  |  |  |  |
| 265 | TFRC |  |  |  |  |  |
| 266 | POU5F1 |  |  |  |  |  |
| 267 | CREB1 |  |  |  |  |  |
| 268 | BCL6 |  |  |  |  |  |
| 269 | ABCG2 |  |  |  |  |  |
| 270 | ABCC1 |  |  |  |  |  |
| 271 | MIR30E |  |  |  |  |  |
| 272 | SUFU |  |  |  |  |  |
| 273 | CDKN2B |  |  |  |  |  |
| 274 | PDCD1 |  |  |  |  |  |
| 275 | MGMT |  |  |  |  |  |
| 276 | HGF |  |  |  |  |  |
| 277 | MIR182 |  |  |  |  |  |
| 278 | PDGFRB |  |  |  |  |  |
| 279 | PHB |  |  |  |  |  |
| 280 | GSTP1 |  |  |  |  |  |
| 281 | TGFA |  |  |  |  |  |
| 282 | KRT7 |  |  |  |  |  |
| 283 | MIR10B |  |  |  |  |  |
| 284 | MIR429 |  |  |  |  |  |
| 285 | MIR183 |  |  |  |  |  |
| 286 | IL3 |  |  |  |  |  |
| 287 | TOP2A |  |  |  |  |  |
| 288 | SOX2 |  |  |  |  |  |
| 289 | PRKCA |  |  |  |  |  |
| 290 | MIR451A |  |  |  |  |  |
| 291 | PLAU |  |  |  |  |  |
| 292 | GDNF |  |  |  |  |  |
| 293 | ATR |  |  |  |  |  |
| 294 | TERC |  |  |  |  |  |
| 295 | ETV6 |  |  |  |  |  |
| 296 | XRCC1 |  |  |  |  |  |
| 297 | ALB |  |  |  |  |  |
| 298 | CD34 |  |  |  |  |  |
| 299 | EZH2 |  |  |  |  |  |
| 300 | WWOX |  |  |  |  |  |
| 301 | ENG |  |  |  |  |  |
| 302 | MKI67 |  |  |  |  |  |
| 303 | SDHA |  |  |  |  |  |
| 304 | FANCM |  |  |  |  |  |
| 305 | E2F1 |  |  |  |  |  |
| 306 | ACTB |  |  |  |  |  |
| 307 | NCAM1 |  |  |  |  |  |
| 308 | HLA-G |  |  |  |  |  |
| 309 | HSP90AA1 | |  |  |  |  |
| 310 | MIR16-1 |  |  |  |  |  |
| 311 | CYP1A1 |  |  |  |  |  |
| 312 | KRT19 |  |  |  |  |  |
| 313 | MIR486-1 |  |  |  |  |  |
| 314 | MYCN |  |  |  |  |  |
| 315 | CD28 |  |  |  |  |  |
| 316 | PECAM1 |  |  |  |  |  |
| 317 | BMP2 |  |  |  |  |  |
| 318 | H19 |  |  |  |  |  |
| 319 | MIR98 |  |  |  |  |  |
| 320 | HOXB13 |  |  |  |  |  |
| 321 | GNAS |  |  |  |  |  |
| 322 | RARA |  |  |  |  |  |
| 323 | MIR107 |  |  |  |  |  |
| 324 | MIR185 |  |  |  |  |  |
| 325 | LOC107303340 | |  |  |  |  |
| 326 | VEGFC |  |  |  |  |  |
| 327 | RASSF1 |  |  |  |  |  |
| 328 | MIR204 |  |  |  |  |  |
| 329 | ARID1A |  |  |  |  |  |
| 330 | NKX2-1 |  |  |  |  |  |
| 331 | MIR140 |  |  |  |  |  |
| 332 | MMP14 |  |  |  |  |  |
| 333 | AKT2 |  |  |  |  |  |
| 334 | GJA1 |  |  |  |  |  |
| 335 | IKBKB |  |  |  |  |  |
| 336 | FOXP3 |  |  |  |  |  |
| 337 | CEACAM5 |  |  |  |  |  |
| 338 | NF2 |  |  |  |  |  |
| 339 | DCC |  |  |  |  |  |
| 340 | EPHB2 |  |  |  |  |  |
| 341 | BMP6 |  |  |  |  |  |
| 342 | MIR133B |  |  |  |  |  |
| 343 | HBB |  |  |  |  |  |
| 344 | MIR137 |  |  |  |  |  |
| 345 | TNFRSF1A | |  |  |  |  |
| 346 | CHEK1 |  |  |  |  |  |
| 347 | KRT8 |  |  |  |  |  |
| 348 | MIR499A |  |  |  |  |  |
| 349 | CASP10 |  |  |  |  |  |
| 350 | IL1A |  |  |  |  |  |
| 351 | MAD1L1 |  |  |  |  |  |
| 352 | B2M |  |  |  |  |  |
| 353 | CA9 |  |  |  |  |  |
| 354 | GSTM1 |  |  |  |  |  |
| 355 | NOS2 |  |  |  |  |  |
| 356 | ANXA5 |  |  |  |  |  |
| 357 | KRT18 |  |  |  |  |  |
| 358 | TYMS |  |  |  |  |  |
| 359 | IDH1 |  |  |  |  |  |
| 360 | VCAM1 |  |  |  |  |  |
| 361 | CYP2A6 |  |  |  |  |  |
| 362 | NTRK1 |  |  |  |  |  |
| 363 | MALAT1 |  |  |  |  |  |
| 364 | RNASEL |  |  |  |  |  |
| 365 | PPM1D |  |  |  |  |  |
| 366 | PMS1 |  |  |  |  |  |
| 367 | CDC42 |  |  |  |  |  |
| 368 | CD36 |  |  |  |  |  |
| 369 | MIR25 |  |  |  |  |  |
| 370 | TIMP1 |  |  |  |  |  |
| 371 | CYP1B1 |  |  |  |  |  |
| 372 | MIR130A |  |  |  |  |  |
| 373 | POT1 |  |  |  |  |  |
| 374 | MIR96 |  |  |  |  |  |
| 375 | SDHC |  |  |  |  |  |
| 376 | HFE |  |  |  |  |  |
| 377 | TLR3 |  |  |  |  |  |
| 378 | CSF1 |  |  |  |  |  |
| 379 | FLT1 |  |  |  |  |  |
| 380 | PLK1 |  |  |  |  |  |
| 381 | KMT2A |  |  |  |  |  |
| 382 | GATA1 |  |  |  |  |  |
| 383 | MAP3K1 |  |  |  |  |  |
| 384 | MIRLET7E |  |  |  |  |  |
| 385 | GREM1 |  |  |  |  |  |
| 386 | IL4R |  |  |  |  |  |
| 387 | MSR1 |  |  |  |  |  |
| 388 | IGFBP3 |  |  |  |  |  |
| 389 | TGFBR1 |  |  |  |  |  |
| 390 | PROM1 |  |  |  |  |  |
| 391 | CCR6 |  |  |  |  |  |
| 392 | CCL5 |  |  |  |  |  |
| 393 | BMP4 |  |  |  |  |  |
| 394 | SOD2 |  |  |  |  |  |
| 395 | TYMP |  |  |  |  |  |
| 396 | HOTAIR |  |  |  |  |  |
| 397 | THBS1 |  |  |  |  |  |
| 398 | MIR195 |  |  |  |  |  |
| 399 | HSPB1 |  |  |  |  |  |
| 400 | ERBB4 |  |  |  |  |  |
| 401 | VDR |  |  |  |  |  |
| 402 | TWIST1 |  |  |  |  |  |
| 403 | CBL |  |  |  |  |  |
| 404 | TGFB2 |  |  |  |  |  |
| 405 | INSR |  |  |  |  |  |
| 406 | LEP |  |  |  |  |  |
| 407 | MIR372 |  |  |  |  |  |
| 408 | HDAC1 |  |  |  |  |  |
| 409 | CDH2 |  |  |  |  |  |
| 410 | GATA3 |  |  |  |  |  |
| 411 | PAX5 |  |  |  |  |  |
| 412 | ENO2 |  |  |  |  |  |
| 413 | ABRAXAS1 | |  |  |  |  |
| 414 | FOXO1 |  |  |  |  |  |
| 415 | RPS6KB1 |  |  |  |  |  |
| 416 | ITGB3 |  |  |  |  |  |

**Table S3 The predicted targets of 4i in Hela cell line.**

| **Table S3** The predicted targets of **4i** in Hela cell line. | | |  |
| --- | --- | --- | --- |
| No. | Gene Symbol | Protein name | degree in PPI network |
| 1 | EGFR | epidermal growth factor receptor(EGFR) | 50 |
| 2 | HRAS | HRas proto-oncogene, GTPase(HRAS) | 47 |
| 3 | MAPK1 | mitogen-activated protein kinase 1(MAPK1) | 47 |
| 4 | SRC | SRC proto-oncogene, non-receptor tyrosine kinase(SRC) | 45 |
| 5 | IGF1 | insulin like growth factor 1(IGF1) | 44 |
| 6 | ALB | albumin(ALB) | 42 |
| 7 | ESR1 | estrogen receptor 1(ESR1) | 41 |
| 8 | MAPK8 | mitogen-activated protein kinase 8(MAPK8) | 40 |
| 9 | CASP3 | caspase 3(CASP3) | 38 |
| 10 | HSP90AA1 | heat shock protein 90 alpha family class A member 1(HSP90AA1) | 38 |
| 11 | MDM2 | MDM2 proto-oncogene(MDM2) | 36 |
| 12 | MMP9 | matrix metallopeptidase 9(MMP9) | 35 |
| 13 | MAPK14 | mitogen-activated protein kinase 14(MAPK14) | 35 |
| 14 | BCL2L1 | BCL2 like 1(BCL2L1) | 35 |
| 15 | IGF1R | insulin like growth factor 1 receptor(IGF1R) | 33 |
| 16 | KDR | kinase insert domain receptor(KDR) | 32 |
| 17 | JAK2 | Janus kinase 2(JAK2) | 31 |
| 18 | MAP2K1 | mitogen-activated protein kinase kinase 1(MAP2K1) | 31 |
| 19 | MMP2 | matrix metallopeptidase 2(MMP2) | 31 |
| 20 | PIK3R1 | phosphoinositide-3-kinase regulatory subunit 1(PIK3R1) | 31 |
| 21 | ANXA5 | annexin A5(ANXA5) | 30 |
| 22 | AR | androgen receptor(AR) | 28 |
| 23 | MET | MET proto-oncogene, receptor tyrosine kinase(MET) | 28 |
| 24 | PGR | progesterone receptor(PGR) | 27 |
| 25 | XIAP | X-linked inhibitor of apoptosis(XIAP) | 27 |
| 26 | STAT1 | signal transducer and activator of transcription 1(STAT1) | 27 |
| 27 | IL2 | interleukin 2(IL2) | 26 |
| 28 | CCNA2 | cyclin A2(CCNA2) | 25 |
| 29 | KIT | KIT proto-oncogene receptor tyrosine kinase(KIT) | 25 |
| 30 | PTPN11 | protein tyrosine phosphatase, non-receptor type 11(PTPN11) | 25 |
| 31 | AKT2 | AKT serine/threonine kinase 2(AKT2) | 24 |
| 32 | PARP1 | poly(ADP-ribose) polymerase 1(PARP1) | 22 |
| 33 | BMP2 | bone morphogenetic protein 2(BMP2) | 22 |
| 34 | CDK2 | cyclin dependent kinase 2(CDK2) | 21 |
| 35 | PPARG | peroxisome proliferator activated receptor gamma(PPARG) | 19 |
| 36 | SOD2 | superoxide dismutase 2, mitochondrial(SOD2) | 19 |
| 37 | CHEK1 | checkpoint kinase 1(CHEK1) | 18 |
| 38 | RAC1 | ras-related C3 botulinum toxin substrate 1 (rho family, small GTP binding protein Rac1)(RAC1) | 16 |
| 39 | INSR | insulin receptor(INSR) | 16 |
| 40 | FGFR2 | fibroblast growth factor receptor 2(FGFR2) | 16 |
| 41 | PIK3CG | phosphatidylinositol-4,5-bisphosphate 3-kinase catalytic subunit gamma(PIK3CG) | 15 |
| 42 | PLAU | plasminogen activator, urokinase(PLAU) | 15 |
| 43 | JAK3 | Janus kinase 3(JAK3) | 15 |
| 44 | AURKA | aurora kinase A(AURKA) | 15 |
| 45 | FGFR1 | fibroblast growth factor receptor 1(FGFR1) | 15 |
| 46 | TGFBR1 | transforming growth factor beta receptor 1(TGFBR1) | 14 |
| 47 | ERBB4 | erb-b2 receptor tyrosine kinase 4(ERBB4) | 14 |
| 48 | VDR | vitamin D (1,25- dihydroxyvitamin D3) receptor(VDR) | 13 |
| 49 | ESR2 | estrogen receptor 2(ESR2) | 13 |
| 50 | CTNNA1 | catenin alpha 1(CTNNA1) | 12 |
| 51 | RARA | retinoic acid receptor alpha(RARA) | 11 |
| 52 | TYMS | thymidylate synthetase(TYMS) | 11 |
| 53 | GSTP1 | glutathione S-transferase pi 1(GSTP1) | 10 |
| 54 | TGFB2 | transforming growth factor beta 2(TGFB2) | 8 |
| 55 | GSTM1 | glutathione S-transferase mu 1(GSTM1) | 5 |
| 56 | ITK | IL2 inducible T-cell kinase(ITK) | 4 |
| 57 | PLA2G2A | phospholipase A2 group IIA(PLA2G2A) | 2 |
| 58 | BST1 | bone marrow stromal cell antigen 1(BST1) | 1 |
| 59 | NQO2 | NAD(P)H quinone dehydrogenase 2(NQO2) | None(dispersed) |

**Table S4 GO terms**

| **Table S4** GO terms | |  |  |  |  |  |  |  |  |
| --- | --- | --- | --- | --- | --- | --- | --- | --- | --- |
| ID | Type | Description | GeneRatio | BgRatio | pvalue | p.adjust | qvalue | geneID | Count |
| GO:0048732 | biological process | gland development | 22/59 | 443/18866 | 3.44E-21 | 1.05E-17 | 4.49E-18 | AKT2/AR/AURKA/BMP2/EGFR/ERBB4/ESR1/FGFR1/FGFR2/HRAS/INSR/JAK2/MAP2K1/MAPK1/MET/PGR/RARA/SRC/TGFB2/TGFBR1/TYMS/VDR | 22 |
| GO:0033002 | biological process | muscle cell proliferation | 18/59 | 244/18866 | 2.20E-20 | 3.36E-17 | 1.44E-17 | EGFR/ERBB4/FGFR1/FGFR2/GSTP1/IGF1/JAK2/MAPK1/MAPK14/MDM2/MMP2/MMP9/NQO2/PPARG/SOD2/STAT1/TGFB2/TGFBR1 | 18 |
| GO:0051897 | biological process | positive regulation of protein kinase B signaling | 16/59 | 178/18866 | 1.53E-19 | 1.56E-16 | 6.67E-17 | EGFR/ERBB4/ESR1/FGFR1/FGFR2/HSP90AA1/IGF1R/INSR/KIT/MET/PIK3CG/PIK3R1/PTPN11/RAC1/SRC/TGFBR1 | 16 |
| GO:0043491 | biological process | protein kinase B signaling | 18/59 | 278/18866 | 2.33E-19 | 1.77E-16 | 7.59E-17 | EGFR/ERBB4/ESR1/FGFR1/FGFR2/HSP90AA1/IGF1/IGF1R/INSR/KDR/KIT/MET/PIK3CG/PIK3R1/PTPN11/RAC1/SRC/TGFBR1 | 18 |
| GO:0014066 | biological process | regulation of phosphatidylinositol 3-kinase signaling | 14/59 | 127/18866 | 1.94E-18 | 1.04E-15 | 4.44E-16 | EGFR/ERBB4/FGFR1/IGF1/IGF1R/INSR/JAK2/KDR/KIT/MAPK1/PIK3CG/PIK3R1/SRC/TGFB2 | 14 |
| GO:0048608 | biological process | reproductive structure development | 20/59 | 443/18866 | 2.04E-18 | 1.04E-15 | 4.44E-16 | AR/BCL2L1/CASP3/CTNNA1/EGFR/ESR1/FGFR2/INSR/KIT/MAP2K1/MAPK1/MAPK14/PGR/PPARG/PTPN11/RARA/SRC/TGFB2/TGFBR1/VDR | 20 |
| GO:0061458 | biological process | reproductive system development | 20/59 | 447/18866 | 2.43E-18 | 1.06E-15 | 4.54E-16 | AR/BCL2L1/CASP3/CTNNA1/EGFR/ESR1/FGFR2/INSR/KIT/MAP2K1/MAPK1/MAPK14/PGR/PPARG/PTPN11/RARA/SRC/TGFB2/TGFBR1/VDR | 20 |
| GO:0014065 | biological process | phosphatidylinositol 3-kinase signaling | 14/59 | 154/18866 | 3.10E-17 | 1.18E-14 | 5.06E-15 | EGFR/ERBB4/FGFR1/IGF1/IGF1R/INSR/JAK2/KDR/KIT/MAPK1/PIK3CG/PIK3R1/SRC/TGFB2 | 14 |
| GO:0051896 | biological process | regulation of protein kinase B signaling | 16/59 | 253/18866 | 4.46E-17 | 1.27E-14 | 5.45E-15 | EGFR/ERBB4/ESR1/FGFR1/FGFR2/HSP90AA1/IGF1R/INSR/KIT/MET/PIK3CG/PIK3R1/PTPN11/RAC1/SRC/TGFBR1 | 16 |
| GO:0014068 | biological process | positive regulation of phosphatidylinositol 3-kinase signaling | 12/59 | 88/18866 | 4.56E-17 | 1.27E-14 | 5.45E-15 | ERBB4/FGFR1/IGF1/IGF1R/INSR/JAK2/KDR/KIT/PIK3CG/PIK3R1/SRC/TGFB2 | 12 |
| GO:0018108 | biological process | peptidyl-tyrosine phosphorylation | 18/59 | 374/18866 | 4.59E-17 | 1.27E-14 | 5.45E-15 | BST1/EGFR/ERBB4/FGFR1/FGFR2/IGF1/IGF1R/IL2/INSR/ITK/JAK2/JAK3/KDR/KIT/MAP2K1/MET/PTPN11/SRC | 18 |
| GO:0018212 | biological process | peptidyl-tyrosine modification | 18/59 | 377/18866 | 5.29E-17 | 1.34E-14 | 5.75E-15 | BST1/EGFR/ERBB4/FGFR1/FGFR2/IGF1/IGF1R/IL2/INSR/ITK/JAK2/JAK3/KDR/KIT/MAP2K1/MET/PTPN11/SRC | 18 |
| GO:0043405 | biological process | regulation of MAP kinase activity | 17/59 | 342/18866 | 2.31E-16 | 5.42E-14 | 2.32E-14 | BMP2/EGFR/FGFR1/GSTP1/HRAS/IGF1/IGF1R/INSR/JAK2/KIT/MAP2K1/MAPK1/MAPK14/PIK3CG/PTPN11/SRC/TGFBR1 | 17 |
| GO:0048015 | biological process | phosphatidylinositol-mediated signaling | 14/59 | 192/18866 | 7.06E-16 | 1.54E-13 | 6.58E-14 | EGFR/ERBB4/FGFR1/IGF1/IGF1R/INSR/JAK2/KDR/KIT/MAPK1/PIK3CG/PIK3R1/SRC/TGFB2 | 14 |
| GO:0048017 | biological process | inositol lipid-mediated signaling | 14/59 | 196/18866 | 9.43E-16 | 1.92E-13 | 8.21E-14 | EGFR/ERBB4/FGFR1/IGF1/IGF1R/INSR/JAK2/KDR/KIT/MAPK1/PIK3CG/PIK3R1/SRC/TGFB2 | 14 |
| GO:0050673 | biological process | epithelial cell proliferation | 18/59 | 453/18866 | 1.32E-15 | 2.51E-13 | 1.08E-13 | AR/BMP2/EGFR/ESR1/FGFR1/FGFR2/HRAS/IGF1/KDR/KIT/MAP2K1/MAPK1/PGR/PPARG/STAT1/TGFB2/TGFBR1/VDR | 18 |
| GO:0043406 | biological process | positive regulation of MAP kinase activity | 15/59 | 264/18866 | 2.39E-15 | 4.29E-13 | 1.83E-13 | BMP2/EGFR/FGFR1/HRAS/IGF1/INSR/JAK2/KIT/MAP2K1/MAPK1/MAPK14/PIK3CG/PTPN11/SRC/TGFBR1 | 15 |
| GO:0062197 | biological process | cellular response to chemical stress | 16/59 | 360/18866 | 1.16E-14 | 1.91E-12 | 8.16E-13 | CASP3/CCNA2/CDK2/EGFR/GSTP1/JAK2/MAPK1/MAPK8/MDM2/MET/MMP2/MMP9/PARP1/PPARG/SOD2/SRC | 16 |
| GO:0000302 | biological process | response to reactive oxygen species | 14/59 | 235/18866 | 1.19E-14 | 1.91E-12 | 8.16E-13 | CASP3/CCNA2/CDK2/EGFR/GSTP1/MAPK1/MAPK8/MDM2/MET/MMP2/MMP9/SOD2/SRC/STAT1 | 14 |
| GO:0061180 | biological process | mammary gland epithelium development | 10/59 | 71/18866 | 1.60E-14 | 2.44E-12 | 1.04E-12 | AKT2/AR/ERBB4/ESR1/FGFR2/JAK2/MAPK1/PGR/SRC/VDR | 10 |
| GO:0071902 | biological process | positive regulation of protein serine/threonine kinase activity | 15/59 | 345/18866 | 1.22E-13 | 1.77E-11 | 7.56E-12 | BMP2/EGFR/FGFR1/HRAS/IGF1/INSR/JAK2/KIT/MAP2K1/MAPK1/MAPK14/PIK3CG/PTPN11/SRC/TGFBR1 | 15 |
| GO:0048660 | biological process | regulation of smooth muscle cell proliferation | 12/59 | 173/18866 | 1.85E-13 | 2.57E-11 | 1.10E-11 | EGFR/FGFR2/GSTP1/IGF1/JAK2/MDM2/MMP2/MMP9/NQO2/PPARG/SOD2/STAT1 | 12 |
| GO:0048659 | biological process | smooth muscle cell proliferation | 12/59 | 175/18866 | 2.13E-13 | 2.82E-11 | 1.21E-11 | EGFR/FGFR2/GSTP1/IGF1/JAK2/MDM2/MMP2/MMP9/NQO2/PPARG/SOD2/STAT1 | 12 |
| GO:0097191 | biological process | extrinsic apoptotic signaling pathway | 13/59 | 230/18866 | 2.31E-13 | 2.93E-11 | 1.25E-11 | AR/BCL2L1/CASP3/CTNNA1/FGFR1/GSTP1/IGF1/IL2/JAK2/PIK3R1/SRC/TGFB2/TGFBR1 | 13 |
| GO:0006979 | biological process | response to oxidative stress | 16/59 | 458/18866 | 4.73E-13 | 5.76E-11 | 2.47E-11 | CASP3/CCNA2/CDK2/EGFR/GSTP1/JAK2/MAPK1/MAPK8/MDM2/MET/MMP2/MMP9/PARP1/SOD2/SRC/STAT1 | 16 |
| GO:0034599 | biological process | cellular response to oxidative stress | 14/59 | 310/18866 | 5.34E-13 | 6.27E-11 | 2.68E-11 | CCNA2/CDK2/EGFR/GSTP1/JAK2/MAPK1/MAPK8/MDM2/MET/MMP2/MMP9/PARP1/SOD2/SRC | 14 |
| GO:0035265 | biological process | organ growth | 12/59 | 191/18866 | 6.05E-13 | 6.84E-11 | 2.93E-11 | AR/ERBB4/ESR1/FGFR1/FGFR2/IGF1/MAPK1/MAPK14/PTPN11/RARA/TGFB2/TGFBR1 | 12 |
| GO:1901653 | biological process | cellular response to peptide | 15/59 | 398/18866 | 9.62E-13 | 1.05E-10 | 4.48E-11 | AKT2/CCNA2/GSTP1/IGF1/IGF1R/INSR/JAK2/JAK3/MDM2/PARP1/PIK3R1/PPARG/PTPN11/SRC/STAT1 | 15 |
| GO:0070371 | biological process | ERK1 and ERK2 cascade | 14/59 | 325/18866 | 1.01E-12 | 1.07E-10 | 4.57E-11 | BMP2/EGFR/ERBB4/FGFR2/GSTP1/HRAS/IGF1/KDR/MAP2K1/MAPK1/NQO2/PLA2G2A/PTPN11/SRC | 14 |
| GO:0071375 | biological process | cellular response to peptide hormone stimulus | 14/59 | 330/18866 | 1.25E-12 | 1.27E-10 | 5.43E-11 | AKT2/CCNA2/GSTP1/IGF1R/INSR/JAK2/JAK3/MDM2/PARP1/PIK3R1/PPARG/PTPN11/SRC/STAT1 | 14 |
| GO:0007548 | biological process | sex differentiation | 13/59 | 280/18866 | 2.83E-12 | 2.78E-10 | 1.19E-10 | AR/BCL2L1/CASP3/CTNNA1/ESR1/INSR/KIT/PGR/PTPN11/RARA/SRC/TGFB2/TGFBR1 | 13 |
| GO:0051222 | biological process | positive regulation of protein transport | 14/59 | 354/18866 | 3.22E-12 | 3.07E-10 | 1.31E-10 | AKT2/EGFR/HRAS/IGF1/IL2/JAK2/MAPK1/MAPK14/MAPK8/MDM2/PIK3R1/RAC1/SRC/TGFB2 | 14 |
| GO:0008406 | biological process | gonad development | 12/59 | 223/18866 | 3.80E-12 | 3.51E-10 | 1.50E-10 | AR/BCL2L1/CASP3/CTNNA1/ESR1/INSR/KIT/PGR/RARA/SRC/TGFB2/TGFBR1 | 12 |
| GO:0034614 | biological process | cellular response to reactive oxygen species | 11/59 | 170/18866 | 4.42E-12 | 3.97E-10 | 1.70E-10 | CCNA2/CDK2/EGFR/MAPK1/MAPK8/MDM2/MET/MMP2/MMP9/SOD2/SRC | 11 |
| GO:0045137 | biological process | development of primary sexual characteristics | 12/59 | 229/18866 | 5.19E-12 | 4.52E-10 | 1.94E-10 | AR/BCL2L1/CASP3/CTNNA1/ESR1/INSR/KIT/PGR/RARA/SRC/TGFB2/TGFBR1 | 12 |
| GO:1904951 | biological process | positive regulation of establishment of protein localization | 14/59 | 370/18866 | 5.83E-12 | 4.94E-10 | 2.11E-10 | AKT2/EGFR/HRAS/IGF1/IL2/JAK2/MAPK1/MAPK14/MAPK8/MDM2/PIK3R1/RAC1/SRC/TGFB2 | 14 |
| GO:1904705 | biological process | regulation of vascular associated smooth muscle cell proliferation | 9/59 | 87/18866 | 6.42E-12 | 5.15E-10 | 2.21E-10 | GSTP1/IGF1/JAK2/MDM2/MMP2/MMP9/NQO2/PPARG/SOD2 | 9 |
| GO:1990874 | biological process | vascular associated smooth muscle cell proliferation | 9/59 | 87/18866 | 6.42E-12 | 5.15E-10 | 2.21E-10 | GSTP1/IGF1/JAK2/MDM2/MMP2/MMP9/NQO2/PPARG/SOD2 | 9 |
| GO:0046777 | biological process | protein autophosphorylation | 12/59 | 237/18866 | 7.77E-12 | 6.08E-10 | 2.60E-10 | AURKA/EGFR/ERBB4/FGFR1/FGFR2/IGF1R/INSR/ITK/JAK2/KDR/KIT/SRC | 12 |
| GO:0050678 | biological process | regulation of epithelial cell proliferation | 14/59 | 395/18866 | 1.40E-11 | 1.07E-09 | 4.56E-10 | AR/BMP2/EGFR/FGFR1/FGFR2/HRAS/IGF1/KDR/PGR/PPARG/STAT1/TGFB2/TGFBR1/VDR | 14 |
| GO:0030879 | biological process | mammary gland development | 10/59 | 142/18866 | 1.94E-11 | 1.45E-09 | 6.19E-10 | AKT2/AR/ERBB4/ESR1/FGFR2/JAK2/MAPK1/PGR/SRC/VDR | 10 |
| GO:0032147 | biological process | activation of protein kinase activity | 13/59 | 331/18866 | 2.31E-11 | 1.60E-09 | 6.86E-10 | BMP2/EGFR/IGF1/INSR/JAK2/KIT/MAP2K1/MAPK1/MAPK14/PTPN11/SRC/TGFB2/TGFBR1 | 13 |
| GO:0071214 | biological process | cellular response to abiotic stimulus | 13/59 | 331/18866 | 2.31E-11 | 1.60E-09 | 6.86E-10 | AKT2/BCL2L1/CASP3/CHEK1/EGFR/HRAS/MAPK14/MAPK8/MDM2/PARP1/PIK3R1/PTPN11/RAC1 | 13 |
| GO:0104004 | biological process | cellular response to environmental stimulus | 13/59 | 331/18866 | 2.31E-11 | 1.60E-09 | 6.86E-10 | AKT2/BCL2L1/CASP3/CHEK1/EGFR/HRAS/MAPK14/MAPK8/MDM2/PARP1/PIK3R1/PTPN11/RAC1 | 13 |
| GO:2001233 | biological process | regulation of apoptotic signaling pathway | 14/59 | 413/18866 | 2.53E-11 | 1.72E-09 | 7.34E-10 | AR/BCL2L1/CTNNA1/FGFR1/GSTP1/IGF1/JAK2/MAPK8/MDM2/MMP9/PARP1/SOD2/SRC/TGFBR1 | 14 |
| GO:0031099 | biological process | regeneration | 11/59 | 201/18866 | 2.73E-11 | 1.81E-09 | 7.75E-10 | AURKA/CCNA2/CTNNA1/EGFR/ERBB4/GSTP1/IGF1/JAK2/MAP2K1/PPARG/TYMS | 11 |
| GO:0048661 | biological process | positive regulation of smooth muscle cell proliferation | 9/59 | 103/18866 | 3.02E-11 | 1.96E-09 | 8.40E-10 | EGFR/FGFR2/IGF1/JAK2/MDM2/MMP2/MMP9/NQO2/STAT1 | 9 |
| GO:0048545 | biological process | response to steroid hormone | 13/59 | 346/18866 | 4.02E-11 | 2.55E-09 | 1.09E-09 | AR/CASP3/EGFR/ESR1/ESR2/GSTP1/JAK2/MDM2/PARP1/PGR/SRC/TGFB2/TYMS | 13 |
| GO:0070374 | biological process | positive regulation of ERK1 and ERK2 cascade | 11/59 | 215/18866 | 5.65E-11 | 3.52E-09 | 1.50E-09 | BMP2/EGFR/ERBB4/FGFR2/HRAS/KDR/MAP2K1/NQO2/PLA2G2A/PTPN11/SRC | 11 |
| GO:0043434 | biological process | response to peptide hormone | 14/59 | 447/18866 | 7.22E-11 | 4.40E-09 | 1.88E-09 | AKT2/CCNA2/GSTP1/IGF1R/INSR/JAK2/JAK3/MDM2/PARP1/PIK3R1/PPARG/PTPN11/SRC/STAT1 | 14 |
| GO:0070372 | biological process | regulation of ERK1 and ERK2 cascade | 12/59 | 306/18866 | 1.52E-10 | 9.06E-09 | 3.88E-09 | BMP2/EGFR/ERBB4/FGFR2/GSTP1/HRAS/KDR/MAP2K1/NQO2/PLA2G2A/PTPN11/SRC | 12 |
| GO:0022612 | biological process | gland morphogenesis | 9/59 | 124/18866 | 1.63E-10 | 9.54E-09 | 4.08E-09 | AR/EGFR/ESR1/FGFR1/FGFR2/PGR/SRC/TGFB2/VDR | 9 |
| GO:0014855 | biological process | striated muscle cell proliferation | 8/59 | 83/18866 | 1.84E-10 | 1.06E-08 | 4.52E-09 | ERBB4/FGFR1/FGFR2/JAK2/MAPK1/MAPK14/TGFB2/TGFBR1 | 8 |
| GO:0045787 | biological process | positive regulation of cell cycle | 13/59 | 396/18866 | 2.13E-10 | 1.20E-08 | 5.15E-09 | AURKA/CDK2/CHEK1/EGFR/FGFR1/FGFR2/IGF1/INSR/MDM2/PTPN11/RARA/SRC/TGFB2 | 13 |
| GO:0061138 | biological process | morphogenesis of a branching epithelium | 10/59 | 187/18866 | 2.97E-10 | 1.64E-08 | 7.04E-09 | AR/BMP2/ESR1/FGFR1/FGFR2/KDR/MET/PGR/SRC/VDR | 10 |
| GO:0060562 | biological process | epithelial tube morphogenesis | 12/59 | 331/18866 | 3.73E-10 | 2.03E-08 | 8.69E-09 | AR/BMP2/CASP3/ESR1/FGFR2/KDR/MET/PGR/RARA/SRC/TGFB2/VDR | 12 |
| GO:1903829 | biological process | positive regulation of cellular protein localization | 12/59 | 338/18866 | 4.74E-10 | 2.54E-08 | 1.08E-08 | AKT2/EGFR/ERBB4/HRAS/JAK2/MAPK1/MAPK14/MAPK8/MDM2/PARP1/PIK3R1/SRC | 12 |
| GO:0050727 | biological process | regulation of inflammatory response | 13/59 | 425/18866 | 5.07E-10 | 2.66E-08 | 1.14E-08 | BST1/EGFR/ESR1/GSTP1/IGF1/IL2/JAK2/MAPK14/MMP9/PIK3CG/PLA2G2A/PPARG/XIAP | 13 |
| GO:0008584 | biological process | male gonad development | 9/59 | 141/18866 | 5.15E-10 | 2.66E-08 | 1.14E-08 | AR/BCL2L1/CTNNA1/ESR1/INSR/KIT/RARA/TGFB2/TGFBR1 | 9 |
| GO:0030522 | biological process | intracellular receptor signaling pathway | 11/59 | 265/18866 | 5.26E-10 | 2.68E-08 | 1.15E-08 | AR/ESR1/ESR2/JAK2/PARP1/PGR/PPARG/RARA/SRC/VDR/XIAP | 11 |
| GO:0046546 | biological process | development of primary male sexual characteristics | 9/59 | 142/18866 | 5.48E-10 | 2.74E-08 | 1.17E-08 | AR/BCL2L1/CTNNA1/ESR1/INSR/KIT/RARA/TGFB2/TGFBR1 | 9 |
| GO:0009755 | biological process | hormone-mediated signaling pathway | 10/59 | 200/18866 | 5.72E-10 | 2.82E-08 | 1.21E-08 | AR/ESR1/ESR2/JAK2/PARP1/PGR/PPARG/PTPN11/RARA/SRC | 10 |
| GO:0001763 | biological process | morphogenesis of a branching structure | 10/59 | 201/18866 | 6.01E-10 | 2.86E-08 | 1.23E-08 | AR/BMP2/ESR1/FGFR1/FGFR2/KDR/MET/PGR/SRC/VDR | 10 |
| GO:0030099 | biological process | myeloid cell differentiation | 13/59 | 431/18866 | 6.01E-10 | 2.86E-08 | 1.23E-08 | CASP3/JAK2/JAK3/KIT/MAPK14/MMP9/PARP1/PIK3R1/PPARG/PTPN11/RARA/SRC/STAT1 | 13 |
| GO:0060603 | biological process | mammary gland duct morphogenesis | 6/59 | 32/18866 | 6.13E-10 | 2.88E-08 | 1.23E-08 | AR/ESR1/FGFR2/PGR/SRC/VDR | 6 |
| GO:0045927 | biological process | positive regulation of growth | 11/59 | 274/18866 | 7.49E-10 | 3.46E-08 | 1.48E-08 | EGFR/ERBB4/FGFR1/FGFR2/IGF1/IL2/INSR/MAPK1/MAPK14/TGFB2/TGFBR1 | 11 |
| GO:0008585 | biological process | female gonad development | 8/59 | 100/18866 | 8.32E-10 | 3.79E-08 | 1.62E-08 | BCL2L1/CASP3/CTNNA1/ESR1/INSR/KIT/PGR/SRC | 8 |
| GO:0010518 | biological process | positive regulation of phospholipase activity | 7/59 | 62/18866 | 8.71E-10 | 3.88E-08 | 1.66E-08 | EGFR/ESR1/FGFR1/FGFR2/HRAS/ITK/KIT | 7 |
| GO:0009612 | biological process | response to mechanical stimulus | 10/59 | 209/18866 | 8.79E-10 | 3.88E-08 | 1.66E-08 | CHEK1/EGFR/KIT/MAPK14/MAPK8/PPARG/PTPN11/RAC1/SRC/STAT1 | 10 |
| GO:0055024 | biological process | regulation of cardiac muscle tissue development | 8/59 | 102/18866 | 9.76E-10 | 4.25E-08 | 1.82E-08 | BMP2/ERBB4/FGFR1/FGFR2/IGF1/MAPK1/MAPK14/TGFBR1 | 8 |
| GO:0048754 | biological process | branching morphogenesis of an epithelial tube | 9/59 | 155/18866 | 1.19E-09 | 5.12E-08 | 2.19E-08 | AR/BMP2/ESR1/FGFR2/KDR/MET/PGR/SRC/VDR | 9 |
| GO:0051098 | biological process | regulation of binding | 12/59 | 367/18866 | 1.21E-09 | 5.12E-08 | 2.19E-08 | AURKA/BMP2/IGF1/JAK2/MAPK8/MET/MMP9/PARP1/PPARG/RARA/SRC/TGFBR1 | 12 |
| GO:0046545 | biological process | development of primary female sexual characteristics | 8/59 | 105/18866 | 1.23E-09 | 5.15E-08 | 2.20E-08 | BCL2L1/CASP3/CTNNA1/ESR1/INSR/KIT/PGR/SRC | 8 |
| GO:0055017 | biological process | cardiac muscle tissue growth | 8/59 | 107/18866 | 1.43E-09 | 5.91E-08 | 2.53E-08 | ERBB4/FGFR1/FGFR2/IGF1/MAPK1/MAPK14/TGFB2/TGFBR1 | 8 |
| GO:0032869 | biological process | cellular response to insulin stimulus | 10/59 | 226/18866 | 1.88E-09 | 7.54E-08 | 3.23E-08 | AKT2/GSTP1/IGF1R/INSR/PARP1/PIK3R1/PPARG/PTPN11/SRC/STAT1 | 10 |
| GO:0060038 | biological process | cardiac muscle cell proliferation | 7/59 | 69/18866 | 1.88E-09 | 7.54E-08 | 3.23E-08 | ERBB4/FGFR1/FGFR2/MAPK1/MAPK14/TGFB2/TGFBR1 | 7 |
| GO:0046661 | biological process | male sex differentiation | 9/59 | 164/18866 | 1.97E-09 | 7.80E-08 | 3.34E-08 | AR/BCL2L1/CTNNA1/ESR1/INSR/KIT/RARA/TGFB2/TGFBR1 | 9 |
| GO:0046326 | biological process | positive regulation of glucose import | 6/59 | 39/18866 | 2.17E-09 | 8.48E-08 | 3.63E-08 | AKT2/IGF1/INSR/MAPK14/PIK3R1/PTPN11 | 6 |
| GO:0048738 | biological process | cardiac muscle tissue development | 10/59 | 231/18866 | 2.32E-09 | 8.95E-08 | 3.83E-08 | BMP2/ERBB4/FGFR1/FGFR2/IGF1/MAPK1/MAPK14/RARA/TGFB2/TGFBR1 | 10 |
| GO:2001234 | biological process | negative regulation of apoptotic signaling pathway | 10/59 | 233/18866 | 2.52E-09 | 9.60E-08 | 4.11E-08 | AR/BCL2L1/CTNNA1/GSTP1/IGF1/MDM2/MMP9/SOD2/SRC/TGFBR1 | 10 |
| GO:0060419 | biological process | heart growth | 8/59 | 115/18866 | 2.55E-09 | 9.60E-08 | 4.11E-08 | ERBB4/FGFR1/FGFR2/IGF1/MAPK1/MAPK14/TGFB2/TGFBR1 | 8 |
| GO:0097305 | biological process | response to alcohol | 10/59 | 234/18866 | 2.62E-09 | 9.76E-08 | 4.18E-08 | BCL2L1/CTNNA1/FGFR2/GSTP1/IL2/PARP1/PPARG/RARA/TGFBR1/TYMS | 10 |
| GO:0010517 | biological process | regulation of phospholipase activity | 7/59 | 73/18866 | 2.81E-09 | 1.03E-07 | 4.41E-08 | EGFR/ESR1/FGFR1/FGFR2/HRAS/ITK/KIT | 7 |
| GO:0060326 | biological process | cell chemotaxis | 11/59 | 311/18866 | 2.84E-09 | 1.03E-07 | 4.41E-08 | BST1/FGFR1/GSTP1/KDR/KIT/MAPK1/MAPK14/MET/PIK3CG/RAC1/TGFB2 | 11 |
| GO:0038083 | biological process | peptidyl-tyrosine autophosphorylation | 6/59 | 41/18866 | 2.98E-09 | 1.07E-07 | 4.57E-08 | EGFR/IGF1R/INSR/ITK/KDR/SRC | 6 |
| GO:0046660 | biological process | female sex differentiation | 8/59 | 119/18866 | 3.35E-09 | 1.19E-07 | 5.08E-08 | BCL2L1/CASP3/CTNNA1/ESR1/INSR/KIT/PGR/SRC | 8 |
| GO:0043627 | biological process | response to estrogen | 7/59 | 75/18866 | 3.41E-09 | 1.19E-07 | 5.11E-08 | AR/CTNNA1/ESR1/MAPK1/MDM2/PPARG/RARA | 7 |
| GO:0060193 | biological process | positive regulation of lipase activity | 7/59 | 76/18866 | 3.74E-09 | 1.30E-07 | 5.55E-08 | EGFR/ESR1/FGFR1/FGFR2/HRAS/ITK/KIT | 7 |
| GO:0051402 | biological process | neuron apoptotic process | 10/59 | 245/18866 | 4.08E-09 | 1.40E-07 | 5.99E-08 | AKT2/BCL2L1/CASP3/HRAS/JAK2/NQO2/PARP1/SOD2/TGFB2/XIAP | 10 |
| GO:0071496 | biological process | cellular response to external stimulus | 11/59 | 326/18866 | 4.63E-09 | 1.57E-07 | 6.72E-08 | ALB/CHEK1/EGFR/GSTP1/MAPK1/MAPK8/MDM2/PPARG/PTPN11/RAC1/VDR | 11 |
| GO:0010828 | biological process | positive regulation of glucose transmembrane transport | 6/59 | 45/18866 | 5.34E-09 | 1.77E-07 | 7.56E-08 | AKT2/IGF1/INSR/MAPK14/PIK3R1/PTPN11 | 6 |
| GO:0060443 | biological process | mammary gland morphogenesis | 6/59 | 45/18866 | 5.34E-09 | 1.77E-07 | 7.56E-08 | AR/ESR1/FGFR2/PGR/SRC/VDR | 6 |
| GO:0055021 | biological process | regulation of cardiac muscle tissue growth | 7/59 | 80/18866 | 5.39E-09 | 1.77E-07 | 7.56E-08 | ERBB4/FGFR1/FGFR2/IGF1/MAPK1/MAPK14/TGFBR1 | 7 |
| GO:2000027 | biological process | regulation of animal organ morphogenesis | 10/59 | 254/18866 | 5.77E-09 | 1.85E-07 | 7.94E-08 | AR/BMP2/ESR1/FGFR1/FGFR2/RAC1/STAT1/TGFB2/TGFBR1/VDR | 10 |
| GO:0018209 | biological process | peptidyl-serine modification | 11/59 | 333/18866 | 5.78E-09 | 1.85E-07 | 7.94E-08 | AKT2/AURKA/CDK2/EGFR/HSP90AA1/MAPK1/MAPK14/MAPK8/PARP1/SRC/TGFBR1 | 11 |
| GO:0010863 | biological process | positive regulation of phospholipase C activity | 6/59 | 46/18866 | 6.12E-09 | 1.95E-07 | 8.33E-08 | EGFR/ESR1/FGFR1/HRAS/ITK/KIT | 6 |
| GO:0055023 | biological process | positive regulation of cardiac muscle tissue growth | 6/59 | 48/18866 | 7.99E-09 | 2.49E-07 | 1.06E-07 | ERBB4/FGFR1/FGFR2/IGF1/MAPK1/MAPK14 | 6 |
| GO:1900274 | biological process | regulation of phospholipase C activity | 6/59 | 48/18866 | 7.99E-09 | 2.49E-07 | 1.06E-07 | EGFR/ESR1/FGFR1/HRAS/ITK/KIT | 6 |
| GO:0110110 | biological process | positive regulation of animal organ morphogenesis | 7/59 | 85/18866 | 8.27E-09 | 2.55E-07 | 1.09E-07 | AR/BMP2/FGFR1/FGFR2/TGFB2/TGFBR1/VDR | 7 |
| GO:0022407 | biological process | regulation of cell-cell adhesion | 12/59 | 439/18866 | 9.03E-09 | 2.75E-07 | 1.18E-07 | BMP2/CASP3/IGF1/IL2/JAK2/JAK3/MAPK14/PIK3R1/PTPN11/RAC1/RARA/SRC | 12 |
| GO:0060420 | biological process | regulation of heart growth | 7/59 | 87/18866 | 9.75E-09 | 2.94E-07 | 1.26E-07 | ERBB4/FGFR1/FGFR2/IGF1/MAPK1/MAPK14/TGFBR1 | 7 |
| GO:0060444 | biological process | branching involved in mammary gland duct morphogenesis | 5/59 | 24/18866 | 1.02E-08 | 3.05E-07 | 1.30E-07 | AR/ESR1/PGR/SRC/VDR | 5 |
| GO:1904707 | biological process | positive regulation of vascular associated smooth muscle cell proliferation | 6/59 | 50/18866 | 1.03E-08 | 3.05E-07 | 1.30E-07 | IGF1/JAK2/MDM2/MMP2/MMP9/NQO2 | 6 |
| GO:0009314 | biological process | response to radiation | 12/59 | 447/18866 | 1.10E-08 | 3.24E-07 | 1.39E-07 | AKT2/BCL2L1/CASP3/CHEK1/EGFR/HRAS/KIT/MAPK14/MAPK8/MDM2/PARP1/PIK3R1 | 12 |
| GO:2000377 | biological process | regulation of reactive oxygen species metabolic process | 9/59 | 200/18866 | 1.12E-08 | 3.26E-07 | 1.40E-07 | BST1/EGFR/GSTP1/HSP90AA1/INSR/JAK2/MAPK14/NQO2/RAC1 | 9 |
| GO:0032386 | biological process | regulation of intracellular transport | 11/59 | 358/18866 | 1.22E-08 | 3.52E-07 | 1.50E-07 | AKT2/HRAS/JAK2/MAP2K1/MAPK1/MAPK14/MAPK8/MDM2/PIK3R1/PTPN11/SRC | 11 |
| GO:0060421 | biological process | positive regulation of heart growth | 6/59 | 52/18866 | 1.31E-08 | 3.74E-07 | 1.60E-07 | ERBB4/FGFR1/FGFR2/IGF1/MAPK1/MAPK14 | 6 |
| GO:0033273 | biological process | response to vitamin | 7/59 | 91/18866 | 1.34E-08 | 3.78E-07 | 1.62E-07 | EGFR/GSTP1/MDM2/PPARG/RARA/TYMS/VDR | 7 |
| GO:0071383 | biological process | cellular response to steroid hormone stimulus | 9/59 | 206/18866 | 1.45E-08 | 4.06E-07 | 1.74E-07 | AR/EGFR/ESR1/ESR2/GSTP1/JAK2/PARP1/PGR/SRC | 9 |
| GO:0038127 | biological process | ERBB signaling pathway | 8/59 | 145/18866 | 1.60E-08 | 4.43E-07 | 1.89E-07 | EGFR/ERBB4/HSP90AA1/MAPK1/MMP9/PIK3R1/PTPN11/SRC | 8 |
| GO:0032868 | biological process | response to insulin | 10/59 | 283/18866 | 1.62E-08 | 4.45E-07 | 1.90E-07 | AKT2/GSTP1/IGF1R/INSR/PARP1/PIK3R1/PPARG/PTPN11/SRC/STAT1 | 10 |
| GO:0050679 | biological process | positive regulation of epithelial cell proliferation | 9/59 | 211/18866 | 1.79E-08 | 4.83E-07 | 2.07E-07 | AR/BMP2/EGFR/FGFR1/FGFR2/HRAS/IGF1/KDR/TGFBR1 | 9 |
| GO:0051403 | biological process | stress-activated MAPK cascade | 10/59 | 286/18866 | 1.79E-08 | 4.83E-07 | 2.07E-07 | BMP2/EGFR/GSTP1/HRAS/IGF1R/MAP2K1/MAPK1/MAPK14/MAPK8/TGFB2 | 10 |
| GO:0072593 | biological process | reactive oxygen species metabolic process | 10/59 | 288/18866 | 1.91E-08 | 5.11E-07 | 2.19E-07 | BST1/EGFR/GSTP1/HSP90AA1/INSR/JAK2/MAPK14/NQO2/RAC1/SOD2 | 10 |
| GO:0097193 | biological process | intrinsic apoptotic signaling pathway | 10/59 | 290/18866 | 2.04E-08 | 5.41E-07 | 2.31E-07 | BCL2L1/CASP3/HRAS/JAK2/MDM2/MMP9/PARP1/PIK3R1/SOD2/SRC | 10 |
| GO:0031667 | biological process | response to nutrient levels | 12/59 | 473/18866 | 2.06E-08 | 5.43E-07 | 2.32E-07 | ALB/EGFR/GSTP1/MAPK1/MAPK8/MDM2/PPARG/RARA/SRC/STAT1/TYMS/VDR | 12 |
| GO:0016202 | biological process | regulation of striated muscle tissue development | 8/59 | 153/18866 | 2.43E-08 | 6.34E-07 | 2.71E-07 | BMP2/ERBB4/FGFR1/FGFR2/IGF1/MAPK1/MAPK14/TGFBR1 | 8 |
| GO:0042110 | biological process | T cell activation | 12/59 | 483/18866 | 2.60E-08 | 6.72E-07 | 2.87E-07 | CASP3/IGF1/IL2/ITK/JAK3/KIT/PIK3CG/PIK3R1/PTPN11/RAC1/RARA/SRC | 12 |
| GO:0060191 | biological process | regulation of lipase activity | 7/59 | 101/18866 | 2.77E-08 | 7.08E-07 | 3.03E-07 | EGFR/ESR1/FGFR1/FGFR2/HRAS/ITK/KIT | 7 |
| GO:0031098 | biological process | stress-activated protein kinase signaling cascade | 10/59 | 300/18866 | 2.81E-08 | 7.08E-07 | 3.03E-07 | BMP2/EGFR/GSTP1/HRAS/IGF1R/MAP2K1/MAPK1/MAPK14/MAPK8/TGFB2 | 10 |
| GO:0000187 | biological process | activation of MAPK activity | 8/59 | 156/18866 | 2.83E-08 | 7.08E-07 | 3.03E-07 | BMP2/IGF1/INSR/KIT/MAP2K1/MAPK1/MAPK14/PTPN11 | 8 |
| GO:1901861 | biological process | regulation of muscle tissue development | 8/59 | 156/18866 | 2.83E-08 | 7.08E-07 | 3.03E-07 | BMP2/ERBB4/FGFR1/FGFR2/IGF1/MAPK1/MAPK14/TGFBR1 | 8 |
| GO:0060043 | biological process | regulation of cardiac muscle cell proliferation | 6/59 | 59/18866 | 2.86E-08 | 7.08E-07 | 3.03E-07 | ERBB4/FGFR1/FGFR2/MAPK1/MAPK14/TGFBR1 | 6 |
| GO:0048634 | biological process | regulation of muscle organ development | 8/59 | 157/18866 | 2.98E-08 | 7.32E-07 | 3.13E-07 | BMP2/ERBB4/FGFR1/FGFR2/IGF1/MAPK1/MAPK14/TGFBR1 | 8 |
| GO:0046324 | biological process | regulation of glucose import | 6/59 | 61/18866 | 3.50E-08 | 8.54E-07 | 3.66E-07 | AKT2/IGF1/INSR/MAPK14/PIK3R1/PTPN11 | 6 |
| GO:2001236 | biological process | regulation of extrinsic apoptotic signaling pathway | 8/59 | 162/18866 | 3.80E-08 | 9.18E-07 | 3.93E-07 | AR/BCL2L1/CTNNA1/FGFR1/GSTP1/IGF1/SRC/TGFBR1 | 8 |
| GO:0018105 | biological process | peptidyl-serine phosphorylation | 10/59 | 310/18866 | 3.83E-08 | 9.18E-07 | 3.93E-07 | AKT2/AURKA/CDK2/EGFR/HSP90AA1/MAPK1/MAPK14/MAPK8/SRC/TGFBR1 | 10 |
| GO:0055025 | biological process | positive regulation of cardiac muscle tissue development | 6/59 | 62/18866 | 3.87E-08 | 9.18E-07 | 3.93E-07 | ERBB4/FGFR1/FGFR2/IGF1/MAPK1/MAPK14 | 6 |
| GO:2000379 | biological process | positive regulation of reactive oxygen species metabolic process | 7/59 | 106/18866 | 3.88E-08 | 9.18E-07 | 3.93E-07 | EGFR/GSTP1/HSP90AA1/INSR/JAK2/MAPK14/NQO2 | 7 |
| GO:2001237 | biological process | negative regulation of extrinsic apoptotic signaling pathway | 7/59 | 107/18866 | 4.14E-08 | 9.72E-07 | 4.16E-07 | AR/BCL2L1/CTNNA1/GSTP1/IGF1/SRC/TGFBR1 | 7 |
| GO:0046622 | biological process | positive regulation of organ growth | 6/59 | 63/18866 | 4.26E-08 | 9.93E-07 | 4.25E-07 | ERBB4/FGFR1/FGFR2/IGF1/MAPK1/MAPK14 | 6 |
| GO:0071695 | biological process | anatomical structure maturation | 9/59 | 235/18866 | 4.53E-08 | 1.05E-06 | 4.48E-07 | AURKA/BMP2/FGFR1/IGF1/MMP2/PGR/PPARG/TGFB2/TYMS | 9 |
| GO:0038128 | biological process | ERBB2 signaling pathway | 5/59 | 32/18866 | 4.75E-08 | 1.09E-06 | 4.66E-07 | EGFR/ERBB4/HSP90AA1/PIK3R1/SRC | 5 |
| GO:0048645 | biological process | animal organ formation | 6/59 | 65/18866 | 5.16E-08 | 1.17E-06 | 5.03E-07 | AR/BMP2/FGFR1/FGFR2/MAP2K1/MAPK1 | 6 |
| GO:0007584 | biological process | response to nutrient | 8/59 | 171/18866 | 5.78E-08 | 1.31E-06 | 5.59E-07 | EGFR/GSTP1/MDM2/PPARG/RARA/STAT1/TYMS/VDR | 8 |
| GO:0051099 | biological process | positive regulation of binding | 8/59 | 174/18866 | 6.62E-08 | 1.48E-06 | 6.35E-07 | BMP2/IGF1/JAK2/MET/MMP9/PARP1/PPARG/RARA | 8 |
| GO:0001655 | biological process | urogenital system development | 10/59 | 330/18866 | 6.88E-08 | 1.53E-06 | 6.55E-07 | AR/BMP2/ESR1/FGFR1/FGFR2/MMP9/RARA/STAT1/TGFB2/TGFBR1 | 10 |
| GO:0030518 | biological process | intracellular steroid hormone receptor signaling pathway | 7/59 | 116/18866 | 7.25E-08 | 1.59E-06 | 6.81E-07 | AR/ESR1/ESR2/JAK2/PARP1/PGR/SRC | 7 |
| GO:0046620 | biological process | regulation of organ growth | 7/59 | 116/18866 | 7.25E-08 | 1.59E-06 | 6.81E-07 | ERBB4/FGFR1/FGFR2/IGF1/MAPK1/MAPK14/TGFBR1 | 7 |
| GO:0042698 | biological process | ovulation cycle | 6/59 | 69/18866 | 7.42E-08 | 1.62E-06 | 6.91E-07 | CASP3/EGFR/ESR1/PGR/SRC/TGFB2 | 6 |
| GO:0045785 | biological process | positive regulation of cell adhesion | 11/59 | 428/18866 | 7.58E-08 | 1.64E-06 | 7.01E-07 | IGF1/IL2/JAK2/JAK3/KDR/PIK3R1/PTPN11/RAC1/RARA/SRC/TGFB2 | 11 |
| GO:0090316 | biological process | positive regulation of intracellular protein transport | 8/59 | 182/18866 | 9.37E-08 | 2.01E-06 | 8.60E-07 | AKT2/HRAS/JAK2/MAPK1/MAPK14/MAPK8/MDM2/PIK3R1 | 8 |
| GO:0048872 | biological process | homeostasis of number of cells | 9/59 | 256/18866 | 9.42E-08 | 2.01E-06 | 8.60E-07 | CASP3/IL2/JAK2/JAK3/KIT/MAPK14/PLA2G2A/PTPN11/STAT1 | 9 |
| GO:0051147 | biological process | regulation of muscle cell differentiation | 8/59 | 186/18866 | 1.11E-07 | 2.35E-06 | 1.00E-06 | BMP2/CTNNA1/FGFR2/IGF1/KIT/MAPK14/MDM2/SOD2 | 8 |
| GO:0048009 | biological process | insulin-like growth factor receptor signaling pathway | 5/59 | 38/18866 | 1.17E-07 | 2.44E-06 | 1.04E-06 | AR/BMP2/IGF1/IGF1R/PIK3R1 | 5 |
| GO:0060045 | biological process | positive regulation of cardiac muscle cell proliferation | 5/59 | 38/18866 | 1.17E-07 | 2.44E-06 | 1.04E-06 | ERBB4/FGFR1/FGFR2/MAPK1/MAPK14 | 5 |
| GO:0050730 | biological process | regulation of peptidyl-tyrosine phosphorylation | 9/59 | 263/18866 | 1.19E-07 | 2.46E-06 | 1.05E-06 | BST1/EGFR/ERBB4/IGF1/IL2/JAK2/KIT/PTPN11/SRC | 9 |
| GO:0071478 | biological process | cellular response to radiation | 8/59 | 188/18866 | 1.20E-07 | 2.48E-06 | 1.06E-06 | AKT2/BCL2L1/CHEK1/HRAS/MAPK14/MDM2/PARP1/PIK3R1 | 8 |
| GO:0033157 | biological process | regulation of intracellular protein transport | 9/59 | 264/18866 | 1.22E-07 | 2.48E-06 | 1.06E-06 | AKT2/HRAS/JAK2/MAPK1/MAPK14/MAPK8/MDM2/PIK3R1/PTPN11 | 9 |
| GO:0031100 | biological process | animal organ regeneration | 6/59 | 75/18866 | 1.23E-07 | 2.48E-06 | 1.06E-06 | AURKA/CCNA2/EGFR/GSTP1/PPARG/TYMS | 6 |
| GO:0046323 | biological process | glucose import | 6/59 | 75/18866 | 1.23E-07 | 2.48E-06 | 1.06E-06 | AKT2/IGF1/INSR/MAPK14/PIK3R1/PTPN11 | 6 |
| GO:0016572 | biological process | histone phosphorylation | 5/59 | 40/18866 | 1.52E-07 | 3.02E-06 | 1.29E-06 | AURKA/CCNA2/CDK2/CHEK1/JAK2 | 5 |
| GO:0045740 | biological process | positive regulation of DNA replication | 5/59 | 40/18866 | 1.52E-07 | 3.02E-06 | 1.29E-06 | CDK2/EGFR/FGFR1/HRAS/RAC1 | 5 |
| GO:0071391 | biological process | cellular response to estrogen stimulus | 4/59 | 16/18866 | 1.53E-07 | 3.02E-06 | 1.29E-06 | AR/ESR1/MDM2/RARA | 4 |
| GO:0070997 | biological process | neuron death | 10/59 | 360/18866 | 1.55E-07 | 3.04E-06 | 1.30E-06 | AKT2/BCL2L1/CASP3/HRAS/JAK2/NQO2/PARP1/SOD2/TGFB2/XIAP | 10 |
| GO:0007565 | biological process | female pregnancy | 8/59 | 196/18866 | 1.66E-07 | 3.24E-06 | 1.39E-06 | AR/ESR1/MAPK1/MMP2/MMP9/PGR/RARA/VDR | 8 |
| GO:0010827 | biological process | regulation of glucose transmembrane transport | 6/59 | 79/18866 | 1.68E-07 | 3.26E-06 | 1.40E-06 | AKT2/IGF1/INSR/MAPK14/PIK3R1/PTPN11 | 6 |
| GO:0010631 | biological process | epithelial cell migration | 10/59 | 365/18866 | 1.76E-07 | 3.39E-06 | 1.45E-06 | FGFR1/KDR/KIT/MET/MMP9/PPARG/PTPN11/SRC/TGFB2/TGFBR1 | 10 |
| GO:1900182 | biological process | positive regulation of protein localization to nucleus | 6/59 | 80/18866 | 1.81E-07 | 3.47E-06 | 1.49E-06 | JAK2/MAPK1/MAPK14/PARP1/PIK3R1/SRC | 6 |
| GO:0090132 | biological process | epithelium migration | 10/59 | 368/18866 | 1.90E-07 | 3.60E-06 | 1.54E-06 | FGFR1/KDR/KIT/MET/MMP9/PPARG/PTPN11/SRC/TGFB2/TGFBR1 | 10 |
| GO:0045165 | biological process | cell fate commitment | 9/59 | 278/18866 | 1.90E-07 | 3.60E-06 | 1.54E-06 | AR/BMP2/CASP3/ERBB4/FGFR1/FGFR2/PPARG/RARA/TGFBR1 | 9 |
| GO:1901654 | biological process | response to ketone | 8/59 | 200/18866 | 1.94E-07 | 3.64E-06 | 1.56E-06 | AR/BCL2L1/EGFR/PARP1/PPARG/SRC/TGFB2/TYMS | 8 |
| GO:0090130 | biological process | tissue migration | 10/59 | 374/18866 | 2.20E-07 | 4.12E-06 | 1.76E-06 | FGFR1/KDR/KIT/MET/MMP9/PPARG/PTPN11/SRC/TGFB2/TGFBR1 | 10 |
| GO:0048145 | biological process | regulation of fibroblast proliferation | 6/59 | 83/18866 | 2.26E-07 | 4.20E-06 | 1.80E-06 | CCNA2/EGFR/ESR1/GSTP1/IGF1/PPARG | 6 |
| GO:0048144 | biological process | fibroblast proliferation | 6/59 | 84/18866 | 2.43E-07 | 4.48E-06 | 1.92E-06 | CCNA2/EGFR/ESR1/GSTP1/IGF1/PPARG | 6 |
| GO:0001667 | biological process | ameboidal-type cell migration | 11/59 | 481/18866 | 2.45E-07 | 4.50E-06 | 1.93E-06 | ERBB4/FGFR1/KDR/KIT/MET/MMP9/PPARG/PTPN11/SRC/TGFB2/TGFBR1 | 11 |
| GO:0021700 | biological process | developmental maturation | 9/59 | 287/18866 | 2.49E-07 | 4.54E-06 | 1.94E-06 | AURKA/BMP2/FGFR1/IGF1/MMP2/PGR/PPARG/TGFB2/TYMS | 9 |
| GO:0043401 | biological process | steroid hormone mediated signaling pathway | 7/59 | 139/18866 | 2.51E-07 | 4.56E-06 | 1.95E-06 | AR/ESR1/ESR2/JAK2/PARP1/PGR/SRC | 7 |
| GO:0045844 | biological process | positive regulation of striated muscle tissue development | 6/59 | 85/18866 | 2.60E-07 | 4.67E-06 | 2.00E-06 | ERBB4/FGFR1/FGFR2/IGF1/MAPK1/MAPK14 | 6 |
| GO:0048636 | biological process | positive regulation of muscle organ development | 6/59 | 85/18866 | 2.60E-07 | 4.67E-06 | 2.00E-06 | ERBB4/FGFR1/FGFR2/IGF1/MAPK1/MAPK14 | 6 |
| GO:0009411 | biological process | response to UV | 7/59 | 140/18866 | 2.64E-07 | 4.70E-06 | 2.01E-06 | CASP3/CHEK1/EGFR/MAPK8/MDM2/PARP1/PIK3R1 | 7 |
| GO:0060485 | biological process | mesenchyme development | 9/59 | 290/18866 | 2.71E-07 | 4.81E-06 | 2.06E-06 | BMP2/ERBB4/FGFR1/FGFR2/MAPK1/MDM2/STAT1/TGFB2/TGFBR1 | 9 |
| GO:0001701 | biological process | in utero embryonic development | 10/59 | 383/18866 | 2.74E-07 | 4.83E-06 | 2.07E-06 | AR/BCL2L1/BMP2/EGFR/FGFR1/FGFR2/IGF1/MAP2K1/MAPK1/TGFBR1 | 10 |
| GO:0032355 | biological process | response to estradiol | 7/59 | 141/18866 | 2.77E-07 | 4.85E-06 | 2.07E-06 | CASP3/CCNA2/EGFR/ESR1/ESR2/GSTP1/RARA | 7 |
| GO:1901863 | biological process | positive regulation of muscle tissue development | 6/59 | 86/18866 | 2.79E-07 | 4.87E-06 | 2.08E-06 | ERBB4/FGFR1/FGFR2/IGF1/MAPK1/MAPK14 | 6 |
| GO:0002573 | biological process | myeloid leukocyte differentiation | 8/59 | 210/18866 | 2.81E-07 | 4.87E-06 | 2.08E-06 | KIT/MAPK14/MMP9/PARP1/PIK3R1/PPARG/RARA/SRC | 8 |
| GO:0048013 | biological process | ephrin receptor signaling pathway | 6/59 | 87/18866 | 2.99E-07 | 5.15E-06 | 2.21E-06 | HRAS/MMP2/MMP9/PTPN11/RAC1/SRC | 6 |
| GO:0014706 | biological process | striated muscle tissue development | 10/59 | 389/18866 | 3.16E-07 | 5.42E-06 | 2.32E-06 | BMP2/ERBB4/FGFR1/FGFR2/IGF1/MAPK1/MAPK14/RARA/TGFB2/TGFBR1 | 10 |
| GO:0031647 | biological process | regulation of protein stability | 9/59 | 296/18866 | 3.22E-07 | 5.44E-06 | 2.33E-06 | AURKA/BMP2/CASP3/HSP90AA1/IGF1/MAPK1/MDM2/PIK3R1/SRC | 9 |
| GO:0042692 | biological process | muscle cell differentiation | 10/59 | 390/18866 | 3.24E-07 | 5.44E-06 | 2.33E-06 | BMP2/CASP3/CTNNA1/FGFR2/IGF1/KIT/MAPK14/MDM2/RARA/SOD2 | 10 |
| GO:0043523 | biological process | regulation of neuron apoptotic process | 8/59 | 214/18866 | 3.24E-07 | 5.44E-06 | 2.33E-06 | BCL2L1/CASP3/HRAS/JAK2/NQO2/PARP1/SOD2/TGFB2 | 8 |
| GO:0050870 | biological process | positive regulation of T cell activation | 8/59 | 214/18866 | 3.24E-07 | 5.44E-06 | 2.33E-06 | IGF1/IL2/JAK3/PIK3R1/PTPN11/RAC1/RARA/SRC | 8 |
| GO:0007260 | biological process | tyrosine phosphorylation of STAT protein | 6/59 | 89/18866 | 3.43E-07 | 5.71E-06 | 2.44E-06 | ERBB4/IGF1/IL2/JAK2/JAK3/KIT | 6 |
| GO:0022602 | biological process | ovulation cycle process | 5/59 | 47/18866 | 3.49E-07 | 5.75E-06 | 2.46E-06 | CASP3/ESR1/PGR/SRC/TGFB2 | 5 |
| GO:0070849 | biological process | response to epidermal growth factor | 5/59 | 47/18866 | 3.49E-07 | 5.75E-06 | 2.46E-06 | EGFR/ERBB4/GSTP1/MAPK1/PTPN11 | 5 |
| GO:0055123 | biological process | digestive system development | 7/59 | 147/18866 | 3.67E-07 | 6.02E-06 | 2.58E-06 | EGFR/FGFR2/INSR/KIT/SRC/TGFB2/TYMS | 7 |
| GO:0000075 | biological process | cell cycle checkpoint | 8/59 | 219/18866 | 3.87E-07 | 6.30E-06 | 2.70E-06 | AURKA/BCL2L1/CDK2/CHEK1/HRAS/MAPK14/MDM2/PTPN11 | 8 |
| GO:0060324 | biological process | face development | 5/59 | 48/18866 | 3.89E-07 | 6.30E-06 | 2.70E-06 | MAP2K1/MAPK1/MMP2/PTPN11/RARA | 5 |
| GO:0051767 | biological process | nitric-oxide synthase biosynthetic process | 4/59 | 20/18866 | 4.03E-07 | 6.46E-06 | 2.77E-06 | GSTP1/JAK2/KDR/STAT1 | 4 |
| GO:0051769 | biological process | regulation of nitric-oxide synthase biosynthetic process | 4/59 | 20/18866 | 4.03E-07 | 6.46E-06 | 2.77E-06 | GSTP1/JAK2/KDR/STAT1 | 4 |
| GO:0002064 | biological process | epithelial cell development | 8/59 | 221/18866 | 4.14E-07 | 6.59E-06 | 2.82E-06 | AR/ESR1/FGFR1/MET/PGR/RARA/SRC/TYMS | 8 |
| GO:0048511 | biological process | rhythmic process | 9/59 | 305/18866 | 4.15E-07 | 6.59E-06 | 2.82E-06 | CASP3/EGFR/ESR1/MAPK8/PGR/PPARG/SRC/TGFB2/TYMS | 9 |
| GO:0032388 | biological process | positive regulation of intracellular transport | 8/59 | 222/18866 | 4.29E-07 | 6.78E-06 | 2.90E-06 | AKT2/HRAS/JAK2/MAPK1/MAPK14/MAPK8/MDM2/PIK3R1 | 8 |
| GO:0042063 | biological process | gliogenesis | 9/59 | 307/18866 | 4.38E-07 | 6.89E-06 | 2.95E-06 | AKT2/BMP2/EGFR/GSTP1/MAP2K1/MAPK1/PPARG/PTPN11/TGFB2 | 9 |
| GO:0044706 | biological process | multi-multicellular organism process | 8/59 | 226/18866 | 4.91E-07 | 7.68E-06 | 3.29E-06 | AR/ESR1/MAPK1/MMP2/MMP9/PGR/RARA/VDR | 8 |
| GO:0060537 | biological process | muscle tissue development | 10/59 | 409/18866 | 5.00E-07 | 7.79E-06 | 3.33E-06 | BMP2/ERBB4/FGFR1/FGFR2/IGF1/MAPK1/MAPK14/RARA/TGFB2/TGFBR1 | 10 |
| GO:0070661 | biological process | leukocyte proliferation | 9/59 | 313/18866 | 5.15E-07 | 7.93E-06 | 3.40E-06 | BST1/CASP3/GSTP1/IGF1/IL2/JAK3/KIT/MAPK1/PIK3CG | 9 |
| GO:1903532 | biological process | positive regulation of secretion by cell | 9/59 | 313/18866 | 5.15E-07 | 7.93E-06 | 3.40E-06 | EGFR/FGFR1/IGF1/IL2/JAK2/PTPN11/RAC1/SRC/TGFB2 | 9 |
| GO:0035272 | biological process | exocrine system development | 5/59 | 51/18866 | 5.29E-07 | 8.11E-06 | 3.47E-06 | EGFR/FGFR1/FGFR2/INSR/TGFB2 | 5 |
| GO:0048762 | biological process | mesenchymal cell differentiation | 8/59 | 229/18866 | 5.43E-07 | 8.24E-06 | 3.52E-06 | BMP2/ERBB4/FGFR1/FGFR2/MAPK1/STAT1/TGFB2/TGFBR1 | 8 |
| GO:0050920 | biological process | regulation of chemotaxis | 8/59 | 229/18866 | 5.43E-07 | 8.24E-06 | 3.52E-06 | BST1/FGFR1/GSTP1/KDR/MAPK1/MAPK14/MET/RAC1 | 8 |
| GO:0001890 | biological process | placenta development | 7/59 | 156/18866 | 5.49E-07 | 8.29E-06 | 3.55E-06 | EGFR/FGFR2/MAP2K1/MAPK1/MAPK14/PPARG/VDR | 7 |
| GO:0010001 | biological process | glial cell differentiation | 8/59 | 230/18866 | 5.61E-07 | 8.43E-06 | 3.61E-06 | AKT2/BMP2/EGFR/GSTP1/MAP2K1/MAPK1/PPARG/PTPN11 | 8 |
| GO:0072132 | biological process | mesenchyme morphogenesis | 5/59 | 52/18866 | 5.84E-07 | 8.74E-06 | 3.74E-06 | BMP2/FGFR1/MDM2/TGFB2/TGFBR1 | 5 |
| GO:0009416 | biological process | response to light stimulus | 9/59 | 319/18866 | 6.04E-07 | 8.98E-06 | 3.84E-06 | AKT2/CASP3/CHEK1/EGFR/KIT/MAPK8/MDM2/PARP1/PIK3R1 | 9 |
| GO:0048010 | biological process | vascular endothelial growth factor receptor signaling pathway | 6/59 | 98/18866 | 6.07E-07 | 8.99E-06 | 3.85E-06 | HSP90AA1/KDR/MAPK14/PIK3R1/RAC1/SRC | 6 |
| GO:0007050 | biological process | cell cycle arrest | 8/59 | 234/18866 | 6.39E-07 | 9.42E-06 | 4.03E-06 | AKT2/AURKA/CDK2/HRAS/MAP2K1/MDM2/TGFB2/TGFBR1 | 8 |
| GO:0050867 | biological process | positive regulation of cell activation | 10/59 | 421/18866 | 6.51E-07 | 9.55E-06 | 4.09E-06 | BST1/IGF1/IL2/JAK2/JAK3/PIK3R1/PTPN11/RAC1/RARA/SRC | 10 |
| GO:1903039 | biological process | positive regulation of leukocyte cell-cell adhesion | 8/59 | 235/18866 | 6.60E-07 | 9.63E-06 | 4.12E-06 | IGF1/IL2/JAK3/PIK3R1/PTPN11/RAC1/RARA/SRC | 8 |
| GO:0032872 | biological process | regulation of stress-activated MAPK cascade | 8/59 | 237/18866 | 7.04E-07 | 1.02E-05 | 4.37E-06 | BMP2/EGFR/GSTP1/HRAS/IGF1R/MAP2K1/MAPK1/TGFB2 | 8 |
| GO:0007259 | biological process | receptor signaling pathway via JAK-STAT | 7/59 | 164/18866 | 7.70E-07 | 1.11E-05 | 4.76E-06 | ERBB4/IGF1/IL2/JAK2/JAK3/KIT/STAT1 | 7 |
| GO:0070302 | biological process | regulation of stress-activated protein kinase signaling cascade | 8/59 | 240/18866 | 7.74E-07 | 1.11E-05 | 4.76E-06 | BMP2/EGFR/GSTP1/HRAS/IGF1R/MAP2K1/MAPK1/TGFB2 | 8 |
| GO:1903037 | biological process | regulation of leukocyte cell-cell adhesion | 9/59 | 329/18866 | 7.81E-07 | 1.12E-05 | 4.78E-06 | CASP3/IGF1/IL2/JAK3/PIK3R1/PTPN11/RAC1/RARA/SRC | 9 |
| GO:0038093 | biological process | Fc receptor signaling pathway | 8/59 | 241/18866 | 7.98E-07 | 1.14E-05 | 4.87E-06 | HSP90AA1/ITK/KIT/MAPK1/MAPK8/PIK3R1/RAC1/SRC | 8 |
| GO:0050863 | biological process | regulation of T cell activation | 9/59 | 332/18866 | 8.42E-07 | 1.19E-05 | 5.11E-06 | CASP3/IGF1/IL2/JAK3/PIK3R1/PTPN11/RAC1/RARA/SRC | 9 |
| GO:1904645 | biological process | response to amyloid-beta | 5/59 | 56/18866 | 8.51E-07 | 1.20E-05 | 5.14E-06 | IGF1/IGF1R/MMP2/MMP9/PARP1 | 5 |
| GO:0032496 | biological process | response to lipopolysaccharide | 9/59 | 334/18866 | 8.85E-07 | 1.24E-05 | 5.32E-06 | CASP3/FGFR2/GSTP1/JAK2/MAPK1/MAPK14/MAPK8/RARA/SRC | 9 |
| GO:0045931 | biological process | positive regulation of mitotic cell cycle | 7/59 | 168/18866 | 9.05E-07 | 1.27E-05 | 5.42E-06 | AURKA/EGFR/FGFR1/IGF1/INSR/MDM2/PTPN11 | 7 |
| GO:0001541 | biological process | ovarian follicle development | 5/59 | 57/18866 | 9.30E-07 | 1.29E-05 | 5.52E-06 | BCL2L1/CTNNA1/ESR1/KIT/SRC | 5 |
| GO:0030520 | biological process | intracellular estrogen receptor signaling pathway | 5/59 | 57/18866 | 9.30E-07 | 1.29E-05 | 5.52E-06 | AR/ESR1/ESR2/PARP1/SRC | 5 |
| GO:0051047 | biological process | positive regulation of secretion | 9/59 | 340/18866 | 1.03E-06 | 1.42E-05 | 6.06E-06 | EGFR/FGFR1/IGF1/IL2/JAK2/PTPN11/RAC1/SRC/TGFB2 | 9 |
| GO:0050714 | biological process | positive regulation of protein secretion | 7/59 | 172/18866 | 1.06E-06 | 1.46E-05 | 6.23E-06 | EGFR/IGF1/IL2/JAK2/RAC1/SRC/TGFB2 | 7 |
| GO:0007596 | biological process | blood coagulation | 9/59 | 343/18866 | 1.10E-06 | 1.51E-05 | 6.46E-06 | ANXA5/JAK2/MAPK1/PIK3CG/PIK3R1/PLAU/PTPN11/RAC1/SRC | 9 |
| GO:0097696 | biological process | receptor signaling pathway via STAT | 7/59 | 174/18866 | 1.15E-06 | 1.56E-05 | 6.68E-06 | ERBB4/IGF1/IL2/JAK2/JAK3/KIT/STAT1 | 7 |
| GO:0007265 | biological process | Ras protein signal transduction | 9/59 | 346/18866 | 1.19E-06 | 1.60E-05 | 6.87E-06 | CCNA2/CDK2/HRAS/IGF1/JAK2/MAPK14/MET/RAC1/TGFB2 | 9 |
| GO:0050680 | biological process | negative regulation of epithelial cell proliferation | 7/59 | 175/18866 | 1.19E-06 | 1.60E-05 | 6.87E-06 | AR/FGFR2/PPARG/STAT1/TGFB2/TGFBR1/VDR | 7 |
| GO:0032680 | biological process | regulation of tumor necrosis factor production | 6/59 | 110/18866 | 1.20E-06 | 1.60E-05 | 6.87E-06 | GSTP1/IGF1/JAK2/PIK3R1/PTPN11/RARA | 6 |
| GO:1904659 | biological process | glucose transmembrane transport | 6/59 | 110/18866 | 1.20E-06 | 1.60E-05 | 6.87E-06 | AKT2/IGF1/INSR/MAPK14/PIK3R1/PTPN11 | 6 |
| GO:0043388 | biological process | positive regulation of DNA binding | 5/59 | 60/18866 | 1.20E-06 | 1.60E-05 | 6.87E-06 | IGF1/JAK2/MMP9/PARP1/PPARG | 5 |
| GO:0048568 | biological process | embryonic organ development | 10/59 | 451/18866 | 1.22E-06 | 1.61E-05 | 6.90E-06 | EGFR/FGFR1/FGFR2/KDR/KIT/MAP2K1/MAPK1/RARA/TGFB2/TGFBR1 | 10 |
| GO:0007599 | biological process | hemostasis | 9/59 | 348/18866 | 1.24E-06 | 1.64E-05 | 7.03E-06 | ANXA5/JAK2/MAPK1/PIK3CG/PIK3R1/PLAU/PTPN11/RAC1/SRC | 9 |
| GO:0051149 | biological process | positive regulation of muscle cell differentiation | 6/59 | 111/18866 | 1.27E-06 | 1.66E-05 | 7.12E-06 | CTNNA1/IGF1/KIT/MAPK14/MDM2/SOD2 | 6 |
| GO:0050817 | biological process | coagulation | 9/59 | 349/18866 | 1.27E-06 | 1.67E-05 | 7.13E-06 | ANXA5/JAK2/MAPK1/PIK3CG/PIK3R1/PLAU/PTPN11/RAC1/SRC | 9 |
| GO:0006275 | biological process | regulation of DNA replication | 6/59 | 112/18866 | 1.33E-06 | 1.72E-05 | 7.38E-06 | CCNA2/CDK2/EGFR/FGFR1/HRAS/RAC1 | 6 |
| GO:0046822 | biological process | regulation of nucleocytoplasmic transport | 6/59 | 112/18866 | 1.33E-06 | 1.72E-05 | 7.38E-06 | JAK2/MAPK1/MAPK14/MDM2/PIK3R1/PTPN11 | 6 |
| GO:0048771 | biological process | tissue remodeling | 7/59 | 178/18866 | 1.33E-06 | 1.72E-05 | 7.38E-06 | EGFR/IL2/MDM2/MMP2/RAC1/SRC/VDR | 7 |
| GO:0048638 | biological process | regulation of developmental growth | 9/59 | 353/18866 | 1.40E-06 | 1.80E-05 | 7.71E-06 | AR/ERBB4/FGFR1/FGFR2/IGF1/INSR/MAPK1/MAPK14/TGFBR1 | 9 |
| GO:1903555 | biological process | regulation of tumor necrosis factor superfamily cytokine production | 6/59 | 113/18866 | 1.41E-06 | 1.80E-05 | 7.71E-06 | GSTP1/IGF1/JAK2/PIK3R1/PTPN11/RARA | 6 |
| GO:0008645 | biological process | hexose transmembrane transport | 6/59 | 114/18866 | 1.48E-06 | 1.89E-05 | 8.08E-06 | AKT2/IGF1/INSR/MAPK14/PIK3R1/PTPN11 | 6 |
| GO:0002237 | biological process | response to molecule of bacterial origin | 9/59 | 356/18866 | 1.50E-06 | 1.91E-05 | 8.16E-06 | CASP3/FGFR2/GSTP1/JAK2/MAPK1/MAPK14/MAPK8/RARA/SRC | 9 |
| GO:0051251 | biological process | positive regulation of lymphocyte activation | 9/59 | 357/18866 | 1.54E-06 | 1.94E-05 | 8.32E-06 | BST1/IGF1/IL2/JAK3/PIK3R1/PTPN11/RAC1/RARA/SRC | 9 |
| GO:0015749 | biological process | monosaccharide transmembrane transport | 6/59 | 116/18866 | 1.64E-06 | 2.06E-05 | 8.80E-06 | AKT2/IGF1/INSR/MAPK14/PIK3R1/PTPN11 | 6 |
| GO:0032640 | biological process | tumor necrosis factor production | 6/59 | 116/18866 | 1.64E-06 | 2.06E-05 | 8.80E-06 | GSTP1/IGF1/JAK2/PIK3R1/PTPN11/RARA | 6 |
| GO:0046686 | biological process | response to cadmium ion | 5/59 | 64/18866 | 1.67E-06 | 2.07E-05 | 8.87E-06 | EGFR/KIT/MAPK1/MAPK8/MMP9 | 5 |
| GO:0060135 | biological process | maternal process involved in female pregnancy | 5/59 | 64/18866 | 1.67E-06 | 2.07E-05 | 8.87E-06 | AR/ESR1/MAPK1/PGR/VDR | 5 |
| GO:0007159 | biological process | leukocyte cell-cell adhesion | 9/59 | 364/18866 | 1.80E-06 | 2.23E-05 | 9.56E-06 | CASP3/IGF1/IL2/JAK3/PIK3R1/PTPN11/RAC1/RARA/SRC | 9 |
| GO:0034219 | biological process | carbohydrate transmembrane transport | 6/59 | 118/18866 | 1.81E-06 | 2.24E-05 | 9.57E-06 | AKT2/IGF1/INSR/MAPK14/PIK3R1/PTPN11 | 6 |
| GO:0051052 | biological process | regulation of DNA metabolic process | 9/59 | 365/18866 | 1.84E-06 | 2.27E-05 | 9.70E-06 | CDK2/CHEK1/EGFR/HSP90AA1/IL2/MAPK1/PARP1/PPARG/SRC | 9 |
| GO:0048639 | biological process | positive regulation of developmental growth | 7/59 | 187/18866 | 1.85E-06 | 2.27E-05 | 9.72E-06 | ERBB4/FGFR1/FGFR2/IGF1/INSR/MAPK1/MAPK14 | 7 |
| GO:0071706 | biological process | tumor necrosis factor superfamily cytokine production | 6/59 | 119/18866 | 1.90E-06 | 2.32E-05 | 9.93E-06 | GSTP1/IGF1/JAK2/PIK3R1/PTPN11/RARA | 6 |
| GO:0046824 | biological process | positive regulation of nucleocytoplasmic transport | 5/59 | 66/18866 | 1.94E-06 | 2.36E-05 | 1.01E-05 | JAK2/MAPK1/MAPK14/MDM2/PIK3R1 | 5 |
| GO:0006367 | biological process | transcription initiation from RNA polymerase II promoter | 7/59 | 189/18866 | 1.99E-06 | 2.41E-05 | 1.03E-05 | AR/ESR1/ESR2/PGR/PPARG/RARA/VDR | 7 |
| GO:0002065 | biological process | columnar/cuboidal epithelial cell differentiation | 6/59 | 121/18866 | 2.10E-06 | 2.52E-05 | 1.08E-05 | BMP2/FGFR1/FGFR2/RARA/SRC/TYMS | 6 |
| GO:0030218 | biological process | erythrocyte differentiation | 6/59 | 121/18866 | 2.10E-06 | 2.52E-05 | 1.08E-05 | CASP3/JAK2/JAK3/KIT/MAPK14/STAT1 | 6 |
| GO:0002793 | biological process | positive regulation of peptide secretion | 7/59 | 193/18866 | 2.29E-06 | 2.74E-05 | 1.17E-05 | EGFR/IGF1/IL2/JAK2/RAC1/SRC/TGFB2 | 7 |
| GO:0022409 | biological process | positive regulation of cell-cell adhesion | 8/59 | 279/18866 | 2.39E-06 | 2.85E-05 | 1.22E-05 | IGF1/IL2/JAK3/PIK3R1/PTPN11/RAC1/RARA/SRC | 8 |
| GO:0051101 | biological process | regulation of DNA binding | 6/59 | 124/18866 | 2.42E-06 | 2.87E-05 | 1.23E-05 | IGF1/JAK2/MAPK8/MMP9/PARP1/PPARG | 6 |
| GO:0050731 | biological process | positive regulation of peptidyl-tyrosine phosphorylation | 7/59 | 195/18866 | 2.45E-06 | 2.89E-05 | 1.23E-05 | ERBB4/IGF1/IL2/JAK2/KIT/PTPN11/SRC | 7 |
| GO:1905475 | biological process | regulation of protein localization to membrane | 7/59 | 195/18866 | 2.45E-06 | 2.89E-05 | 1.23E-05 | AKT2/AR/BCL2L1/EGFR/HRAS/MAPK8/PIK3R1 | 7 |
| GO:0001952 | biological process | regulation of cell-matrix adhesion | 6/59 | 125/18866 | 2.54E-06 | 2.96E-05 | 1.27E-05 | BST1/KDR/PIK3R1/PLAU/RAC1/SRC | 6 |
| GO:0008637 | biological process | apoptotic mitochondrial changes | 6/59 | 125/18866 | 2.54E-06 | 2.96E-05 | 1.27E-05 | BCL2L1/ERBB4/IGF1/MAPK8/MMP9/SOD2 | 6 |
| GO:0051216 | biological process | cartilage development | 7/59 | 197/18866 | 2.62E-06 | 3.05E-05 | 1.31E-05 | BMP2/FGFR1/MAPK14/PTPN11/RARA/TGFBR1/TYMS | 7 |
| GO:0001822 | biological process | kidney development | 8/59 | 283/18866 | 2.65E-06 | 3.08E-05 | 1.32E-05 | BMP2/FGFR1/FGFR2/MMP9/RARA/STAT1/TGFB2/TGFBR1 | 8 |
| GO:0071621 | biological process | granulocyte chemotaxis | 6/59 | 127/18866 | 2.78E-06 | 3.20E-05 | 1.37E-05 | BST1/MAPK1/MAPK14/PIK3CG/RAC1/TGFB2 | 6 |
| GO:1900180 | biological process | regulation of protein localization to nucleus | 6/59 | 127/18866 | 2.78E-06 | 3.20E-05 | 1.37E-05 | JAK2/MAPK1/MAPK14/PARP1/PIK3R1/SRC | 6 |
| GO:0042531 | biological process | positive regulation of tyrosine phosphorylation of STAT protein | 5/59 | 71/18866 | 2.80E-06 | 3.21E-05 | 1.37E-05 | ERBB4/IGF1/IL2/JAK2/KIT | 5 |
| GO:0051054 | biological process | positive regulation of DNA metabolic process | 7/59 | 200/18866 | 2.90E-06 | 3.31E-05 | 1.42E-05 | CDK2/EGFR/HSP90AA1/IL2/MAPK1/PARP1/SRC | 7 |
| GO:0034101 | biological process | erythrocyte homeostasis | 6/59 | 129/18866 | 3.04E-06 | 3.47E-05 | 1.48E-05 | CASP3/JAK2/JAK3/KIT/MAPK14/STAT1 | 6 |
| GO:0060348 | biological process | bone development | 7/59 | 204/18866 | 3.30E-06 | 3.73E-05 | 1.60E-05 | BMP2/FGFR2/IGF1/KIT/PTPN11/RARA/SRC | 7 |
| GO:0060541 | biological process | respiratory system development | 7/59 | 204/18866 | 3.30E-06 | 3.73E-05 | 1.60E-05 | EGFR/FGFR1/FGFR2/MAP2K1/MAPK1/PGR/RARA | 7 |
| GO:0072001 | biological process | renal system development | 8/59 | 292/18866 | 3.35E-06 | 3.77E-05 | 1.61E-05 | BMP2/FGFR1/FGFR2/MMP9/RARA/STAT1/TGFB2/TGFBR1 | 8 |
| GO:0051881 | biological process | regulation of mitochondrial membrane potential | 5/59 | 74/18866 | 3.44E-06 | 3.85E-05 | 1.65E-05 | AKT2/BCL2L1/KDR/PARP1/SRC | 5 |
| GO:0070482 | biological process | response to oxygen levels | 9/59 | 396/18866 | 3.59E-06 | 4.01E-05 | 1.72E-05 | BMP2/CASP3/CCNA2/MDM2/MMP2/PLAU/PPARG/SRC/TGFB2 | 9 |
| GO:0038034 | biological process | signal transduction in absence of ligand | 5/59 | 75/18866 | 3.67E-06 | 4.07E-05 | 1.74E-05 | BCL2L1/CASP3/CTNNA1/FGFR1/IL2 | 5 |
| GO:0097192 | biological process | extrinsic apoptotic signaling pathway in absence of ligand | 5/59 | 75/18866 | 3.67E-06 | 4.07E-05 | 1.74E-05 | BCL2L1/CASP3/CTNNA1/FGFR1/IL2 | 5 |
| GO:0070102 | biological process | interleukin-6-mediated signaling pathway | 4/59 | 34/18866 | 3.73E-06 | 4.12E-05 | 1.76E-05 | JAK2/PTPN11/SRC/STAT1 | 4 |
| GO:0048565 | biological process | digestive tract development | 6/59 | 135/18866 | 3.96E-06 | 4.36E-05 | 1.87E-05 | EGFR/FGFR2/KIT/SRC/TGFB2/TYMS | 6 |
| GO:0043393 | biological process | regulation of protein binding | 7/59 | 211/18866 | 4.13E-06 | 4.53E-05 | 1.94E-05 | AURKA/BMP2/MAPK8/MET/MMP9/SRC/TGFBR1 | 7 |
| GO:0060411 | biological process | cardiac septum morphogenesis | 5/59 | 77/18866 | 4.18E-06 | 4.53E-05 | 1.94E-05 | FGFR2/MDM2/RARA/TGFB2/TGFBR1 | 5 |
| GO:0007162 | biological process | negative regulation of cell adhesion | 8/59 | 301/18866 | 4.19E-06 | 4.53E-05 | 1.94E-05 | BMP2/CASP3/IL2/JAK2/JAK3/PIK3R1/PTPN11/SRC | 8 |
| GO:0003203 | biological process | endocardial cushion morphogenesis | 4/59 | 35/18866 | 4.20E-06 | 4.53E-05 | 1.94E-05 | BMP2/MDM2/TGFB2/TGFBR1 | 4 |
| GO:0007435 | biological process | salivary gland morphogenesis | 4/59 | 35/18866 | 4.20E-06 | 4.53E-05 | 1.94E-05 | EGFR/FGFR1/FGFR2/TGFB2 | 4 |
| GO:0071312 | biological process | cellular response to alkaloid | 4/59 | 35/18866 | 4.20E-06 | 4.53E-05 | 1.94E-05 | BCL2L1/CASP3/CCNA2/MDM2 | 4 |
| GO:0030278 | biological process | regulation of ossification | 7/59 | 212/18866 | 4.26E-06 | 4.57E-05 | 1.96E-05 | BMP2/FGFR2/IGF1/MAPK1/MAPK14/PTPN11/TGFB2 | 7 |
| GO:0002696 | biological process | positive regulation of leukocyte activation | 9/59 | 406/18866 | 4.40E-06 | 4.70E-05 | 2.01E-05 | BST1/IGF1/IL2/JAK3/PIK3R1/PTPN11/RAC1/RARA/SRC | 9 |
| GO:0051057 | biological process | positive regulation of small GTPase mediated signal transduction | 5/59 | 78/18866 | 4.46E-06 | 4.74E-05 | 2.03E-05 | HRAS/IGF1/JAK2/RAC1/SRC | 5 |
| GO:0060395 | biological process | SMAD protein signal transduction | 5/59 | 78/18866 | 4.46E-06 | 4.74E-05 | 2.03E-05 | BMP2/JAK2/PARP1/TGFB2/TGFBR1 | 5 |
| GO:0007517 | biological process | muscle organ development | 9/59 | 407/18866 | 4.48E-06 | 4.75E-05 | 2.03E-05 | BMP2/ERBB4/FGFR1/FGFR2/IGF1/MAPK1/MAPK14/TGFB2/TGFBR1 | 9 |
| GO:0010092 | biological process | specification of animal organ identity | 4/59 | 36/18866 | 4.72E-06 | 4.98E-05 | 2.13E-05 | AR/BMP2/FGFR1/FGFR2 | 4 |
| GO:0071260 | biological process | cellular response to mechanical stimulus | 5/59 | 79/18866 | 4.75E-06 | 5.00E-05 | 2.14E-05 | CHEK1/EGFR/MAPK8/PTPN11/RAC1 | 5 |
| GO:0001503 | biological process | ossification | 9/59 | 412/18866 | 4.95E-06 | 5.19E-05 | 2.22E-05 | BMP2/EGFR/FGFR2/IGF1/MAPK1/MAPK14/MMP2/PTPN11/TGFB2 | 9 |
| GO:0010810 | biological process | regulation of cell-substrate adhesion | 7/59 | 217/18866 | 4.97E-06 | 5.19E-05 | 2.22E-05 | BST1/JAK2/KDR/PIK3R1/PLAU/RAC1/SRC | 7 |
| GO:0001889 | biological process | liver development | 6/59 | 141/18866 | 5.10E-06 | 5.30E-05 | 2.27E-05 | AURKA/EGFR/HRAS/MET/RARA/TYMS | 6 |
| GO:0046425 | biological process | regulation of receptor signaling pathway via JAK-STAT | 6/59 | 142/18866 | 5.31E-06 | 5.51E-05 | 2.36E-05 | ERBB4/IGF1/IL2/JAK2/JAK3/KIT | 6 |
| GO:0008286 | biological process | insulin receptor signaling pathway | 6/59 | 143/18866 | 5.53E-06 | 5.71E-05 | 2.45E-05 | AKT2/IGF1R/INSR/PIK3R1/PTPN11/SRC | 6 |
| GO:0071241 | biological process | cellular response to inorganic substance | 7/59 | 221/18866 | 5.60E-06 | 5.77E-05 | 2.47E-05 | CCNA2/CDK2/EGFR/MAPK1/MAPK8/MMP9/PARP1 | 7 |
| GO:0010212 | biological process | response to ionizing radiation | 6/59 | 144/18866 | 5.75E-06 | 5.86E-05 | 2.51E-05 | BCL2L1/CASP3/HRAS/MAPK14/MDM2/PARP1 | 6 |
| GO:0050921 | biological process | positive regulation of chemotaxis | 6/59 | 144/18866 | 5.75E-06 | 5.86E-05 | 2.51E-05 | FGFR1/KDR/MAPK1/MAPK14/MET/RAC1 | 6 |
| GO:0061008 | biological process | hepaticobiliary system development | 6/59 | 144/18866 | 5.75E-06 | 5.86E-05 | 2.51E-05 | AURKA/EGFR/HRAS/MET/RARA/TYMS | 6 |
| GO:0097529 | biological process | myeloid leukocyte migration | 7/59 | 222/18866 | 5.77E-06 | 5.86E-05 | 2.51E-05 | BST1/KIT/MAPK1/MAPK14/PIK3CG/RAC1/TGFB2 | 7 |
| GO:0071276 | biological process | cellular response to cadmium ion | 4/59 | 38/18866 | 5.88E-06 | 5.94E-05 | 2.54E-05 | EGFR/MAPK1/MAPK8/MMP9 | 4 |
| GO:0071392 | biological process | cellular response to estradiol stimulus | 4/59 | 38/18866 | 5.88E-06 | 5.94E-05 | 2.54E-05 | CCNA2/EGFR/ESR1/ESR2 | 4 |
| GO:0048708 | biological process | astrocyte differentiation | 5/59 | 83/18866 | 6.06E-06 | 6.10E-05 | 2.61E-05 | BMP2/EGFR/MAP2K1/MAPK1/PTPN11 | 5 |
| GO:0043583 | biological process | ear development | 7/59 | 224/18866 | 6.12E-06 | 6.14E-05 | 2.63E-05 | BMP2/CCNA2/FGFR1/FGFR2/MAPK1/PTPN11/TGFB2 | 7 |
| GO:0060736 | biological process | prostate gland growth | 3/59 | 12/18866 | 6.26E-06 | 6.26E-05 | 2.68E-05 | AR/ESR1/FGFR2 | 3 |
| GO:0007568 | biological process | aging | 8/59 | 319/18866 | 6.41E-06 | 6.39E-05 | 2.74E-05 | CHEK1/CTNNA1/HRAS/MAP2K1/MAPK1/MAPK14/SOD2/TYMS | 8 |
| GO:0007431 | biological process | salivary gland development | 4/59 | 39/18866 | 6.54E-06 | 6.47E-05 | 2.77E-05 | EGFR/FGFR1/FGFR2/TGFB2 | 4 |
| GO:0033146 | biological process | regulation of intracellular estrogen receptor signaling pathway | 4/59 | 39/18866 | 6.54E-06 | 6.47E-05 | 2.77E-05 | AR/ESR1/PARP1/SRC | 4 |
| GO:1901214 | biological process | regulation of neuron death | 8/59 | 321/18866 | 6.71E-06 | 6.63E-05 | 2.84E-05 | BCL2L1/CASP3/HRAS/JAK2/NQO2/PARP1/SOD2/TGFB2 | 8 |
| GO:0014910 | biological process | regulation of smooth muscle cell migration | 5/59 | 86/18866 | 7.22E-06 | 7.08E-05 | 3.03E-05 | GSTP1/IGF1/MDM2/PLAU/SRC | 5 |
| GO:0042509 | biological process | regulation of tyrosine phosphorylation of STAT protein | 5/59 | 86/18866 | 7.22E-06 | 7.08E-05 | 3.03E-05 | ERBB4/IGF1/IL2/JAK2/KIT | 5 |
| GO:0042307 | biological process | positive regulation of protein import into nucleus | 4/59 | 40/18866 | 7.25E-06 | 7.08E-05 | 3.03E-05 | JAK2/MAPK1/MAPK14/PIK3R1 | 4 |
| GO:0097530 | biological process | granulocyte migration | 6/59 | 150/18866 | 7.28E-06 | 7.09E-05 | 3.03E-05 | BST1/MAPK1/MAPK14/PIK3CG/RAC1/TGFB2 | 6 |
| GO:0000077 | biological process | DNA damage checkpoint | 6/59 | 151/18866 | 7.56E-06 | 7.29E-05 | 3.12E-05 | AURKA/CDK2/CHEK1/MAPK14/MDM2/PTPN11 | 6 |
| GO:0008643 | biological process | carbohydrate transport | 6/59 | 151/18866 | 7.56E-06 | 7.29E-05 | 3.12E-05 | AKT2/IGF1/INSR/MAPK14/PIK3R1/PTPN11 | 6 |
| GO:1904892 | biological process | regulation of receptor signaling pathway via STAT | 6/59 | 151/18866 | 7.56E-06 | 7.29E-05 | 3.12E-05 | ERBB4/IGF1/IL2/JAK2/JAK3/KIT | 6 |
| GO:0030595 | biological process | leukocyte chemotaxis | 7/59 | 232/18866 | 7.69E-06 | 7.40E-05 | 3.17E-05 | BST1/KIT/MAPK1/MAPK14/PIK3CG/RAC1/TGFB2 | 7 |
| GO:0002262 | biological process | myeloid cell homeostasis | 6/59 | 154/18866 | 8.46E-06 | 8.11E-05 | 3.47E-05 | CASP3/JAK2/JAK3/KIT/MAPK14/STAT1 | 6 |
| GO:0010907 | biological process | positive regulation of glucose metabolic process | 4/59 | 42/18866 | 8.83E-06 | 8.42E-05 | 3.60E-05 | AKT2/IGF1/INSR/SRC | 4 |
| GO:1904591 | biological process | positive regulation of protein import | 4/59 | 42/18866 | 8.83E-06 | 8.42E-05 | 3.60E-05 | JAK2/MAPK1/MAPK14/PIK3R1 | 4 |
| GO:0046427 | biological process | positive regulation of receptor signaling pathway via JAK-STAT | 5/59 | 90/18866 | 9.02E-06 | 8.57E-05 | 3.67E-05 | ERBB4/IGF1/IL2/JAK2/KIT | 5 |
| GO:0001819 | biological process | positive regulation of cytokine production | 9/59 | 447/18866 | 9.54E-06 | 9.04E-05 | 3.87E-05 | HRAS/IL2/JAK2/MAPK14/PIK3R1/PTPN11/RARA/SRC/STAT1 | 9 |
| GO:0070663 | biological process | regulation of leukocyte proliferation | 7/59 | 240/18866 | 9.60E-06 | 9.06E-05 | 3.88E-05 | BST1/CASP3/GSTP1/IGF1/IL2/JAK3/MAPK1 | 7 |
| GO:0071364 | biological process | cellular response to epidermal growth factor stimulus | 4/59 | 43/18866 | 9.72E-06 | 9.15E-05 | 3.92E-05 | EGFR/ERBB4/GSTP1/PTPN11 | 4 |
| GO:0035051 | biological process | cardiocyte differentiation | 6/59 | 158/18866 | 9.80E-06 | 9.19E-05 | 3.93E-05 | BMP2/EGFR/IGF1/MAPK1/RARA/TGFB2 | 6 |
| GO:0014909 | biological process | smooth muscle cell migration | 5/59 | 93/18866 | 1.06E-05 | 9.88E-05 | 4.23E-05 | GSTP1/IGF1/MDM2/PLAU/SRC | 5 |
| GO:1904894 | biological process | positive regulation of receptor signaling pathway via STAT | 5/59 | 93/18866 | 1.06E-05 | 9.88E-05 | 4.23E-05 | ERBB4/IGF1/IL2/JAK2/KIT | 5 |
| GO:0010463 | biological process | mesenchymal cell proliferation | 4/59 | 44/18866 | 1.07E-05 | 9.89E-05 | 4.23E-05 | BMP2/FGFR1/FGFR2/STAT1 | 4 |
| GO:0017145 | biological process | stem cell division | 4/59 | 44/18866 | 1.07E-05 | 9.89E-05 | 4.23E-05 | FGFR1/FGFR2/KIT/TGFB2 | 4 |
| GO:0031570 | biological process | DNA integrity checkpoint | 6/59 | 161/18866 | 1.09E-05 | 1.01E-04 | 4.31E-05 | AURKA/CDK2/CHEK1/MAPK14/MDM2/PTPN11 | 6 |
| GO:0051090 | biological process | regulation of DNA-binding transcription factor activity | 9/59 | 455/18866 | 1.10E-05 | 1.01E-04 | 4.34E-05 | AR/ESR1/ESR2/JAK2/KIT/MAPK1/MAPK14/MAPK8/PPARG | 9 |
| GO:0062012 | biological process | regulation of small molecule metabolic process | 9/59 | 456/18866 | 1.12E-05 | 1.03E-04 | 4.40E-05 | AKT2/BMP2/IGF1/INSR/KIT/PARP1/PPARG/SRC/VDR | 9 |
| GO:0003197 | biological process | endocardial cushion development | 4/59 | 45/18866 | 1.17E-05 | 1.07E-04 | 4.58E-05 | BMP2/MDM2/TGFB2/TGFBR1 | 4 |
| GO:0031960 | biological process | response to corticosteroid | 6/59 | 164/18866 | 1.21E-05 | 1.11E-04 | 4.74E-05 | CASP3/EGFR/GSTP1/PARP1/SRC/TYMS | 6 |
| GO:0006352 | biological process | DNA-templated transcription, initiation | 7/59 | 249/18866 | 1.22E-05 | 1.11E-04 | 4.75E-05 | AR/ESR1/ESR2/PGR/PPARG/RARA/VDR | 7 |
| GO:0009636 | biological process | response to toxic substance | 7/59 | 250/18866 | 1.25E-05 | 1.14E-04 | 4.86E-05 | ALB/GSTM1/GSTP1/MAPK1/MDM2/SOD2/TYMS | 7 |
| GO:0060391 | biological process | positive regulation of SMAD protein signal transduction | 3/59 | 15/18866 | 1.29E-05 | 1.16E-04 | 4.97E-05 | JAK2/PARP1/TGFBR1 | 3 |
| GO:2001028 | biological process | positive regulation of endothelial cell chemotaxis | 3/59 | 15/18866 | 1.29E-05 | 0.000116137 | 4.97E-05 | FGFR1/KDR/MET | 3 |
| GO:2001242 | biological process | regulation of intrinsic apoptotic signaling pathway | 6/59 | 166/18866 | 1.30E-05 | 0.000116841 | 5.00E-05 | BCL2L1/MDM2/MMP9/PARP1/SOD2/SRC | 6 |
| GO:0050708 | biological process | regulation of protein secretion | 8/59 | 352/18866 | 1.31E-05 | 0.000117729 | 5.04E-05 | EGFR/IGF1/IL2/JAK2/PTPN11/RAC1/SRC/TGFB2 | 8 |
| GO:0031668 | biological process | cellular response to extracellular stimulus | 7/59 | 253/18866 | 1.35E-05 | 0.000120895 | 5.17E-05 | ALB/GSTP1/MAPK1/MAPK8/MDM2/PPARG/VDR | 7 |
| GO:2001243 | biological process | negative regulation of intrinsic apoptotic signaling pathway | 5/59 | 98/18866 | 1.37E-05 | 0.000122005 | 5.22E-05 | BCL2L1/MDM2/MMP9/SOD2/SRC | 5 |
| GO:0046677 | biological process | response to antibiotic | 4/59 | 47/18866 | 1.39E-05 | 0.000123741 | 5.30E-05 | CASP3/HSP90AA1/JAK2/MDM2 | 4 |
| GO:2000241 | biological process | regulation of reproductive process | 6/59 | 170/18866 | 1.49E-05 | 0.000131838 | 5.64E-05 | AR/AURKA/ESR1/IGF1/INSR/SRC | 6 |
| GO:0001666 | biological process | response to hypoxia | 8/59 | 359/18866 | 1.51E-05 | 0.000132741 | 5.68E-05 | BMP2/CASP3/CCNA2/MDM2/MMP2/PLAU/SRC/TGFB2 | 8 |
| GO:0007178 | biological process | transmembrane receptor protein serine/threonine kinase signaling pathway | 8/59 | 359/18866 | 1.51E-05 | 0.000132741 | 5.68E-05 | BMP2/JAK2/MAPK14/PARP1/SRC/TGFB2/TGFBR1/XIAP | 8 |
| GO:0033628 | biological process | regulation of cell adhesion mediated by integrin | 4/59 | 48/18866 | 1.51E-05 | 0.000132741 | 5.68E-05 | PIK3CG/PLAU/PTPN11/TGFB2 | 4 |
| GO:0051972 | biological process | regulation of telomerase activity | 4/59 | 48/18866 | 1.51E-05 | 0.000132741 | 5.68E-05 | HSP90AA1/MAPK1/PPARG/SRC | 4 |
| GO:0003007 | biological process | heart morphogenesis | 7/59 | 258/18866 | 1.53E-05 | 0.000134057 | 5.74E-05 | BMP2/FGFR2/INSR/MDM2/RARA/TGFB2/TGFBR1 | 7 |
| GO:0045725 | biological process | positive regulation of glycogen biosynthetic process | 3/59 | 16/18866 | 1.58E-05 | 1.37E-04 | 5.88E-05 | AKT2/IGF1/INSR | 3 |
| GO:0051770 | biological process | positive regulation of nitric-oxide synthase biosynthetic process | 3/59 | 16/18866 | 1.58E-05 | 1.37E-04 | 5.88E-05 | JAK2/KDR/STAT1 | 3 |
| GO:0030850 | biological process | prostate gland development | 4/59 | 49/18866 | 1.65E-05 | 1.43E-04 | 6.10E-05 | AR/ESR1/FGFR2/RARA | 4 |
| GO:0061448 | biological process | connective tissue development | 7/59 | 262/18866 | 1.69E-05 | 1.46E-04 | 6.26E-05 | BMP2/FGFR1/MAPK14/PTPN11/RARA/TGFBR1/TYMS | 7 |
| GO:0010038 | biological process | response to metal ion | 8/59 | 366/18866 | 1.74E-05 | 1.50E-04 | 6.41E-05 | CASP3/EGFR/KIT/MAPK1/MAPK8/MDM2/MMP9/PARP1 | 8 |
| GO:0010634 | biological process | positive regulation of epithelial cell migration | 6/59 | 176/18866 | 1.81E-05 | 1.56E-04 | 6.66E-05 | FGFR1/KDR/MET/MMP9/SRC/TGFB2 | 6 |
| GO:0043279 | biological process | response to alkaloid | 5/59 | 104/18866 | 1.83E-05 | 1.56E-04 | 6.70E-05 | BCL2L1/CASP3/CCNA2/MDM2/PPARG | 5 |
| GO:0030324 | biological process | lung development | 6/59 | 177/18866 | 1.87E-05 | 1.60E-04 | 6.83E-05 | EGFR/FGFR1/FGFR2/MAP2K1/MAPK1/PGR | 6 |
| GO:0070875 | biological process | positive regulation of glycogen metabolic process | 3/59 | 17/18866 | 1.91E-05 | 1.63E-04 | 6.97E-05 | AKT2/IGF1/INSR | 3 |
| GO:0036293 | biological process | response to decreased oxygen levels | 8/59 | 371/18866 | 1.92E-05 | 1.63E-04 | 6.97E-05 | BMP2/CASP3/CCNA2/MDM2/MMP2/PLAU/SRC/TGFB2 | 8 |
| GO:0048146 | biological process | positive regulation of fibroblast proliferation | 4/59 | 51/18866 | 1.93E-05 | 1.63E-04 | 6.98E-05 | CCNA2/EGFR/ESR1/IGF1 | 4 |
| GO:0071354 | biological process | cellular response to interleukin-6 | 4/59 | 51/18866 | 1.93E-05 | 1.63E-04 | 6.98E-05 | JAK2/PTPN11/SRC/STAT1 | 4 |
| GO:0016049 | biological process | cell growth | 9/59 | 490/18866 | 1.98E-05 | 1.67E-04 | 7.14E-05 | AURKA/EGFR/ESR2/HSP90AA1/IGF1/IL2/PPARG/TGFB2/TGFBR1 | 9 |
| GO:0014812 | biological process | muscle cell migration | 5/59 | 106/18866 | 2.00E-05 | 1.67E-04 | 7.16E-05 | GSTP1/IGF1/MDM2/PLAU/SRC | 5 |
| GO:0030038 | biological process | contractile actin filament bundle assembly | 5/59 | 106/18866 | 2.00E-05 | 1.67E-04 | 7.16E-05 | MET/PIK3R1/RAC1/SRC/TGFBR1 | 5 |
| GO:0043149 | biological process | stress fiber assembly | 5/59 | 106/18866 | 2.00E-05 | 1.67E-04 | 7.16E-05 | MET/PIK3R1/RAC1/SRC/TGFBR1 | 5 |
| GO:0000186 | biological process | activation of MAPKK activity | 4/59 | 52/18866 | 2.09E-05 | 1.74E-04 | 7.43E-05 | EGFR/JAK2/MAPK1/TGFBR1 | 4 |
| GO:0071622 | biological process | regulation of granulocyte chemotaxis | 4/59 | 52/18866 | 2.09E-05 | 1.74E-04 | 7.43E-05 | BST1/MAPK1/MAPK14/RAC1 | 4 |
| GO:0030323 | biological process | respiratory tube development | 6/59 | 181/18866 | 2.12E-05 | 1.76E-04 | 7.52E-05 | EGFR/FGFR1/FGFR2/MAP2K1/MAPK1/PGR | 6 |
| GO:0002062 | biological process | chondrocyte differentiation | 5/59 | 108/18866 | 2.19E-05 | 1.81E-04 | 7.76E-05 | BMP2/FGFR1/MAPK14/PTPN11/TGFBR1 | 5 |
| GO:0006260 | biological process | DNA replication | 7/59 | 273/18866 | 2.21E-05 | 1.82E-04 | 7.79E-05 | CCNA2/CDK2/CHEK1/EGFR/FGFR1/HRAS/RAC1 | 7 |
| GO:2001235 | biological process | positive regulation of apoptotic signaling pathway | 6/59 | 183/18866 | 2.26E-05 | 1.86E-04 | 7.94E-05 | BCL2L1/CTNNA1/JAK2/MAPK8/MMP9/TGFBR1 | 6 |
| GO:2000641 | biological process | regulation of early endosome to late endosome transport | 3/59 | 18/18866 | 2.29E-05 | 1.88E-04 | 8.04E-05 | MAP2K1/MAPK1/SRC | 3 |
| GO:0002791 | biological process | regulation of peptide secretion | 8/59 | 381/18866 | 2.32E-05 | 1.90E-04 | 8.12E-05 | EGFR/IGF1/IL2/JAK2/PTPN11/RAC1/SRC/TGFB2 | 8 |
| GO:0001936 | biological process | regulation of endothelial cell proliferation | 6/59 | 184/18866 | 2.33E-05 | 1.90E-04 | 8.12E-05 | BMP2/FGFR1/KDR/PPARG/STAT1/TGFBR1 | 6 |
| GO:0006909 | biological process | phagocytosis | 8/59 | 382/18866 | 2.36E-05 | 1.92E-04 | 8.22E-05 | HSP90AA1/MAPK1/MET/PIK3R1/PPARG/RAC1/RARA/SRC | 8 |
| GO:0090100 | biological process | positive regulation of transmembrane receptor protein serine/threonine kinase signaling pathway | 5/59 | 110/18866 | 2.40E-05 | 1.94E-04 | 8.32E-05 | BMP2/JAK2/PARP1/TGFB2/TGFBR1 | 5 |
| GO:0034504 | biological process | protein localization to nucleus | 7/59 | 277/18866 | 2.42E-05 | 1.96E-04 | 8.39E-05 | JAK2/MAPK1/MAPK14/MDM2/PARP1/PIK3R1/SRC | 7 |
| GO:0070741 | biological process | response to interleukin-6 | 4/59 | 55/18866 | 2.61E-05 | 2.11E-04 | 9.02E-05 | JAK2/PTPN11/SRC/STAT1 | 4 |
| GO:0033189 | biological process | response to vitamin A | 3/59 | 19/18866 | 2.72E-05 | 2.16E-04 | 9.23E-05 | PPARG/RARA/TYMS | 3 |
| GO:0060438 | biological process | trachea development | 3/59 | 19/18866 | 2.72E-05 | 2.16E-04 | 9.23E-05 | MAP2K1/MAPK1/RARA | 3 |
| GO:0060749 | biological process | mammary gland alveolus development | 3/59 | 19/18866 | 2.72E-05 | 2.16E-04 | 9.23E-05 | AR/ERBB4/ESR1 | 3 |
| GO:0061377 | biological process | mammary gland lobule development | 3/59 | 19/18866 | 2.72E-05 | 2.16E-04 | 9.23E-05 | AR/ERBB4/ESR1 | 3 |
| GO:1905288 | biological process | vascular associated smooth muscle cell apoptotic process | 3/59 | 19/18866 | 2.72E-05 | 2.16E-04 | 9.23E-05 | IGF1/PPARG/SOD2 | 3 |
| GO:1905459 | biological process | regulation of vascular associated smooth muscle cell apoptotic process | 3/59 | 19/18866 | 2.72E-05 | 2.16E-04 | 9.23E-05 | IGF1/PPARG/SOD2 | 3 |
| GO:0030900 | biological process | forebrain development | 8/59 | 391/18866 | 2.79E-05 | 2.21E-04 | 9.44E-05 | BMP2/CASP3/EGFR/ERBB4/FGFR1/FGFR2/RARA/SRC | 8 |
| GO:0010332 | biological process | response to gamma radiation | 4/59 | 56/18866 | 2.81E-05 | 2.21E-04 | 9.44E-05 | BCL2L1/HRAS/MDM2/PARP1 | 4 |
| GO:0031295 | biological process | T cell costimulation | 4/59 | 56/18866 | 2.81E-05 | 2.21E-04 | 9.44E-05 | PIK3R1/PTPN11/RAC1/SRC | 4 |
| GO:0060688 | biological process | regulation of morphogenesis of a branching structure | 4/59 | 56/18866 | 2.81E-05 | 2.21E-04 | 9.44E-05 | AR/ESR1/FGFR1/FGFR2 | 4 |
| GO:0003279 | biological process | cardiac septum development | 5/59 | 114/18866 | 2.85E-05 | 2.23E-04 | 9.53E-05 | FGFR2/MDM2/RARA/TGFB2/TGFBR1 | 5 |
| GO:0043200 | biological process | response to amino acid | 5/59 | 114/18866 | 2.85E-05 | 0.000222628 | 9.53E-05 | BCL2L1/CASP3/EGFR/GSTP1/MMP2 | 5 |
| GO:0031346 | biological process | positive regulation of cell projection organization | 8/59 | 394/18866 | 2.95E-05 | 0.000229833 | 9.84E-05 | FGFR1/HRAS/IL2/KIT/MAP2K1/RAC1/SRC/TGFBR1 | 8 |
| GO:0042493 | biological process | response to drug | 8/59 | 397/18866 | 3.11E-05 | 0.000241958 | 1.04E-04 | CASP3/EGFR/MDM2/PPARG/SRC/STAT1/TGFB2/TYMS | 8 |
| GO:0046578 | biological process | regulation of Ras protein signal transduction | 6/59 | 194/18866 | 3.13E-05 | 0.000243251 | 0.000104101 | HRAS/IGF1/JAK2/MET/RAC1/TGFB2 | 6 |
| GO:0018107 | biological process | peptidyl-threonine phosphorylation | 5/59 | 117/18866 | 3.23E-05 | 0.000248495 | 0.000106345 | CHEK1/MAP2K1/MAPK1/MAPK8/TGFBR1 | 5 |
| GO:0007219 | biological process | Notch signaling pathway | 6/59 | 195/18866 | 3.23E-05 | 0.000248495 | 0.000106345 | BMP2/EGFR/KIT/SRC/STAT1/TGFB2 | 6 |
| GO:0031294 | biological process | lymphocyte costimulation | 4/59 | 58/18866 | 3.23E-05 | 0.000248495 | 0.000106345 | PIK3R1/PTPN11/RAC1/SRC | 4 |
| GO:0048839 | biological process | inner ear development | 6/59 | 197/18866 | 3.42E-05 | 0.000262438 | 0.000112312 | BMP2/CCNA2/FGFR1/FGFR2/PTPN11/TGFB2 | 6 |
| GO:0042306 | biological process | regulation of protein import into nucleus | 4/59 | 59/18866 | 3.45E-05 | 0.00026461 | 0.000113241 | JAK2/MAPK1/MAPK14/PIK3R1 | 4 |
| GO:0001935 | biological process | endothelial cell proliferation | 6/59 | 199/18866 | 3.61E-05 | 0.00027632 | 0.000118253 | BMP2/FGFR1/KDR/PPARG/STAT1/TGFBR1 | 6 |
| GO:0001836 | biological process | release of cytochrome c from mitochondria | 4/59 | 60/18866 | 3.69E-05 | 0.000281438 | 0.000120443 | BCL2L1/IGF1/MMP9/SOD2 | 4 |
| GO:0060065 | biological process | uterus development | 3/59 | 21/18866 | 3.71E-05 | 0.000282346 | 0.000120832 | ESR1/SRC/TGFB2 | 3 |
| GO:0045471 | biological process | response to ethanol | 5/59 | 122/18866 | 3.94E-05 | 0.000299214 | 0.00012805 | FGFR2/GSTP1/IL2/RARA/TYMS | 5 |
| GO:0007173 | biological process | epidermal growth factor receptor signaling pathway | 5/59 | 123/18866 | 4.10E-05 | 0.00030961 | 0.000132499 | EGFR/MMP9/PIK3R1/PTPN11/SRC | 5 |
| GO:1904375 | biological process | regulation of protein localization to cell periphery | 5/59 | 123/18866 | 4.10E-05 | 0.00030961 | 0.000132499 | AR/BCL2L1/EGFR/HRAS/PIK3R1 | 5 |
| GO:0010632 | biological process | regulation of epithelial cell migration | 7/59 | 301/18866 | 4.12E-05 | 0.000309912 | 0.000132629 | FGFR1/KDR/MET/MMP9/PPARG/SRC/TGFB2 | 7 |
| GO:0090068 | biological process | positive regulation of cell cycle process | 7/59 | 302/18866 | 4.20E-05 | 3.14E-04 | 1.34E-04 | AURKA/CDK2/EGFR/FGFR1/IGF1/INSR/MDM2 | 7 |
| GO:0010676 | biological process | positive regulation of cellular carbohydrate metabolic process | 4/59 | 62/18866 | 4.20E-05 | 3.14E-04 | 1.34E-04 | AKT2/IGF1/INSR/SRC | 4 |
| GO:1904589 | biological process | regulation of protein import | 4/59 | 62/18866 | 4.20E-05 | 3.14E-04 | 1.34E-04 | JAK2/MAPK1/MAPK14/PIK3R1 | 4 |
| GO:0051900 | biological process | regulation of mitochondrial depolarization | 3/59 | 22/18866 | 4.29E-05 | 3.20E-04 | 1.37E-04 | KDR/PARP1/SRC | 3 |
| GO:0035270 | biological process | endocrine system development | 5/59 | 125/18866 | 4.43E-05 | 3.30E-04 | 1.41E-04 | BMP2/INSR/MAP2K1/MAPK1/TGFBR1 | 5 |
| GO:0055008 | biological process | cardiac muscle tissue morphogenesis | 4/59 | 63/18866 | 4.48E-05 | 3.32E-04 | 1.42E-04 | BMP2/FGFR2/TGFB2/TGFBR1 | 4 |
| GO:0018210 | biological process | peptidyl-threonine modification | 5/59 | 126/18866 | 4.60E-05 | 3.41E-04 | 1.46E-04 | CHEK1/MAP2K1/MAPK1/MAPK8/TGFBR1 | 5 |
| GO:0071222 | biological process | cellular response to lipopolysaccharide | 6/59 | 208/18866 | 4.63E-05 | 3.42E-04 | 1.46E-04 | GSTP1/MAPK1/MAPK14/MAPK8/RARA/SRC | 6 |
| GO:0045088 | biological process | regulation of innate immune response | 7/59 | 307/18866 | 4.66E-05 | 3.44E-04 | 1.47E-04 | HRAS/JAK2/PPARG/PTPN11/SRC/STAT1/XIAP | 7 |
| GO:0002066 | biological process | columnar/cuboidal epithelial cell development | 4/59 | 64/18866 | 4.76E-05 | 3.50E-04 | 1.50E-04 | FGFR1/RARA/SRC/TYMS | 4 |
| GO:1905477 | biological process | positive regulation of protein localization to membrane | 5/59 | 127/18866 | 4.78E-05 | 3.50E-04 | 1.50E-04 | AKT2/EGFR/HRAS/MAPK8/PIK3R1 | 5 |
| GO:0034764 | biological process | positive regulation of transmembrane transport | 6/59 | 210/18866 | 4.88E-05 | 3.57E-04 | 1.53E-04 | AKT2/IGF1/INSR/MAPK14/PIK3R1/PTPN11 | 6 |
| GO:1903798 | biological process | regulation of production of miRNAs involved in gene silencing by miRNA | 3/59 | 23/18866 | 4.92E-05 | 3.59E-04 | 1.54E-04 | EGFR/ESR1/MAP2K1 | 3 |
| GO:0071482 | biological process | cellular response to light stimulus | 5/59 | 129/18866 | 5.15E-05 | 3.75E-04 | 1.60E-04 | AKT2/CHEK1/MDM2/PARP1/PIK3R1 | 5 |
| GO:1905207 | biological process | regulation of cardiocyte differentiation | 4/59 | 66/18866 | 5.38E-05 | 3.91E-04 | 1.67E-04 | BMP2/EGFR/IGF1/TGFB2 | 4 |
| GO:0003206 | biological process | cardiac chamber morphogenesis | 5/59 | 131/18866 | 5.54E-05 | 4.00E-04 | 1.71E-04 | FGFR2/MDM2/RARA/TGFB2/TGFBR1 | 5 |
| GO:0046887 | biological process | positive regulation of hormone secretion | 5/59 | 131/18866 | 5.54E-05 | 4.00E-04 | 1.71E-04 | EGFR/FGFR1/JAK2/PTPN11/RAC1 | 5 |
| GO:0019216 | biological process | regulation of lipid metabolic process | 8/59 | 431/18866 | 5.56E-05 | 4.00E-04 | 1.71E-04 | AKT2/BMP2/KIT/PIK3CG/PIK3R1/PPARG/SRC/VDR | 8 |
| GO:0043281 | biological process | regulation of cysteine-type endopeptidase activity involved in apoptotic process | 6/59 | 215/18866 | 5.56E-05 | 4.00E-04 | 1.71E-04 | JAK2/MDM2/MMP9/PPARG/SRC/XIAP | 6 |
| GO:0001759 | biological process | organ induction | 3/59 | 24/18866 | 5.61E-05 | 4.00E-04 | 1.71E-04 | AR/BMP2/FGFR1 | 3 |
| GO:0003181 | biological process | atrioventricular valve morphogenesis | 3/59 | 24/18866 | 5.61E-05 | 4.00E-04 | 1.71E-04 | BMP2/MDM2/TGFB2 | 3 |
| GO:0051882 | biological process | mitochondrial depolarization | 3/59 | 24/18866 | 5.61E-05 | 4.00E-04 | 1.71E-04 | KDR/PARP1/SRC | 3 |
| GO:0060396 | biological process | growth hormone receptor signaling pathway | 3/59 | 24/18866 | 5.61E-05 | 4.00E-04 | 1.71E-04 | JAK2/JAK3/PIK3R1 | 3 |
| GO:0001101 | biological process | response to acid chemical | 5/59 | 132/18866 | 5.75E-05 | 4.08E-04 | 1.74E-04 | BCL2L1/CASP3/EGFR/GSTP1/MMP2 | 5 |
| GO:0042476 | biological process | odontogenesis | 5/59 | 132/18866 | 5.75E-05 | 4.08E-04 | 1.74E-04 | BMP2/CTNNA1/FGFR2/SRC/TGFB2 | 5 |
| GO:0042770 | biological process | signal transduction in response to DNA damage | 5/59 | 133/18866 | 5.96E-05 | 4.22E-04 | 1.80E-04 | AURKA/CDK2/CHEK1/MAPK14/MDM2 | 5 |
| GO:0045428 | biological process | regulation of nitric oxide biosynthetic process | 4/59 | 68/18866 | 6.05E-05 | 4.26E-04 | 1.82E-04 | HSP90AA1/INSR/JAK2/RAC1 | 4 |
| GO:0071479 | biological process | cellular response to ionizing radiation | 4/59 | 68/18866 | 6.05E-05 | 4.26E-04 | 1.82E-04 | BCL2L1/HRAS/MAPK14/MDM2 | 4 |
| GO:0002053 | biological process | positive regulation of mesenchymal cell proliferation | 3/59 | 25/18866 | 6.36E-05 | 4.41E-04 | 1.89E-04 | FGFR1/FGFR2/STAT1 | 3 |
| GO:0060330 | biological process | regulation of response to interferon-gamma | 3/59 | 25/18866 | 6.36E-05 | 4.41E-04 | 1.89E-04 | JAK2/PPARG/STAT1 | 3 |
| GO:0060334 | biological process | regulation of interferon-gamma-mediated signaling pathway | 3/59 | 25/18866 | 6.36E-05 | 4.41E-04 | 1.89E-04 | JAK2/PPARG/STAT1 | 3 |
| GO:0060571 | biological process | morphogenesis of an epithelial fold | 3/59 | 25/18866 | 6.36E-05 | 4.41E-04 | 1.89E-04 | AR/EGFR/FGFR2 | 3 |
| GO:0070920 | biological process | regulation of production of small RNA involved in gene silencing by RNA | 3/59 | 25/18866 | 6.36E-05 | 4.41E-04 | 1.89E-04 | EGFR/ESR1/MAP2K1 | 3 |
| GO:0071378 | biological process | cellular response to growth hormone stimulus | 3/59 | 25/18866 | 6.36E-05 | 4.41E-04 | 1.89E-04 | JAK2/JAK3/PIK3R1 | 3 |
| GO:2001026 | biological process | regulation of endothelial cell chemotaxis | 3/59 | 25/18866 | 6.36E-05 | 4.41E-04 | 1.89E-04 | FGFR1/KDR/MET | 3 |
| GO:0051056 | biological process | regulation of small GTPase mediated signal transduction | 7/59 | 323/18866 | 6.42E-05 | 4.44E-04 | 1.90E-04 | HRAS/IGF1/JAK2/MET/RAC1/SRC/TGFB2 | 7 |
| GO:0031669 | biological process | cellular response to nutrient levels | 6/59 | 221/18866 | 6.48E-05 | 4.47E-04 | 1.91E-04 | ALB/MAPK1/MAPK8/MDM2/PPARG/VDR | 6 |
| GO:0071219 | biological process | cellular response to molecule of bacterial origin | 6/59 | 222/18866 | 6.64E-05 | 4.57E-04 | 1.96E-04 | GSTP1/MAPK1/MAPK14/MAPK8/RARA/SRC | 6 |
| GO:0016485 | biological process | protein processing | 6/59 | 223/18866 | 6.81E-05 | 4.68E-04 | 2.00E-04 | CASP3/MDM2/PARP1/PLAU/SRC/XIAP | 6 |
| GO:0033627 | biological process | cell adhesion mediated by integrin | 4/59 | 71/18866 | 7.17E-05 | 4.85E-04 | 2.08E-04 | PIK3CG/PLAU/PTPN11/TGFB2 | 4 |
| GO:0046579 | biological process | positive regulation of Ras protein signal transduction | 4/59 | 71/18866 | 7.17E-05 | 4.85E-04 | 2.08E-04 | HRAS/IGF1/JAK2/RAC1 | 4 |
| GO:0003171 | biological process | atrioventricular valve development | 3/59 | 26/18866 | 7.18E-05 | 4.85E-04 | 2.08E-04 | BMP2/MDM2/TGFB2 | 3 |
| GO:0043567 | biological process | regulation of insulin-like growth factor receptor signaling pathway | 3/59 | 26/18866 | 7.18E-05 | 4.85E-04 | 2.08E-04 | AR/BMP2/IGF1 | 3 |
| GO:0060390 | biological process | regulation of SMAD protein signal transduction | 3/59 | 26/18866 | 7.18E-05 | 4.85E-04 | 2.08E-04 | JAK2/PARP1/TGFBR1 | 3 |
| GO:0060740 | biological process | prostate gland epithelium morphogenesis | 3/59 | 26/18866 | 7.18E-05 | 4.85E-04 | 2.08E-04 | AR/ESR1/FGFR2 | 3 |
| GO:1903649 | biological process | regulation of cytoplasmic transport | 3/59 | 26/18866 | 7.18E-05 | 4.85E-04 | 2.08E-04 | MAP2K1/MAPK1/SRC | 3 |
| GO:0002433 | biological process | immune response-regulating cell surface receptor signaling pathway involved in phagocytosis | 5/59 | 139/18866 | 7.35E-05 | 0.000494609 | 2.12E-04 | HSP90AA1/MAPK1/PIK3R1/RAC1/SRC | 5 |
| GO:0038096 | biological process | Fc-gamma receptor signaling pathway involved in phagocytosis | 5/59 | 139/18866 | 7.35E-05 | 0.000494609 | 2.12E-04 | HSP90AA1/MAPK1/PIK3R1/RAC1/SRC | 5 |
| GO:0061035 | biological process | regulation of cartilage development | 4/59 | 72/18866 | 7.57E-05 | 0.000508731 | 2.18E-04 | BMP2/PTPN11/RARA/TGFBR1 | 4 |
| GO:0060968 | biological process | regulation of gene silencing | 5/59 | 140/18866 | 7.60E-05 | 0.000509459 | 2.18E-04 | CDK2/EGFR/ESR1/MAP2K1/PPARG | 5 |
| GO:0048705 | biological process | skeletal system morphogenesis | 6/59 | 228/18866 | 7.70E-05 | 0.000515015 | 2.20E-04 | FGFR1/FGFR2/MAPK14/MMP2/RARA/TGFBR1 | 6 |
| GO:0072401 | biological process | signal transduction involved in DNA integrity checkpoint | 4/59 | 73/18866 | 7.99E-05 | 0.000532282 | 0.000227793 | AURKA/CDK2/CHEK1/MDM2 | 4 |
| GO:0072422 | biological process | signal transduction involved in DNA damage checkpoint | 4/59 | 73/18866 | 7.99E-05 | 0.000532282 | 0.000227793 | AURKA/CDK2/CHEK1/MDM2 | 4 |
| GO:0003148 | biological process | outflow tract septum morphogenesis | 3/59 | 27/18866 | 8.06E-05 | 0.000535294 | 0.000229082 | FGFR2/RARA/TGFB2 | 3 |
| GO:0007160 | biological process | cell-matrix adhesion | 6/59 | 230/18866 | 8.08E-05 | 0.000535731 | 0.000229269 | BST1/KDR/PIK3R1/PLAU/RAC1/SRC | 6 |
| GO:0038094 | biological process | Fc-gamma receptor signaling pathway | 5/59 | 142/18866 | 8.13E-05 | 0.00053661 | 0.000229645 | HSP90AA1/MAPK1/PIK3R1/RAC1/SRC | 5 |
| GO:0072073 | biological process | kidney epithelium development | 5/59 | 142/18866 | 8.13E-05 | 0.00053661 | 0.000229645 | BMP2/FGFR1/FGFR2/RARA/STAT1 | 5 |
| GO:0006801 | biological process | superoxide metabolic process | 4/59 | 74/18866 | 8.43E-05 | 0.000554131 | 0.000237143 | BST1/EGFR/GSTP1/SOD2 | 4 |
| GO:0072395 | biological process | signal transduction involved in cell cycle checkpoint | 4/59 | 74/18866 | 8.43E-05 | 0.000554131 | 0.000237143 | AURKA/CDK2/CHEK1/MDM2 | 4 |
| GO:0033143 | biological process | regulation of intracellular steroid hormone receptor signaling pathway | 4/59 | 75/18866 | 8.88E-05 | 0.000582734 | 0.000249384 | AR/ESR1/PARP1/SRC | 4 |
| GO:0002431 | biological process | Fc receptor mediated stimulatory signaling pathway | 5/59 | 145/18866 | 8.97E-05 | 0.000587055 | 0.000251233 | HSP90AA1/MAPK1/PIK3R1/RAC1/SRC | 5 |
| GO:0060512 | biological process | prostate gland morphogenesis | 3/59 | 28/18866 | 9.00E-05 | 0.000587055 | 0.000251233 | AR/ESR1/FGFR2 | 3 |
| GO:0045930 | biological process | negative regulation of mitotic cell cycle | 7/59 | 341/18866 | 9.02E-05 | 0.000587055 | 0.000251233 | AURKA/BCL2L1/CDK2/CHEK1/EGFR/HRAS/MDM2 | 7 |
| GO:0009306 | biological process | protein secretion | 8/59 | 462/18866 | 9.03E-05 | 5.87E-04 | 2.51E-04 | EGFR/IGF1/IL2/JAK2/PTPN11/RAC1/SRC/TGFB2 | 8 |
| GO:0035592 | biological process | establishment of protein localization to extracellular region | 8/59 | 463/18866 | 9.16E-05 | 5.95E-04 | 2.54E-04 | EGFR/IGF1/IL2/JAK2/PTPN11/RAC1/SRC/TGFB2 | 8 |
| GO:0042542 | biological process | response to hydrogen peroxide | 5/59 | 146/18866 | 9.27E-05 | 6.00E-04 | 2.57E-04 | CASP3/MDM2/MET/SRC/STAT1 | 5 |
| GO:0003281 | biological process | ventricular septum development | 4/59 | 76/18866 | 9.36E-05 | 6.03E-04 | 2.58E-04 | FGFR2/MDM2/TGFB2/TGFBR1 | 4 |
| GO:0060415 | biological process | muscle tissue morphogenesis | 4/59 | 76/18866 | 9.36E-05 | 6.03E-04 | 2.58E-04 | BMP2/FGFR2/TGFB2/TGFBR1 | 4 |
| GO:0150076 | biological process | neuroinflammatory response | 4/59 | 77/18866 | 9.85E-05 | 6.34E-04 | 2.71E-04 | EGFR/IGF1/JAK2/MMP9 | 4 |
| GO:2000116 | biological process | regulation of cysteine-type endopeptidase activity | 6/59 | 239/18866 | 9.98E-05 | 6.41E-04 | 2.74E-04 | JAK2/MDM2/MMP9/PPARG/SRC/XIAP | 6 |
| GO:0045822 | biological process | negative regulation of heart contraction | 3/59 | 29/18866 | 1.00E-04 | 6.41E-04 | 2.74E-04 | IL2/JAK2/PIK3CG | 3 |
| GO:0060249 | biological process | anatomical structure homeostasis | 8/59 | 469/18866 | 1.00E-04 | 6.41E-04 | 2.74E-04 | ALB/EGFR/HSP90AA1/MAPK1/PARP1/PTPN11/RAC1/SRC | 8 |
| GO:0071692 | biological process | protein localization to extracellular region | 8/59 | 470/18866 | 1.02E-04 | 6.49E-04 | 2.78E-04 | EGFR/IGF1/IL2/JAK2/PTPN11/RAC1/SRC/TGFB2 | 8 |
| GO:0070665 | biological process | positive regulation of leukocyte proliferation | 5/59 | 150/18866 | 1.05E-04 | 6.70E-04 | 2.87E-04 | BST1/IGF1/IL2/JAK3/MAPK1 | 5 |
| GO:0016331 | biological process | morphogenesis of embryonic epithelium | 5/59 | 151/18866 | 1.09E-04 | 6.90E-04 | 2.95E-04 | AR/CASP3/FGFR2/RARA/TGFB2 | 5 |
| GO:0006809 | biological process | nitric oxide biosynthetic process | 4/59 | 79/18866 | 1.09E-04 | 6.90E-04 | 2.95E-04 | HSP90AA1/INSR/JAK2/RAC1 | 4 |
| GO:0005979 | biological process | regulation of glycogen biosynthetic process | 3/59 | 30/18866 | 1.11E-04 | 7.00E-04 | 3.00E-04 | AKT2/IGF1/INSR | 3 |
| GO:0010962 | biological process | regulation of glucan biosynthetic process | 3/59 | 30/18866 | 1.11E-04 | 7.00E-04 | 3.00E-04 | AKT2/IGF1/INSR | 3 |
| GO:0071480 | biological process | cellular response to gamma radiation | 3/59 | 30/18866 | 1.11E-04 | 7.00E-04 | 3.00E-04 | BCL2L1/HRAS/MDM2 | 3 |
| GO:0001837 | biological process | epithelial to mesenchymal transition | 5/59 | 152/18866 | 1.12E-04 | 7.05E-04 | 3.02E-04 | BMP2/FGFR1/FGFR2/TGFB2/TGFBR1 | 5 |
| GO:0008544 | biological process | epidermis development | 8/59 | 477/18866 | 1.13E-04 | 7.07E-04 | 3.02E-04 | CASP3/EGFR/FGFR1/FGFR2/INSR/MAP2K1/TGFB2/VDR | 8 |
| GO:0042593 | biological process | glucose homeostasis | 6/59 | 245/18866 | 1.14E-04 | 7.16E-04 | 3.06E-04 | IGF1R/INSR/PIK3R1/PPARG/PTPN11/RAC1 | 6 |
| GO:0033500 | biological process | carbohydrate homeostasis | 6/59 | 246/18866 | 1.17E-04 | 7.29E-04 | 3.12E-04 | IGF1R/INSR/PIK3R1/PPARG/PTPN11/RAC1 | 6 |
| GO:0071216 | biological process | cellular response to biotic stimulus | 6/59 | 246/18866 | 1.17E-04 | 7.29E-04 | 3.12E-04 | GSTP1/MAPK1/MAPK14/MAPK8/RARA/SRC | 6 |
| GO:0062013 | biological process | positive regulation of small molecule metabolic process | 5/59 | 154/18866 | 1.19E-04 | 7.42E-04 | 3.17E-04 | AKT2/IGF1/INSR/PPARG/SRC | 5 |
| GO:0010833 | biological process | telomere maintenance via telomere lengthening | 4/59 | 81/18866 | 1.20E-04 | 7.43E-04 | 3.18E-04 | HSP90AA1/MAPK1/PARP1/SRC | 4 |
| GO:0034644 | biological process | cellular response to UV | 4/59 | 81/18866 | 1.20E-04 | 7.43E-04 | 3.18E-04 | CHEK1/MDM2/PARP1/PIK3R1 | 4 |
| GO:0007409 | biological process | axonogenesis | 8/59 | 482/18866 | 1.21E-04 | 7.49E-04 | 3.20E-04 | FGFR2/HSP90AA1/MAP2K1/MAPK1/PIK3R1/PTPN11/RAC1/SRC | 8 |
| GO:0061037 | biological process | negative regulation of cartilage development | 3/59 | 31/18866 | 1.23E-04 | 7.58E-04 | 3.24E-04 | PTPN11/RARA/TGFBR1 | 3 |
| GO:0031589 | biological process | cell-substrate adhesion | 7/59 | 359/18866 | 1.24E-04 | 7.65E-04 | 3.27E-04 | BST1/JAK2/KDR/PIK3R1/PLAU/RAC1/SRC | 7 |
| GO:0001818 | biological process | negative regulation of cytokine production | 7/59 | 360/18866 | 1.26E-04 | 7.77E-04 | 3.33E-04 | FGFR1/GSTP1/IGF1/JAK3/RAC1/RARA/TGFB2 | 7 |
| GO:0043312 | biological process | neutrophil degranulation | 8/59 | 487/18866 | 1.30E-04 | 7.97E-04 | 3.41E-04 | BST1/GSTP1/HSP90AA1/MAPK1/MAPK14/MMP9/PLAU/RAC1 | 8 |
| GO:0051017 | biological process | actin filament bundle assembly | 5/59 | 157/18866 | 1.30E-04 | 7.99E-04 | 3.42E-04 | MET/PIK3R1/RAC1/SRC/TGFBR1 | 5 |
| GO:0048644 | biological process | muscle organ morphogenesis | 4/59 | 83/18866 | 1.32E-04 | 8.04E-04 | 3.44E-04 | BMP2/FGFR2/TGFB2/TGFBR1 | 4 |
| GO:2000243 | biological process | positive regulation of reproductive process | 4/59 | 83/18866 | 1.32E-04 | 8.04E-04 | 3.44E-04 | AR/AURKA/INSR/SRC | 4 |
| GO:0030168 | biological process | platelet activation | 5/59 | 158/18866 | 1.34E-04 | 8.14E-04 | 3.48E-04 | MAPK1/PIK3CG/PIK3R1/PTPN11/SRC | 5 |
| GO:0050729 | biological process | positive regulation of inflammatory response | 5/59 | 158/18866 | 1.34E-04 | 8.14E-04 | 3.48E-04 | EGFR/IL2/JAK2/PIK3CG/PLA2G2A | 5 |
| GO:0035767 | biological process | endothelial cell chemotaxis | 3/59 | 32/18866 | 1.35E-04 | 0.000814298 | 3.48E-04 | FGFR1/KDR/MET | 3 |
| GO:0060317 | biological process | cardiac epithelial to mesenchymal transition | 3/59 | 32/18866 | 1.35E-04 | 0.000814298 | 3.48E-04 | BMP2/TGFB2/TGFBR1 | 3 |
| GO:0071295 | biological process | cellular response to vitamin | 3/59 | 32/18866 | 1.35E-04 | 0.000814298 | 3.48E-04 | MDM2/PPARG/VDR | 3 |
| GO:0097421 | biological process | liver regeneration | 3/59 | 32/18866 | 1.35E-04 | 0.000814298 | 3.48E-04 | AURKA/EGFR/TYMS | 3 |
| GO:0002283 | biological process | neutrophil activation involved in immune response | 8/59 | 490/18866 | 1.36E-04 | 0.000815346 | 0.000348931 | BST1/GSTP1/HSP90AA1/MAPK1/MAPK14/MMP9/PLAU/RAC1 | 8 |
| GO:0045913 | biological process | positive regulation of carbohydrate metabolic process | 4/59 | 84/18866 | 1.38E-04 | 0.000827605 | 0.000354178 | AKT2/IGF1/INSR/SRC | 4 |
| GO:0046209 | biological process | nitric oxide metabolic process | 4/59 | 84/18866 | 1.38E-04 | 0.000827605 | 0.000354178 | HSP90AA1/INSR/JAK2/RAC1 | 4 |
| GO:0090092 | biological process | regulation of transmembrane receptor protein serine/threonine kinase signaling pathway | 6/59 | 254/18866 | 1.39E-04 | 0.000832523 | 0.000356282 | BMP2/JAK2/PARP1/TGFB2/TGFBR1/XIAP | 6 |
| GO:0043154 | biological process | negative regulation of cysteine-type endopeptidase activity involved in apoptotic process | 4/59 | 85/18866 | 0.000144599 | 0.000863067 | 0.000369354 | MDM2/MMP9/SRC/XIAP | 4 |
| GO:0061572 | biological process | actin filament bundle organization | 5/59 | 161/18866 | 0.000146803 | 0.000874513 | 0.000374252 | MET/PIK3R1/RAC1/SRC/TGFBR1 | 5 |
| GO:0010464 | biological process | regulation of mesenchymal cell proliferation | 3/59 | 33/18866 | 0.000148273 | 0.000877164 | 0.000375387 | FGFR1/FGFR2/STAT1 | 3 |
| GO:0048011 | biological process | neurotrophin TRK receptor signaling pathway | 3/59 | 33/18866 | 0.000148273 | 0.000877164 | 0.000375387 | CASP3/PTPN11/SRC | 3 |
| GO:0120255 | biological process | olefinic compound biosynthetic process | 3/59 | 33/18866 | 0.000148273 | 0.000877164 | 0.000375387 | BMP2/GSTM1/GSTP1 | 3 |
| GO:0031334 | biological process | positive regulation of protein-containing complex assembly | 6/59 | 257/18866 | 0.000148399 | 0.000877164 | 0.000375387 | ESR1/HSP90AA1/MET/PARP1/RAC1/SRC | 6 |
| GO:0007611 | biological process | learning or memory | 6/59 | 260/18866 | 0.000158064 | 0.000931627 | 0.000398694 | CASP3/EGFR/INSR/KIT/MAPK1/NQO2 | 6 |
| GO:2001057 | biological process | reactive nitrogen species metabolic process | 4/59 | 87/18866 | 0.000158224 | 0.000931627 | 0.000398694 | HSP90AA1/INSR/JAK2/RAC1 | 4 |
| GO:0032148 | biological process | activation of protein kinase B activity | 3/59 | 34/18866 | 0.000162261 | 0.000951504 | 0.000407201 | IGF1/INSR/SRC | 3 |
| GO:0022412 | biological process | cellular process involved in reproduction in multicellular organism | 7/59 | 375/18866 | 0.000162536 | 0.000951504 | 0.000407201 | AURKA/BCL2L1/IGF1/KIT/RARA/SRC/TGFBR1 | 7 |
| GO:0030111 | biological process | regulation of Wnt signaling pathway | 7/59 | 375/18866 | 0.000162536 | 0.000951504 | 0.000407201 | BMP2/EGFR/ESR1/FGFR2/MAPK14/SRC/XIAP | 7 |
| GO:0007093 | biological process | mitotic cell cycle checkpoint | 5/59 | 166/18866 | 0.00016936 | 0.000989553 | 0.000423484 | AURKA/BCL2L1/CDK2/HRAS/MDM2 | 5 |
| GO:0048863 | biological process | stem cell differentiation | 6/59 | 264/18866 | 0.00017172 | 0.001001426 | 0.000428565 | ERBB4/ESR1/FGFR2/KIT/MAPK1/TGFB2 | 6 |
| GO:0001892 | biological process | embryonic placenta development | 4/59 | 89/18866 | 0.000172754 | 0.001005532 | 0.000430322 | EGFR/FGFR2/MAP2K1/MAPK1 | 4 |
| GO:0034390 | biological process | smooth muscle cell apoptotic process | 3/59 | 35/18866 | 0.000177079 | 0.001024841 | 0.000438586 | IGF1/PPARG/SOD2 | 3 |
| GO:0034391 | biological process | regulation of smooth muscle cell apoptotic process | 3/59 | 35/18866 | 0.000177079 | 0.001024841 | 0.000438586 | IGF1/PPARG/SOD2 | 3 |
| GO:0098751 | biological process | bone cell development | 3/59 | 35/18866 | 0.000177079 | 0.001024841 | 0.000438586 | KIT/PTPN11/SRC | 3 |
| GO:0038095 | biological process | Fc-epsilon receptor signaling pathway | 5/59 | 169/18866 | 0.00018411 | 0.001063513 | 0.000455136 | ITK/MAPK1/MAPK8/PIK3R1/RAC1 | 5 |
| GO:0034103 | biological process | regulation of tissue remodeling | 4/59 | 91/18866 | 0.000188224 | 0.001083175 | 0.00046355 | EGFR/IL2/SRC/VDR | 4 |
| GO:0051492 | biological process | regulation of stress fiber assembly | 4/59 | 91/18866 | 0.000188224 | 0.001083175 | 0.00046355 | MET/PIK3R1/RAC1/TGFBR1 | 4 |
| GO:0031349 | biological process | positive regulation of defense response | 7/59 | 385/18866 | 0.000191023 | 0.001097213 | 0.000469558 | EGFR/HRAS/IL2/JAK2/PIK3CG/PLA2G2A/SRC | 7 |
| GO:0031128 | biological process | developmental induction | 3/59 | 36/18866 | 0.000192749 | 0.001100905 | 0.000471138 | AR/BMP2/FGFR1 | 3 |
| GO:0045622 | biological process | regulation of T-helper cell differentiation | 3/59 | 36/18866 | 0.000192749 | 0.001100905 | 0.000471138 | IL2/JAK3/RARA | 3 |
| GO:0070873 | biological process | regulation of glycogen metabolic process | 3/59 | 36/18866 | 0.000192749 | 0.001100905 | 0.000471138 | AKT2/IGF1/INSR | 3 |
| GO:0051091 | biological process | positive regulation of DNA-binding transcription factor activity | 6/59 | 270/18866 | 1.94E-04 | 1.11E-03 | 4.73E-04 | AR/ESR1/ESR2/JAK2/KIT/PPARG | 6 |
| GO:0030307 | biological process | positive regulation of cell growth | 5/59 | 171/18866 | 1.94E-04 | 1.11E-03 | 4.74E-04 | EGFR/IGF1/IL2/TGFB2/TGFBR1 | 5 |
| GO:2000117 | biological process | negative regulation of cysteine-type endopeptidase activity | 4/59 | 93/18866 | 2.05E-04 | 1.16E-03 | 4.97E-04 | MDM2/MMP9/SRC/XIAP | 4 |
| GO:0031331 | biological process | positive regulation of cellular catabolic process | 7/59 | 390/18866 | 2.07E-04 | 1.17E-03 | 5.02E-04 | AKT2/AURKA/HSP90AA1/IGF1/INSR/KDR/MDM2 | 7 |
| GO:0045730 | biological process | respiratory burst | 3/59 | 37/18866 | 2.09E-04 | 1.18E-03 | 5.06E-04 | INSR/PIK3CG/RAC1 | 3 |
| GO:0090322 | biological process | regulation of superoxide metabolic process | 3/59 | 37/18866 | 2.09E-04 | 1.18E-03 | 5.06E-04 | BST1/EGFR/GSTP1 | 3 |
| GO:0003205 | biological process | cardiac chamber development | 5/59 | 174/18866 | 2.11E-04 | 1.19E-03 | 5.09E-04 | FGFR2/MDM2/RARA/TGFB2/TGFBR1 | 5 |
| GO:0010660 | biological process | regulation of muscle cell apoptotic process | 4/59 | 94/18866 | 2.13E-04 | 1.20E-03 | 5.13E-04 | IGF1/JAK2/PPARG/SOD2 | 4 |
| GO:0045778 | biological process | positive regulation of ossification | 4/59 | 94/18866 | 2.13E-04 | 1.20E-03 | 5.13E-04 | BMP2/IGF1/PTPN11/TGFB2 | 4 |
| GO:0007281 | biological process | germ cell development | 6/59 | 275/18866 | 2.14E-04 | 1.20E-03 | 5.14E-04 | AURKA/BCL2L1/IGF1/KIT/RARA/SRC | 6 |
| GO:0003156 | biological process | regulation of animal organ formation | 3/59 | 38/18866 | 2.27E-04 | 1.26E-03 | 5.38E-04 | AR/BMP2/FGFR1 | 3 |
| GO:0010742 | biological process | macrophage derived foam cell differentiation | 3/59 | 38/18866 | 2.27E-04 | 1.26E-03 | 5.38E-04 | PLA2G2A/PPARG/STAT1 | 3 |
| GO:0032885 | biological process | regulation of polysaccharide biosynthetic process | 3/59 | 38/18866 | 2.27E-04 | 1.26E-03 | 5.38E-04 | AKT2/IGF1/INSR | 3 |
| GO:0043029 | biological process | T cell homeostasis | 3/59 | 38/18866 | 2.27E-04 | 1.26E-03 | 5.38E-04 | CASP3/IL2/JAK3 | 3 |
| GO:0090077 | biological process | foam cell differentiation | 3/59 | 38/18866 | 2.27E-04 | 1.26E-03 | 5.38E-04 | PLA2G2A/PPARG/STAT1 | 3 |
| GO:1903523 | biological process | negative regulation of blood circulation | 3/59 | 38/18866 | 2.27E-04 | 1.26E-03 | 5.38E-04 | IL2/JAK2/PIK3CG | 3 |
| GO:0048469 | biological process | cell maturation | 5/59 | 179/18866 | 2.40E-04 | 1.32E-03 | 5.67E-04 | AURKA/FGFR1/PGR/PPARG/TYMS | 5 |
| GO:0036473 | biological process | cell death in response to oxidative stress | 4/59 | 97/18866 | 2.41E-04 | 1.32E-03 | 5.67E-04 | JAK2/MET/PARP1/SOD2 | 4 |
| GO:1901216 | biological process | positive regulation of neuron death | 4/59 | 97/18866 | 2.41E-04 | 1.32E-03 | 5.67E-04 | CASP3/NQO2/PARP1/TGFB2 | 4 |
| GO:1901655 | biological process | cellular response to ketone | 4/59 | 97/18866 | 2.41E-04 | 1.32E-03 | 5.67E-04 | AR/EGFR/PPARG/SRC | 4 |
| GO:0038179 | biological process | neurotrophin signaling pathway | 3/59 | 39/18866 | 2.45E-04 | 1.34E-03 | 5.73E-04 | CASP3/PTPN11/SRC | 3 |
| GO:0045022 | biological process | early endosome to late endosome transport | 3/59 | 39/18866 | 2.45E-04 | 1.34E-03 | 5.73E-04 | MAP2K1/MAPK1/SRC | 3 |
| GO:0060416 | biological process | response to growth hormone | 3/59 | 39/18866 | 2.45E-04 | 1.34E-03 | 5.73E-04 | JAK2/JAK3/PIK3R1 | 3 |
| GO:1904706 | biological process | negative regulation of vascular associated smooth muscle cell proliferation | 3/59 | 39/18866 | 2.45E-04 | 1.34E-03 | 5.73E-04 | GSTP1/PPARG/SOD2 | 3 |
| GO:1901796 | biological process | regulation of signal transduction by p53 class mediator | 5/59 | 180/18866 | 2.47E-04 | 1.35E-03 | 5.76E-04 | AURKA/CDK2/CHEK1/MAPK14/MDM2 | 5 |
| GO:0046651 | biological process | lymphocyte proliferation | 6/59 | 283/18866 | 2.50E-04 | 1.36E-03 | 5.81E-04 | BST1/CASP3/IGF1/IL2/JAK3/PIK3CG | 6 |
| GO:0001657 | biological process | ureteric bud development | 4/59 | 98/18866 | 2.50E-04 | 1.36E-03 | 5.81E-04 | BMP2/FGFR1/FGFR2/RARA | 4 |
| GO:0010657 | biological process | muscle cell apoptotic process | 4/59 | 98/18866 | 2.50E-04 | 1.36E-03 | 5.81E-04 | IGF1/JAK2/PPARG/SOD2 | 4 |
| GO:1905330 | biological process | regulation of morphogenesis of an epithelium | 5/59 | 181/18866 | 2.53E-04 | 1.37E-03 | 5.87E-04 | AR/ESR1/FGFR1/RAC1/STAT1 | 5 |
| GO:0072163 | biological process | mesonephric epithelium development | 4/59 | 99/18866 | 2.60E-04 | 1.40E-03 | 6.01E-04 | BMP2/FGFR1/FGFR2/RARA | 4 |
| GO:0072164 | biological process | mesonephric tubule development | 4/59 | 99/18866 | 2.60E-04 | 1.40E-03 | 6.01E-04 | BMP2/FGFR1/FGFR2/RARA | 4 |
| GO:1902895 | biological process | positive regulation of pri-miRNA transcription by RNA polymerase II | 3/59 | 40/18866 | 2.64E-04 | 1.42E-03 | 6.09E-04 | BMP2/PPARG/TGFB2 | 3 |
| GO:0032943 | biological process | mononuclear cell proliferation | 6/59 | 286/18866 | 2.64E-04 | 1.42E-03 | 6.09E-04 | BST1/CASP3/IGF1/IL2/JAK3/PIK3CG | 6 |
| GO:0001959 | biological process | regulation of cytokine-mediated signaling pathway | 5/59 | 183/18866 | 2.66E-04 | 1.43E-03 | 6.11E-04 | GSTP1/JAK2/PPARG/PTPN11/STAT1 | 5 |
| GO:0030177 | biological process | positive regulation of Wnt signaling pathway | 5/59 | 183/18866 | 2.66E-04 | 1.43E-03 | 6.11E-04 | BMP2/EGFR/FGFR2/SRC/XIAP | 5 |
| GO:0002831 | biological process | regulation of response to biotic stimulus | 7/59 | 409/18866 | 2.76E-04 | 1.48E-03 | 6.32E-04 | HRAS/JAK2/PPARG/PTPN11/SRC/STAT1/XIAP | 7 |
| GO:0060491 | biological process | regulation of cell projection assembly | 5/59 | 185/18866 | 2.80E-04 | 1.50E-03 | 6.40E-04 | HRAS/KIT/RAC1/SRC/TGFBR1 | 5 |
| GO:2000826 | biological process | regulation of heart morphogenesis | 3/59 | 41/18866 | 2.85E-04 | 1.52E-03 | 6.49E-04 | BMP2/TGFB2/TGFBR1 | 3 |
| GO:0000079 | biological process | regulation of cyclin-dependent protein serine/threonine kinase activity | 4/59 | 102/18866 | 2.92E-04 | 1.55E-03 | 6.62E-04 | CASP3/CCNA2/EGFR/SRC | 4 |
| GO:0110020 | biological process | regulation of actomyosin structure organization | 4/59 | 102/18866 | 2.92E-04 | 1.55E-03 | 6.62E-04 | MET/PIK3R1/RAC1/TGFBR1 | 4 |
| GO:1903076 | biological process | regulation of protein localization to plasma membrane | 4/59 | 102/18866 | 2.92E-04 | 1.55E-03 | 6.62E-04 | AR/BCL2L1/EGFR/PIK3R1 | 4 |
| GO:0051604 | biological process | protein maturation | 6/59 | 293/18866 | 3.01E-04 | 1.59E-03 | 6.82E-04 | CASP3/MDM2/PARP1/PLAU/SRC/XIAP | 6 |
| GO:0001823 | biological process | mesonephros development | 4/59 | 103/18866 | 3.03E-04 | 1.60E-03 | 6.84E-04 | BMP2/FGFR1/FGFR2/RARA | 4 |
| GO:0030593 | biological process | neutrophil chemotaxis | 4/59 | 103/18866 | 3.03E-04 | 1.60E-03 | 6.84E-04 | BST1/PIK3CG/RAC1/TGFB2 | 4 |
| GO:0046006 | biological process | regulation of activated T cell proliferation | 3/59 | 42/18866 | 3.06E-04 | 1.61E-03 | 6.88E-04 | CASP3/IGF1/IL2 | 3 |
| GO:0098927 | biological process | vesicle-mediated transport between endosomal compartments | 3/59 | 42/18866 | 3.06E-04 | 1.61E-03 | 6.88E-04 | MAP2K1/MAPK1/SRC | 3 |
| GO:0060828 | biological process | regulation of canonical Wnt signaling pathway | 6/59 | 295/18866 | 3.12E-04 | 1.64E-03 | 7.01E-04 | BMP2/EGFR/FGFR2/MAPK14/SRC/XIAP | 6 |
| GO:0032231 | biological process | regulation of actin filament bundle assembly | 4/59 | 104/18866 | 3.14E-04 | 1.64E-03 | 7.03E-04 | MET/PIK3R1/RAC1/TGFBR1 | 4 |
| GO:0120034 | biological process | positive regulation of plasma membrane bounded cell projection assembly | 4/59 | 104/18866 | 3.14E-04 | 1.64E-03 | 7.03E-04 | HRAS/KIT/RAC1/TGFBR1 | 4 |
| GO:0003015 | biological process | heart process | 6/59 | 297/18866 | 3.24E-04 | 1.69E-03 | 7.21E-04 | IL2/JAK2/MDM2/PIK3CG/SRC/TGFB2 | 6 |
| GO:0001558 | biological process | regulation of cell growth | 7/59 | 420/18866 | 3.24E-04 | 1.69E-03 | 7.21E-04 | EGFR/ESR2/IGF1/IL2/PPARG/TGFB2/TGFBR1 | 7 |
| GO:0010821 | biological process | regulation of mitochondrion organization | 5/59 | 191/18866 | 3.24E-04 | 1.69E-03 | 7.21E-04 | BCL2L1/IGF1/KDR/MAPK8/MMP9 | 5 |
| GO:0022408 | biological process | negative regulation of cell-cell adhesion | 5/59 | 191/18866 | 3.24E-04 | 1.69E-03 | 7.21E-04 | BMP2/CASP3/IL2/JAK2/JAK3 | 5 |
| GO:1902106 | biological process | negative regulation of leukocyte differentiation | 4/59 | 105/18866 | 3.26E-04 | 1.69E-03 | 7.23E-04 | IL2/JAK3/PIK3R1/RARA | 4 |
| GO:0008631 | biological process | intrinsic apoptotic signaling pathway in response to oxidative stress | 3/59 | 43/18866 | 3.28E-04 | 1.70E-03 | 7.27E-04 | JAK2/PARP1/SOD2 | 3 |
| GO:0046632 | biological process | alpha-beta T cell differentiation | 4/59 | 106/18866 | 3.38E-04 | 1.74E-03 | 7.45E-04 | IL2/ITK/JAK3/RARA | 4 |
| GO:0071156 | biological process | regulation of cell cycle arrest | 4/59 | 106/18866 | 3.38E-04 | 1.74E-03 | 7.45E-04 | AKT2/AURKA/CDK2/MDM2 | 4 |
| GO:1904029 | biological process | regulation of cyclin-dependent protein kinase activity | 4/59 | 106/18866 | 3.38E-04 | 1.74E-03 | 7.45E-04 | CASP3/CCNA2/EGFR/SRC | 4 |
| GO:0071248 | biological process | cellular response to metal ion | 5/59 | 193/18866 | 3.40E-04 | 1.75E-03 | 7.49E-04 | EGFR/MAPK1/MAPK8/MMP9/PARP1 | 5 |
| GO:0043588 | biological process | skin development | 7/59 | 425/18866 | 3.48E-04 | 1.79E-03 | 7.65E-04 | CASP3/EGFR/FGFR2/MAP2K1/MET/TGFB2/VDR | 7 |
| GO:1903426 | biological process | regulation of reactive oxygen species biosynthetic process | 4/59 | 107/18866 | 3.50E-04 | 1.79E-03 | 7.68E-04 | HSP90AA1/INSR/JAK2/RAC1 | 4 |
| GO:0031670 | biological process | cellular response to nutrient | 3/59 | 44/18866 | 3.51E-04 | 1.79E-03 | 7.68E-04 | MDM2/PPARG/VDR | 3 |
| GO:0032881 | biological process | regulation of polysaccharide metabolic process | 3/59 | 44/18866 | 3.51E-04 | 1.79E-03 | 7.68E-04 | AKT2/IGF1/INSR | 3 |
| GO:0050890 | biological process | cognition | 6/59 | 302/18866 | 3.54E-04 | 1.80E-03 | 7.72E-04 | CASP3/EGFR/INSR/KIT/MAPK1/NQO2 | 6 |
| GO:0042098 | biological process | T cell proliferation | 5/59 | 195/18866 | 3.57E-04 | 1.82E-03 | 7.78E-04 | CASP3/IGF1/IL2/JAK3/PIK3CG | 5 |
| GO:1903522 | biological process | regulation of blood circulation | 6/59 | 303/18866 | 3.60E-04 | 1.83E-03 | 7.83E-04 | EGFR/IL2/JAK2/MDM2/PIK3CG/TGFB2 | 6 |
| GO:0007229 | biological process | integrin-mediated signaling pathway | 4/59 | 108/18866 | 3.63E-04 | 1.84E-03 | 7.87E-04 | BST1/CTNNA1/PTPN11/SRC | 4 |
| GO:0008593 | biological process | regulation of Notch signaling pathway | 4/59 | 108/18866 | 3.63E-04 | 1.84E-03 | 7.87E-04 | EGFR/KIT/SRC/TGFB2 | 4 |
| GO:0060759 | biological process | regulation of response to cytokine stimulus | 5/59 | 196/18866 | 3.65E-04 | 1.84E-03 | 7.89E-04 | GSTP1/JAK2/PPARG/PTPN11/STAT1 | 5 |
| GO:0071897 | biological process | DNA biosynthetic process | 5/59 | 196/18866 | 3.65E-04 | 1.84E-03 | 7.89E-04 | HSP90AA1/MAPK1/PPARG/SRC/TYMS | 5 |
| GO:0034105 | biological process | positive regulation of tissue remodeling | 3/59 | 45/18866 | 3.75E-04 | 1.88E-03 | 8.04E-04 | EGFR/IL2/VDR | 3 |
| GO:0045429 | biological process | positive regulation of nitric oxide biosynthetic process | 3/59 | 45/18866 | 3.75E-04 | 1.88E-03 | 8.04E-04 | HSP90AA1/INSR/JAK2 | 3 |
| GO:0060412 | biological process | ventricular septum morphogenesis | 3/59 | 45/18866 | 3.75E-04 | 1.88E-03 | 8.04E-04 | FGFR2/TGFB2/TGFBR1 | 3 |
| GO:1904646 | biological process | cellular response to amyloid-beta | 3/59 | 45/18866 | 3.75E-04 | 1.88E-03 | 8.04E-04 | IGF1/IGF1R/PARP1 | 3 |
| GO:0021761 | biological process | limbic system development | 4/59 | 109/18866 | 3.76E-04 | 1.88E-03 | 8.04E-04 | CASP3/FGFR1/FGFR2/RARA | 4 |
| GO:2000278 | biological process | regulation of DNA biosynthetic process | 4/59 | 109/18866 | 3.76E-04 | 1.88E-03 | 8.04E-04 | HSP90AA1/MAPK1/PPARG/SRC | 4 |
| GO:0017038 | biological process | protein import | 5/59 | 200/18866 | 4.01E-04 | 1.98E-03 | 8.47E-04 | HSP90AA1/JAK2/MAPK1/MAPK14/PIK3R1 | 5 |
| GO:0031032 | biological process | actomyosin structure organization | 5/59 | 200/18866 | 4.01E-04 | 1.98E-03 | 8.47E-04 | MET/PIK3R1/RAC1/SRC/TGFBR1 | 5 |
| GO:0005978 | biological process | glycogen biosynthetic process | 3/59 | 46/18866 | 4.01E-04 | 0.001978039 | 8.47E-04 | AKT2/IGF1/INSR | 3 |
| GO:0009250 | biological process | glucan biosynthetic process | 3/59 | 46/18866 | 4.01E-04 | 0.001978039 | 8.47E-04 | AKT2/IGF1/INSR | 3 |
| GO:0032570 | biological process | response to progesterone | 3/59 | 46/18866 | 4.01E-04 | 0.001978039 | 8.47E-04 | SRC/TGFB2/TYMS | 3 |
| GO:0048538 | biological process | thymus development | 3/59 | 46/18866 | 4.01E-04 | 0.001978039 | 8.47E-04 | MAP2K1/MAPK1/TGFBR1 | 3 |
| GO:0050798 | biological process | activated T cell proliferation | 3/59 | 46/18866 | 4.01E-04 | 0.001978039 | 8.47E-04 | CASP3/IGF1/IL2 | 3 |
| GO:1904407 | biological process | positive regulation of nitric oxide metabolic process | 3/59 | 46/18866 | 4.01E-04 | 0.001978039 | 8.47E-04 | HSP90AA1/INSR/JAK2 | 3 |
| GO:0044843 | biological process | cell cycle G1/S phase transition | 6/59 | 310/18866 | 4.06E-04 | 0.002002731 | 8.57E-04 | AURKA/CCNA2/CDK2/EGFR/MDM2/TYMS | 6 |
| GO:0003149 | biological process | membranous septum morphogenesis | 2/59 | 10/18866 | 4.26E-04 | 0.002084333 | 8.92E-04 | FGFR2/TGFB2 | 2 |
| GO:0051901 | biological process | positive regulation of mitochondrial depolarization | 2/59 | 10/18866 | 4.26E-04 | 0.002084333 | 0.000892001 | KDR/PARP1 | 2 |
| GO:0060502 | biological process | epithelial cell proliferation involved in lung morphogenesis | 2/59 | 10/18866 | 4.26E-04 | 0.002084333 | 0.000892001 | FGFR2/MAP2K1 | 2 |
| GO:0071104 | biological process | response to interleukin-9 | 2/59 | 10/18866 | 4.26E-04 | 0.002084333 | 0.000892001 | JAK3/STAT1 | 2 |
| GO:0048806 | biological process | genitalia development | 3/59 | 47/18866 | 4.27E-04 | 0.002084611 | 0.00089212 | AR/ESR1/PTPN11 | 3 |
| GO:0051150 | biological process | regulation of smooth muscle cell differentiation | 3/59 | 47/18866 | 4.27E-04 | 0.002084611 | 0.00089212 | FGFR2/KIT/SOD2 | 3 |
| GO:0001938 | biological process | positive regulation of endothelial cell proliferation | 4/59 | 113/18866 | 4.31E-04 | 0.002099253 | 0.000898386 | BMP2/FGFR1/KDR/TGFBR1 | 4 |
| GO:1990748 | biological process | cellular detoxification | 4/59 | 114/18866 | 4.45E-04 | 0.002167084 | 0.000927415 | ALB/GSTM1/GSTP1/SOD2 | 4 |
| GO:0010862 | biological process | positive regulation of pathway-restricted SMAD protein phosphorylation | 3/59 | 48/18866 | 0.000454643 | 0.002194084 | 0.000938969 | BMP2/TGFB2/TGFBR1 | 3 |
| GO:0014911 | biological process | positive regulation of smooth muscle cell migration | 3/59 | 48/18866 | 0.000454643 | 0.002194084 | 0.000938969 | IGF1/MDM2/SRC | 3 |
| GO:0043370 | biological process | regulation of CD4-positive, alpha-beta T cell differentiation | 3/59 | 48/18866 | 0.000454643 | 0.002194084 | 0.000938969 | IL2/JAK3/RARA | 3 |
| GO:0055010 | biological process | ventricular cardiac muscle tissue morphogenesis | 3/59 | 48/18866 | 0.000454643 | 0.002194084 | 0.000938969 | FGFR2/TGFB2/TGFBR1 | 3 |
| GO:2001239 | biological process | regulation of extrinsic apoptotic signaling pathway in absence of ligand | 3/59 | 48/18866 | 0.000454643 | 0.002194084 | 0.000938969 | BCL2L1/CTNNA1/FGFR1 | 3 |
| GO:0032609 | biological process | interferon-gamma production | 4/59 | 115/18866 | 0.00046048 | 0.002218741 | 0.000949522 | HRAS/IL2/ITK/RARA | 4 |
| GO:0060964 | biological process | regulation of gene silencing by miRNA | 4/59 | 116/18866 | 0.00047582 | 0.002289039 | 0.000979606 | EGFR/ESR1/MAP2K1/PPARG | 4 |
| GO:0034612 | biological process | response to tumor necrosis factor | 6/59 | 320/18866 | 0.000480798 | 0.002295295 | 0.000982283 | CASP3/GSTP1/JAK2/MAPK1/MAPK14/STAT1 | 6 |
| GO:0002067 | biological process | glandular epithelial cell differentiation | 3/59 | 49/18866 | 0.00048322 | 0.002295295 | 0.000982283 | BMP2/FGFR2/RARA | 3 |
| GO:0002673 | biological process | regulation of acute inflammatory response | 3/59 | 49/18866 | 0.00048322 | 0.002295295 | 0.000982283 | GSTP1/PIK3CG/PPARG | 3 |
| GO:0003254 | biological process | regulation of membrane depolarization | 3/59 | 49/18866 | 0.00048322 | 0.002295295 | 0.000982283 | KDR/PARP1/SRC | 3 |
| GO:0035196 | biological process | production of miRNAs involved in gene silencing by miRNA | 3/59 | 49/18866 | 0.00048322 | 0.002295295 | 0.000982283 | EGFR/ESR1/MAP2K1 | 3 |
| GO:0060711 | biological process | labyrinthine layer development | 3/59 | 49/18866 | 0.00048322 | 0.002295295 | 0.000982283 | FGFR2/MAP2K1/MAPK1 | 3 |
| GO:0061383 | biological process | trabecula morphogenesis | 3/59 | 49/18866 | 0.00048322 | 0.002295295 | 0.000982283 | MMP2/TGFB2/TGFBR1 | 3 |
| GO:0090199 | biological process | regulation of release of cytochrome c from mitochondria | 3/59 | 49/18866 | 0.00048322 | 0.002295295 | 0.000982283 | BCL2L1/IGF1/MMP9 | 3 |
| GO:0043254 | biological process | regulation of protein-containing complex assembly | 7/59 | 449/18866 | 0.000483893 | 0.002295295 | 0.000982283 | ESR1/HSP90AA1/MET/PARP1/PTPN11/RAC1/SRC | 7 |
| GO:1904019 | biological process | epithelial cell apoptotic process | 4/59 | 117/18866 | 0.000491521 | 0.002327856 | 0.000996218 | BCL2L1/JAK2/KDR/PIK3CG | 4 |
| GO:0007569 | biological process | cell aging | 4/59 | 118/18866 | 0.000507588 | 0.002392616 | 0.001023932 | CHEK1/HRAS/MAP2K1/MAPK14 | 4 |
| GO:0008543 | biological process | fibroblast growth factor receptor signaling pathway | 4/59 | 118/18866 | 0.000507588 | 0.002392616 | 0.001023932 | FGFR1/FGFR2/MAPK1/PTPN11 | 4 |
| GO:0010718 | biological process | positive regulation of epithelial to mesenchymal transition | 3/59 | 50/18866 | 0.000512925 | 0.002392616 | 0.001023932 | BMP2/TGFB2/TGFBR1 | 3 |
| GO:0031648 | biological process | protein destabilization | 3/59 | 50/18866 | 5.13E-04 | 2.39E-03 | 1.02E-03 | BMP2/MDM2/SRC | 3 |
| GO:0032760 | biological process | positive regulation of tumor necrosis factor production | 3/59 | 50/18866 | 5.13E-04 | 2.39E-03 | 1.02E-03 | JAK2/PIK3R1/PTPN11 | 3 |
| GO:0009896 | biological process | positive regulation of catabolic process | 7/59 | 454/18866 | 5.17E-04 | 2.39E-03 | 1.02E-03 | AKT2/AURKA/HSP90AA1/IGF1/INSR/KDR/MDM2 | 7 |
| GO:0050804 | biological process | modulation of chemical synaptic transmission | 7/59 | 454/18866 | 5.17E-04 | 2.39E-03 | 1.02E-03 | EGFR/HRAS/JAK2/KIT/MAPK1/RARA/SRC | 7 |
| GO:0033148 | biological process | positive regulation of intracellular estrogen receptor signaling pathway | 2/59 | 11/18866 | 5.19E-04 | 2.39E-03 | 1.02E-03 | AR/PARP1 | 2 |
| GO:0033483 | biological process | gas homeostasis | 2/59 | 11/18866 | 5.19E-04 | 2.39E-03 | 1.02E-03 | GSTP1/SOD2 | 2 |
| GO:0048103 | biological process | somatic stem cell division | 2/59 | 11/18866 | 5.19E-04 | 2.39E-03 | 1.02E-03 | KIT/TGFB2 | 2 |
| GO:0051974 | biological process | negative regulation of telomerase activity | 2/59 | 11/18866 | 5.19E-04 | 2.39E-03 | 1.02E-03 | PPARG/SRC | 2 |
| GO:0060281 | biological process | regulation of oocyte development | 2/59 | 11/18866 | 5.19E-04 | 2.39E-03 | 1.02E-03 | AURKA/IGF1 | 2 |
| GO:0060439 | biological process | trachea morphogenesis | 2/59 | 11/18866 | 5.19E-04 | 2.39E-03 | 1.02E-03 | MAP2K1/MAPK1 | 2 |
| GO:0060525 | biological process | prostate glandular acinus development | 2/59 | 11/18866 | 5.19E-04 | 2.39E-03 | 1.02E-03 | ESR1/FGFR2 | 2 |
| GO:0070106 | biological process | interleukin-27-mediated signaling pathway | 2/59 | 11/18866 | 5.19E-04 | 2.39E-03 | 1.02E-03 | JAK2/STAT1 | 2 |
| GO:0070757 | biological process | interleukin-35-mediated signaling pathway | 2/59 | 11/18866 | 5.19E-04 | 2.39E-03 | 1.02E-03 | JAK2/STAT1 | 2 |
| GO:1903800 | biological process | positive regulation of production of miRNAs involved in gene silencing by miRNA | 2/59 | 11/18866 | 5.19E-04 | 2.39E-03 | 1.02E-03 | EGFR/MAP2K1 | 2 |
| GO:1905879 | biological process | regulation of oogenesis | 2/59 | 11/18866 | 5.19E-04 | 2.39E-03 | 1.02E-03 | AURKA/IGF1 | 2 |
| GO:0051701 | biological process | interaction with host | 5/59 | 212/18866 | 5.23E-04 | 2.40E-03 | 1.03E-03 | BCL2L1/EGFR/INSR/MET/SRC | 5 |
| GO:0007389 | biological process | pattern specification process | 7/59 | 455/18866 | 5.24E-04 | 2.40E-03 | 1.03E-03 | AR/AURKA/BMP2/ERBB4/FGFR1/FGFR2/TGFBR1 | 7 |
| GO:0099177 | biological process | regulation of trans-synaptic signaling | 7/59 | 455/18866 | 5.24E-04 | 2.40E-03 | 1.03E-03 | EGFR/HRAS/JAK2/KIT/MAPK1/RARA/SRC | 7 |
| GO:0007254 | biological process | JNK cascade | 5/59 | 213/18866 | 5.34E-04 | 2.45E-03 | 1.05E-03 | EGFR/GSTP1/HRAS/IGF1R/MAPK8 | 5 |
| GO:0060147 | biological process | regulation of posttranscriptional gene silencing | 4/59 | 120/18866 | 5.41E-04 | 2.47E-03 | 1.06E-03 | EGFR/ESR1/MAP2K1/PPARG | 4 |
| GO:0060966 | biological process | regulation of gene silencing by RNA | 4/59 | 120/18866 | 5.41E-04 | 2.47E-03 | 1.06E-03 | EGFR/ESR1/MAP2K1/PPARG | 4 |
| GO:1902893 | biological process | regulation of pri-miRNA transcription by RNA polymerase II | 3/59 | 51/18866 | 5.44E-04 | 2.48E-03 | 1.06E-03 | BMP2/PPARG/TGFB2 | 3 |
| GO:0006006 | biological process | glucose metabolic process | 5/59 | 214/18866 | 5.45E-04 | 2.48E-03 | 1.06E-03 | AKT2/IGF1/INSR/MAPK14/SRC | 5 |
| GO:0042113 | biological process | B cell activation | 6/59 | 328/18866 | 5.48E-04 | 2.49E-03 | 1.07E-03 | BST1/CASP3/IL2/JAK3/KIT/PIK3R1 | 6 |
| GO:0010906 | biological process | regulation of glucose metabolic process | 4/59 | 121/18866 | 5.58E-04 | 2.53E-03 | 1.08E-03 | AKT2/IGF1/INSR/SRC | 4 |
| GO:0097237 | biological process | cellular response to toxic substance | 4/59 | 122/18866 | 5.76E-04 | 2.59E-03 | 1.11E-03 | ALB/GSTM1/GSTP1/SOD2 | 4 |
| GO:1990266 | biological process | neutrophil migration | 4/59 | 122/18866 | 5.76E-04 | 2.59E-03 | 1.11E-03 | BST1/PIK3CG/RAC1/TGFB2 | 4 |
| GO:0003179 | biological process | heart valve morphogenesis | 3/59 | 52/18866 | 5.76E-04 | 2.59E-03 | 1.11E-03 | BMP2/MDM2/TGFB2 | 3 |
| GO:0031103 | biological process | axon regeneration | 3/59 | 52/18866 | 5.76E-04 | 2.59E-03 | 1.11E-03 | CTNNA1/JAK2/MAP2K1 | 3 |
| GO:0060425 | biological process | lung morphogenesis | 3/59 | 52/18866 | 5.76E-04 | 2.59E-03 | 1.11E-03 | FGFR2/MAP2K1/MAPK1 | 3 |
| GO:1903557 | biological process | positive regulation of tumor necrosis factor superfamily cytokine production | 3/59 | 52/18866 | 5.76E-04 | 2.59E-03 | 1.11E-03 | JAK2/PIK3R1/PTPN11 | 3 |
| GO:0002683 | biological process | negative regulation of immune system process | 7/59 | 463/18866 | 5.81E-04 | 2.61E-03 | 1.12E-03 | CASP3/IL2/JAK3/PIK3R1/PPARG/RARA/TGFB2 | 7 |
| GO:0052547 | biological process | regulation of peptidase activity | 7/59 | 466/18866 | 6.03E-04 | 2.71E-03 | 1.16E-03 | JAK2/MAPK14/MDM2/MMP9/PPARG/SRC/XIAP | 7 |
| GO:0050670 | biological process | regulation of lymphocyte proliferation | 5/59 | 219/18866 | 6.06E-04 | 2.71E-03 | 1.16E-03 | BST1/CASP3/IGF1/IL2/JAK3 | 5 |
| GO:0043124 | biological process | negative regulation of I-kappaB kinase/NF-kappaB signaling | 3/59 | 53/18866 | 6.09E-04 | 2.72E-03 | 1.16E-03 | ESR1/GSTP1/STAT1 | 3 |
| GO:0061614 | biological process | pri-miRNA transcription by RNA polymerase II | 3/59 | 53/18866 | 6.09E-04 | 2.72E-03 | 1.16E-03 | BMP2/PPARG/TGFB2 | 3 |
| GO:0002688 | biological process | regulation of leukocyte chemotaxis | 4/59 | 124/18866 | 6.12E-04 | 2.73E-03 | 1.17E-03 | BST1/MAPK1/MAPK14/RAC1 | 4 |
| GO:0002551 | biological process | mast cell chemotaxis | 2/59 | 12/18866 | 6.22E-04 | 2.73E-03 | 1.17E-03 | KIT/RAC1 | 2 |
| GO:0035630 | biological process | bone mineralization involved in bone maturation | 2/59 | 12/18866 | 6.22E-04 | 2.73E-03 | 1.17E-03 | BMP2/IGF1 | 2 |
| GO:0038110 | biological process | interleukin-2-mediated signaling pathway | 2/59 | 12/18866 | 6.22E-04 | 2.73E-03 | 1.17E-03 | IL2/JAK3 | 2 |
| GO:0060442 | biological process | branching involved in prostate gland morphogenesis | 2/59 | 12/18866 | 6.22E-04 | 2.73E-03 | 1.17E-03 | ESR1/FGFR2 | 2 |
| GO:0060742 | biological process | epithelial cell differentiation involved in prostate gland development | 2/59 | 12/18866 | 6.22E-04 | 2.73E-03 | 1.17E-03 | AR/FGFR2 | 2 |
| GO:0072584 | biological process | caveolin-mediated endocytosis | 2/59 | 12/18866 | 6.22E-04 | 2.73E-03 | 1.17E-03 | MAPK1/SRC | 2 |
| GO:0097284 | biological process | hepatocyte apoptotic process | 2/59 | 12/18866 | 6.22E-04 | 2.73E-03 | 1.17E-03 | BCL2L1/PIK3CG | 2 |
| GO:1902337 | biological process | regulation of apoptotic process involved in morphogenesis | 2/59 | 12/18866 | 6.22E-04 | 2.73E-03 | 1.17E-03 | TGFB2/VDR | 2 |
| GO:1904181 | biological process | positive regulation of membrane depolarization | 2/59 | 12/18866 | 6.22E-04 | 2.73E-03 | 1.17E-03 | KDR/PARP1 | 2 |
| GO:1905461 | biological process | positive regulation of vascular associated smooth muscle cell apoptotic process | 2/59 | 12/18866 | 6.22E-04 | 2.73E-03 | 1.17E-03 | PPARG/SOD2 | 2 |
| GO:2000105 | biological process | positive regulation of DNA-dependent DNA replication | 2/59 | 12/18866 | 6.22E-04 | 2.73E-03 | 1.17E-03 | CDK2/FGFR1 | 2 |
| GO:0032944 | biological process | regulation of mononuclear cell proliferation | 5/59 | 221/18866 | 6.31E-04 | 0.002765495 | 1.18E-03 | BST1/CASP3/IGF1/IL2/JAK3 | 5 |
| GO:0031050 | biological process | dsRNA processing | 3/59 | 54/18866 | 6.43E-04 | 0.002811334 | 1.20E-03 | EGFR/ESR1/MAP2K1 | 3 |
| GO:0070918 | biological process | production of small RNA involved in gene silencing by RNA | 3/59 | 54/18866 | 6.43E-04 | 0.002811334 | 1.20E-03 | EGFR/ESR1/MAP2K1 | 3 |
| GO:0060070 | biological process | canonical Wnt signaling pathway | 6/59 | 339/18866 | 6.51E-04 | 0.002840518 | 1.22E-03 | BMP2/EGFR/FGFR2/MAPK14/SRC/XIAP | 6 |
| GO:1990778 | biological process | protein localization to cell periphery | 6/59 | 340/18866 | 6.61E-04 | 0.002880481 | 1.23E-03 | AKT2/AR/BCL2L1/EGFR/HRAS/PIK3R1 | 6 |
| GO:0001704 | biological process | formation of primary germ layer | 4/59 | 127/18866 | 6.69E-04 | 0.002912789 | 1.25E-03 | FGFR1/FGFR2/MMP2/MMP9 | 4 |
| GO:0003229 | biological process | ventricular cardiac muscle tissue development | 3/59 | 55/18866 | 6.79E-04 | 0.002945841 | 1.26E-03 | FGFR2/TGFB2/TGFBR1 | 3 |
| GO:0032720 | biological process | negative regulation of tumor necrosis factor production | 3/59 | 55/18866 | 6.79E-04 | 0.002945841 | 1.26E-03 | GSTP1/IGF1/RARA | 3 |
| GO:1903409 | biological process | reactive oxygen species biosynthetic process | 4/59 | 128/18866 | 6.89E-04 | 0.002987002 | 0.001278303 | HSP90AA1/INSR/JAK2/RAC1 | 4 |
| GO:2001020 | biological process | regulation of response to DNA damage stimulus | 5/59 | 226/18866 | 6.98E-04 | 0.003020363 | 0.00129258 | BCL2L1/CHEK1/EGFR/MDM2/PARP1 | 5 |
| GO:0002576 | biological process | platelet degranulation | 4/59 | 129/18866 | 7.10E-04 | 0.003066734 | 0.001312425 | ALB/ANXA5/IGF1/TGFB2 | 4 |
| GO:0006977 | biological process | DNA damage response, signal transduction by p53 class mediator resulting in  cell cycle arrest | 3/59 | 56/18866 | 7.16E-04 | 0.003088124 | 0.001321579 | AURKA/CDK2/MDM2 | 3 |
| GO:0002429 | biological process | immune response-activating cell surface receptor signaling pathway | 7/59 | 481/18866 | 7.27E-04 | 0.003120296 | 0.001335347 | HRAS/HSP90AA1/ITK/MAPK1/PIK3R1/RAC1/SRC | 7 |
| GO:0002757 | biological process | immune response-activating signal transduction | 7/59 | 481/18866 | 7.27E-04 | 0.003120296 | 0.001335347 | HRAS/HSP90AA1/ITK/MAPK1/PIK3R1/RAC1/SRC | 7 |
| GO:0002371 | biological process | dendritic cell cytokine production | 2/59 | 13/18866 | 0.000733525 | 0.003120296 | 0.001335347 | JAK3/KIT | 2 |
| GO:0002674 | biological process | negative regulation of acute inflammatory response | 2/59 | 13/18866 | 0.000733525 | 0.003120296 | 0.001335347 | GSTP1/PPARG | 2 |
| GO:0033145 | biological process | positive regulation of intracellular steroid hormone receptor signaling pathway | 2/59 | 13/18866 | 0.000733525 | 0.003120296 | 0.001335347 | AR/PARP1 | 2 |
| GO:0043568 | biological process | positive regulation of insulin-like growth factor receptor signaling pathway | 2/59 | 13/18866 | 0.000733525 | 0.003120296 | 0.001335347 | AR/IGF1 | 2 |
| GO:0060601 | biological process | lateral sprouting from an epithelium | 2/59 | 13/18866 | 0.000733525 | 0.003120296 | 0.001335347 | AR/FGFR2 | 2 |
| GO:0071352 | biological process | cellular response to interleukin-2 | 2/59 | 13/18866 | 0.000733525 | 0.003120296 | 0.001335347 | IL2/JAK3 | 2 |
| GO:0097531 | biological process | mast cell migration | 2/59 | 13/18866 | 0.000733525 | 0.003120296 | 0.001335347 | KIT/RAC1 | 2 |
| GO:2000194 | biological process | regulation of female gonad development | 2/59 | 13/18866 | 0.000733525 | 0.003120296 | 0.001335347 | INSR/SRC | 2 |
| GO:0003231 | biological process | cardiac ventricle development | 4/59 | 131/18866 | 0.000751982 | 0.003180493 | 0.001361109 | FGFR2/MDM2/TGFB2/TGFBR1 | 4 |
| GO:0045747 | biological process | positive regulation of Notch signaling pathway | 3/59 | 57/18866 | 0.000753933 | 0.003180493 | 0.001361109 | KIT/SRC/TGFB2 | 3 |
| GO:0045840 | biological process | positive regulation of mitotic nuclear division | 3/59 | 57/18866 | 0.000753933 | 0.003180493 | 0.001361109 | AURKA/IGF1/INSR | 3 |
| GO:0072431 | biological process | signal transduction involved in mitotic G1 DNA damage checkpoint | 3/59 | 57/18866 | 0.000753933 | 0.003180493 | 0.001361109 | AURKA/CDK2/MDM2 | 3 |
| GO:1902400 | biological process | intracellular signal transduction involved in G1 DNA damage checkpoint | 3/59 | 57/18866 | 0.000753933 | 0.003180493 | 0.001361109 | AURKA/CDK2/MDM2 | 3 |
| GO:1903556 | biological process | negative regulation of tumor necrosis factor superfamily cytokine production | 3/59 | 57/18866 | 0.000753933 | 0.003180493 | 0.001361109 | GSTP1/IGF1/RARA | 3 |
| GO:0050769 | biological process | positive regulation of neurogenesis | 7/59 | 485/18866 | 0.000763689 | 0.003217196 | 0.001376816 | BMP2/FGFR1/IL2/KIT/MAP2K1/PPARG/RARA | 7 |
| GO:0035306 | biological process | positive regulation of dephosphorylation | 3/59 | 58/18866 | 0.000793297 | 0.003332722 | 0.001426256 | BMP2/JAK2/SRC | 3 |
| GO:0043551 | biological process | regulation of phosphatidylinositol 3-kinase activity | 3/59 | 58/18866 | 0.000793297 | 0.003332722 | 0.001426256 | KIT/PIK3R1/SRC | 3 |
| GO:0072175 | biological process | epithelial tube formation | 4/59 | 133/18866 | 0.000795829 | 0.00333876 | 0.00142884 | CASP3/FGFR2/RARA/TGFB2 | 4 |
| GO:0006913 | biological process | nucleocytoplasmic transport | 6/59 | 354/18866 | 0.000815755 | 0.003417656 | 0.001462604 | JAK2/MAPK1/MAPK14/MDM2/PIK3R1/PTPN11 | 6 |
| GO:0045995 | biological process | regulation of embryonic development | 4/59 | 134/18866 | 0.000818417 | 0.003424103 | 0.001465362 | AR/FGFR1/IGF1/INSR | 4 |
| GO:0003002 | biological process | regionalization | 6/59 | 355/18866 | 0.000827788 | 0.003455888 | 0.001478965 | AR/AURKA/BMP2/FGFR1/FGFR2/TGFBR1 | 6 |
| GO:0031102 | biological process | neuron projection regeneration | 3/59 | 59/18866 | 0.000833945 | 0.003455888 | 0.001478965 | CTNNA1/JAK2/MAP2K1 | 3 |
| GO:0043525 | biological process | positive regulation of neuron apoptotic process | 3/59 | 59/18866 | 0.000833945 | 0.003455888 | 0.001478965 | CASP3/NQO2/TGFB2 | 3 |
| GO:0048008 | biological process | platelet-derived growth factor receptor signaling pathway | 3/59 | 59/18866 | 0.000833945 | 0.003455888 | 0.001478965 | JAK2/PTPN11/SRC | 3 |
| GO:0072413 | biological process | signal transduction involved in mitotic cell cycle checkpoint | 3/59 | 59/18866 | 0.000833945 | 0.003455888 | 0.001478965 | AURKA/CDK2/MDM2 | 3 |
| GO:1902402 | biological process | signal transduction involved in mitotic DNA damage checkpoint | 3/59 | 59/18866 | 0.000833945 | 0.003455888 | 0.001478965 | AURKA/CDK2/MDM2 | 3 |
| GO:1902403 | biological process | signal transduction involved in mitotic DNA integrity checkpoint | 3/59 | 59/18866 | 8.34E-04 | 3.46E-03 | 1.48E-03 | AURKA/CDK2/MDM2 | 3 |
| GO:0051169 | biological process | nuclear transport | 6/59 | 357/18866 | 8.52E-04 | 3.51E-03 | 1.50E-03 | JAK2/MAPK1/MAPK14/MDM2/PIK3R1/PTPN11 | 6 |
| GO:0007100 | biological process | mitotic centrosome separation | 2/59 | 14/18866 | 8.54E-04 | 3.51E-03 | 1.50E-03 | AURKA/CHEK1 | 2 |
| GO:0042659 | biological process | regulation of cell fate specification | 2/59 | 14/18866 | 8.54E-04 | 3.51E-03 | 1.50E-03 | AR/FGFR1 | 2 |
| GO:0046321 | biological process | positive regulation of fatty acid oxidation | 2/59 | 14/18866 | 8.54E-04 | 3.51E-03 | 1.50E-03 | AKT2/PPARG | 2 |
| GO:0060397 | biological process | growth hormone receptor signaling pathway via JAK-STAT | 2/59 | 14/18866 | 8.54E-04 | 3.51E-03 | 1.50E-03 | JAK2/JAK3 | 2 |
| GO:0070669 | biological process | response to interleukin-2 | 2/59 | 14/18866 | 8.54E-04 | 3.51E-03 | 1.50E-03 | IL2/JAK3 | 2 |
| GO:1904748 | biological process | regulation of apoptotic process involved in development | 2/59 | 14/18866 | 8.54E-04 | 3.51E-03 | 1.50E-03 | TGFB2/VDR | 2 |
| GO:0050671 | biological process | positive regulation of lymphocyte proliferation | 4/59 | 136/18866 | 8.65E-04 | 3.55E-03 | 1.52E-03 | BST1/IGF1/IL2/JAK3 | 4 |
| GO:0042093 | biological process | T-helper cell differentiation | 3/59 | 60/18866 | 8.76E-04 | 3.58E-03 | 1.53E-03 | IL2/JAK3/RARA | 3 |
| GO:1903428 | biological process | positive regulation of reactive oxygen species biosynthetic process | 3/59 | 60/18866 | 8.76E-04 | 3.58E-03 | 1.53E-03 | HSP90AA1/INSR/JAK2 | 3 |
| GO:0032946 | biological process | positive regulation of mononuclear cell proliferation | 4/59 | 137/18866 | 8.89E-04 | 3.63E-03 | 1.55E-03 | BST1/IGF1/IL2/JAK3 | 4 |
| GO:0032956 | biological process | regulation of actin cytoskeleton organization | 6/59 | 360/18866 | 8.90E-04 | 3.63E-03 | 1.55E-03 | BST1/HRAS/MET/PIK3R1/RAC1/TGFBR1 | 6 |
| GO:0098754 | biological process | detoxification | 4/59 | 138/18866 | 9.13E-04 | 3.72E-03 | 1.59E-03 | ALB/GSTM1/GSTP1/SOD2 | 4 |
| GO:0003170 | biological process | heart valve development | 3/59 | 61/18866 | 9.19E-04 | 3.73E-03 | 1.60E-03 | BMP2/MDM2/TGFB2 | 3 |
| GO:0021872 | biological process | forebrain generation of neurons | 3/59 | 61/18866 | 9.19E-04 | 3.73E-03 | 1.60E-03 | ERBB4/FGFR1/FGFR2 | 3 |
| GO:1904356 | biological process | regulation of telomere maintenance via telomere lengthening | 3/59 | 61/18866 | 9.19E-04 | 3.73E-03 | 1.60E-03 | MAPK1/PARP1/SRC | 3 |
| GO:0002260 | biological process | lymphocyte homeostasis | 3/59 | 62/18866 | 9.64E-04 | 3.88E-03 | 1.66E-03 | CASP3/IL2/JAK3 | 3 |
| GO:0002294 | biological process | CD4-positive, alpha-beta T cell differentiation involved in immune response | 3/59 | 62/18866 | 9.64E-04 | 3.88E-03 | 1.66E-03 | IL2/JAK3/RARA | 3 |
| GO:0007405 | biological process | neuroblast proliferation | 3/59 | 62/18866 | 9.64E-04 | 3.88E-03 | 1.66E-03 | CTNNA1/FGFR1/FGFR2 | 3 |
| GO:0030888 | biological process | regulation of B cell proliferation | 3/59 | 62/18866 | 9.64E-04 | 3.88E-03 | 1.66E-03 | BST1/CASP3/IL2 | 3 |
| GO:0060393 | biological process | regulation of pathway-restricted SMAD protein phosphorylation | 3/59 | 62/18866 | 9.64E-04 | 3.88E-03 | 1.66E-03 | BMP2/TGFB2/TGFBR1 | 3 |
| GO:0030238 | biological process | male sex determination | 2/59 | 15/18866 | 9.83E-04 | 3.92E-03 | 1.68E-03 | AR/INSR | 2 |
| GO:0035635 | biological process | entry of bacterium into host cell | 2/59 | 15/18866 | 9.83E-04 | 3.92E-03 | 1.68E-03 | MET/SRC | 2 |
| GO:0048308 | biological process | organelle inheritance | 2/59 | 15/18866 | 9.83E-04 | 3.92E-03 | 1.68E-03 | MAP2K1/MAPK1 | 2 |
| GO:0048313 | biological process | Golgi inheritance | 2/59 | 15/18866 | 9.83E-04 | 3.92E-03 | 1.68E-03 | MAP2K1/MAPK1 | 2 |
| GO:0051299 | biological process | centrosome separation | 2/59 | 15/18866 | 9.83E-04 | 3.92E-03 | 1.68E-03 | AURKA/CHEK1 | 2 |
| GO:0055057 | biological process | neuroblast division | 2/59 | 15/18866 | 9.83E-04 | 3.92E-03 | 1.68E-03 | FGFR1/FGFR2 | 2 |
| GO:1905065 | biological process | positive regulation of vascular associated smooth muscle cell differentiation | 2/59 | 15/18866 | 9.83E-04 | 3.92E-03 | 1.68E-03 | KIT/SOD2 | 2 |
| GO:2001044 | biological process | regulation of integrin-mediated signaling pathway | 2/59 | 15/18866 | 9.83E-04 | 3.92E-03 | 1.68E-03 | BST1/CTNNA1 | 2 |
| GO:0030098 | biological process | lymphocyte differentiation | 6/59 | 368/18866 | 9.97E-04 | 3.97E-03 | 1.70E-03 | IL2/ITK/JAK3/KIT/PIK3R1/RARA | 6 |
| GO:0002287 | biological process | alpha-beta T cell activation involved in immune response | 3/59 | 63/18866 | 1.01E-03 | 3.99E-03 | 1.71E-03 | IL2/JAK3/RARA | 3 |
| GO:0002293 | biological process | alpha-beta T cell differentiation involved in immune response | 3/59 | 63/18866 | 1.01E-03 | 3.99E-03 | 1.71E-03 | IL2/JAK3/RARA | 3 |
| GO:0031571 | biological process | mitotic G1 DNA damage checkpoint | 3/59 | 63/18866 | 1.01E-03 | 3.99E-03 | 1.71E-03 | AURKA/CDK2/MDM2 | 3 |
| GO:0051893 | biological process | regulation of focal adhesion assembly | 3/59 | 63/18866 | 1.01E-03 | 3.99E-03 | 1.71E-03 | KDR/RAC1/SRC | 3 |
| GO:0090109 | biological process | regulation of cell-substrate junction assembly | 3/59 | 63/18866 | 1.01E-03 | 3.99E-03 | 1.71E-03 | KDR/RAC1/SRC | 3 |
| GO:2000514 | biological process | regulation of CD4-positive, alpha-beta T cell activation | 3/59 | 63/18866 | 1.01E-03 | 3.99E-03 | 1.71E-03 | IL2/JAK3/RARA | 3 |
| GO:0007292 | biological process | female gamete generation | 4/59 | 142/18866 | 1.02E-03 | 4.01E-03 | 1.72E-03 | AURKA/IGF1/PGR/SRC | 4 |
| GO:0045862 | biological process | positive regulation of proteolysis | 6/59 | 370/18866 | 1.03E-03 | 4.04E-03 | 1.73E-03 | AURKA/JAK2/MAPK14/MDM2/PPARG/SRC | 6 |
| GO:0044783 | biological process | G1 DNA damage checkpoint | 3/59 | 64/18866 | 1.06E-03 | 4.14E-03 | 1.77E-03 | AURKA/CDK2/MDM2 | 3 |
| GO:0044819 | biological process | mitotic G1/S transition checkpoint | 3/59 | 64/18866 | 1.06E-03 | 4.14E-03 | 1.77E-03 | AURKA/CDK2/MDM2 | 3 |
| GO:0045453 | biological process | bone resorption | 3/59 | 64/18866 | 1.06E-03 | 4.14E-03 | 1.77E-03 | EGFR/RAC1/SRC | 3 |
| GO:0046637 | biological process | regulation of alpha-beta T cell differentiation | 3/59 | 64/18866 | 1.06E-03 | 4.14E-03 | 1.77E-03 | IL2/JAK3/RARA | 3 |
| GO:0051205 | biological process | protein insertion into membrane | 3/59 | 65/18866 | 1.11E-03 | 4.32E-03 | 1.85E-03 | EGFR/HSP90AA1/MAPK8 | 3 |
| GO:0060389 | biological process | pathway-restricted SMAD protein phosphorylation | 3/59 | 65/18866 | 1.11E-03 | 4.32E-03 | 1.85E-03 | BMP2/TGFB2/TGFBR1 | 3 |
| GO:1905953 | biological process | negative regulation of lipid localization | 3/59 | 65/18866 | 1.11E-03 | 4.32E-03 | 1.85E-03 | AKT2/PPARG/PTPN11 | 3 |
| GO:0002070 | biological process | epithelial cell maturation | 2/59 | 16/18866 | 1.12E-03 | 4.33E-03 | 1.85E-03 | PGR/TYMS | 2 |
| GO:0003222 | biological process | ventricular trabecula myocardium morphogenesis | 2/59 | 16/18866 | 1.12E-03 | 0.004325185 | 1.85E-03 | TGFB2/TGFBR1 | 2 |
| GO:0006206 | biological process | pyrimidine nucleobase metabolic process | 2/59 | 16/18866 | 1.12E-03 | 0.004325185 | 1.85E-03 | MAPK1/TYMS | 2 |
| GO:0048012 | biological process | hepatocyte growth factor receptor signaling pathway | 2/59 | 16/18866 | 1.12E-03 | 0.004325185 | 1.85E-03 | MET/RAC1 | 2 |
| GO:0060572 | biological process | morphogenesis of an epithelial bud | 2/59 | 16/18866 | 1.12E-03 | 0.004325185 | 1.85E-03 | AR/FGFR2 | 2 |
| GO:0060576 | biological process | intestinal epithelial cell development | 2/59 | 16/18866 | 1.12E-03 | 0.004325185 | 1.85E-03 | SRC/TYMS | 2 |
| GO:0071732 | biological process | cellular response to nitric oxide | 2/59 | 16/18866 | 1.12E-03 | 0.004325185 | 1.85E-03 | CCNA2/CDK2 | 2 |
| GO:0090136 | biological process | epithelial cell-cell adhesion | 2/59 | 16/18866 | 1.12E-03 | 0.004325185 | 1.85E-03 | CTNNA1/KIT | 2 |
| GO:1903358 | biological process | regulation of Golgi organization | 2/59 | 16/18866 | 1.12E-03 | 0.004325185 | 0.001850985 | MAP2K1/MAPK1 | 2 |
| GO:1903729 | biological process | regulation of plasma membrane organization | 2/59 | 16/18866 | 1.12E-03 | 0.004325185 | 0.001850985 | AR/TGFB2 | 2 |
| GO:0044344 | biological process | cellular response to fibroblast growth factor stimulus | 4/59 | 146/18866 | 1.13E-03 | 0.004330551 | 0.001853281 | FGFR1/FGFR2/MAPK1/PTPN11 | 4 |
| GO:0046631 | biological process | alpha-beta T cell activation | 4/59 | 146/18866 | 1.13E-03 | 0.004330551 | 0.001853281 | IL2/ITK/JAK3/RARA | 4 |
| GO:0071560 | biological process | cellular response to transforming growth factor beta stimulus | 5/59 | 252/18866 | 1.14E-03 | 0.004366189 | 0.001868533 | FGFR2/PARP1/SRC/TGFB2/TGFBR1 | 5 |
| GO:0051384 | biological process | response to glucocorticoid | 4/59 | 147/18866 | 1.15E-03 | 0.004415858 | 0.001889789 | CASP3/EGFR/GSTP1/TYMS | 4 |
| GO:0032768 | biological process | regulation of monooxygenase activity | 3/59 | 66/18866 | 1.16E-03 | 0.004415858 | 0.001889789 | EGFR/HSP90AA1/VDR | 3 |
| GO:0046626 | biological process | regulation of insulin receptor signaling pathway | 3/59 | 66/18866 | 1.16E-03 | 0.004415858 | 0.001889789 | PIK3R1/PTPN11/SRC | 3 |
| GO:0150116 | biological process | regulation of cell-substrate junction organization | 3/59 | 66/18866 | 1.16E-03 | 0.004415858 | 0.001889789 | KDR/RAC1/SRC | 3 |
| GO:0030217 | biological process | T cell differentiation | 5/59 | 253/18866 | 1.16E-03 | 0.004415858 | 0.001889789 | IL2/ITK/JAK3/KIT/RARA | 5 |
| GO:0019318 | biological process | hexose metabolic process | 5/59 | 254/18866 | 1.18E-03 | 0.004488242 | 0.001920766 | AKT2/IGF1/INSR/MAPK14/SRC | 5 |
| GO:0010675 | biological process | regulation of cellular carbohydrate metabolic process | 4/59 | 148/18866 | 1.18E-03 | 0.004508713 | 0.001929527 | AKT2/IGF1/INSR/SRC | 4 |
| GO:0043550 | biological process | regulation of lipid kinase activity | 3/59 | 67/18866 | 0.001207315 | 0.004585694 | 0.001962471 | KIT/PIK3R1/SRC | 3 |
| GO:0070613 | biological process | regulation of protein processing | 3/59 | 67/18866 | 0.001207315 | 0.004585694 | 0.001962471 | MDM2/SRC/XIAP | 3 |
| GO:0043524 | biological process | negative regulation of neuron apoptotic process | 4/59 | 149/18866 | 0.001213942 | 0.004605126 | 0.001970788 | BCL2L1/HRAS/JAK2/SOD2 | 4 |
| GO:0008016 | biological process | regulation of heart contraction | 5/59 | 256/18866 | 0.001218914 | 0.004618244 | 0.001976401 | IL2/JAK2/MDM2/PIK3CG/TGFB2 | 5 |
| GO:0006606 | biological process | protein import into nucleus | 4/59 | 150/18866 | 0.001244315 | 0.004691177 | 0.002007613 | JAK2/MAPK1/MAPK14/PIK3R1 | 4 |
| GO:0035148 | biological process | tube formation | 4/59 | 150/18866 | 0.001244315 | 0.004691177 | 0.002007613 | CASP3/FGFR2/RARA/TGFB2 | 4 |
| GO:0035264 | biological process | multicellular organism growth | 4/59 | 150/18866 | 0.001244315 | 0.004691177 | 0.002007613 | AR/IGF1/PTPN11/RARA | 4 |
| GO:0090263 | biological process | positive regulation of canonical Wnt signaling pathway | 4/59 | 150/18866 | 0.001244315 | 0.004691177 | 0.002007613 | EGFR/FGFR2/SRC/XIAP | 4 |
| GO:1904888 | biological process | cranial skeletal system development | 3/59 | 68/18866 | 0.001260245 | 0.004730559 | 0.002024467 | FGFR2/TGFB2/TGFBR1 | 3 |
| GO:0071559 | biological process | response to transforming growth factor beta | 5/59 | 258/18866 | 0.001261667 | 0.004730559 | 0.002024467 | FGFR2/PARP1/SRC/TGFB2/TGFBR1 | 5 |
| GO:0003198 | biological process | epithelial to mesenchymal transition involved in endocardial cushion formation | 2/59 | 17/18866 | 0.00126872 | 0.004730559 | 0.002024467 | TGFB2/TGFBR1 | 2 |
| GO:0035855 | biological process | megakaryocyte development | 2/59 | 17/18866 | 0.00126872 | 0.004730559 | 0.002024467 | KIT/PTPN11 | 2 |
| GO:0045623 | biological process | negative regulation of T-helper cell differentiation | 2/59 | 17/18866 | 0.00126872 | 0.004730559 | 0.002024467 | IL2/JAK3 | 2 |
| GO:0060263 | biological process | regulation of respiratory burst | 2/59 | 17/18866 | 0.00126872 | 0.004730559 | 0.002024467 | INSR/RAC1 | 2 |
| GO:0060644 | biological process | mammary gland epithelial cell differentiation | 2/59 | 17/18866 | 0.00126872 | 0.004730559 | 0.002024467 | AKT2/ERBB4 | 2 |
| GO:0090185 | biological process | negative regulation of kidney development | 2/59 | 17/18866 | 0.00126872 | 0.004730559 | 0.002024467 | MMP9/STAT1 | 2 |
| GO:2001267 | biological process | regulation of cysteine-type endopeptidase activity involved in apoptotic signaling pathway | 2/59 | 17/18866 | 0.00126872 | 0.004730559 | 0.002024467 | JAK2/MMP9 | 2 |
| GO:0021537 | biological process | telencephalon development | 5/59 | 259/18866 | 0.001283456 | 0.004779661 | 0.00204548 | BMP2/CASP3/EGFR/ERBB4/RARA | 5 |
| GO:0007004 | biological process | telomere maintenance via telomerase | 3/59 | 69/18866 | 0.001314616 | 0.004865995 | 0.002082428 | HSP90AA1/MAPK1/SRC | 3 |
| GO:0048662 | biological process | negative regulation of smooth muscle cell proliferation | 3/59 | 69/18866 | 0.001314616 | 0.004865995 | 0.002082428 | GSTP1/PPARG/SOD2 | 3 |
| GO:0071230 | biological process | cellular response to amino acid stimulus | 3/59 | 69/18866 | 0.001314616 | 0.004865995 | 0.002082428 | BCL2L1/EGFR/MMP2 | 3 |
| GO:1903317 | biological process | regulation of protein maturation | 3/59 | 69/18866 | 0.001314616 | 0.004865995 | 0.002082428 | MDM2/SRC/XIAP | 3 |
| GO:2000573 | biological process | positive regulation of DNA biosynthetic process | 3/59 | 69/18866 | 0.001314616 | 0.004865995 | 0.002082428 | HSP90AA1/MAPK1/SRC | 3 |
| GO:0001894 | biological process | tissue homeostasis | 5/59 | 261/18866 | 0.001327869 | 0.004909092 | 0.002100871 | ALB/EGFR/PTPN11/RAC1/SRC | 5 |
| GO:0045834 | biological process | positive regulation of lipid metabolic process | 4/59 | 153/18866 | 0.001338629 | 0.004936904 | 0.002112774 | AKT2/KIT/PPARG/SRC | 4 |
| GO:0071774 | biological process | response to fibroblast growth factor | 4/59 | 153/18866 | 0.001338629 | 0.004936904 | 0.002112774 | FGFR1/FGFR2/MAPK1/PTPN11 | 4 |
| GO:0010839 | biological process | negative regulation of keratinocyte proliferation | 2/59 | 18/18866 | 0.001424444 | 0.005240208 | 0.002242574 | FGFR2/VDR | 2 |
| GO:0002292 | biological process | T cell differentiation involved in immune response | 3/59 | 71/18866 | 0.001427742 | 0.005240208 | 0.002242574 | IL2/JAK3/RARA | 3 |
| GO:0033692 | biological process | cellular polysaccharide biosynthetic process | 3/59 | 71/18866 | 0.001427742 | 0.005240208 | 0.002242574 | AKT2/IGF1/INSR | 3 |
| GO:0051785 | biological process | positive regulation of nuclear division | 3/59 | 71/18866 | 0.001427742 | 0.005240208 | 0.002242574 | AURKA/IGF1/INSR | 3 |
| GO:0046883 | biological process | regulation of hormone secretion | 5/59 | 267/18866 | 0.001467944 | 0.005374826 | 0.002300184 | EGFR/FGFR1/JAK2/PTPN11/RAC1 | 5 |
| GO:0072331 | biological process | signal transduction by p53 class mediator | 5/59 | 267/18866 | 0.001467944 | 0.005374826 | 0.002300184 | AURKA/CDK2/CHEK1/MAPK14/MDM2 | 5 |
| GO:0045600 | biological process | positive regulation of fat cell differentiation | 3/59 | 72/18866 | 0.001486525 | 0.005429823 | 0.002323721 | BMP2/MAPK14/PPARG | 3 |
| GO:0061515 | biological process | myeloid cell development | 3/59 | 72/18866 | 0.001486525 | 0.005429823 | 0.002323721 | KIT/PTPN11/SRC | 3 |
| GO:1903707 | biological process | negative regulation of hemopoiesis | 4/59 | 158/18866 | 0.001506758 | 0.005497145 | 0.002352531 | IL2/JAK3/PIK3R1/RARA | 4 |
| GO:0050777 | biological process | negative regulation of immune response | 4/59 | 159/18866 | 0.001542069 | 0.005616386 | 0.002403561 | IL2/JAK3/PPARG/TGFB2 | 4 |
| GO:0003208 | biological process | cardiac ventricle morphogenesis | 3/59 | 73/18866 | 0.001546808 | 0.005616386 | 0.002403561 | FGFR2/TGFB2/TGFBR1 | 3 |
| GO:0005977 | biological process | glycogen metabolic process | 3/59 | 73/18866 | 0.001546808 | 0.005616386 | 0.002403561 | AKT2/IGF1/INSR | 3 |
| GO:0051145 | biological process | smooth muscle cell differentiation | 3/59 | 73/18866 | 0.001546808 | 0.005616386 | 0.002403561 | FGFR2/KIT/SOD2 | 3 |
| GO:0030258 | biological process | lipid modification | 5/59 | 271/18866 | 1.57E-03 | 5.68E-03 | 2.43E-03 | AKT2/MAPK14/PIK3CG/PIK3R1/PPARG | 5 |
| GO:0010759 | biological process | positive regulation of macrophage chemotaxis | 2/59 | 19/18866 | 1.59E-03 | 5.72E-03 | 2.45E-03 | MAPK1/MAPK14 | 2 |
| GO:0032930 | biological process | positive regulation of superoxide anion generation | 2/59 | 19/18866 | 1.59E-03 | 5.72E-03 | 2.45E-03 | EGFR/GSTP1 | 2 |
| GO:0034393 | biological process | positive regulation of smooth muscle cell apoptotic process | 2/59 | 19/18866 | 1.59E-03 | 5.72E-03 | 2.45E-03 | PPARG/SOD2 | 2 |
| GO:0060602 | biological process | branch elongation of an epithelium | 2/59 | 19/18866 | 1.59E-03 | 5.72E-03 | 2.45E-03 | ESR1/FGFR2 | 2 |
| GO:1902170 | biological process | cellular response to reactive nitrogen species | 2/59 | 19/18866 | 1.59E-03 | 5.72E-03 | 2.45E-03 | CCNA2/CDK2 | 2 |
| GO:2000696 | biological process | regulation of epithelial cell differentiation involved in kidney development | 2/59 | 19/18866 | 1.59E-03 | 5.72E-03 | 2.45E-03 | MMP9/STAT1 | 2 |
| GO:0006073 | biological process | cellular glucan metabolic process | 3/59 | 74/18866 | 1.61E-03 | 5.78E-03 | 2.47E-03 | AKT2/IGF1/INSR | 3 |
| GO:0044042 | biological process | glucan metabolic process | 3/59 | 74/18866 | 1.61E-03 | 5.78E-03 | 2.47E-03 | AKT2/IGF1/INSR | 3 |
| GO:0000723 | biological process | telomere maintenance | 4/59 | 161/18866 | 1.61E-03 | 5.79E-03 | 2.48E-03 | HSP90AA1/MAPK1/PARP1/SRC | 4 |
| GO:0044839 | biological process | cell cycle G2/M phase transition | 5/59 | 273/18866 | 1.62E-03 | 5.80E-03 | 2.48E-03 | AURKA/CCNA2/CDK2/CHEK1/HSP90AA1 | 5 |
| GO:0032970 | biological process | regulation of actin filament-based process | 6/59 | 405/18866 | 1.63E-03 | 5.82E-03 | 2.49E-03 | BST1/HRAS/MET/PIK3R1/RAC1/TGFBR1 | 6 |
| GO:0015909 | biological process | long-chain fatty acid transport | 3/59 | 75/18866 | 1.67E-03 | 5.97E-03 | 2.56E-03 | AKT2/PLA2G2A/PPARG | 3 |
| GO:1900076 | biological process | regulation of cellular response to insulin stimulus | 3/59 | 75/18866 | 1.67E-03 | 5.97E-03 | 2.56E-03 | PIK3R1/PTPN11/SRC | 3 |
| GO:0007088 | biological process | regulation of mitotic nuclear division | 4/59 | 163/18866 | 1.69E-03 | 6.03E-03 | 2.58E-03 | AURKA/CHEK1/IGF1/INSR | 4 |
| GO:0006278 | biological process | RNA-dependent DNA biosynthetic process | 3/59 | 76/18866 | 1.74E-03 | 6.18E-03 | 2.65E-03 | HSP90AA1/MAPK1/SRC | 3 |
| GO:0071229 | biological process | cellular response to acid chemical | 3/59 | 76/18866 | 1.74E-03 | 6.18E-03 | 2.65E-03 | BCL2L1/EGFR/MMP2 | 3 |
| GO:0043931 | biological process | ossification involved in bone maturation | 2/59 | 20/18866 | 1.76E-03 | 6.23E-03 | 2.66E-03 | BMP2/IGF1 | 2 |
| GO:0045056 | biological process | transcytosis | 2/59 | 20/18866 | 1.76E-03 | 6.23E-03 | 2.66E-03 | IGF1R/SRC | 2 |
| GO:0060965 | biological process | negative regulation of gene silencing by miRNA | 2/59 | 20/18866 | 1.76E-03 | 6.23E-03 | 2.66E-03 | ESR1/PPARG | 2 |
| GO:0071731 | biological process | response to nitric oxide | 2/59 | 20/18866 | 1.76E-03 | 6.23E-03 | 2.66E-03 | CCNA2/CDK2 | 2 |
| GO:0097062 | biological process | dendritic spine maintenance | 2/59 | 20/18866 | 1.76E-03 | 6.23E-03 | 2.66E-03 | IGF1R/INSR | 2 |
| GO:1905939 | biological process | regulation of gonad development | 2/59 | 20/18866 | 1.76E-03 | 6.23E-03 | 2.66E-03 | INSR/SRC | 2 |
| GO:1903201 | biological process | regulation of oxidative stress-induced cell death | 3/59 | 77/18866 | 1.80E-03 | 6.37E-03 | 2.72E-03 | MET/PARP1/SOD2 | 3 |
| GO:0072659 | biological process | protein localization to plasma membrane | 5/59 | 281/18866 | 1.84E-03 | 6.48E-03 | 2.77E-03 | AKT2/AR/BCL2L1/EGFR/PIK3R1 | 5 |
| GO:0042129 | biological process | regulation of T cell proliferation | 4/59 | 167/18866 | 1.85E-03 | 6.50E-03 | 2.78E-03 | CASP3/IGF1/IL2/JAK3 | 4 |
| GO:0010639 | biological process | negative regulation of organelle organization | 6/59 | 416/18866 | 1.86E-03 | 6.55E-03 | 2.80E-03 | BCL2L1/CHEK1/IGF1/MET/PARP1/SRC | 6 |
| GO:0000271 | biological process | polysaccharide biosynthetic process | 3/59 | 78/18866 | 1.87E-03 | 6.57E-03 | 2.81E-03 | AKT2/IGF1/INSR | 3 |
| GO:0043367 | biological process | CD4-positive, alpha-beta T cell differentiation | 3/59 | 78/18866 | 1.87E-03 | 6.57E-03 | 2.81E-03 | IL2/JAK3/RARA | 3 |
| GO:0016579 | biological process | protein deubiquitination | 5/59 | 283/18866 | 1.89E-03 | 6.64E-03 | 2.84E-03 | AR/CCNA2/ESR1/MDM2/TGFBR1 | 5 |
| GO:0007411 | biological process | axon guidance | 5/59 | 284/18866 | 1.92E-03 | 6.74E-03 | 2.88E-03 | MAPK1/PIK3R1/PTPN11/RAC1/SRC | 5 |
| GO:0051100 | biological process | negative regulation of binding | 4/59 | 169/18866 | 1.93E-03 | 6.74E-03 | 2.89E-03 | AURKA/JAK2/MAPK8/MET | 4 |
| GO:0090398 | biological process | cellular senescence | 3/59 | 79/18866 | 1.94E-03 | 6.75E-03 | 2.89E-03 | HRAS/MAP2K1/MAPK14 | 3 |
| GO:0030220 | biological process | platelet formation | 2/59 | 21/18866 | 1.94E-03 | 6.75E-03 | 2.89E-03 | CASP3/PTPN11 | 2 |
| GO:0030878 | biological process | thyroid gland development | 2/59 | 21/18866 | 1.94E-03 | 6.75E-03 | 2.89E-03 | MAP2K1/MAPK1 | 2 |
| GO:0043371 | biological process | negative regulation of CD4-positive, alpha-beta T cell differentiation | 2/59 | 21/18866 | 1.94E-03 | 6.75E-03 | 2.89E-03 | IL2/JAK3 | 2 |
| GO:0072111 | biological process | cell proliferation involved in kidney development | 2/59 | 21/18866 | 1.94E-03 | 6.75E-03 | 2.89E-03 | BMP2/STAT1 | 2 |
| GO:0090201 | biological process | negative regulation of release of cytochrome c from mitochondria | 2/59 | 21/18866 | 1.94E-03 | 6.75E-03 | 2.89E-03 | BCL2L1/IGF1 | 2 |
| GO:0097485 | biological process | neuron projection guidance | 5/59 | 285/18866 | 1.95E-03 | 6.78E-03 | 2.90E-03 | MAPK1/PIK3R1/PTPN11/RAC1/SRC | 5 |
| GO:0043542 | biological process | endothelial cell migration | 5/59 | 286/18866 | 1.98E-03 | 6.87E-03 | 2.94E-03 | FGFR1/KDR/MET/PPARG/TGFBR1 | 5 |
| GO:0140014 | biological process | mitotic nuclear division | 5/59 | 286/18866 | 1.98E-03 | 6.87E-03 | 2.94E-03 | AURKA/CHEK1/IGF1/INSR/XIAP | 5 |
| GO:0051170 | biological process | import into nucleus | 4/59 | 171/18866 | 2.01E-03 | 6.92E-03 | 2.96E-03 | JAK2/MAPK1/MAPK14/PIK3R1 | 4 |
| GO:0051302 | biological process | regulation of cell division | 4/59 | 171/18866 | 2.01E-03 | 6.92E-03 | 2.96E-03 | AURKA/BCL2L1/FGFR2/TGFB2 | 4 |
| GO:0003151 | biological process | outflow tract morphogenesis | 3/59 | 80/18866 | 2.01E-03 | 6.92E-03 | 2.96E-03 | FGFR2/RARA/TGFB2 | 3 |
| GO:0032204 | biological process | regulation of telomere maintenance | 3/59 | 80/18866 | 2.01E-03 | 6.92E-03 | 2.96E-03 | MAPK1/PARP1/SRC | 3 |
| GO:0048678 | biological process | response to axon injury | 3/59 | 80/18866 | 2.01E-03 | 6.92E-03 | 2.96E-03 | CTNNA1/JAK2/MAP2K1 | 3 |
| GO:0000082 | biological process | G1/S transition of mitotic cell cycle | 5/59 | 287/18866 | 2.01E-03 | 6.92E-03 | 2.96E-03 | AURKA/CDK2/EGFR/MDM2/TYMS | 5 |
| GO:0060047 | biological process | heart contraction | 5/59 | 287/18866 | 2.01E-03 | 6.92E-03 | 2.96E-03 | IL2/JAK2/MDM2/PIK3CG/TGFB2 | 5 |
| GO:0001937 | biological process | negative regulation of endothelial cell proliferation | 3/59 | 81/18866 | 2.08E-03 | 7.14E-03 | 3.06E-03 | PPARG/STAT1/TGFBR1 | 3 |
| GO:0071158 | biological process | positive regulation of cell cycle arrest | 3/59 | 81/18866 | 2.08E-03 | 7.14E-03 | 3.06E-03 | AURKA/CDK2/MDM2 | 3 |
| GO:0032928 | biological process | regulation of superoxide anion generation | 2/59 | 22/18866 | 2.13E-03 | 7.25E-03 | 3.10E-03 | EGFR/GSTP1 | 2 |
| GO:0036120 | biological process | cellular response to platelet-derived growth factor stimulus | 2/59 | 22/18866 | 2.13E-03 | 7.25E-03 | 3.10E-03 | CCNA2/SRC | 2 |
| GO:0036344 | biological process | platelet morphogenesis | 2/59 | 22/18866 | 2.13E-03 | 7.25E-03 | 3.10E-03 | CASP3/PTPN11 | 2 |
| GO:0042359 | biological process | vitamin D metabolic process | 2/59 | 22/18866 | 2.13E-03 | 7.25E-03 | 3.10E-03 | FGFR1/VDR | 2 |
| GO:0051152 | biological process | positive regulation of smooth muscle cell differentiation | 2/59 | 22/18866 | 2.13E-03 | 7.25E-03 | 3.10E-03 | KIT/SOD2 | 2 |
| GO:1901685 | biological process | glutathione derivative metabolic process | 2/59 | 22/18866 | 2.13E-03 | 7.25E-03 | 3.10E-03 | GSTM1/GSTP1 | 2 |
| GO:1901687 | biological process | glutathione derivative biosynthetic process | 2/59 | 22/18866 | 2.13E-03 | 7.25E-03 | 3.10E-03 | GSTM1/GSTP1 | 2 |
| GO:0032200 | biological process | telomere organization | 4/59 | 174/18866 | 2.14E-03 | 0.007279472 | 3.12E-03 | HSP90AA1/MAPK1/PARP1/SRC | 4 |
| GO:0048041 | biological process | focal adhesion assembly | 3/59 | 83/18866 | 2.24E-03 | 0.007582958 | 3.25E-03 | KDR/RAC1/SRC | 3 |
| GO:0051146 | biological process | striated muscle cell differentiation | 5/59 | 295/18866 | 2.27E-03 | 0.007692538 | 3.29E-03 | BMP2/CASP3/IGF1/MAPK14/RARA | 5 |
| GO:0005996 | biological process | monosaccharide metabolic process | 5/59 | 296/18866 | 2.30E-03 | 0.007772938 | 3.33E-03 | AKT2/IGF1/INSR/MAPK14/SRC | 5 |
| GO:0051348 | biological process | negative regulation of transferase activity | 5/59 | 296/18866 | 2.30E-03 | 0.007772938 | 3.33E-03 | CASP3/GSTP1/IGF1R/PPARG/SRC | 5 |
| GO:0007015 | biological process | actin filament organization | 6/59 | 434/18866 | 2.30E-03 | 0.007772938 | 3.33E-03 | JAK2/MET/PIK3R1/RAC1/SRC/TGFBR1 | 6 |
| GO:0052548 | biological process | regulation of endopeptidase activity | 6/59 | 434/18866 | 2.30E-03 | 0.007772938 | 3.33E-03 | JAK2/MDM2/MMP9/PPARG/SRC/XIAP | 6 |
| GO:0003283 | biological process | atrial septum development | 2/59 | 23/18866 | 2.33E-03 | 0.007790079 | 3.33E-03 | MDM2/TGFB2 | 2 |
| GO:0031281 | biological process | positive regulation of cyclase activity | 2/59 | 23/18866 | 2.33E-03 | 0.007790079 | 3.33E-03 | MAPK14/MAPK8 | 2 |
| GO:0035162 | biological process | embryonic hemopoiesis | 2/59 | 23/18866 | 2.33E-03 | 0.007790079 | 3.33E-03 | KDR/KIT | 2 |
| GO:0036119 | biological process | response to platelet-derived growth factor | 2/59 | 23/18866 | 2.33E-03 | 0.007790079 | 3.33E-03 | CCNA2/SRC | 2 |
| GO:0043586 | biological process | tongue development | 2/59 | 23/18866 | 2.33E-03 | 0.007790079 | 0.003333804 | EGFR/KIT | 2 |
| GO:0060149 | biological process | negative regulation of posttranscriptional gene silencing | 2/59 | 23/18866 | 2.33E-03 | 0.007790079 | 0.003333804 | ESR1/PPARG | 2 |
| GO:0060445 | biological process | branching involved in salivary gland morphogenesis | 2/59 | 23/18866 | 2.33E-03 | 0.007790079 | 0.003333804 | FGFR1/FGFR2 | 2 |
| GO:0060967 | biological process | negative regulation of gene silencing by RNA | 2/59 | 23/18866 | 2.33E-03 | 0.007790079 | 0.003333804 | ESR1/PPARG | 2 |
| GO:0070977 | biological process | bone maturation | 2/59 | 23/18866 | 2.33E-03 | 0.007790079 | 0.003333804 | BMP2/IGF1 | 2 |
| GO:0071356 | biological process | cellular response to tumor necrosis factor | 5/59 | 297/18866 | 2.34E-03 | 0.007799764 | 0.003337949 | GSTP1/JAK2/MAPK1/MAPK14/STAT1 | 5 |
| GO:0070646 | biological process | protein modification by small protein removal | 5/59 | 300/18866 | 2.44E-03 | 0.008137544 | 0.003482504 | AR/CCNA2/ESR1/MDM2/TGFBR1 | 5 |
| GO:0048736 | biological process | appendage development | 4/59 | 181/18866 | 2.47E-03 | 0.00818993 | 0.003504923 | FGFR1/FGFR2/RARA/TGFB2 | 4 |
| GO:0060173 | biological process | limb development | 4/59 | 181/18866 | 2.47E-03 | 0.00818993 | 0.003504923 | FGFR1/FGFR2/RARA/TGFB2 | 4 |
| GO:0001776 | biological process | leukocyte homeostasis | 3/59 | 86/18866 | 2.47E-03 | 0.00818993 | 0.003504923 | CASP3/IL2/JAK3 | 3 |
| GO:0006112 | biological process | energy reserve metabolic process | 3/59 | 86/18866 | 2.47E-03 | 0.00818993 | 0.003504923 | AKT2/IGF1/INSR | 3 |
| GO:0034637 | biological process | cellular carbohydrate biosynthetic process | 3/59 | 86/18866 | 2.47E-03 | 0.00818993 | 0.003504923 | AKT2/IGF1/INSR | 3 |
| GO:0070664 | biological process | negative regulation of leukocyte proliferation | 3/59 | 86/18866 | 2.47E-03 | 0.00818993 | 0.003504923 | CASP3/GSTP1/IL2 | 3 |
| GO:0002719 | biological process | negative regulation of cytokine production involved in immune response | 2/59 | 24/18866 | 2.54E-03 | 0.008335155 | 0.003567072 | JAK3/TGFB2 | 2 |
| GO:0003272 | biological process | endocardial cushion formation | 2/59 | 24/18866 | 0.002538806 | 0.008335155 | 0.003567072 | TGFB2/TGFBR1 | 2 |
| GO:0010869 | biological process | regulation of receptor biosynthetic process | 2/59 | 24/18866 | 0.002538806 | 0.008335155 | 0.003567072 | JAK2/PPARG | 2 |
| GO:0021697 | biological process | cerebellar cortex formation | 2/59 | 24/18866 | 0.002538806 | 0.008335155 | 0.003567072 | MAP2K1/PTPN11 | 2 |
| GO:0032331 | biological process | negative regulation of chondrocyte differentiation | 2/59 | 24/18866 | 0.002538806 | 0.008335155 | 0.003567072 | PTPN11/TGFBR1 | 2 |
| GO:0045821 | biological process | positive regulation of glycolytic process | 2/59 | 24/18866 | 0.002538806 | 0.008335155 | 0.003567072 | IGF1/INSR | 2 |
| GO:0046628 | biological process | positive regulation of insulin receptor signaling pathway | 2/59 | 24/18866 | 0.002538806 | 0.008335155 | 0.003567072 | PTPN11/SRC | 2 |
| GO:2000637 | biological process | positive regulation of gene silencing by miRNA | 2/59 | 24/18866 | 0.002538806 | 0.008335155 | 0.003567072 | EGFR/MAP2K1 | 2 |
| GO:0001942 | biological process | hair follicle development | 3/59 | 87/18866 | 0.002555764 | 0.008372805 | 0.003583185 | EGFR/FGFR2/TGFB2 | 3 |
| GO:0097581 | biological process | lamellipodium organization | 3/59 | 87/18866 | 0.002555764 | 0.008372805 | 0.003583185 | KIT/RAC1/SRC | 3 |
| GO:0120032 | biological process | regulation of plasma membrane bounded cell projection assembly | 4/59 | 183/18866 | 0.002572739 | 0.008419371 | 0.003603113 | HRAS/KIT/RAC1/TGFBR1 | 4 |
| GO:1901342 | biological process | regulation of vasculature development | 6/59 | 444/18866 | 0.002580665 | 0.008436256 | 0.003610339 | KDR/KIT/PPARG/SOD2/STAT1/TGFB2 | 6 |
| GO:0046328 | biological process | regulation of JNK cascade | 4/59 | 184/18866 | 0.002623841 | 0.008568219 | 0.003666813 | EGFR/GSTP1/HRAS/IGF1R | 4 |
| GO:2000045 | biological process | regulation of G1/S transition of mitotic cell cycle | 4/59 | 185/18866 | 0.002675638 | 0.008728017 | 0.003735199 | AURKA/CDK2/EGFR/MDM2 | 4 |
| GO:0022404 | biological process | molting cycle process | 3/59 | 89/18866 | 0.002726202 | 0.008873978 | 0.003797664 | EGFR/FGFR2/TGFB2 | 3 |
| GO:0022405 | biological process | hair cycle process | 3/59 | 89/18866 | 0.002726202 | 0.008873978 | 0.003797664 | EGFR/FGFR2/TGFB2 | 3 |
| GO:0007530 | biological process | sex determination | 2/59 | 25/18866 | 0.002754039 | 0.008888698 | 0.003803964 | AR/INSR | 2 |
| GO:0046639 | biological process | negative regulation of alpha-beta T cell differentiation | 2/59 | 25/18866 | 0.002754039 | 0.008888698 | 0.003803964 | IL2/JAK3 | 2 |
| GO:0046697 | biological process | decidualization | 2/59 | 25/18866 | 0.002754039 | 0.008888698 | 0.003803964 | MAPK1/VDR | 2 |
| GO:0048169 | biological process | regulation of long-term neuronal synaptic plasticity | 2/59 | 25/18866 | 0.002754039 | 0.008888698 | 0.003803964 | HRAS/KIT | 2 |
| GO:0051894 | biological process | positive regulation of focal adhesion assembly | 2/59 | 25/18866 | 0.002754039 | 0.008888698 | 0.003803964 | KDR/RAC1 | 2 |
| GO:0060148 | biological process | positive regulation of posttranscriptional gene silencing | 2/59 | 25/18866 | 0.002754039 | 0.008888698 | 0.003803964 | EGFR/MAP2K1 | 2 |
| GO:0060575 | biological process | intestinal epithelial cell differentiation | 2/59 | 25/18866 | 0.002754039 | 0.008888698 | 0.003803964 | SRC/TYMS | 2 |
| GO:2000679 | biological process | positive regulation of transcription regulatory region DNA binding | 2/59 | 25/18866 | 0.002754039 | 0.008888698 | 0.003803964 | IGF1/PARP1 | 2 |
| GO:0098773 | biological process | skin epidermis development | 3/59 | 90/18866 | 0.00281399 | 0.009072588 | 0.003882661 | EGFR/FGFR2/TGFB2 | 3 |
| GO:0051783 | biological process | regulation of nuclear division | 4/59 | 188/18866 | 0.002835239 | 0.009131444 | 0.003907848 | AURKA/CHEK1/IGF1/INSR | 4 |
| GO:0007369 | biological process | gastrulation | 4/59 | 189/18866 | 0.002889858 | 0.009292433 | 0.003976744 | FGFR1/FGFR2/MMP2/MMP9 | 4 |
| GO:0001843 | biological process | neural tube closure | 3/59 | 91/18866 | 0.002903504 | 0.009292433 | 0.003976744 | CASP3/RARA/TGFB2 | 3 |
| GO:0046849 | biological process | bone remodeling | 3/59 | 91/18866 | 0.002903504 | 0.009292433 | 0.003976744 | EGFR/RAC1/SRC | 3 |
| GO:0048477 | biological process | oogenesis | 3/59 | 91/18866 | 0.002903504 | 0.009292433 | 0.003976744 | AURKA/IGF1/SRC | 3 |
| GO:0051899 | biological process | membrane depolarization | 3/59 | 91/18866 | 0.002903504 | 0.009292433 | 0.003976744 | KDR/PARP1/SRC | 3 |
| GO:0060333 | biological process | interferon-gamma-mediated signaling pathway | 3/59 | 91/18866 | 0.002903504 | 0.009292433 | 0.003976744 | JAK2/PPARG/STAT1 | 3 |
| GO:0006575 | biological process | cellular modified amino acid metabolic process | 4/59 | 190/18866 | 0.002945195 | 0.009406122 | 0.004025398 | GSTM1/GSTP1/PLA2G2A/TYMS | 4 |
| GO:0070507 | biological process | regulation of microtubule cytoskeleton organization | 4/59 | 190/18866 | 0.002945195 | 0.009406122 | 0.004025398 | AURKA/CHEK1/MET/RAC1 | 4 |
| GO:0046879 | biological process | hormone secretion | 5/59 | 314/18866 | 0.002971095 | 0.009450119 | 0.004044227 | EGFR/FGFR1/JAK2/PTPN11/RAC1 | 5 |
| GO:0035902 | biological process | response to immobilization stress | 2/59 | 26/18866 | 0.002977562 | 0.009450119 | 0.004044227 | MDM2/PPARG | 2 |
| GO:0042104 | biological process | positive regulation of activated T cell proliferation | 2/59 | 26/18866 | 0.002977562 | 0.009450119 | 0.004044227 | IGF1/IL2 | 2 |
| GO:0060037 | biological process | pharyngeal system development | 2/59 | 26/18866 | 0.002977562 | 0.009450119 | 0.004044227 | TGFB2/TGFBR1 | 2 |
| GO:0060561 | biological process | apoptotic process involved in morphogenesis | 2/59 | 26/18866 | 0.002977562 | 0.009450119 | 0.004044227 | TGFB2/VDR | 2 |
| GO:1905523 | biological process | positive regulation of macrophage migration | 2/59 | 26/18866 | 0.002977562 | 0.009450119 | 0.004044227 | MAPK1/MAPK14 | 2 |
| GO:0060606 | biological process | tube closure | 3/59 | 92/18866 | 0.002994758 | 0.009484954 | 0.004059135 | CASP3/RARA/TGFB2 | 3 |
| GO:1900407 | biological process | regulation of cellular response to oxidative stress | 3/59 | 92/18866 | 0.002994758 | 0.009484954 | 0.004059135 | MET/PARP1/SOD2 | 3 |
| GO:0048167 | biological process | regulation of synaptic plasticity | 4/59 | 191/18866 | 0.003001255 | 0.009495672 | 0.004063721 | HRAS/KIT/MAPK1/RARA | 4 |
| GO:0003416 | biological process | endochondral bone growth | 2/59 | 27/18866 | 0.003209322 | 0.010091168 | 0.004318567 | FGFR2/RARA | 2 |
| GO:0048799 | biological process | animal organ maturation | 2/59 | 27/18866 | 0.003209322 | 0.010091168 | 0.004318567 | BMP2/IGF1 | 2 |
| GO:1900078 | biological process | positive regulation of cellular response to insulin stimulus | 2/59 | 27/18866 | 0.003209322 | 0.010091168 | 0.004318567 | PTPN11/SRC | 2 |
| GO:1902175 | biological process | regulation of oxidative stress-induced intrinsic apoptotic signaling pathway | 2/59 | 27/18866 | 0.003209322 | 0.010091168 | 0.004318567 | PARP1/SOD2 | 2 |
| GO:1904357 | biological process | negative regulation of telomere maintenance via telomere lengthening | 2/59 | 27/18866 | 0.003209322 | 0.010091168 | 0.004318567 | PARP1/SRC | 2 |
| GO:1905208 | biological process | negative regulation of cardiocyte differentiation | 2/59 | 27/18866 | 0.003209322 | 0.010091168 | 0.004318567 | BMP2/EGFR | 2 |
| GO:1903725 | biological process | regulation of phospholipid metabolic process | 3/59 | 95/18866 | 0.003279059 | 0.010299824 | 0.004407863 | KIT/PIK3R1/SRC | 3 |
| GO:0050728 | biological process | negative regulation of inflammatory response | 4/59 | 196/18866 | 0.003292547 | 0.010331551 | 0.00442144 | GSTP1/IGF1/IL2/PPARG | 4 |
| GO:0016570 | biological process | histone modification | 6/59 | 468/18866 | 0.003347046 | 0.010491767 | 0.004490005 | AURKA/CCNA2/CDK2/CHEK1/JAK2/MAPK8 | 6 |
| GO:0009914 | biological process | hormone transport | 5/59 | 323/18866 | 0.003352912 | 0.010499366 | 0.004493258 | EGFR/FGFR1/JAK2/PTPN11/RAC1 | 5 |
| GO:0002690 | biological process | positive regulation of leukocyte chemotaxis | 3/59 | 96/18866 | 0.003377375 | 0.010565122 | 0.004521398 | MAPK1/MAPK14/RAC1 | 3 |
| GO:0021846 | biological process | cell proliferation in forebrain | 2/59 | 28/18866 | 0.003449268 | 0.010734968 | 0.004594085 | FGFR1/FGFR2 | 2 |
| GO:0031069 | biological process | hair follicle morphogenesis | 2/59 | 28/18866 | 0.003449268 | 0.010734968 | 0.004594085 | FGFR2/TGFB2 | 2 |
| GO:0032800 | biological process | receptor biosynthetic process | 2/59 | 28/18866 | 0.003449268 | 0.010734968 | 0.004594085 | JAK2/PPARG | 2 |
| GO:0033598 | biological process | mammary gland epithelial cell proliferation | 2/59 | 28/18866 | 0.003449268 | 0.010734968 | 0.004594085 | ESR1/MAPK1 | 2 |
| GO:1905063 | biological process | regulation of vascular associated smooth muscle cell differentiation | 2/59 | 28/18866 | 0.003449268 | 0.010734968 | 0.004594085 | KIT/SOD2 | 2 |
| GO:0032651 | biological process | regulation of interleukin-1 beta production | 3/59 | 97/18866 | 0.003477484 | 0.010778787 | 0.004612837 | GSTP1/IGF1/JAK2 | 3 |
| GO:0035710 | biological process | CD4-positive, alpha-beta T cell activation | 3/59 | 97/18866 | 0.003477484 | 0.010778787 | 0.004612837 | IL2/JAK3/RARA | 3 |
| GO:0042100 | biological process | B cell proliferation | 3/59 | 97/18866 | 0.003477484 | 0.010778787 | 0.004612837 | BST1/CASP3/IL2 | 3 |
| GO:0046634 | biological process | regulation of alpha-beta T cell activation | 3/59 | 97/18866 | 0.003477484 | 0.010778787 | 0.004612837 | IL2/JAK3/RARA | 3 |
| GO:0051346 | biological process | negative regulation of hydrolase activity | 6/59 | 473/18866 | 3.53E-03 | 1.09E-02 | 4.67E-03 | HRAS/MDM2/MMP9/SRC/TGFB2/XIAP | 6 |
| GO:0014020 | biological process | primary neural tube formation | 3/59 | 98/18866 | 3.58E-03 | 1.11E-02 | 4.73E-03 | CASP3/RARA/TGFB2 | 3 |
| GO:0030316 | biological process | osteoclast differentiation | 3/59 | 98/18866 | 3.58E-03 | 1.11E-02 | 4.73E-03 | MAPK14/PIK3R1/SRC | 3 |
| GO:0048285 | biological process | organelle fission | 6/59 | 476/18866 | 3.64E-03 | 1.12E-02 | 4.80E-03 | AURKA/CHEK1/IGF1/INSR/KDR/XIAP | 6 |
| GO:0007179 | biological process | transforming growth factor beta receptor signaling pathway | 4/59 | 202/18866 | 3.67E-03 | 1.13E-02 | 4.84E-03 | PARP1/SRC/TGFB2/TGFBR1 | 4 |
| GO:0007044 | biological process | cell-substrate junction assembly | 3/59 | 99/18866 | 3.68E-03 | 1.13E-02 | 4.84E-03 | KDR/RAC1/SRC | 3 |
| GO:0010717 | biological process | regulation of epithelial to mesenchymal transition | 3/59 | 99/18866 | 3.68E-03 | 1.13E-02 | 4.84E-03 | BMP2/TGFB2/TGFBR1 | 3 |
| GO:0043255 | biological process | regulation of carbohydrate biosynthetic process | 3/59 | 99/18866 | 3.68E-03 | 1.13E-02 | 4.84E-03 | AKT2/IGF1/INSR | 3 |
| GO:0090277 | biological process | positive regulation of peptide hormone secretion | 3/59 | 99/18866 | 3.68E-03 | 1.13E-02 | 4.84E-03 | EGFR/JAK2/RAC1 | 3 |
| GO:0019048 | biological process | modulation by virus of host process | 2/59 | 29/18866 | 3.70E-03 | 1.13E-02 | 4.85E-03 | BCL2L1/INSR | 2 |
| GO:0150117 | biological process | positive regulation of cell-substrate junction organization | 2/59 | 29/18866 | 3.70E-03 | 1.13E-02 | 4.85E-03 | KDR/RAC1 | 2 |
| GO:0008202 | biological process | steroid metabolic process | 5/59 | 332/18866 | 3.77E-03 | 1.15E-02 | 4.94E-03 | BMP2/ESR1/FGFR1/KIT/VDR | 5 |
| GO:0070301 | biological process | cellular response to hydrogen peroxide | 3/59 | 100/18866 | 3.79E-03 | 1.16E-02 | 4.96E-03 | MDM2/MET/SRC | 3 |
| GO:1903708 | biological process | positive regulation of hemopoiesis | 4/59 | 204/18866 | 3.80E-03 | 1.16E-02 | 4.97E-03 | IL2/MAPK14/RARA/STAT1 | 4 |
| GO:0016569 | biological process | covalent chromatin modification | 6/59 | 481/18866 | 3.83E-03 | 1.17E-02 | 5.00E-03 | AURKA/CCNA2/CDK2/CHEK1/JAK2/MAPK8 | 6 |
| GO:0015908 | biological process | fatty acid transport | 3/59 | 101/18866 | 3.90E-03 | 1.18E-02 | 5.07E-03 | AKT2/PLA2G2A/PPARG | 3 |
| GO:0042102 | biological process | positive regulation of T cell proliferation | 3/59 | 101/18866 | 3.90E-03 | 1.18E-02 | 5.07E-03 | IGF1/IL2/JAK3 | 3 |
| GO:0044264 | biological process | cellular polysaccharide metabolic process | 3/59 | 101/18866 | 3.90E-03 | 1.18E-02 | 5.07E-03 | AKT2/IGF1/INSR | 3 |
| GO:0044773 | biological process | mitotic DNA damage checkpoint | 3/59 | 101/18866 | 3.90E-03 | 1.18E-02 | 5.07E-03 | AURKA/CDK2/MDM2 | 3 |
| GO:1902882 | biological process | regulation of response to oxidative stress | 3/59 | 101/18866 | 3.90E-03 | 1.18E-02 | 5.07E-03 | MET/PARP1/SOD2 | 3 |
| GO:0042594 | biological process | response to starvation | 4/59 | 206/18866 | 3.93E-03 | 1.19E-02 | 5.09E-03 | ALB/MAPK1/MAPK8/PPARG | 4 |
| GO:0050852 | biological process | T cell receptor signaling pathway | 4/59 | 206/18866 | 3.93E-03 | 1.19E-02 | 5.09E-03 | HRAS/ITK/MAPK1/PIK3R1 | 4 |
| GO:1902806 | biological process | regulation of cell cycle G1/S phase transition | 4/59 | 206/18866 | 3.93E-03 | 1.19E-02 | 5.09E-03 | AURKA/CDK2/EGFR/MDM2 | 4 |
| GO:0003401 | biological process | axis elongation | 2/59 | 30/18866 | 3.95E-03 | 1.19E-02 | 5.09E-03 | ESR1/FGFR2 | 2 |
| GO:0010758 | biological process | regulation of macrophage chemotaxis | 2/59 | 30/18866 | 3.95E-03 | 1.19E-02 | 5.09E-03 | MAPK1/MAPK14 | 2 |
| GO:0031116 | biological process | positive regulation of microtubule polymerization | 2/59 | 30/18866 | 3.95E-03 | 1.19E-02 | 5.09E-03 | MET/RAC1 | 2 |
| GO:0033688 | biological process | regulation of osteoblast proliferation | 2/59 | 30/18866 | 3.95E-03 | 1.19E-02 | 5.09E-03 | BMP2/FGFR2 | 2 |
| GO:0048147 | biological process | negative regulation of fibroblast proliferation | 2/59 | 30/18866 | 3.95E-03 | 1.19E-02 | 5.09E-03 | GSTP1/PPARG | 2 |
| GO:0061082 | biological process | myeloid leukocyte cytokine production | 2/59 | 30/18866 | 3.95E-03 | 1.19E-02 | 5.09E-03 | KIT/TGFB2 | 2 |
| GO:0090314 | biological process | positive regulation of protein targeting to membrane | 2/59 | 30/18866 | 3.95E-03 | 1.19E-02 | 5.09E-03 | AKT2/HRAS | 2 |
| GO:0034284 | biological process | response to monosaccharide | 4/59 | 207/18866 | 4.00E-03 | 1.20E-02 | 5.14E-03 | CASP3/GSTP1/IGF1R/RAC1 | 4 |
| GO:0150115 | biological process | cell-substrate junction organization | 3/59 | 102/18866 | 4.01E-03 | 1.20E-02 | 5.15E-03 | KDR/RAC1/SRC | 3 |
| GO:1901987 | biological process | regulation of cell cycle phase transition | 6/59 | 486/18866 | 4.03E-03 | 1.21E-02 | 5.17E-03 | AURKA/CDK2/CHEK1/EGFR/HSP90AA1/MDM2 | 6 |
| GO:0098869 | biological process | cellular oxidant detoxification | 3/59 | 103/18866 | 4.12E-03 | 1.23E-02 | 5.28E-03 | ALB/GSTP1/SOD2 | 3 |
| GO:0035303 | biological process | regulation of dephosphorylation | 4/59 | 209/18866 | 4.14E-03 | 1.24E-02 | 5.30E-03 | BMP2/JAK2/SRC/TGFB2 | 4 |
| GO:0006109 | biological process | regulation of carbohydrate metabolic process | 4/59 | 210/18866 | 4.21E-03 | 1.25E-02 | 5.35E-03 | AKT2/IGF1/INSR/SRC | 4 |
| GO:0030100 | biological process | regulation of endocytosis | 4/59 | 210/18866 | 4.21E-03 | 1.25E-02 | 5.35E-03 | INSR/PPARG/RAC1/SRC | 4 |
| GO:0045216 | biological process | cell-cell junction organization | 4/59 | 210/18866 | 4.21E-03 | 1.25E-02 | 5.35E-03 | CTNNA1/SRC/TGFB2/TGFBR1 | 4 |
| GO:0034368 | biological process | protein-lipid complex remodeling | 2/59 | 31/18866 | 4.22E-03 | 1.25E-02 | 5.35E-03 | ALB/PLA2G2A | 2 |
| GO:0034369 | biological process | plasma lipoprotein particle remodeling | 2/59 | 31/18866 | 4.22E-03 | 1.25E-02 | 5.35E-03 | ALB/PLA2G2A | 2 |
| GO:0045737 | biological process | positive regulation of cyclin-dependent protein serine/threonine kinase activity | 2/59 | 31/18866 | 4.22E-03 | 1.25E-02 | 5.35E-03 | EGFR/SRC | 2 |
| GO:0060325 | biological process | face morphogenesis | 2/59 | 31/18866 | 4.22E-03 | 1.25E-02 | 5.35E-03 | MMP2/PTPN11 | 2 |
| GO:0070229 | biological process | negative regulation of lymphocyte apoptotic process | 2/59 | 31/18866 | 4.22E-03 | 1.25E-02 | 5.35E-03 | IL2/JAK3 | 2 |
| GO:0098868 | biological process | bone growth | 2/59 | 31/18866 | 4.22E-03 | 1.25E-02 | 5.35E-03 | FGFR2/RARA | 2 |
| GO:2000515 | biological process | negative regulation of CD4-positive, alpha-beta T cell activation | 2/59 | 31/18866 | 4.22E-03 | 1.25E-02 | 5.35E-03 | IL2/JAK3 | 2 |
| GO:0002367 | biological process | cytokine production involved in immune response | 3/59 | 104/18866 | 4.23E-03 | 1.25E-02 | 5.35E-03 | JAK3/KIT/TGFB2 | 3 |
| GO:0032649 | biological process | regulation of interferon-gamma production | 3/59 | 104/18866 | 4.23E-03 | 1.25E-02 | 5.35E-03 | HRAS/IL2/RARA | 3 |
| GO:0090150 | biological process | establishment of protein localization to membrane | 5/59 | 342/18866 | 4.27E-03 | 1.26E-02 | 5.41E-03 | AKT2/EGFR/HRAS/HSP90AA1/MAPK8 | 5 |
| GO:0002685 | biological process | regulation of leukocyte migration | 4/59 | 212/18866 | 4.35E-03 | 1.28E-02 | 5.50E-03 | BST1/MAPK1/MAPK14/RAC1 | 4 |
| GO:1901215 | biological process | negative regulation of neuron death | 4/59 | 212/18866 | 4.35E-03 | 1.28E-02 | 5.50E-03 | BCL2L1/HRAS/JAK2/SOD2 | 4 |
| GO:0090276 | biological process | regulation of peptide hormone secretion | 4/59 | 213/18866 | 4.43E-03 | 1.30E-02 | 5.58E-03 | EGFR/JAK2/PTPN11/RAC1 | 4 |
| GO:0001841 | biological process | neural tube formation | 3/59 | 106/18866 | 4.46E-03 | 1.31E-02 | 5.60E-03 | CASP3/RARA/TGFB2 | 3 |
| GO:0019395 | biological process | fatty acid oxidation | 3/59 | 106/18866 | 4.46E-03 | 1.31E-02 | 5.60E-03 | AKT2/MAPK14/PPARG | 3 |
| GO:0032091 | biological process | negative regulation of protein binding | 3/59 | 106/18866 | 4.46E-03 | 1.31E-02 | 5.60E-03 | AURKA/MAPK8/MET | 3 |
| GO:0032611 | biological process | interleukin-1 beta production | 3/59 | 106/18866 | 4.46E-03 | 1.31E-02 | 5.60E-03 | GSTP1/IGF1/JAK2 | 3 |
| GO:0034446 | biological process | substrate adhesion-dependent cell spreading | 3/59 | 106/18866 | 4.46E-03 | 1.31E-02 | 5.60E-03 | PIK3R1/RAC1/SRC | 3 |
| GO:0010743 | biological process | regulation of macrophage derived foam cell differentiation | 2/59 | 32/18866 | 4.49E-03 | 1.31E-02 | 5.61E-03 | PLA2G2A/PPARG | 2 |
| GO:0034367 | biological process | protein-containing complex remodeling | 2/59 | 32/18866 | 4.49E-03 | 1.31E-02 | 5.61E-03 | ALB/PLA2G2A | 2 |
| GO:0046320 | biological process | regulation of fatty acid oxidation | 2/59 | 32/18866 | 4.49E-03 | 1.31E-02 | 5.61E-03 | AKT2/PPARG | 2 |
| GO:0048730 | biological process | epidermis morphogenesis | 2/59 | 32/18866 | 4.49E-03 | 0.013104431 | 5.61E-03 | FGFR2/TGFB2 | 2 |
| GO:0051385 | biological process | response to mineralocorticoid | 2/59 | 32/18866 | 4.49E-03 | 0.013104431 | 5.61E-03 | PARP1/SRC | 2 |
| GO:1903706 | biological process | regulation of hemopoiesis | 6/59 | 498/18866 | 4.53E-03 | 0.01321101 | 5.65E-03 | IL2/JAK3/MAPK14/PIK3R1/RARA/STAT1 | 6 |
| GO:0000018 | biological process | regulation of DNA recombination | 3/59 | 107/18866 | 4.58E-03 | 0.013327285 | 5.70E-03 | CHEK1/IL2/PARP1 | 3 |
| GO:0030330 | biological process | DNA damage response, signal transduction by p53 class mediator | 3/59 | 107/18866 | 4.58E-03 | 0.013327285 | 5.70E-03 | AURKA/CDK2/MDM2 | 3 |
| GO:0032526 | biological process | response to retinoic acid | 3/59 | 108/18866 | 4.70E-03 | 0.013638855 | 5.84E-03 | FGFR2/PPARG/RARA | 3 |
| GO:0044774 | biological process | mitotic DNA integrity checkpoint | 3/59 | 108/18866 | 4.70E-03 | 0.013638855 | 5.84E-03 | AURKA/CDK2/MDM2 | 3 |
| GO:0071887 | biological process | leukocyte apoptotic process | 3/59 | 108/18866 | 4.70E-03 | 0.013638855 | 5.84E-03 | CASP3/IL2/JAK3 | 3 |
| GO:1902749 | biological process | regulation of cell cycle G2/M phase transition | 4/59 | 217/18866 | 4.73E-03 | 0.013705403 | 5.87E-03 | AURKA/CDK2/CHEK1/HSP90AA1 | 4 |
| GO:0010955 | biological process | negative regulation of protein processing | 2/59 | 33/18866 | 4.77E-03 | 0.01372495 | 5.87E-03 | MDM2/XIAP | 2 |
| GO:0021696 | biological process | cerebellar cortex morphogenesis | 2/59 | 33/18866 | 4.77E-03 | 0.01372495 | 5.87E-03 | MAP2K1/PTPN11 | 2 |
| GO:0043552 | biological process | positive regulation of phosphatidylinositol 3-kinase activity | 2/59 | 33/18866 | 4.77E-03 | 0.01372495 | 0.005873663 | KIT/SRC | 2 |
| GO:0048384 | biological process | retinoic acid receptor signaling pathway | 2/59 | 33/18866 | 4.77E-03 | 0.01372495 | 0.005873663 | PPARG/RARA | 2 |
| GO:0090022 | biological process | regulation of neutrophil chemotaxis | 2/59 | 33/18866 | 4.77E-03 | 0.01372495 | 0.005873663 | BST1/RAC1 | 2 |
| GO:1902003 | biological process | regulation of amyloid-beta formation | 2/59 | 33/18866 | 4.77E-03 | 0.01372495 | 0.005873663 | CASP3/IGF1 | 2 |
| GO:1902745 | biological process | positive regulation of lamellipodium organization | 2/59 | 33/18866 | 4.77E-03 | 0.01372495 | 0.005873663 | RAC1/SRC | 2 |
| GO:1903318 | biological process | negative regulation of protein maturation | 2/59 | 33/18866 | 4.77E-03 | 0.01372495 | 0.005873663 | MDM2/XIAP | 2 |
| GO:0007623 | biological process | circadian rhythm | 4/59 | 218/18866 | 4.80E-03 | 0.013811273 | 0.005910605 | EGFR/MAPK8/PPARG/TYMS | 4 |
| GO:0002286 | biological process | T cell activation involved in immune response | 3/59 | 109/18866 | 4.82E-03 | 0.013849052 | 0.005926773 | IL2/JAK3/RARA | 3 |
| GO:0007202 | biological process | activation of phospholipase C activity | 2/59 | 34/18866 | 5.06E-03 | 0.014392332 | 0.006159272 | EGFR/ITK | 2 |
| GO:0010922 | biological process | positive regulation of phosphatase activity | 2/59 | 34/18866 | 5.06E-03 | 0.014392332 | 0.006159272 | BMP2/JAK2 | 2 |
| GO:0031112 | biological process | positive regulation of microtubule polymerization or depolymerization | 2/59 | 34/18866 | 5.06E-03 | 0.014392332 | 0.006159272 | MET/RAC1 | 2 |
| GO:0042759 | biological process | long-chain fatty acid biosynthetic process | 2/59 | 34/18866 | 5.06E-03 | 0.014392332 | 0.006159272 | GSTM1/GSTP1 | 2 |
| GO:0045648 | biological process | positive regulation of erythrocyte differentiation | 2/59 | 34/18866 | 0.005057965 | 0.014392332 | 0.006159272 | MAPK14/STAT1 | 2 |
| GO:0048566 | biological process | embryonic digestive tract development | 2/59 | 34/18866 | 0.005057965 | 0.014392332 | 0.006159272 | FGFR2/TGFB2 | 2 |
| GO:0061384 | biological process | heart trabecula morphogenesis | 2/59 | 34/18866 | 0.005057965 | 0.014392332 | 0.006159272 | TGFB2/TGFBR1 | 2 |
| GO:0033044 | biological process | regulation of chromosome organization | 5/59 | 356/18866 | 0.005058674 | 0.014392332 | 0.006159272 | CHEK1/MAPK1/MAPK8/PARP1/SRC | 5 |
| GO:0002526 | biological process | acute inflammatory response | 3/59 | 111/18866 | 0.005072707 | 0.014392332 | 0.006159272 | GSTP1/PIK3CG/PPARG | 3 |
| GO:0032652 | biological process | regulation of interleukin-1 production | 3/59 | 111/18866 | 0.005072707 | 0.014392332 | 0.006159272 | GSTP1/IGF1/JAK2 | 3 |
| GO:0034440 | biological process | lipid oxidation | 3/59 | 111/18866 | 0.005072707 | 0.014392332 | 0.006159272 | AKT2/MAPK14/PPARG | 3 |
| GO:0042303 | biological process | molting cycle | 3/59 | 111/18866 | 0.005072707 | 0.014392332 | 0.006159272 | EGFR/FGFR2/TGFB2 | 3 |
| GO:0042633 | biological process | hair cycle | 3/59 | 111/18866 | 0.005072707 | 0.014392332 | 0.006159272 | EGFR/FGFR2/TGFB2 | 3 |
| GO:0019722 | biological process | calcium-mediated signaling | 4/59 | 222/18866 | 0.00512211 | 0.014518993 | 0.006213478 | BST1/EGFR/IGF1/KDR | 4 |
| GO:0005976 | biological process | polysaccharide metabolic process | 3/59 | 112/18866 | 0.005200857 | 0.014728519 | 0.006303145 | AKT2/IGF1/INSR | 3 |
| GO:0007009 | biological process | plasma membrane organization | 3/59 | 113/18866 | 0.005330948 | 0.015035928 | 0.006434703 | AKT2/AR/TGFB2 | 3 |
| GO:0032963 | biological process | collagen metabolic process | 3/59 | 113/18866 | 0.005330948 | 0.015035928 | 0.006434703 | MMP2/MMP9/PPARG | 3 |
| GO:0009112 | biological process | nucleobase metabolic process | 2/59 | 35/18866 | 0.005353776 | 0.015035928 | 0.006434703 | MAPK1/TYMS | 2 |
| GO:0032633 | biological process | interleukin-4 production | 2/59 | 35/18866 | 0.005353776 | 0.015035928 | 0.006434703 | ITK/RARA | 2 |
| GO:0033687 | biological process | osteoblast proliferation | 2/59 | 35/18866 | 0.005353776 | 0.015035928 | 0.006434703 | BMP2/FGFR2 | 2 |
| GO:0048333 | biological process | mesodermal cell differentiation | 2/59 | 35/18866 | 0.005353776 | 0.015035928 | 0.006434703 | FGFR1/FGFR2 | 2 |
| GO:0060674 | biological process | placenta blood vessel development | 2/59 | 35/18866 | 0.005353776 | 0.015035928 | 0.006434703 | MAP2K1/MAPK1 | 2 |
| GO:0110111 | biological process | negative regulation of animal organ morphogenesis | 2/59 | 35/18866 | 0.005353776 | 0.015035928 | 0.006434703 | STAT1/TGFB2 | 2 |
| GO:1904031 | biological process | positive regulation of cyclin-dependent protein kinase activity | 2/59 | 35/18866 | 0.005353776 | 0.015035928 | 0.006434703 | EGFR/SRC | 2 |
| GO:0001893 | biological process | maternal placenta development | 2/59 | 36/18866 | 0.005657366 | 0.015729232 | 0.006731405 | MAPK1/VDR | 2 |
| GO:0002701 | biological process | negative regulation of production of molecular mediator of immune response | 2/59 | 36/18866 | 0.005657366 | 0.015729232 | 0.006731405 | JAK3/TGFB2 | 2 |
| GO:0003230 | biological process | cardiac atrium development | 2/59 | 36/18866 | 0.005657366 | 0.015729232 | 0.006731405 | MDM2/TGFB2 | 2 |
| GO:0003382 | biological process | epithelial cell morphogenesis | 2/59 | 36/18866 | 0.005657366 | 0.015729232 | 0.006731405 | AR/MET | 2 |
| GO:0032205 | biological process | negative regulation of telomere maintenance | 2/59 | 36/18866 | 0.005657366 | 0.015729232 | 0.006731405 | PARP1/SRC | 2 |
| GO:0042554 | biological process | superoxide anion generation | 2/59 | 36/18866 | 0.005657366 | 0.015729232 | 0.006731405 | EGFR/GSTP1 | 2 |
| GO:0051973 | biological process | positive regulation of telomerase activity | 2/59 | 36/18866 | 0.005657366 | 0.015729232 | 0.006731405 | HSP90AA1/MAPK1 | 2 |
| GO:0060323 | biological process | head morphogenesis | 2/59 | 36/18866 | 0.005657366 | 0.015729232 | 0.006731405 | MMP2/PTPN11 | 2 |
| GO:0090313 | biological process | regulation of protein targeting to membrane | 2/59 | 36/18866 | 0.005657366 | 0.015729232 | 0.006731405 | AKT2/HRAS | 2 |
| GO:1901099 | biological process | negative regulation of signal transduction in absence of ligand | 2/59 | 36/18866 | 0.005657366 | 0.015729232 | 0.006731405 | BCL2L1/CTNNA1 | 2 |
| GO:2001240 | biological process | negative regulation of extrinsic apoptotic signaling pathway in absence of ligand | 2/59 | 36/18866 | 0.005657366 | 0.015729232 | 0.006731405 | BCL2L1/CTNNA1 | 2 |
| GO:0051495 | biological process | positive regulation of cytoskeleton organization | 4/59 | 230/18866 | 0.005799044 | 0.016108454 | 0.006893696 | HRAS/MET/RAC1/TGFBR1 | 4 |
| GO:0030282 | biological process | bone mineralization | 3/59 | 117/18866 | 0.005870875 | 0.016278336 | 0.006966398 | BMP2/FGFR2/IGF1 | 3 |
| GO:0051341 | biological process | regulation of oxidoreductase activity | 3/59 | 117/18866 | 0.005870875 | 0.016278336 | 0.006966398 | EGFR/HSP90AA1/VDR | 3 |
| GO:0043243 | biological process | positive regulation of protein-containing complex disassembly | 2/59 | 37/18866 | 0.005968685 | 0.01647465 | 0.007050411 | IGF1R/INSR | 2 |
| GO:0060428 | biological process | lung epithelium development | 2/59 | 37/18866 | 0.005968685 | 0.01647465 | 0.007050411 | FGFR2/MAP2K1 | 2 |
| GO:0060969 | biological process | negative regulation of gene silencing | 2/59 | 37/18866 | 0.005968685 | 0.01647465 | 0.007050411 | ESR1/PPARG | 2 |
| GO:0090218 | biological process | positive regulation of lipid kinase activity | 2/59 | 37/18866 | 0.005968685 | 0.01647465 | 0.007050411 | KIT/SRC | 2 |
| GO:0097242 | biological process | amyloid-beta clearance | 2/59 | 37/18866 | 0.005968685 | 0.01647465 | 0.007050411 | IGF1R/INSR | 2 |
| GO:0006805 | biological process | xenobiotic metabolic process | 3/59 | 118/18866 | 0.006010787 | 0.016560886 | 0.007087316 | GSTM1/GSTP1/NQO2 | 3 |
| GO:0050868 | biological process | negative regulation of T cell activation | 3/59 | 118/18866 | 0.006010787 | 0.016560886 | 0.007087316 | CASP3/IL2/JAK3 | 3 |
| GO:0009266 | biological process | response to temperature stimulus | 4/59 | 233/18866 | 0.00606758 | 0.016687213 | 0.007141379 | HSP90AA1/IGF1/MAPK1/PPARG | 4 |
| GO:0009743 | biological process | response to carbohydrate | 4/59 | 233/18866 | 0.00606758 | 0.016687213 | 0.007141379 | CASP3/GSTP1/IGF1R/RAC1 | 4 |
| GO:0010822 | biological process | positive regulation of mitochondrion organization | 3/59 | 119/18866 | 0.006152686 | 0.016890813 | 0.00722851 | KDR/MAPK8/MMP9 | 3 |
| GO:0051153 | biological process | regulation of striated muscle cell differentiation | 3/59 | 119/18866 | 0.006152686 | 0.016890813 | 0.00722851 | BMP2/IGF1/MAPK14 | 3 |
| GO:0045444 | biological process | fat cell differentiation | 4/59 | 235/18866 | 0.006251143 | 0.017130266 | 0.007330985 | AKT2/BMP2/MAPK14/PPARG | 4 |
| GO:0071453 | biological process | cellular response to oxygen levels | 4/59 | 235/18866 | 0.006251143 | 0.017130266 | 0.007330985 | CCNA2/MDM2/PPARG/SRC | 4 |
| GO:0010661 | biological process | positive regulation of muscle cell apoptotic process | 2/59 | 38/18866 | 0.006287683 | 0.017184079 | 0.007354015 | PPARG/SOD2 | 2 |
| GO:0044003 | biological process | modulation by symbiont of host process | 2/59 | 38/18866 | 0.006287683 | 0.017184079 | 0.007354015 | BCL2L1/INSR | 2 |
| GO:0045923 | biological process | positive regulation of fatty acid metabolic process | 2/59 | 38/18866 | 0.006287683 | 0.017184079 | 0.007354015 | AKT2/PPARG | 2 |
| GO:0060560 | biological process | developmental growth involved in morphogenesis | 4/59 | 236/18866 | 0.006344298 | 0.017323283 | 0.007413588 | AURKA/ESR1/FGFR2/HSP90AA1 | 4 |
| GO:0032612 | biological process | interleukin-1 production | 3/59 | 121/18866 | 0.006442475 | 0.01754424 | 0.007508148 | GSTP1/IGF1/JAK2 | 3 |
| GO:0120254 | biological process | olefinic compound metabolic process | 3/59 | 121/18866 | 0.006442475 | 0.01754424 | 0.007508148 | BMP2/GSTM1/GSTP1 | 3 |
| GO:1900542 | biological process | regulation of purine nucleotide metabolic process | 3/59 | 121/18866 | 0.006442475 | 0.01754424 | 0.007508148 | IGF1/INSR/PARP1 | 3 |
| GO:0010594 | biological process | regulation of endothelial cell migration | 4/59 | 238/18866 | 0.006533373 | 0.017775905 | 0.00760729 | FGFR1/KDR/MET/PPARG | 4 |
| GO:0010811 | biological process | positive regulation of cell-substrate adhesion | 3/59 | 122/18866 | 0.006590379 | 0.017868597 | 0.007646958 | JAK2/KDR/RAC1 | 3 |
| GO:0021782 | biological process | glial cell development | 3/59 | 122/18866 | 0.006590379 | 0.017868597 | 0.007646958 | AKT2/EGFR/GSTP1 | 3 |
| GO:0010453 | biological process | regulation of cell fate commitment | 2/59 | 39/18866 | 0.00661431 | 0.017868597 | 0.007646958 | AR/FGFR1 | 2 |
| GO:0010837 | biological process | regulation of keratinocyte proliferation | 2/59 | 39/18866 | 0.00661431 | 0.017868597 | 0.007646958 | FGFR2/VDR | 2 |
| GO:0032691 | biological process | negative regulation of interleukin-1 beta production | 2/59 | 39/18866 | 0.00661431 | 0.017868597 | 0.007646958 | GSTP1/IGF1 | 2 |
| GO:0034205 | biological process | amyloid-beta formation | 2/59 | 39/18866 | 0.00661431 | 0.017868597 | 0.007646958 | CASP3/IGF1 | 2 |
| GO:0035886 | biological process | vascular associated smooth muscle cell differentiation | 2/59 | 39/18866 | 0.00661431 | 0.017868597 | 0.007646958 | KIT/SOD2 | 2 |
| GO:2000279 | biological process | negative regulation of DNA biosynthetic process | 2/59 | 39/18866 | 0.00661431 | 0.017868597 | 0.007646958 | PPARG/SRC | 2 |
| GO:0045666 | biological process | positive regulation of neuron differentiation | 5/59 | 380/18866 | 0.006635442 | 0.017909821 | 0.0076646 | BMP2/FGFR1/IL2/MAP2K1/RARA | 5 |
| GO:0032886 | biological process | regulation of microtubule-based process | 4/59 | 240/18866 | 0.006726164 | 0.01812867 | 0.007758257 | AURKA/CHEK1/MET/RAC1 | 4 |
| GO:0006140 | biological process | regulation of nucleotide metabolic process | 3/59 | 123/18866 | 0.006740299 | 0.01812867 | 0.007758257 | IGF1/INSR/PARP1 | 3 |
| GO:0042471 | biological process | ear morphogenesis | 3/59 | 123/18866 | 0.006740299 | 0.01812867 | 0.007758257 | FGFR1/FGFR2/MAPK1 | 3 |
| GO:0071466 | biological process | cellular response to xenobiotic stimulus | 3/59 | 123/18866 | 0.006740299 | 0.01812867 | 0.007758257 | GSTM1/GSTP1/NQO2 | 3 |
| GO:0002698 | biological process | negative regulation of immune effector process | 3/59 | 124/18866 | 0.006892241 | 0.018521001 | 0.007926158 | IL2/JAK3/TGFB2 | 3 |
| GO:0010613 | biological process | positive regulation of cardiac muscle hypertrophy | 2/59 | 40/18866 | 0.006948518 | 0.01857404 | 0.007948856 | IGF1/PARP1 | 2 |
| GO:0021587 | biological process | cerebellum morphogenesis | 2/59 | 40/18866 | 0.006948518 | 0.01857404 | 0.007948856 | MAP2K1/PTPN11 | 2 |
| GO:0046636 | biological process | negative regulation of alpha-beta T cell activation | 2/59 | 40/18866 | 0.006948518 | 0.01857404 | 0.007948856 | IL2/JAK3 | 2 |
| GO:0071526 | biological process | semaphorin-plexin signaling pathway | 2/59 | 40/18866 | 0.006948518 | 0.01857404 | 0.007948856 | MET/RAC1 | 2 |
| GO:1902742 | biological process | apoptotic process involved in development | 2/59 | 40/18866 | 0.006948518 | 0.01857404 | 0.007948856 | TGFB2/VDR | 2 |
| GO:1902991 | biological process | regulation of amyloid precursor protein catabolic process | 2/59 | 40/18866 | 0.006948518 | 0.01857404 | 0.007948856 | CASP3/IGF1 | 2 |
| GO:0001838 | biological process | embryonic epithelial tube formation | 3/59 | 125/18866 | 0.007046213 | 0.018785796 | 0.008039478 | CASP3/RARA/TGFB2 | 3 |
| GO:0007098 | biological process | centrosome cycle | 3/59 | 125/18866 | 0.007046213 | 0.018785796 | 0.008039478 | AURKA/CDK2/CHEK1 | 3 |
| GO:1903578 | biological process | regulation of ATP metabolic process | 3/59 | 125/18866 | 0.007046213 | 0.018785796 | 0.008039478 | IGF1/INSR/PARP1 | 3 |
| GO:0055007 | biological process | cardiac muscle cell differentiation | 3/59 | 126/18866 | 0.007202222 | 0.019168216 | 0.008203136 | BMP2/IGF1/RARA | 3 |
| GO:2000134 | biological process | negative regulation of G1/S transition of mitotic cell cycle | 3/59 | 126/18866 | 0.007202222 | 0.019168216 | 0.008203136 | AURKA/CDK2/MDM2 | 3 |
| GO:0014742 | biological process | positive regulation of muscle hypertrophy | 2/59 | 41/18866 | 0.007290258 | 0.019318233 | 0.008267337 | IGF1/PARP1 | 2 |
| GO:0030890 | biological process | positive regulation of B cell proliferation | 2/59 | 41/18866 | 0.007290258 | 0.019318233 | 0.008267337 | BST1/IL2 | 2 |
| GO:0046825 | biological process | regulation of protein export from nucleus | 2/59 | 41/18866 | 0.007290258 | 0.019318233 | 0.008267337 | MDM2/PTPN11 | 2 |
| GO:0048286 | biological process | lung alveolus development | 2/59 | 41/18866 | 0.007290258 | 0.019318233 | 0.008267337 | FGFR2/PGR | 2 |
| GO:1905209 | biological process | positive regulation of cardiocyte differentiation | 2/59 | 41/18866 | 0.007290258 | 0.019318233 | 0.008267337 | IGF1/TGFB2 | 2 |
| GO:0051053 | biological process | negative regulation of DNA metabolic process | 3/59 | 127/18866 | 0.007360272 | 0.019486832 | 0.00833949 | PARP1/PPARG/SRC | 3 |
| GO:0072089 | biological process | stem cell proliferation | 3/59 | 128/18866 | 0.007520372 | 0.019893438 | 0.008513499 | CTNNA1/FGFR1/FGFR2 | 3 |
| GO:0048246 | biological process | macrophage chemotaxis | 2/59 | 42/18866 | 0.00763948 | 0.020173519 | 0.008633361 | MAPK1/MAPK14 | 2 |
| GO:1902622 | biological process | regulation of neutrophil migration | 2/59 | 42/18866 | 0.00763948 | 0.020173519 | 0.008633361 | BST1/RAC1 | 2 |
| GO:0030198 | biological process | extracellular matrix organization | 5/59 | 395/18866 | 0.007780457 | 0.020528022 | 0.008785072 | KDR/MMP2/MMP9/TGFB2/TGFBR1 | 5 |
| GO:0009410 | biological process | response to xenobiotic stimulus | 3/59 | 130/18866 | 0.007846742 | 0.020685017 | 0.008852259 | GSTM1/GSTP1/NQO2 | 3 |
| GO:0006631 | biological process | fatty acid metabolic process | 5/59 | 396/18866 | 0.007861388 | 0.020687863 | 0.008853477 | AKT2/GSTM1/GSTP1/MAPK14/PPARG | 5 |
| GO:0043062 | biological process | extracellular structure organization | 5/59 | 396/18866 | 0.007861388 | 0.020687863 | 0.008853477 | KDR/MMP2/MMP9/TGFB2/TGFBR1 | 5 |
| GO:0001953 | biological process | negative regulation of cell-matrix adhesion | 2/59 | 43/18866 | 0.007996137 | 0.020952077 | 0.008966549 | PIK3R1/SRC | 2 |
| GO:0002823 | biological process | negative regulation of adaptive immune response based on somatic recombination of immune receptors built from immunoglobulin superfamily domains | 2/59 | 43/18866 | 8.00E-03 | 2.10E-02 | 8.97E-03 | IL2/JAK3 | 2 |
| GO:0040019 | biological process | positive regulation of embryonic development | 2/59 | 43/18866 | 8.00E-03 | 2.10E-02 | 8.97E-03 | AR/IGF1 | 2 |
| GO:0045124 | biological process | regulation of bone resorption | 2/59 | 43/18866 | 8.00E-03 | 2.10E-02 | 8.97E-03 | EGFR/SRC | 2 |
| GO:0150077 | biological process | regulation of neuroinflammatory response | 2/59 | 43/18866 | 8.00E-03 | 2.10E-02 | 8.97E-03 | IGF1/MMP9 | 2 |
| GO:0019218 | biological process | regulation of steroid metabolic process | 3/59 | 131/18866 | 8.01E-03 | 2.10E-02 | 8.97E-03 | BMP2/KIT/VDR | 3 |
| GO:0045667 | biological process | regulation of osteoblast differentiation | 3/59 | 131/18866 | 8.01E-03 | 2.10E-02 | 8.97E-03 | BMP2/FGFR2/IGF1 | 3 |
| GO:0006282 | biological process | regulation of DNA repair | 3/59 | 132/18866 | 8.18E-03 | 2.13E-02 | 9.13E-03 | CHEK1/EGFR/PARP1 | 3 |
| GO:0010595 | biological process | positive regulation of endothelial cell migration | 3/59 | 132/18866 | 8.18E-03 | 2.13E-02 | 9.13E-03 | FGFR1/KDR/MET | 3 |
| GO:1902807 | biological process | negative regulation of cell cycle G1/S phase transition | 3/59 | 132/18866 | 8.18E-03 | 2.13E-02 | 9.13E-03 | AURKA/CDK2/MDM2 | 3 |
| GO:0000086 | biological process | G2/M transition of mitotic cell cycle | 4/59 | 254/18866 | 8.18E-03 | 2.13E-02 | 9.13E-03 | AURKA/CCNA2/CDK2/HSP90AA1 | 4 |
| GO:0021575 | biological process | hindbrain morphogenesis | 2/59 | 44/18866 | 8.36E-03 | 2.16E-02 | 9.26E-03 | MAP2K1/PTPN11 | 2 |
| GO:0035329 | biological process | hippo signaling | 2/59 | 44/18866 | 8.36E-03 | 2.16E-02 | 9.26E-03 | CASP3/MAPK14 | 2 |
| GO:0042088 | biological process | T-helper 1 type immune response | 2/59 | 44/18866 | 8.36E-03 | 2.16E-02 | 9.26E-03 | HRAS/JAK3 | 2 |
| GO:0044060 | biological process | regulation of endocrine process | 2/59 | 44/18866 | 8.36E-03 | 2.16E-02 | 9.26E-03 | FGFR1/PTPN11 | 2 |
| GO:0045684 | biological process | positive regulation of epidermis development | 2/59 | 44/18866 | 8.36E-03 | 2.16E-02 | 9.26E-03 | TGFB2/VDR | 2 |
| GO:0045687 | biological process | positive regulation of glial cell differentiation | 2/59 | 44/18866 | 8.36E-03 | 2.16E-02 | 9.26E-03 | BMP2/PPARG | 2 |
| GO:0097178 | biological process | ruffle assembly | 2/59 | 44/18866 | 8.36E-03 | 2.16E-02 | 9.26E-03 | HRAS/RAC1 | 2 |
| GO:1905521 | biological process | regulation of macrophage migration | 2/59 | 44/18866 | 8.36E-03 | 2.16E-02 | 9.26E-03 | MAPK1/MAPK14 | 2 |
| GO:0090257 | biological process | regulation of muscle system process | 4/59 | 256/18866 | 8.41E-03 | 2.17E-02 | 9.31E-03 | IGF1/KIT/PARP1/PIK3CG | 4 |
| GO:0030072 | biological process | peptide hormone secretion | 4/59 | 257/18866 | 8.52E-03 | 2.20E-02 | 9.41E-03 | EGFR/JAK2/PTPN11/RAC1 | 4 |
| GO:0007498 | biological process | mesoderm development | 3/59 | 134/18866 | 8.52E-03 | 2.20E-02 | 9.41E-03 | FGFR1/FGFR2/JAK2 | 3 |
| GO:0046683 | biological process | response to organophosphorus | 3/59 | 134/18866 | 8.52E-03 | 2.20E-02 | 9.41E-03 | PIK3CG/STAT1/TYMS | 3 |
| GO:0010951 | biological process | negative regulation of endopeptidase activity | 4/59 | 258/18866 | 8.63E-03 | 2.23E-02 | 9.53E-03 | MDM2/MMP9/SRC/XIAP | 4 |
| GO:0032368 | biological process | regulation of lipid transport | 3/59 | 135/18866 | 8.70E-03 | 2.24E-02 | 9.59E-03 | AKT2/PPARG/PTPN11 | 3 |
| GO:0006775 | biological process | fat-soluble vitamin metabolic process | 2/59 | 45/18866 | 8.73E-03 | 2.24E-02 | 9.59E-03 | FGFR1/VDR | 2 |
| GO:0034198 | biological process | cellular response to amino acid starvation | 2/59 | 45/18866 | 8.73E-03 | 2.24E-02 | 9.59E-03 | MAPK1/MAPK8 | 2 |
| GO:0035094 | biological process | response to nicotine | 2/59 | 45/18866 | 8.73E-03 | 2.24E-02 | 9.59E-03 | CASP3/MAPK1 | 2 |
| GO:0045911 | biological process | positive regulation of DNA recombination | 2/59 | 45/18866 | 8.73E-03 | 2.24E-02 | 9.59E-03 | IL2/PARP1 | 2 |
| GO:0051204 | biological process | protein insertion into mitochondrial membrane | 2/59 | 45/18866 | 8.73E-03 | 2.24E-02 | 9.59E-03 | HSP90AA1/MAPK8 | 2 |
| GO:0031023 | biological process | microtubule organizing center organization | 3/59 | 136/18866 | 8.88E-03 | 2.27E-02 | 9.74E-03 | AURKA/CDK2/CHEK1 | 3 |
| GO:0030183 | biological process | B cell differentiation | 3/59 | 137/18866 | 9.05E-03 | 2.31E-02 | 9.91E-03 | JAK3/KIT/PIK3R1 | 3 |
| GO:0032273 | biological process | positive regulation of protein polymerization | 3/59 | 137/18866 | 9.05E-03 | 2.31E-02 | 9.91E-03 | HSP90AA1/MET/RAC1 | 3 |
| GO:1903038 | biological process | negative regulation of leukocyte cell-cell adhesion | 3/59 | 137/18866 | 9.05E-03 | 2.31E-02 | 9.91E-03 | CASP3/IL2/JAK3 | 3 |
| GO:0032692 | biological process | negative regulation of interleukin-1 production | 2/59 | 46/18866 | 9.11E-03 | 2.32E-02 | 9.94E-03 | GSTP1/IGF1 | 2 |
| GO:0035987 | biological process | endodermal cell differentiation | 2/59 | 46/18866 | 9.11E-03 | 2.32E-02 | 9.94E-03 | MMP2/MMP9 | 2 |
| GO:0045581 | biological process | negative regulation of T cell differentiation | 2/59 | 46/18866 | 9.11E-03 | 2.32E-02 | 9.94E-03 | IL2/JAK3 | 2 |
| GO:0045637 | biological process | regulation of myeloid cell differentiation | 4/59 | 263/18866 | 9.22E-03 | 2.35E-02 | 1.01E-02 | MAPK14/PIK3R1/RARA/STAT1 | 4 |
| GO:0010559 | biological process | regulation of glycoprotein biosynthetic process | 2/59 | 47/18866 | 9.50E-03 | 2.40E-02 | 1.03E-02 | IGF1/JAK3 | 2 |
| GO:0010569 | biological process | regulation of double-strand break repair via homologous recombination | 2/59 | 47/18866 | 9.50E-03 | 2.40E-02 | 1.03E-02 | CHEK1/PARP1 | 2 |
| GO:0030225 | biological process | macrophage differentiation | 2/59 | 47/18866 | 9.50E-03 | 2.40E-02 | 1.03E-02 | MMP9/PARP1 | 2 |
| GO:0030574 | biological process | collagen catabolic process | 2/59 | 47/18866 | 9.50E-03 | 2.40E-02 | 1.03E-02 | MMP2/MMP9 | 2 |
| GO:0032369 | biological process | negative regulation of lipid transport | 2/59 | 47/18866 | 9.50E-03 | 2.40E-02 | 1.03E-02 | AKT2/PTPN11 | 2 |
| GO:0035722 | biological process | interleukin-12-mediated signaling pathway | 2/59 | 47/18866 | 9.50E-03 | 2.40E-02 | 1.03E-02 | JAK2/SOD2 | 2 |
| GO:0035850 | biological process | epithelial cell differentiation involved in kidney development | 2/59 | 47/18866 | 9.50E-03 | 2.40E-02 | 1.03E-02 | MMP9/STAT1 | 2 |
| GO:0043303 | biological process | mast cell degranulation | 2/59 | 47/18866 | 9.50E-03 | 2.40E-02 | 1.03E-02 | KIT/PIK3CG | 2 |
| GO:0048599 | biological process | oocyte development | 2/59 | 47/18866 | 9.50E-03 | 2.40E-02 | 1.03E-02 | AURKA/IGF1 | 2 |
| GO:0060986 | biological process | endocrine hormone secretion | 2/59 | 47/18866 | 9.50E-03 | 2.40E-02 | 1.03E-02 | FGFR1/PTPN11 | 2 |
| GO:0002279 | biological process | mast cell activation involved in immune response | 2/59 | 48/18866 | 9.89E-03 | 0.024865809 | 1.06E-02 | KIT/PIK3CG | 2 |
| GO:0002820 | biological process | negative regulation of adaptive immune response | 2/59 | 48/18866 | 9.89E-03 | 0.024865809 | 1.06E-02 | IL2/JAK3 | 2 |
| GO:0009409 | biological process | response to cold | 2/59 | 48/18866 | 9.89E-03 | 0.024865809 | 1.06E-02 | HSP90AA1/PPARG | 2 |
| GO:0042220 | biological process | response to cocaine | 2/59 | 48/18866 | 9.89E-03 | 0.024865809 | 1.06E-02 | CCNA2/MDM2 | 2 |
| GO:0048701 | biological process | embryonic cranial skeleton morphogenesis | 2/59 | 48/18866 | 9.89E-03 | 0.024865809 | 1.06E-02 | FGFR2/TGFBR1 | 2 |
| GO:1990928 | biological process | response to amino acid starvation | 2/59 | 48/18866 | 9.89E-03 | 0.024865809 | 1.06E-02 | MAPK1/MAPK8 | 2 |
| GO:0031348 | biological process | negative regulation of defense response | 4/59 | 269/18866 | 9.96E-03 | 0.025017076 | 1.07E-02 | GSTP1/IGF1/IL2/PPARG | 4 |
| GO:0002687 | biological process | positive regulation of leukocyte migration | 3/59 | 142/18866 | 9.98E-03 | 0.025032582 | 1.07E-02 | MAPK1/MAPK14/RAC1 | 3 |
| GO:0007006 | biological process | mitochondrial membrane organization | 3/59 | 142/18866 | 9.98E-03 | 0.025032582 | 1.07E-02 | BCL2L1/HSP90AA1/MAPK8 | 3 |
| GO:1901988 | biological process | negative regulation of cell cycle phase transition | 4/59 | 270/18866 | 1.01E-02 | 0.025272566 | 1.08E-02 | AURKA/CDK2/CHEK1/MDM2 | 4 |
| GO:0045598 | biological process | regulation of fat cell differentiation | 3/59 | 143/18866 | 1.02E-02 | 0.025471068 | 1.09E-02 | BMP2/MAPK14/PPARG | 3 |
| GO:0002448 | biological process | mast cell mediated immunity | 2/59 | 49/18866 | 1.03E-02 | 0.025556196 | 1.09E-02 | KIT/PIK3CG | 2 |
| GO:0006692 | biological process | prostanoid metabolic process | 2/59 | 49/18866 | 1.03E-02 | 0.025556196 | 0.010936905 | GSTM1/GSTP1 | 2 |
| GO:0006693 | biological process | prostaglandin metabolic process | 2/59 | 49/18866 | 1.03E-02 | 0.025556196 | 0.010936905 | GSTM1/GSTP1 | 2 |
| GO:0007595 | biological process | lactation | 2/59 | 49/18866 | 1.03E-02 | 0.025556196 | 0.010936905 | ERBB4/VDR | 2 |
| GO:0010171 | biological process | body morphogenesis | 2/59 | 49/18866 | 1.03E-02 | 0.025556196 | 0.010936905 | MMP2/PTPN11 | 2 |
| GO:0031279 | biological process | regulation of cyclase activity | 2/59 | 49/18866 | 1.03E-02 | 0.025556196 | 0.010936905 | MAPK14/MAPK8 | 2 |
| GO:0071349 | biological process | cellular response to interleukin-12 | 2/59 | 49/18866 | 1.03E-02 | 0.025556196 | 0.010936905 | JAK2/SOD2 | 2 |
| GO:0090151 | biological process | establishment of protein localization to mitochondrial membrane | 2/59 | 49/18866 | 1.03E-02 | 0.025556196 | 0.010936905 | HSP90AA1/MAPK8 | 2 |
| GO:1900087 | biological process | positive regulation of G1/S transition of mitotic cell cycle | 2/59 | 49/18866 | 1.03E-02 | 0.025556196 | 0.010936905 | EGFR/MDM2 | 2 |
| GO:2000107 | biological process | negative regulation of leukocyte apoptotic process | 2/59 | 49/18866 | 1.03E-02 | 0.025556196 | 0.010936905 | IL2/JAK3 | 2 |
| GO:0010466 | biological process | negative regulation of peptidase activity | 4/59 | 272/18866 | 1.03E-02 | 0.025661705 | 0.010982058 | MDM2/MMP9/SRC/XIAP | 4 |
| GO:0002700 | biological process | regulation of production of molecular mediator of immune response | 3/59 | 144/18866 | 0.010365382 | 0.025702777 | 0.010999635 | IL2/JAK3/TGFB2 | 3 |
| GO:0021695 | biological process | cerebellar cortex development | 2/59 | 50/18866 | 0.010696869 | 0.026205181 | 0.011214642 | MAP2K1/PTPN11 | 2 |
| GO:0042987 | biological process | amyloid precursor protein catabolic process | 2/59 | 50/18866 | 0.010696869 | 0.026205181 | 0.011214642 | CASP3/IGF1 | 2 |
| GO:0043616 | biological process | keratinocyte proliferation | 2/59 | 50/18866 | 0.010696869 | 0.026205181 | 0.011214642 | FGFR2/VDR | 2 |
| GO:0045646 | biological process | regulation of erythrocyte differentiation | 2/59 | 50/18866 | 0.010696869 | 0.026205181 | 0.011214642 | MAPK14/STAT1 | 2 |
| GO:0045981 | biological process | positive regulation of nucleotide metabolic process | 2/59 | 50/18866 | 0.010696869 | 0.026205181 | 0.011214642 | IGF1/INSR | 2 |
| GO:0046850 | biological process | regulation of bone remodeling | 2/59 | 50/18866 | 0.010696869 | 0.026205181 | 0.011214642 | EGFR/SRC | 2 |
| GO:0048546 | biological process | digestive tract morphogenesis | 2/59 | 50/18866 | 0.010696869 | 0.026205181 | 0.011214642 | EGFR/FGFR2 | 2 |
| GO:0070671 | biological process | response to interleukin-12 | 2/59 | 50/18866 | 0.010696869 | 0.026205181 | 0.011214642 | JAK2/SOD2 | 2 |
| GO:0101023 | biological process | vascular endothelial cell proliferation | 2/59 | 50/18866 | 0.010696869 | 0.026205181 | 0.011214642 | FGFR1/PPARG | 2 |
| GO:1900544 | biological process | positive regulation of purine nucleotide metabolic process | 2/59 | 50/18866 | 0.010696869 | 0.026205181 | 0.011214642 | IGF1/INSR | 2 |
| GO:1902743 | biological process | regulation of lamellipodium organization | 2/59 | 50/18866 | 0.010696869 | 0.026205181 | 0.011214642 | RAC1/SRC | 2 |
| GO:1903580 | biological process | positive regulation of ATP metabolic process | 2/59 | 50/18866 | 0.010696869 | 0.026205181 | 0.011214642 | IGF1/INSR | 2 |
| GO:1903727 | biological process | positive regulation of phospholipid metabolic process | 2/59 | 50/18866 | 0.010696869 | 0.026205181 | 0.011214642 | KIT/SRC | 2 |
| GO:1905562 | biological process | regulation of vascular endothelial cell proliferation | 2/59 | 50/18866 | 0.010696869 | 0.026205181 | 0.011214642 | FGFR1/PPARG | 2 |
| GO:2000725 | biological process | regulation of cardiac muscle cell differentiation | 2/59 | 50/18866 | 0.010696869 | 0.026205181 | 0.011214642 | BMP2/IGF1 | 2 |
| GO:0000280 | biological process | nuclear division | 5/59 | 428/18866 | 0.010772453 | 0.026369166 | 0.01128482 | AURKA/CHEK1/IGF1/INSR/XIAP | 5 |
| GO:0044409 | biological process | entry into host | 3/59 | 147/18866 | 0.010959213 | 0.02680481 | 0.011471256 | EGFR/MET/SRC | 3 |
| GO:0002762 | biological process | negative regulation of myeloid leukocyte differentiation | 2/59 | 51/18866 | 0.011111281 | 0.027025045 | 0.011565507 | PIK3R1/RARA | 2 |
| GO:0021879 | biological process | forebrain neuron differentiation | 2/59 | 51/18866 | 0.011111281 | 0.027025045 | 0.011565507 | ERBB4/FGFR2 | 2 |
| GO:0031113 | biological process | regulation of microtubule polymerization | 2/59 | 51/18866 | 0.011111281 | 0.027025045 | 0.011565507 | MET/RAC1 | 2 |
| GO:0043277 | biological process | apoptotic cell clearance | 2/59 | 51/18866 | 0.011111281 | 0.027025045 | 0.011565507 | RAC1/RARA | 2 |
| GO:0050435 | biological process | amyloid-beta metabolic process | 2/59 | 51/18866 | 0.011111281 | 0.027025045 | 0.011565507 | CASP3/IGF1 | 2 |
| GO:0071675 | biological process | regulation of mononuclear cell migration | 2/59 | 51/18866 | 0.011111281 | 0.027025045 | 0.011565507 | MAPK1/MAPK14 | 2 |
| GO:0071827 | biological process | plasma lipoprotein particle organization | 2/59 | 51/18866 | 0.011111281 | 0.027025045 | 0.011565507 | ALB/PLA2G2A | 2 |
| GO:0110053 | biological process | regulation of actin filament organization | 4/59 | 278/18866 | 0.011134415 | 0.027059733 | 0.011580352 | MET/PIK3R1/RAC1/TGFBR1 | 4 |
| GO:0032102 | biological process | negative regulation of response to external stimulus | 5/59 | 433/18866 | 0.011285991 | 0.027406269 | 0.011728654 | GSTP1/IGF1/IL2/PLAU/PPARG | 5 |
| GO:0014074 | biological process | response to purine-containing compound | 3/59 | 149/18866 | 0.011365858 | 0.027556332 | 0.011792874 | PIK3CG/PPARG/STAT1 | 3 |
| GO:1903364 | biological process | positive regulation of cellular protein catabolic process | 3/59 | 149/18866 | 0.011365858 | 0.027556332 | 0.011792874 | AURKA/HSP90AA1/MDM2 | 3 |
| GO:0034329 | biological process | cell junction assembly | 5/59 | 434/18866 | 0.011390678 | 0.027594575 | 0.01180924 | CTNNA1/ERBB4/KDR/RAC1/SRC | 5 |
| GO:0009994 | biological process | oocyte differentiation | 2/59 | 52/18866 | 0.011532701 | 0.027762223 | 0.011880986 | AURKA/IGF1 | 2 |
| GO:0032330 | biological process | regulation of chondrocyte differentiation | 2/59 | 52/18866 | 0.011532701 | 0.027762223 | 0.011880986 | PTPN11/TGFBR1 | 2 |
| GO:0038066 | biological process | p38MAPK cascade | 2/59 | 52/18866 | 0.011532701 | 0.027762223 | 0.011880986 | BMP2/MAPK14 | 2 |
| GO:0046580 | biological process | negative regulation of Ras protein signal transduction | 2/59 | 52/18866 | 0.011532701 | 0.027762223 | 0.011880986 | MET/TGFB2 | 2 |
| GO:0050999 | biological process | regulation of nitric-oxide synthase activity | 2/59 | 52/18866 | 0.011532701 | 0.027762223 | 0.011880986 | EGFR/HSP90AA1 | 2 |
| GO:0051496 | biological process | positive regulation of stress fiber assembly | 2/59 | 52/18866 | 0.011532701 | 0.027762223 | 0.011880986 | RAC1/TGFBR1 | 2 |
| GO:0090329 | biological process | regulation of DNA-dependent DNA replication | 2/59 | 52/18866 | 0.011532701 | 0.027762223 | 0.011880986 | CDK2/FGFR1 | 2 |
| GO:2000677 | biological process | regulation of transcription regulatory region DNA binding | 2/59 | 52/18866 | 0.011532701 | 0.027762223 | 0.011880986 | IGF1/PARP1 | 2 |
| GO:0035107 | biological process | appendage morphogenesis | 3/59 | 150/18866 | 0.01157242 | 0.027792031 | 0.011893743 | FGFR1/FGFR2/TGFB2 | 3 |
| GO:0035108 | biological process | limb morphogenesis | 3/59 | 150/18866 | 0.01157242 | 0.027792031 | 0.011893743 | FGFR1/FGFR2/TGFB2 | 3 |
| GO:0045580 | biological process | regulation of T cell differentiation | 3/59 | 150/18866 | 0.01157242 | 0.027792031 | 0.011893743 | IL2/JAK3/RARA | 3 |
| GO:0032210 | biological process | regulation of telomere maintenance via telomerase | 2/59 | 53/18866 | 0.011961082 | 0.028680266 | 0.012273867 | MAPK1/SRC | 2 |
| GO:0048168 | biological process | regulation of neuronal synaptic plasticity | 2/59 | 53/18866 | 0.011961082 | 0.028680266 | 0.012273867 | HRAS/KIT | 2 |
| GO:0007605 | biological process | sensory perception of sound | 3/59 | 152/18866 | 0.011992043 | 0.028731916 | 0.012295971 | CASP3/FGFR1/KIT | 3 |
| GO:0001678 | biological process | cellular glucose homeostasis | 3/59 | 153/18866 | 0.012205111 | 0.029219457 | 0.012504617 | IGF1R/PIK3R1/RAC1 | 3 |
| GO:0042391 | biological process | regulation of membrane potential | 5/59 | 443/18866 | 0.012362984 | 0.029574198 | 0.01265643 | AKT2/BCL2L1/KDR/PARP1/SRC | 5 |
| GO:0006749 | biological process | glutathione metabolic process | 2/59 | 54/18866 | 0.012396378 | 0.029584469 | 0.012660826 | GSTM1/GSTP1 | 2 |
| GO:0030857 | biological process | negative regulation of epithelial cell differentiation | 2/59 | 54/18866 | 0.012396378 | 0.029584469 | 0.012660826 | MMP9/STAT1 | 2 |
| GO:1903018 | biological process | regulation of glycoprotein metabolic process | 2/59 | 54/18866 | 0.012396378 | 0.029584469 | 0.012660826 | IGF1/JAK3 | 2 |
| GO:0051250 | biological process | negative regulation of lymphocyte activation | 3/59 | 154/18866 | 0.012420355 | 0.029595377 | 0.012665494 | CASP3/IL2/JAK3 | 3 |
| GO:0061351 | biological process | neural precursor cell proliferation | 3/59 | 154/18866 | 0.012420355 | 0.029595377 | 0.012665494 | CTNNA1/FGFR1/FGFR2 | 3 |
| GO:0030509 | biological process | BMP signaling pathway | 3/59 | 155/18866 | 0.012637779 | 0.03008995 | 0.012877148 | BMP2/TGFB2/XIAP | 3 |
| GO:1902105 | biological process | regulation of leukocyte differentiation | 4/59 | 290/18866 | 0.012838514 | 0.030378244 | 0.013000526 | IL2/JAK3/PIK3R1/RARA | 4 |
| GO:0001706 | biological process | endoderm formation | 2/59 | 55/18866 | 0.012838543 | 0.030378244 | 0.013000526 | MMP2/MMP9 | 2 |
| GO:0001954 | biological process | positive regulation of cell-matrix adhesion | 2/59 | 55/18866 | 0.012838543 | 0.030378244 | 0.013000526 | KDR/RAC1 | 2 |
| GO:0030199 | biological process | collagen fibril organization | 2/59 | 55/18866 | 0.012838543 | 0.030378244 | 0.013000526 | TGFB2/TGFBR1 | 2 |
| GO:0032655 | biological process | regulation of interleukin-12 production | 2/59 | 55/18866 | 0.012838543 | 0.030378244 | 0.013000526 | JAK3/MAPK14 | 2 |
| GO:0045620 | biological process | negative regulation of lymphocyte differentiation | 2/59 | 55/18866 | 0.012838543 | 0.030378244 | 0.013000526 | IL2/JAK3 | 2 |
| GO:0050819 | biological process | negative regulation of coagulation | 2/59 | 55/18866 | 0.012838543 | 0.030378244 | 0.013000526 | ANXA5/PLAU | 2 |
| GO:0071825 | biological process | protein-lipid complex subunit organization | 2/59 | 55/18866 | 0.012838543 | 0.030378244 | 0.013000526 | ALB/PLA2G2A | 2 |
| GO:0008360 | biological process | regulation of cell shape | 3/59 | 156/18866 | 0.012857386 | 0.030399245 | 0.013009513 | KDR/KIT/RAC1 | 3 |
| GO:1901990 | biological process | regulation of mitotic cell cycle phase transition | 5/59 | 448/18866 | 0.012926916 | 0.030539964 | 0.013069734 | AURKA/CDK2/EGFR/HSP90AA1/MDM2 | 5 |
| GO:0031529 | biological process | ruffle organization | 2/59 | 56/18866 | 0.013287532 | 0.03129496 | 0.013392839 | HRAS/RAC1 | 2 |
| GO:0060563 | biological process | neuroepithelial cell differentiation | 2/59 | 56/18866 | 0.013287532 | 0.03129496 | 0.013392839 | BMP2/FGFR1 | 2 |
| GO:0070228 | biological process | regulation of lymphocyte apoptotic process | 2/59 | 56/18866 | 0.013287532 | 0.03129496 | 0.013392839 | IL2/JAK3 | 2 |
| GO:0090183 | biological process | regulation of kidney development | 2/59 | 56/18866 | 0.013287532 | 0.03129496 | 0.013392839 | MMP9/STAT1 | 2 |
| GO:0044262 | biological process | cellular carbohydrate metabolic process | 4/59 | 294/18866 | 0.013441639 | 0.031633487 | 0.013537713 | AKT2/IGF1/INSR/SRC | 4 |
| GO:0032615 | biological process | interleukin-12 production | 2/59 | 57/18866 | 0.0137433 | 0.032174766 | 0.013769356 | JAK3/MAPK14 | 2 |
| GO:0042743 | biological process | hydrogen peroxide metabolic process | 2/59 | 57/18866 | 0.0137433 | 0.032174766 | 0.013769356 | EGFR/RAC1 | 2 |
| GO:0046854 | biological process | phosphatidylinositol phosphorylation | 2/59 | 57/18866 | 0.0137433 | 0.032174766 | 0.013769356 | PIK3CG/PIK3R1 | 2 |
| GO:0061005 | biological process | cell differentiation involved in kidney development | 2/59 | 57/18866 | 0.0137433 | 0.032174766 | 0.013769356 | MMP9/STAT1 | 2 |
| GO:0071398 | biological process | cellular response to fatty acid | 2/59 | 57/18866 | 0.0137433 | 0.032174766 | 0.013769356 | PPARG/SRC | 2 |
| GO:1905517 | biological process | macrophage migration | 2/59 | 57/18866 | 0.0137433 | 0.032174766 | 0.013769356 | MAPK1/MAPK14 | 2 |
| GO:0006644 | biological process | phospholipid metabolic process | 5/59 | 455/18866 | 0.013745482 | 0.032174766 | 0.013769356 | KIT/PIK3CG/PIK3R1/PLA2G2A/SRC | 5 |
| GO:0006874 | biological process | cellular calcium ion homeostasis | 5/59 | 456/18866 | 0.01386522 | 0.032430154 | 0.013878651 | ESR1/IL2/JAK2/PIK3CG/VDR | 5 |
| GO:0007566 | biological process | embryo implantation | 2/59 | 58/18866 | 0.0142058 | 0.033125144 | 0.014176076 | MMP2/MMP9 | 2 |
| GO:0010656 | biological process | negative regulation of muscle cell apoptotic process | 2/59 | 58/18866 | 0.0142058 | 0.033125144 | 0.014176076 | IGF1/JAK2 | 2 |
| GO:1900408 | biological process | negative regulation of cellular response to oxidative stress | 2/59 | 58/18866 | 0.0142058 | 0.033125144 | 0.014176076 | MET/SOD2 | 2 |
| GO:1903202 | biological process | negative regulation of oxidative stress-induced cell death | 2/59 | 58/18866 | 0.0142058 | 0.033125144 | 0.014176076 | MET/SOD2 | 2 |
| GO:0043467 | biological process | regulation of generation of precursor metabolites and energy | 3/59 | 162/18866 | 0.014221045 | 0.03313536 | 0.014180448 | AKT2/IGF1/INSR | 3 |
| GO:0051258 | biological process | protein polymerization | 4/59 | 300/18866 | 0.014379845 | 0.033479791 | 0.014327848 | HSP90AA1/JAK2/MET/RAC1 | 4 |
| GO:0009267 | biological process | cellular response to starvation | 3/59 | 163/18866 | 0.014456019 | 0.033554686 | 0.0143599 | ALB/MAPK1/MAPK8 | 3 |
| GO:0021915 | biological process | neural tube development | 3/59 | 163/18866 | 0.014456019 | 0.033554686 | 0.0143599 | CASP3/RARA/TGFB2 | 3 |
| GO:0031214 | biological process | biomineral tissue development | 3/59 | 163/18866 | 0.014456019 | 0.033554686 | 0.0143599 | BMP2/FGFR2/IGF1 | 3 |
| GO:0110148 | biological process | biomineralization | 3/59 | 163/18866 | 0.014456019 | 0.033554686 | 0.0143599 | BMP2/FGFR2/IGF1 | 3 |
| GO:0006636 | biological process | unsaturated fatty acid biosynthetic process | 2/59 | 59/18866 | 0.014674987 | 0.033918224 | 0.014515478 | GSTM1/GSTP1 | 2 |
| GO:0031663 | biological process | lipopolysaccharide-mediated signaling pathway | 2/59 | 59/18866 | 0.014674987 | 0.033918224 | 0.014515478 | MAPK1/MAPK14 | 2 |
| GO:0046605 | biological process | regulation of centrosome cycle | 2/59 | 59/18866 | 0.014674987 | 0.033918224 | 0.014515478 | AURKA/CHEK1 | 2 |
| GO:0051058 | biological process | negative regulation of small GTPase mediated signal transduction | 2/59 | 59/18866 | 0.014674987 | 0.033918224 | 0.014515478 | MET/TGFB2 | 2 |
| GO:0071385 | biological process | cellular response to glucocorticoid stimulus | 2/59 | 59/18866 | 1.47E-02 | 3.39E-02 | 1.45E-02 | EGFR/GSTP1 | 2 |
| GO:0001764 | biological process | neuron migration | 3/59 | 164/18866 | 1.47E-02 | 3.39E-02 | 1.45E-02 | ERBB4/FGFR1/RAC1 | 3 |
| GO:0043535 | biological process | regulation of blood vessel endothelial cell migration | 3/59 | 164/18866 | 1.47E-02 | 3.39E-02 | 1.45E-02 | FGFR1/KDR/PPARG | 3 |
| GO:0048562 | biological process | embryonic organ morphogenesis | 4/59 | 302/18866 | 1.47E-02 | 3.39E-02 | 1.45E-02 | FGFR1/FGFR2/MAPK1/TGFBR1 | 4 |
| GO:0016482 | biological process | cytosolic transport | 3/59 | 165/18866 | 1.49E-02 | 3.44E-02 | 1.47E-02 | MAP2K1/MAPK1/SRC | 3 |
| GO:0010823 | biological process | negative regulation of mitochondrion organization | 2/59 | 60/18866 | 1.52E-02 | 3.48E-02 | 1.49E-02 | BCL2L1/IGF1 | 2 |
| GO:0050994 | biological process | regulation of lipid catabolic process | 2/59 | 60/18866 | 1.52E-02 | 3.48E-02 | 1.49E-02 | AKT2/PIK3CG | 2 |
| GO:0061900 | biological process | glial cell activation | 2/59 | 60/18866 | 1.52E-02 | 3.48E-02 | 1.49E-02 | EGFR/JAK2 | 2 |
| GO:0097755 | biological process | positive regulation of blood vessel diameter | 2/59 | 60/18866 | 1.52E-02 | 3.48E-02 | 1.49E-02 | EGFR/SOD2 | 2 |
| GO:1902808 | biological process | positive regulation of cell cycle G1/S phase transition | 2/59 | 60/18866 | 1.52E-02 | 3.48E-02 | 1.49E-02 | EGFR/MDM2 | 2 |
| GO:1902883 | biological process | negative regulation of response to oxidative stress | 2/59 | 60/18866 | 1.52E-02 | 3.48E-02 | 1.49E-02 | MET/SOD2 | 2 |
| GO:0009408 | biological process | response to heat | 3/59 | 166/18866 | 1.52E-02 | 3.48E-02 | 1.49E-02 | HSP90AA1/IGF1/MAPK1 | 3 |
| GO:0010256 | biological process | endomembrane system organization | 5/59 | 468/18866 | 1.54E-02 | 3.51E-02 | 1.50E-02 | AKT2/AR/MAP2K1/MAPK1/TGFB2 | 5 |
| GO:0055074 | biological process | calcium ion homeostasis | 5/59 | 468/18866 | 1.54E-02 | 3.51E-02 | 1.50E-02 | ESR1/IL2/JAK2/PIK3CG/VDR | 5 |
| GO:0002440 | biological process | production of molecular mediator of immune response | 4/59 | 306/18866 | 1.54E-02 | 3.51E-02 | 1.50E-02 | IL2/JAK3/KIT/TGFB2 | 4 |
| GO:0002697 | biological process | regulation of immune effector process | 5/59 | 470/18866 | 1.56E-02 | 3.57E-02 | 1.53E-02 | IL2/JAK3/RARA/STAT1/TGFB2 | 5 |
| GO:0043954 | biological process | cellular component maintenance | 2/59 | 61/18866 | 1.56E-02 | 0.03566298 | 1.53E-02 | IGF1R/INSR | 2 |
| GO:0045576 | biological process | mast cell activation | 2/59 | 61/18866 | 1.56E-02 | 0.03566298 | 1.53E-02 | KIT/PIK3CG | 2 |
| GO:1903078 | biological process | positive regulation of protein localization to plasma membrane | 2/59 | 61/18866 | 1.56E-02 | 0.03566298 | 1.53E-02 | EGFR/PIK3R1 | 2 |
| GO:0071772 | biological process | response to BMP | 3/59 | 168/18866 | 1.57E-02 | 0.035679849 | 1.53E-02 | BMP2/TGFB2/XIAP | 3 |
| GO:0071773 | biological process | cellular response to BMP stimulus | 3/59 | 168/18866 | 1.57E-02 | 0.035679849 | 1.53E-02 | BMP2/TGFB2/XIAP | 3 |
| GO:0052126 | biological process | movement in host environment | 3/59 | 169/18866 | 1.59E-02 | 0.036218269 | 1.55E-02 | EGFR/MET/SRC | 3 |
| GO:0016236 | biological process | macroautophagy | 4/59 | 310/18866 | 1.60E-02 | 0.036468797 | 1.56E-02 | CASP3/KDR/MAPK8/SRC | 4 |
| GO:0032233 | biological process | positive regulation of actin filament bundle assembly | 2/59 | 62/18866 | 1.61E-02 | 0.036614149 | 1.57E-02 | RAC1/TGFBR1 | 2 |
| GO:0071384 | biological process | cellular response to corticosteroid stimulus | 2/59 | 62/18866 | 1.61E-02 | 0.036614149 | 1.57E-02 | EGFR/GSTP1 | 2 |
| GO:0032874 | biological process | positive regulation of stress-activated MAPK cascade | 3/59 | 170/18866 | 1.62E-02 | 0.036678882 | 1.57E-02 | BMP2/HRAS/TGFB2 | 3 |
| GO:0030856 | biological process | regulation of epithelial cell differentiation | 3/59 | 171/18866 | 1.64E-02 | 0.037196979 | 1.59E-02 | MMP9/STAT1/VDR | 3 |
| GO:1905952 | biological process | regulation of lipid localization | 3/59 | 171/18866 | 1.64E-02 | 0.037196979 | 1.59E-02 | AKT2/PPARG/PTPN11 | 3 |
| GO:0070304 | biological process | positive regulation of stress-activated protein kinase signaling cascade | 3/59 | 172/18866 | 1.67E-02 | 0.037746573 | 1.62E-02 | BMP2/HRAS/TGFB2 | 3 |
| GO:0021543 | biological process | pallium development | 3/59 | 173/18866 | 1.69E-02 | 0.038243648 | 1.64E-02 | CASP3/EGFR/RARA | 3 |
| GO:0033209 | biological process | tumor necrosis factor-mediated signaling pathway | 3/59 | 173/18866 | 1.69E-02 | 0.038243648 | 1.64E-02 | GSTP1/JAK2/STAT1 | 3 |
| GO:0050954 | biological process | sensory perception of mechanical stimulus | 3/59 | 173/18866 | 1.69E-02 | 0.038243648 | 1.64E-02 | CASP3/FGFR1/KIT | 3 |
| GO:0010469 | biological process | regulation of signaling receptor activity | 3/59 | 174/18866 | 1.72E-02 | 0.038772198 | 1.66E-02 | ESR2/PLAU/SRC | 3 |
| GO:0015718 | biological process | monocarboxylic acid transport | 3/59 | 174/18866 | 1.72E-02 | 0.038772198 | 1.66E-02 | AKT2/PLA2G2A/PPARG | 3 |
| GO:0010921 | biological process | regulation of phosphatase activity | 3/59 | 175/18866 | 1.74E-02 | 0.039333259 | 1.68E-02 | BMP2/JAK2/TGFB2 | 3 |
| GO:0032729 | biological process | positive regulation of interferon-gamma production | 2/59 | 65/18866 | 1.76E-02 | 0.039708703 | 1.70E-02 | HRAS/IL2 | 2 |
| GO:0016241 | biological process | regulation of macroautophagy | 3/59 | 176/18866 | 1.77E-02 | 0.039869073 | 1.71E-02 | CASP3/KDR/MAPK8 | 3 |
| GO:0045669 | biological process | positive regulation of osteoblast differentiation | 2/59 | 66/18866 | 1.81E-02 | 0.040807961 | 1.75E-02 | BMP2/IGF1 | 2 |
| GO:0007204 | biological process | positive regulation of cytosolic calcium ion concentration | 4/59 | 322/18866 | 1.82E-02 | 0.040844657 | 0.017479681 | ESR1/IL2/JAK2/PIK3CG | 4 |
| GO:0009895 | biological process | negative regulation of catabolic process | 4/59 | 323/18866 | 1.84E-02 | 0.041231936 | 0.017645419 | EGFR/MAPK14/MET/PIK3CG | 4 |
| GO:0016311 | biological process | dephosphorylation | 5/59 | 492/18866 | 1.87E-02 | 0.041733817 | 0.017860201 | BMP2/JAK2/PTPN11/SRC/TGFB2 | 5 |
| GO:0072503 | biological process | cellular divalent inorganic cation homeostasis | 5/59 | 492/18866 | 1.87E-02 | 0.041733817 | 0.017860201 | ESR1/IL2/JAK2/PIK3CG/VDR | 5 |
| GO:0034332 | biological process | adherens junction organization | 2/59 | 67/18866 | 1.87E-02 | 0.041733817 | 0.017860201 | CTNNA1/SRC | 2 |
| GO:0045123 | biological process | cellular extravasation | 2/59 | 67/18866 | 1.87E-02 | 0.041733817 | 0.017860201 | BST1/PIK3CG | 2 |
| GO:0050918 | biological process | positive chemotaxis | 2/59 | 67/18866 | 1.87E-02 | 0.041733817 | 0.017860201 | KDR/MET | 2 |
| GO:0071300 | biological process | cellular response to retinoic acid | 2/59 | 67/18866 | 1.87E-02 | 0.041733817 | 0.017860201 | FGFR2/PPARG | 2 |
| GO:0050851 | biological process | antigen receptor-mediated signaling pathway | 4/59 | 325/18866 | 1.87E-02 | 0.041858874 | 0.01791372 | HRAS/ITK/MAPK1/PIK3R1 | 4 |
| GO:0045619 | biological process | regulation of lymphocyte differentiation | 3/59 | 181/18866 | 1.91E-02 | 0.042537166 | 0.018203999 | IL2/JAK3/RARA | 3 |
| GO:0050796 | biological process | regulation of insulin secretion | 3/59 | 181/18866 | 1.91E-02 | 0.042537166 | 0.018203999 | JAK2/PTPN11/RAC1 | 3 |
| GO:0010812 | biological process | negative regulation of cell-substrate adhesion | 2/59 | 68/18866 | 1.92E-02 | 0.042756353 | 0.018297801 | PIK3R1/SRC | 2 |
| GO:1904377 | biological process | positive regulation of protein localization to cell periphery | 2/59 | 68/18866 | 1.92E-02 | 0.042756353 | 0.018297801 | EGFR/PIK3R1 | 2 |
| GO:0006898 | biological process | receptor-mediated endocytosis | 4/59 | 328/18866 | 1.93E-02 | 0.04297897 | 0.018393071 | ALB/HSP90AA1/INSR/RAC1 | 4 |
| GO:0071346 | biological process | cellular response to interferon-gamma | 3/59 | 182/18866 | 1.93E-02 | 0.04302985 | 0.018414845 | JAK2/PPARG/STAT1 | 3 |
| GO:1903034 | biological process | regulation of response to wounding | 3/59 | 183/18866 | 1.96E-02 | 0.043619807 | 0.01866732 | HRAS/MAP2K1/PLAU | 3 |
| GO:0022604 | biological process | regulation of cell morphogenesis | 5/59 | 499/18866 | 1.97E-02 | 0.043770472 | 0.018731798 | KDR/KIT/MAP2K1/RAC1/SRC | 5 |
| GO:0030032 | biological process | lamellipodium assembly | 2/59 | 69/18866 | 1.97E-02 | 0.043785317 | 0.018738151 | KIT/RAC1 | 2 |
| GO:0002695 | biological process | negative regulation of leukocyte activation | 3/59 | 184/18866 | 1.99E-02 | 0.044149573 | 0.018894036 | CASP3/IL2/JAK3 | 3 |
| GO:0043010 | biological process | camera-type eye development | 4/59 | 332/18866 | 0.020084389 | 0.04451845 | 0.019051899 | EGFR/RARA/TGFB2/TGFBR1 | 4 |
| GO:0046488 | biological process | phosphatidylinositol metabolic process | 3/59 | 185/18866 | 0.020187472 | 0.044714443 | 0.019135776 | PIK3CG/PIK3R1/PLA2G2A | 3 |
| GO:0009880 | biological process | embryonic pattern specification | 2/59 | 70/18866 | 0.020264764 | 0.04485307 | 0.019195102 | ERBB4/FGFR2 | 2 |
| GO:0002285 | biological process | lymphocyte activation involved in immune response | 3/59 | 187/18866 | 0.020762153 | 0.045854139 | 0.019623514 | IL2/JAK3/RARA | 3 |
| GO:0008217 | biological process | regulation of blood pressure | 3/59 | 187/18866 | 0.020762153 | 0.045854139 | 0.019623514 | AR/PPARG/SOD2 | 3 |
| GO:0050821 | biological process | protein stabilization | 3/59 | 187/18866 | 0.020762153 | 0.045854139 | 0.019623514 | HSP90AA1/IGF1/PIK3R1 | 3 |
| GO:0042982 | biological process | amyloid precursor protein metabolic process | 2/59 | 71/18866 | 0.020810765 | 0.045895034 | 0.019641016 | CASP3/IGF1 | 2 |
| GO:0045739 | biological process | positive regulation of DNA repair | 2/59 | 71/18866 | 0.020810765 | 0.045895034 | 0.019641016 | EGFR/PARP1 | 2 |
| GO:0010565 | biological process | regulation of cellular ketone metabolic process | 3/59 | 189/18866 | 0.021345811 | 0.047007021 | 0.020116896 | AKT2/BMP2/PPARG | 3 |
| GO:0043534 | biological process | blood vessel endothelial cell migration | 3/59 | 189/18866 | 0.021345811 | 0.047007021 | 0.020116896 | FGFR1/KDR/PPARG | 3 |
| GO:0046834 | biological process | lipid phosphorylation | 2/59 | 72/18866 | 0.021362886 | 0.04701068 | 0.020118462 | PIK3CG/PIK3R1 | 2 |
| GO:0003018 | biological process | vascular process in circulatory system | 3/59 | 190/18866 | 0.021641008 | 0.047554088 | 0.020351016 | EGFR/SOD2/SRC | 3 |
| GO:0030308 | biological process | negative regulation of cell growth | 3/59 | 190/18866 | 0.021641008 | 0.047554088 | 0.020351016 | ESR2/PPARG/TGFB2 | 3 |
| GO:0010611 | biological process | regulation of cardiac muscle hypertrophy | 2/59 | 73/18866 | 0.021921085 | 0.048065643 | 0.020569939 | IGF1/PARP1 | 2 |
| GO:0051155 | biological process | positive regulation of striated muscle cell differentiation | 2/59 | 73/18866 | 0.021921085 | 0.048065643 | 0.020569939 | IGF1/MAPK14 | 2 |
| GO:0070227 | biological process | lymphocyte apoptotic process | 2/59 | 73/18866 | 0.021921085 | 0.048065643 | 0.020569939 | IL2/JAK3 | 2 |
| GO:0016525 | biological process | negative regulation of angiogenesis | 3/59 | 192/18866 | 0.02223814 | 0.048690831 | 0.020837491 | PPARG/STAT1/TGFB2 | 3 |
| GO:0021953 | biological process | central nervous system neuron differentiation | 3/59 | 192/18866 | 0.02223814 | 0.048690831 | 0.020837491 | ERBB4/FGFR2/HSP90AA1 | 3 |
| GO:0010721 | biological process | negative regulation of cell development | 4/59 | 343/18866 | 0.022326529 | 0.048849292 | 0.020905305 | CTNNA1/ERBB4/IGF1/MDM2 | 4 |
| GO:0032024 | biological process | positive regulation of insulin secretion | 2/59 | 74/18866 | 0.022485321 | 0.049126238 | 0.021023826 | JAK2/RAC1 | 2 |
| GO:0032481 | biological process | positive regulation of type I interferon production | 2/59 | 74/18866 | 0.022485321 | 0.049126238 | 0.021023826 | PTPN11/STAT1 | 2 |
| GO:2000181 | biological process | negative regulation of blood vessel morphogenesis | 3/59 | 194/18866 | 0.022844258 | 0.049874721 | 0.021344143 | PPARG/STAT1/TGFB2 | 3 |
| GO:0004713 | molecular function | protein tyrosine kinase activity | 14/59 | 135/18352 | 6.84E-18 | 2.11E-15 | 1.19E-15 | EGFR/ERBB4/FGFR1/FGFR2/IGF1R/INSR/ITK/JAK2/JAK3/KDR/KIT/MAP2K1/MET/SRC | 14 |
| GO:0019199 | molecular function | transmembrane receptor protein kinase activity | 10/59 | 80/18352 | 7.32E-14 | 1.13E-11 | 6.36E-12 | EGFR/ERBB4/FGFR1/FGFR2/IGF1R/INSR/KDR/KIT/MET/TGFBR1 | 10 |
| GO:0004714 | molecular function | transmembrane receptor protein tyrosine kinase activity | 9/59 | 61/18352 | 2.95E-13 | 3.03E-11 | 1.71E-11 | EGFR/ERBB4/FGFR1/FGFR2/IGF1R/INSR/KDR/KIT/MET | 9 |
| GO:0043560 | molecular function | insulin receptor substrate binding | 5/59 | 10/18352 | 7.19E-11 | 5.53E-09 | 3.12E-09 | IGF1R/INSR/JAK2/PIK3R1/PTPN11 | 5 |
| GO:0004879 | molecular function | nuclear receptor activity | 7/59 | 52/18352 | 2.94E-10 | 1.51E-08 | 8.50E-09 | AR/ESR1/ESR2/PGR/PPARG/RARA/VDR | 7 |
| GO:0098531 | molecular function | ligand-activated transcription factor activity | 7/59 | 52/18352 | 2.94E-10 | 1.51E-08 | 8.50E-09 | AR/ESR1/ESR2/PGR/PPARG/RARA/VDR | 7 |
| GO:0005158 | molecular function | insulin receptor binding | 5/59 | 23/18352 | 9.30E-09 | 4.09E-07 | 2.31E-07 | IGF1/IGF1R/PIK3R1/PTPN11/SRC | 5 |
| GO:0051427 | molecular function | hormone receptor binding | 8/59 | 177/18352 | 9.32E-08 | 3.59E-06 | 2.02E-06 | ESR1/JAK2/PARP1/PPARG/PTPN11/SRC/STAT1/VDR | 8 |
| GO:0004674 | molecular function | protein serine/threonine kinase activity | 11/59 | 435/18352 | 1.18E-07 | 4.02E-06 | 2.27E-06 | AKT2/AURKA/CDK2/CHEK1/EGFR/MAP2K1/MAPK1/MAPK14/MAPK8/PIK3CG/TGFBR1 | 11 |
| GO:0019902 | molecular function | phosphatase binding | 8/59 | 194/18352 | 1.89E-07 | 5.81E-06 | 3.28E-06 | EGFR/JAK3/MAPK1/MAPK14/MET/PIK3R1/PPARG/STAT1 | 8 |
| GO:0035173 | molecular function | histone kinase activity | 4/59 | 17/18352 | 2.22E-07 | 6.22E-06 | 3.51E-06 | AURKA/CDK2/CHEK1/JAK2 | 4 |
| GO:0019838 | molecular function | growth factor binding | 7/59 | 136/18352 | 2.60E-07 | 6.67E-06 | 3.76E-06 | EGFR/FGFR1/FGFR2/IGF1R/INSR/KDR/TGFBR1 | 7 |
| GO:0051117 | molecular function | ATPase binding | 6/59 | 88/18352 | 3.76E-07 | 8.90E-06 | 5.02E-06 | AR/EGFR/ESR1/PGR/RAC1/SRC | 6 |
| GO:0019903 | molecular function | protein phosphatase binding | 7/59 | 149/18352 | 4.84E-07 | 1.06E-05 | 6.00E-06 | EGFR/JAK3/MAPK14/MET/PIK3R1/PPARG/STAT1 | 7 |
| GO:0043548 | molecular function | phosphatidylinositol 3-kinase binding | 4/59 | 30/18352 | 2.48E-06 | 5.09E-05 | 2.87E-05 | IGF1R/INSR/JAK2/PIK3R1 | 4 |
| GO:0033218 | molecular function | amide binding | 9/59 | 381/18352 | 3.28E-06 | 6.21E-05 | 3.50E-05 | GSTM1/GSTP1/IGF1R/INSR/NQO2/PIK3R1/PPARG/TGFB2/TYMS | 9 |
| GO:0033293 | molecular function | monocarboxylic acid binding | 5/59 | 72/18352 | 3.43E-06 | 6.21E-05 | 3.50E-05 | ALB/GSTP1/PPARG/RARA/VDR | 5 |
| GO:0035257 | molecular function | nuclear hormone receptor binding | 6/59 | 144/18352 | 6.73E-06 | 0.000115111 | 6.49E-05 | ESR1/PARP1/PPARG/SRC/STAT1/VDR | 6 |
| GO:0042562 | molecular function | hormone binding | 5/59 | 84/18352 | 7.34E-06 | 0.000119046 | 6.71E-05 | AR/EGFR/IGF1R/INSR/PIK3R1 | 5 |
| GO:0042169 | molecular function | SH2 domain binding | 4/59 | 41/18352 | 8.93E-06 | 0.000137471 | 7.75E-05 | FGFR1/JAK2/KIT/SRC | 4 |
| GO:0030331 | molecular function | estrogen receptor binding | 4/59 | 42/18352 | 9.84E-06 | 0.00014436 | 8.14E-05 | ESR1/PARP1/PPARG/SRC | 4 |
| GO:0004712 | molecular function | protein serine/threonine/tyrosine kinase activity | 4/59 | 45/18352 | 1.30E-05 | 0.000182116 | 0.000102697 | AURKA/MAP2K1/MAPK1/MAPK14 | 4 |
| GO:0004715 | molecular function | non-membrane spanning protein tyrosine kinase activity | 4/59 | 46/18352 | 1.42E-05 | 0.000190331 | 0.00010733 | ITK/JAK2/JAK3/SRC | 4 |
| GO:0005159 | molecular function | insulin-like growth factor receptor binding | 3/59 | 16/18352 | 1.72E-05 | 0.000220178 | 0.000124161 | IGF1/INSR/PIK3R1 | 3 |
| GO:0016922 | molecular function | nuclear receptor binding | 5/59 | 101/18352 | 1.81E-05 | 0.000222662 | 0.000125561 | ESR1/PARP1/PPARG/SRC/VDR | 5 |
| GO:0008144 | molecular function | drug binding | 5/59 | 104/18352 | 2.08E-05 | 0.000246751 | 0.000139145 | ALB/GSTP1/PPARG/RARA/TYMS | 5 |
| GO:0061629 | molecular function | RNA polymerase II-specific DNA-binding transcription factor binding | 7/59 | 267/18352 | 2.28E-05 | 0.000251258 | 0.000141687 | ESR1/MAPK14/PARP1/PPARG/SRC/STAT1/VDR | 7 |
| GO:0005496 | molecular function | steroid binding | 5/59 | 106/18352 | 2.28E-05 | 0.000251258 | 0.000141687 | AR/ESR1/ESR2/PGR/VDR | 5 |
| GO:0004708 | molecular function | MAP kinase kinase activity | 3/59 | 18/18352 | 2.49E-05 | 0.000257611 | 0.00014527 | MAP2K1/MAPK1/MAPK14 | 3 |
| GO:0005126 | molecular function | cytokine receptor binding | 7/59 | 271/18352 | 2.51E-05 | 0.000257611 | 0.00014527 | CASP3/IL2/JAK2/PIK3R1/STAT1/TGFB2/TGFBR1 | 7 |
| GO:0042826 | molecular function | histone deacetylase binding | 5/59 | 115/18352 | 3.38E-05 | 0.000333102 | 0.00018784 | HSP90AA1/MAPK8/PARP1/RAC1/RARA | 5 |
| GO:0004707 | molecular function | MAP kinase activity | 3/59 | 20/18352 | 3.46E-05 | 0.000333102 | 0.00018784 | MAPK1/MAPK14/MAPK8 | 3 |
| GO:0097110 | molecular function | scaffold protein binding | 4/59 | 60/18352 | 4.11E-05 | 0.000383337 | 0.000216167 | HSP90AA1/MAP2K1/MDM2/SRC | 4 |
| GO:0042277 | molecular function | peptide binding | 7/59 | 308/18352 | 5.66E-05 | 0.000512643 | 0.000289084 | GSTM1/GSTP1/IGF1R/INSR/PIK3R1/PPARG/TGFB2 | 7 |
| GO:0031406 | molecular function | carboxylic acid binding | 6/59 | 212/18352 | 5.99E-05 | 0.000527057 | 0.000297212 | ALB/GSTP1/PPARG/RARA/TYMS/VDR | 6 |
| GO:0019207 | molecular function | kinase regulator activity | 6/59 | 216/18352 | 6.64E-05 | 0.000568341 | 0.000320493 | CASP3/CCNA2/GSTP1/IL2/MAP2K1/PIK3R1 | 6 |
| GO:0003707 | molecular function | steroid hormone receptor activity | 3/59 | 26/18352 | 7.79E-05 | 0.000648091 | 0.000365465 | ESR1/ESR2/PGR | 3 |
| GO:0043177 | molecular function | organic acid binding | 6/59 | 224/18352 | 8.12E-05 | 0.000658248 | 0.000371192 | ALB/GSTP1/PPARG/RARA/TYMS/VDR | 6 |
| GO:0001223 | molecular function | transcription coactivator binding | 3/59 | 29/18352 | 0.000108669 | 0.000836748 | 0.000471851 | AR/ESR1/PGR | 3 |
| GO:0033612 | molecular function | receptor serine/threonine kinase binding | 3/59 | 29/18352 | 0.000108669 | 0.000836748 | 0.000471851 | BMP2/MDM2/SRC | 3 |
| GO:0140297 | molecular function | DNA-binding transcription factor binding | 7/59 | 347/18352 | 0.000119244 | 0.000895787 | 0.000505143 | ESR1/MAPK14/PARP1/PPARG/SRC/STAT1/VDR | 7 |
| GO:0035258 | molecular function | steroid hormone receptor binding | 4/59 | 81/18352 | 0.000133266 | 0.000977285 | 0.0005511 | ESR1/PARP1/PPARG/SRC | 4 |
| GO:0051219 | molecular function | phosphoprotein binding | 4/59 | 85/18352 | 0.000160643 | 0.00115065 | 0.000648863 | MAPK1/PIK3R1/PTPN11/SRC | 4 |
| GO:0005504 | molecular function | fatty acid binding | 3/59 | 39/18352 | 0.000265654 | 0.001859576 | 0.001048633 | ALB/GSTP1/PPARG | 3 |
| GO:0001784 | molecular function | phosphotyrosine residue binding | 3/59 | 42/18352 | 0.000331425 | 0.002268422 | 0.001279186 | MAPK1/PIK3R1/PTPN11 | 3 |
| GO:0008022 | molecular function | protein C-terminus binding | 5/59 | 189/18352 | 0.000350533 | 0.002347046 | 0.001323522 | HRAS/JAK2/MAP2K1/PPARG/SRC | 5 |
| GO:0044389 | molecular function | ubiquitin-like protein ligase binding | 6/59 | 316/18352 | 0.000520007 | 0.003407705 | 0.001921638 | AURKA/EGFR/HSP90AA1/MDM2/SRC/STAT1 | 6 |
| GO:0001221 | molecular function | transcription cofactor binding | 3/59 | 51/18352 | 0.000589 | 0.003702283 | 0.002087753 | AR/ESR1/PGR | 3 |
| GO:0017046 | molecular function | peptide hormone binding | 3/59 | 51/18352 | 0.000589 | 0.003702283 | 0.002087753 | IGF1R/INSR/PIK3R1 | 3 |
| GO:0043295 | molecular function | glutathione binding | 2/59 | 12/18352 | 0.000656881 | 0.003983171 | 0.002246149 | GSTM1/GSTP1 | 2 |
| GO:0045309 | molecular function | protein phosphorylated amino acid binding | 3/59 | 53/18352 | 0.000659551 | 0.003983171 | 0.002246149 | MAPK1/PIK3R1/PTPN11 | 3 |
| GO:0045296 | molecular function | cadherin binding | 6/59 | 332/18352 | 0.000673821 | 0.003991094 | 0.002250617 | CTNNA1/EGFR/KDR/PTPN11/SRC/STAT1 | 6 |
| GO:0031994 | molecular function | insulin-like growth factor I binding | 2/59 | 13/18352 | 0.00077471 | 0.004338376 | 0.002446453 | IGF1R/INSR | 2 |
| GO:0070700 | molecular function | BMP receptor binding | 2/59 | 13/18352 | 0.00077471 | 0.004338376 | 0.002446453 | BMP2/SRC | 2 |
| GO:1900750 | molecular function | oligopeptide binding | 2/59 | 13/18352 | 0.00077471 | 0.004338376 | 0.002446453 | GSTM1/GSTP1 | 2 |
| GO:0070851 | molecular function | growth factor receptor binding | 4/59 | 141/18352 | 0.001095197 | 0.006023581 | 0.003396756 | ERBB4/IL2/JAK2/SRC | 4 |
| GO:0005178 | molecular function | integrin binding | 4/59 | 144/18352 | 0.001184008 | 0.00629006 | 0.003547026 | EGFR/IGF1/KDR/SRC | 4 |
| GO:0000900 | molecular function | translation repressor activity, mRNA regulatory element binding | 2/59 | 16/18352 | 0.001184492 | 0.00629006 | 0.003547026 | RARA/TYMS | 2 |
| GO:0035591 | molecular function | signaling adaptor activity | 3/59 | 68/18352 | 0.001363623 | 0.007118572 | 0.004014232 | MAP2K1/PIK3R1/PTPN11 | 3 |
| GO:0016500 | molecular function | protein-hormone receptor activity | 2/59 | 18/18352 | 0.001503998 | 0.007593959 | 0.004282308 | IGF1R/INSR | 2 |
| GO:0046965 | molecular function | retinoid X receptor binding | 2/59 | 18/18352 | 0.001503998 | 0.007593959 | 0.004282308 | PPARG/VDR | 2 |
| GO:0001091 | molecular function | RNA polymerase II general transcription initiation factor binding | 2/59 | 19/18352 | 0.001677471 | 0.008333242 | 0.004699196 | AR/ESR1 | 2 |
| GO:0008083 | molecular function | growth factor activity | 4/59 | 162/18352 | 0.001825599 | 0.008925152 | 0.00503298 | BMP2/IGF1/IL2/TGFB2 | 4 |
| GO:0051428 | molecular function | peptide hormone receptor binding | 2/59 | 21/18352 | 0.002051561 | 0.009873139 | 0.005567559 | JAK2/PTPN11 | 2 |
| GO:0046332 | molecular function | SMAD binding | 3/59 | 79/18352 | 0.002098545 | 0.009943876 | 0.005607449 | BMP2/PARP1/TGFBR1 | 3 |
| GO:0017134 | molecular function | fibroblast growth factor binding | 2/59 | 23/18352 | 0.002461461 | 0.011486816 | 0.006477528 | FGFR1/FGFR2 | 2 |
| GO:0008013 | molecular function | beta-catenin binding | 3/59 | 85/18352 | 0.002585617 | 0.011624591 | 0.00655522 | AR/CTNNA1/ESR1 | 3 |
| GO:0031625 | molecular function | ubiquitin protein ligase binding | 5/59 | 297/18352 | 0.002632054 | 0.011624591 | 0.00655522 | AURKA/EGFR/HSP90AA1/MDM2/SRC | 5 |
| GO:0016209 | molecular function | antioxidant activity | 3/59 | 86/18352 | 0.002672998 | 0.011624591 | 0.00655522 | ALB/GSTP1/SOD2 | 3 |
| GO:0005160 | molecular function | transforming growth factor beta receptor binding | 2/59 | 24/18352 | 0.002679695 | 0.011624591 | 0.00655522 | TGFB2/TGFBR1 | 2 |
| GO:0070696 | molecular function | transmembrane receptor protein serine/threonine kinase binding | 2/59 | 24/18352 | 0.002679695 | 0.011624591 | 0.00655522 | BMP2/SRC | 2 |
| GO:0004364 | molecular function | glutathione transferase activity | 2/59 | 25/18352 | 0.002906709 | 0.012434255 | 0.007011798 | GSTM1/GSTP1 | 2 |
| GO:0072341 | molecular function | modified amino acid binding | 3/59 | 90/18352 | 0.003040707 | 0.012829286 | 0.00723456 | GSTM1/GSTP1/TYMS | 3 |
| GO:0030371 | molecular function | translation repressor activity | 2/59 | 26/18352 | 0.003142447 | 0.013079373 | 0.007375587 | RARA/TYMS | 2 |
| GO:0042974 | molecular function | retinoic acid receptor binding | 2/59 | 27/18352 | 0.003386852 | 0.013547407 | 0.007639515 | PPARG/VDR | 2 |
| GO:0046875 | molecular function | ephrin receptor binding | 2/59 | 27/18352 | 0.003386852 | 0.013547407 | 0.007639515 | PIK3CG/SRC | 2 |
| GO:0051393 | molecular function | alpha-actinin binding | 2/59 | 27/18352 | 0.003386852 | 0.013547407 | 0.007639515 | PPARG/RARA | 2 |
| GO:1990782 | molecular function | protein tyrosine kinase binding | 3/59 | 98/18352 | 0.003865887 | 0.015210667 | 0.008577444 | HSP90AA1/PIK3R1/PTPN11 | 3 |
| GO:0005520 | molecular function | insulin-like growth factor binding | 2/59 | 29/18352 | 0.003901437 | 0.015210667 | 0.008577444 | IGF1R/INSR | 2 |
| GO:0047485 | molecular function | protein N-terminus binding | 3/59 | 107/18352 | 0.004943151 | 0.019031132 | 0.010731841 | MAP2K1/MDM2/PARP1 | 3 |
| GO:0042805 | molecular function | actinin binding | 2/59 | 36/18352 | 0.005967315 | 0.022413816 | 0.01263937 | PPARG/RARA | 2 |
| GO:0097718 | molecular function | disordered domain specific binding | 2/59 | 36/18352 | 0.005967315 | 0.022413816 | 0.01263937 | HSP90AA1/MDM2 | 2 |
| GO:0030159 | molecular function | signaling receptor complex adaptor activity | 2/59 | 41/18352 | 0.007687527 | 0.02852721 | 0.016086773 | PIK3R1/PTPN11 | 2 |
| GO:0140296 | molecular function | general transcription initiation factor binding | 2/59 | 44/18352 | 0.008814284 | 0.032319043 | 0.018225024 | AR/ESR1 | 2 |
| GO:1901681 | molecular function | sulfur compound binding | 4/59 | 262/18352 | 0.009996414 | 0.0362223 | 0.020426109 | FGFR1/FGFR2/GSTM1/GSTP1 | 4 |
| GO:0032813 | molecular function | tumor necrosis factor receptor superfamily binding | 2/59 | 48/18352 | 0.010424102 | 0.03733283 | 0.021052348 | CASP3/STAT1 | 2 |
| GO:0019842 | molecular function | vitamin binding | 3/59 | 144/18352 | 0.011163759 | 0.039459513 | 0.022251605 | ALB/TYMS/VDR | 3 |
| GO:0016538 | molecular function | cyclin-dependent protein serine/threonine kinase regulator activity | 2/59 | 50/18352 | 0.011274147 | 0.039459513 | 0.022251605 | CASP3/CCNA2 | 2 |
| GO:0016765 | molecular function | transferase activity, transferring alkyl or aryl (other than methyl) groups | 2/59 | 56/18352 | 0.013999976 | 0.048449356 | 0.027321065 | GSTM1/GSTP1 | 2 |
| GO:0001664 | molecular function | G protein-coupled receptor binding | 4/59 | 293/18352 | 0.014568664 | 0.049857206 | 0.028114966 | IL2/JAK2/PTPN11/STAT1 | 4 |
| GO:0045121 | cellular components | membrane raft | 9/59 | 329/19559 | 5.80E-07 | 5.03E-05 | 3.63E-05 | CASP3/CTNNA1/EGFR/INSR/JAK2/KDR/MAPK1/SRC/TGFBR1 | 9 |
| GO:0098857 | cellular components | membrane microdomain | 9/59 | 330/19559 | 5.95E-07 | 5.03E-05 | 3.63E-05 | CASP3/CTNNA1/EGFR/INSR/JAK2/KDR/MAPK1/SRC/TGFBR1 | 9 |
| GO:0098589 | cellular components | membrane region | 9/59 | 343/19559 | 8.20E-07 | 5.03E-05 | 3.63E-05 | CASP3/CTNNA1/EGFR/INSR/JAK2/KDR/MAPK1/SRC/TGFBR1 | 9 |
| GO:0005925 | cellular components | focal adhesion | 9/59 | 415/19559 | 3.93E-06 | 1.47E-04 | 1.06E-04 | ANXA5/CTNNA1/EGFR/JAK2/MAP2K1/MAPK1/PLAU/RAC1/SRC | 9 |
| GO:0030055 | cellular components | cell-substrate junction | 9/59 | 423/19559 | 4.59E-06 | 1.47E-04 | 1.06E-04 | ANXA5/CTNNA1/EGFR/JAK2/MAP2K1/MAPK1/PLAU/RAC1/SRC | 9 |
| GO:0005901 | cellular components | caveola | 5/59 | 82/19559 | 4.80E-06 | 1.47E-04 | 1.06E-04 | CTNNA1/INSR/JAK2/MAPK1/SRC | 5 |
| GO:0031983 | cellular components | vesicle lumen | 8/59 | 328/19559 | 6.05E-06 | 1.59E-04 | 1.15E-04 | ALB/EGFR/GSTP1/HSP90AA1/IGF1/MAPK1/MAPK14/TGFB2 | 8 |
| GO:0061695 | cellular components | transferase complex, transferring phosphorus-containing groups | 7/59 | 253/19559 | 1.07E-05 | 2.47E-04 | 1.78E-04 | CCNA2/CDK2/IGF1R/INSR/PIK3CG/PIK3R1/TGFBR1 | 7 |
| GO:1902911 | cellular components | protein kinase complex | 5/59 | 104/19559 | 1.54E-05 | 3.14E-04 | 2.27E-04 | CCNA2/CDK2/IGF1R/INSR/TGFBR1 | 5 |
| GO:0044853 | cellular components | plasma membrane raft | 5/59 | 113/19559 | 2.30E-05 | 4.23E-04 | 3.05E-04 | CTNNA1/INSR/JAK2/MAPK1/SRC | 5 |
| GO:0101002 | cellular components | ficolin-1-rich granule | 5/59 | 124/19559 | 3.60E-05 | 5.51E-04 | 3.97E-04 | GSTP1/HSP90AA1/MAPK1/MAPK14/MMP9 | 5 |
| GO:1904813 | cellular components | ficolin-1-rich granule lumen | 5/59 | 124/19559 | 3.60E-05 | 5.51E-04 | 3.97E-04 | GSTP1/HSP90AA1/MAPK1/MAPK14/MMP9 | 5 |
| GO:0034774 | cellular components | secretory granule lumen | 7/59 | 322/19559 | 5.03E-05 | 7.12E-04 | 5.13E-04 | ALB/GSTP1/HSP90AA1/IGF1/MAPK1/MAPK14/TGFB2 | 7 |
| GO:0060205 | cellular components | cytoplasmic vesicle lumen | 7/59 | 326/19559 | 5.44E-05 | 7.14E-04 | 5.15E-04 | ALB/GSTP1/HSP90AA1/IGF1/MAPK1/MAPK14/TGFB2 | 7 |
| GO:0009925 | cellular components | basal plasma membrane | 3/59 | 51/19559 | 4.90E-04 | 6.01E-03 | 4.33E-03 | EGFR/ERBB4/MET | 3 |
| GO:0098978 | cellular components | glutamatergic synapse | 6/59 | 361/19559 | 7.50E-04 | 0.008619275 | 0.006212979 | ERBB4/HRAS/JAK2/MAPK14/RAC1/SRC | 6 |
| GO:0045120 | cellular components | pronucleus | 2/59 | 16/19559 | 1.04E-03 | 0.011131307 | 0.008023711 | AURKA/CCNA2 | 2 |
| GO:0031093 | cellular components | platelet alpha granule lumen | 3/59 | 67/19559 | 1.09E-03 | 0.011131307 | 0.008023711 | ALB/IGF1/TGFB2 | 3 |
| GO:0045178 | cellular components | basal part of cell | 3/59 | 69/19559 | 1.19E-03 | 0.011484473 | 0.008278281 | EGFR/ERBB4/MET | 3 |
| GO:0005770 | cellular components | late endosome | 5/59 | 278/19559 | 1.50E-03 | 0.013775937 | 0.009930023 | EGFR/INSR/MAP2K1/MAPK1/SRC | 5 |
| GO:0031256 | cellular components | leading edge membrane | 4/59 | 175/19559 | 1.92E-03 | 0.016624771 | 0.011983531 | AKT2/INSR/RAC1/SRC | 4 |
| GO:0042629 | cellular components | mast cell granule | 2/59 | 22/19559 | 0.001987744 | 0.016624771 | 0.011983531 | KIT/PIK3CG | 2 |
| GO:0019898 | cellular components | extrinsic component of membrane | 5/59 | 306/19559 | 2.28E-03 | 1.82E-02 | 1.31E-02 | BST1/CTNNA1/PIK3CG/PIK3R1/SRC | 5 |
| GO:1902554 | cellular components | serine/threonine protein kinase complex | 3/59 | 89/19559 | 2.46E-03 | 1.89E-02 | 1.36E-02 | CCNA2/CDK2/TGFBR1 | 3 |
| GO:0031091 | cellular components | platelet alpha granule | 3/59 | 91/19559 | 2.62E-03 | 1.93E-02 | 1.39E-02 | ALB/IGF1/TGFB2 | 3 |
| GO:0032587 | cellular components | ruffle membrane | 3/59 | 95/19559 | 2.96E-03 | 2.10E-02 | 1.51E-02 | AKT2/RAC1/SRC | 3 |
| GO:0005942 | cellular components | phosphatidylinositol 3-kinase complex | 2/59 | 29/19559 | 3.45E-03 | 2.35E-02 | 1.69E-02 | PIK3CG/PIK3R1 | 2 |
| GO:0005769 | cellular components | early endosome | 5/59 | 377/19559 | 0.005531382 | 0.036349081 | 0.026201283 | AKT2/EGFR/KDR/MAP2K1/MAPK1 | 5 |
| GO:0000307 | cellular components | cyclin-dependent protein kinase holoenzyme complex | 2/59 | 43/19559 | 0.007461197 | 0.047340008 | 0.034123805 | CCNA2/CDK2 | 2 |

**Table S5 KEGG pathways**

| **Table S5** KEGG pathways | |  |  |  |  |  |
| --- | --- | --- | --- | --- | --- | --- |
| GeneRatio | BgRatio | pvalue | p.adjust | qvalue | geneID | Count |
| 23/57 | 205/8111 | 1.32E-22 | 2.71E-20 | 8.49E-21 | AKT2/CASP3/EGFR/ERBB4/ESR1/  FGFR1/HRAS/IGF1/IGF1R/KDR/  MAP2K1/MAPK1/MAPK14/MDM2  /MET/MMP2/MMP9/PIK3R1/PLAU/  PTPN11/RAC1/SRC/TGFB2 | 23 |
| 17/57 | 97/8111 | 4.61E-20 | 4.72E-18 | 1.48E-18 | AKT2/AR/CDK2/EGFR/FGFR1/  FGFR2/GSTP1/HRAS/HSP90AA1  /IGF1/IGF1R/MAP2K1/MAPK1/  MDM2/MMP9/PIK3R1/PLAU | 17 |
| 24/57 | 354/8111 | 2.14E-18 | 1.15E-16 | 3.60E-17 | AKT2/BCL2L1/CDK2/EGFR/  ERBB4/FGFR1/FGFR2/HRAS/  HSP90AA1/IGF1/IGF1R/IL2/  INSR/JAK2/JAK3/KDR/KIT/  MAP2K1/MAPK1/MDM2/MET/  PIK3CG/PIK3R1/RAC1 | 24 |
| 16/57 | 98/8111 | 2.24E-18 | 1.15E-16 | 3.60E-17 | AKT2/EGFR/ESR1/ESR2/HRAS/  IGF1/IGF1R/MAP2K1/MAPK1/  MAPK14/MAPK8/MDM2/MMP2/  MMP9/PIK3R1/SRC | 16 |
| 14/57 | 79/8111 | 1.14E-16 | 4.69E-15 | 1.47E-15 | AKT2/BCL2L1/EGFR/FGFR2/HRAS  /IGF1/IGF1R/JAK2/KDR/MAP2K1/  MAPK1/MET/PIK3R1/SRC | 14 |
| 16/57 | 131/8111 | 2.79E-16 | 9.54E-15 | 2.99E-15 | AKT2/CDK2/EGFR/HRAS/IGF1/  IGF1R/INSR/MAP2K1/MAPK1/  MAPK14/MAPK8/MDM2/PIK3R1/  SOD2/TGFB2/TGFBR1 | 16 |
| 17/57 | 162/8111 | 3.85E-16 | 1.13E-14 | 3.53E-15 | AKT2/CASP3/CCNA2/CDK2/HRAS/  JAK2/JAK3/MAP2K1/MAPK1/MAPK14/  MAPK8/MMP9/PIK3R1/SRC/STAT1/  TGFB2/TGFBR1 | 17 |
| 19/57 | 232/8111 | 5.47E-16 | 1.40E-14 | 4.39E-15 | AKT2/BCL2L1/EGFR/FGFR1/FGFR2/  HRAS/IGF1/IGF1R/INSR/KDR/KIT/  MAP2K1/MAPK1/MAPK8/MET/  PIK3R1/PLA2G2A/PTPN11/RAC1 | 19 |
| 20/57 | 294/8111 | 2.91E-15 | 6.63E-14 | 2.08E-14 | AKT2/CASP3/EGFR/ERBB4/FGFR1/  FGFR2/HRAS/IGF1/IGF1R/INSR/KDR/  KIT/MAP2K1/MAPK1/MAPK14/MAPK8/  MET/RAC1/TGFB2/TGFBR1 | 20 |
| 17/57 | 210/8111 | 3.10E-14 | 6.19E-13 | 1.94E-13 | AKT2/EGFR/FGFR1/FGFR2/HRAS/  IGF1/IGF1R/INSR/KDR/KIT/MAP2K1/  MAPK1/MAPK14/MET/PIK3R1/RAC1/SRC | 17 |
| 12/57 | 70/8111 | 3.32E-14 | 6.19E-13 | 1.94E-13 | AKT2/ESR1/ESR2/HRAS/JAK2/  MAP2K1/MAPK1/MAPK14/  MAPK8/PIK3R1/SRC/STAT1 | 12 |
| 13/57 | 100/8111 | 1.07E-13 | 1.69E-12 | 5.29E-13 | AKT2/AURKA/CCNA2/CDK2/  HSP90AA1/IGF1/IGF1R/MAP2K1/  MAPK1/MAPK14/MAPK8/PGR/PIK3R1 | 13 |
| 13/57 | 100/8111 | 1.07E-13 | 1.69E-12 | 5.29E-13 | AKT2/CASP3/HRAS/JAK2/MAPK1/  MAPK14/MAPK8/MMP2/PIK3R1/  RAC1/STAT1/TGFB2/TGFBR1 | 13 |
| 14/57 | 138/8111 | 3.61E-13 | 5.28E-12 | 1.65E-12 | AKT2/EGFR/ESR1/ESR2/HRAS/  HSP90AA1/MAP2K1/MAPK1/  MMP2/MMP9/PGR/PIK3R1/RARA/SRC | 14 |
| 16/57 | 212/8111 | 6.06E-13 | 8.28E-12 | 2.59E-12 | AKT2/AR/EGFR/ESR1/ESR2/  GSTM1/HRAS/HSP90AA1/JAK2/  MAP2K1/MAPK1/PGR/PIK3R1/  SRC/VDR/XIAP | 16 |
| 11/57 | 72/8111 | 1.63E-12 | 2.09E-11 | 6.54E-12 | AKT2/EGFR/FGFR1/HRAS/IGF1/  IGF1R/MAP2K1/MAPK1/MDM2/  MET/PIK3R1 | 11 |
| 11/57 | 76/8111 | 3.02E-12 | 3.65E-11 | 1.14E-11 | AKT2/BCL2L1/EGFR/MAP2K1/  MAPK1/MAPK8/PIK3R1/RAC1/  STAT1/TGFB2/TGFBR1 | 11 |
| 15/57 | 215/8111 | 1.14E-11 | 1.29E-10 | 4.05E-11 | AKT2/BCL2L1/CASP3/HRAS/  HSP90AA1/JAK2/MAPK1/MAPK14/  MAPK8/MMP9/PIK3R1/PPARG/  RAC1/SOD2/SRC | 15 |
| 11/57 | 86/8111 | 1.23E-11 | 1.32E-10 | 4.14E-11 | AKT2/CASP3/EGFR/HRAS/MAP2K1/  MAPK1/MAPK8/PIK3R1/RAC1/TGFB2/  TGFBR1 | 11 |
| 15/57 | 219/8111 | 1.48E-11 | 1.52E-10 | 4.75E-11 | AKT2/BCL2L1/CCNA2/CDK2/  CHEK1/HRAS/IL2/JAK3/MAP2K1/  MAPK1/MAPK8/PIK3R1/TGFB2/  TGFBR1/XIAP | 15 |
| 13/57 | 147/8111 | 1.65E-11 | 1.61E-10 | 5.04E-11 | AKT2/EGFR/ESR1/ESR2/FGFR1/  HRAS/IGF1/IGF1R/KIT/MAP2K1/  MAPK1/PGR/PIK3R1 | 13 |
| 10/57 | 70/8111 | 3.69E-11 | 3.44E-10 | 1.08E-10 | AKT2/EGFR/FGFR1/FGFR2/HRAS/  KIT/MAP2K1/MAPK1/MET/PIK3R1 | 10 |
| 10/57 | 71/8111 | 4.27E-11 | 3.81E-10 | 1.19E-10 | CTNNA1/EGFR/FGFR1/IGF1R/INSR/  MAPK1/MET/RAC1/SRC/TGFBR1 | 10 |
| 12/57 | 129/8111 | 6.02E-11 | 5.14E-10 | 1.61E-10 | AKT2/EGFR/HRAS/MAP2K1/MAPK1/  MAPK14/MAPK8/MMP2/MMP9/  PIK3R1/SRC/TGFBR1 | 12 |
| 14/57 | 201/8111 | 6.32E-11 | 5.18E-10 | 1.62E-10 | AKT2/EGFR/HRAS/IGF1/IGF1R/KDR/  MAP2K1/MAPK1/MAPK8/MET/  PIK3R1/RAC1/SRC/XIAP | 14 |
| 10/57 | 76/8111 | 8.60E-11 | 6.78E-10 | 2.12E-10 | AKT2/BCL2L1/HRAS/MAP2K1/  MAPK1/MDM2/PIK3R1/PTPN11/  TGFB2/TGFBR1 | 10 |
| 13/57 | 168/8111 | 9.00E-11 | 6.84E-10 | 2.14E-10 | AKT2/BCL2L1/EGFR/GSTM1/GSTP1/  HRAS/IGF1R/MAP2K1/MAPK1/  MET/PIK3R1/TGFB2/TGFBR1 | 13 |
| 11/57 | 104/8111 | 1.02E-10 | 7.44E-10 | 2.33E-10 | AKT2/HRAS/IL2/MAPK1/MAPK14/  MAPK8/MDM2/PIK3R1/PTPN11/  SRC/STAT1 | 11 |
| 12/57 | 139/8111 | 1.46E-10 | 1.03E-09 | 3.22E-10 | AKT2/GSTM1/GSTP1/HSP90AA1/  KDR/MAPK14/MAPK8/MMP2/MMP9/  PIK3R1/RAC1/SRC | 12 |
| 12/57 | 143/8111 | 2.03E-10 | 1.37E-09 | 4.30E-10 | AKT2/FGFR1/FGFR2/HRAS/IGF1/  IGF1R/JAK2/JAK3/MAP2K1/MAPK1/  MAPK14/PIK3R1 | 12 |
| 9/57 | 59/8111 | 2.08E-10 | 1.37E-09 | 4.30E-10 | AKT2/HRAS/KDR/MAP2K1/MAPK1/  MAPK14/PIK3R1/RAC1/SRC | 9 |
| 11/57 | 112/8111 | 2.29E-10 | 1.47E-09 | 4.60E-10 | AKT2/BCL2L1/CASP3/JAK2/MAPK1/  MAPK14/MAPK8/PIK3CG/STAT1/  TGFB2/XIAP | 11 |
| 8/57 | 41/8111 | 2.85E-10 | 1.77E-09 | 5.55E-10 | EGFR/HRAS/MAP2K1/MAPK1/  MDM2/MMP2/MMP9/SRC | 8 |
| 12/57 | 149/8111 | 3.29E-10 | 1.98E-09 | 6.21E-10 | AKT2/CDK2/CTNNA1/EGFR/FGFR2/  HRAS/MAP2K1/MAPK1/MET/PIK3R1/  TGFB2/TGFBR1 | 12 |
| 10/57 | 89/8111 | 4.28E-10 | 2.50E-09 | 7.85E-10 | AKT2/EGFR/HRAS/JAK2/MAP2K1/  MAPK1/MAPK14/PIK3R1/PTPN11/STAT1 | 10 |
| 13/57 | 193/8111 | 5.12E-10 | 2.92E-09 | 9.13E-10 | AKT2/CASP3/HRAS/JAK2/MAP2K1/  MAPK1/MAPK14/MAPK8/PIK3CG/  PIK3R1/RAC1/SRC/STAT1 | 13 |
| 12/57 | 156/8111 | 5.62E-10 | 3.11E-09 | 9.75E-10 | AKT2/CCNA2/CDK2/CHEK1/HRAS/  MAP2K1/MAPK1/MAPK14/MDM2/  PIK3R1/TGFB2/TGFBR1 | 12 |
| 9/57 | 69/8111 | 8.87E-10 | 4.79E-09 | 1.50E-09 | AKT2/HRAS/MAP2K1/MAPK1/  MET/PIK3R1/PTPN11/RAC1/TGFB2 | 9 |
| 11/57 | 128/8111 | 9.78E-10 | 5.14E-09 | 1.61E-09 | AKT2/MAP2K1/MAPK1/MAPK14/  MAPK8/PIK3R1/PPARG/RAC1/STAT1/  TGFB2/TGFBR1 | 11 |
| 9/57 | 73/8111 | 1.49E-09 | 7.62E-09 | 2.39E-09 | AKT2/BCL2L1/CASP3/GSTM1/  GSTP1/MAPK1/MDM2/PIK3R1/XIAP | 9 |
| 9/57 | 75/8111 | 1.90E-09 | 9.52E-09 | 2.98E-09 | AKT2/EGFR/HRAS/IGF1/IGF1R/  MAP2K1/MAPK1/MDM2/PIK3R1 | 9 |
| 10/57 | 107/8111 | 2.69E-09 | 1.31E-08 | 4.11E-09 | HSP90AA1/IL2/JAK2/JAK3/MAPK1/  MAPK14/MAPK8/RARA/STAT1/TGFBR1 | 10 |
| 9/57 | 85/8111 | 5.91E-09 | 2.82E-08 | 8.83E-09 | AKT2/EGFR/ERBB4/HRAS/MAP2K1/  MAPK1/MAPK8/PIK3R1/SRC | 9 |
| 12/57 | 192/8111 | 6.12E-09 | 2.85E-08 | 8.93E-09 | AKT2/HRAS/ITK/JAK2/JAK3/  MAP2K1/MAPK1/PIK3CG/PIK3R1/  RAC1/SRC/STAT1 | 12 |
| 10/57 | 119/8111 | 7.63E-09 | 3.47E-08 | 1.09E-08 | AKT2/HRAS/IGF1/JAK2/MAP2K1/  MAPK1/MAPK14/MAPK8/PIK3R1/STAT1 | 10 |
| 8/57 | 67/8111 | 1.69E-08 | 7.55E-08 | 2.36E-08 | AKT2/CCNA2/HRAS/KIT/MAP2K1/  MAPK1/PIK3R1/RARA | 8 |
| 8/57 | 68/8111 | 1.91E-08 | 8.32E-08 | 2.61E-08 | AKT2/HRAS/MAP2K1/MAPK1/  MAPK14/MAPK8/PIK3R1/RAC1 | 8 |
| 8/57 | 70/8111 | 2.41E-08 | 1.03E-07 | 3.23E-08 | CASP3/EGFR/MAPK14/MAPK8/  MET/PTPN11/RAC1/SRC | 8 |
| 10/57 | 136/8111 | 2.78E-08 | 1.16E-07 | 3.65E-08 | AKT2/BCL2L1/CASP3/HRAS/  MAP2K1/MAPK1/MAPK8/PARP1/  PIK3R1/XIAP | 10 |
| 8/57 | 72/8111 | 3.02E-08 | 1.24E-07 | 3.88E-08 | AKT2/EGFR/HRAS/JAK3/MAP2K1/  MAPK1/MET/PIK3R1 | 8 |
| 9/57 | 104/8111 | 3.56E-08 | 1.43E-07 | 4.49E-08 | AKT2/HRAS/IL2/ITK/MAP2K1/  MAPK1/MAPK14/MAPK8/PIK3R1 | 9 |
| 10/57 | 148/8111 | 6.25E-08 | 2.47E-07 | 7.72E-08 | AKT2/EGFR/HRAS/INSR/KIT/  MAP2K1/MAPK1/PIK3CG/  PIK3R1/PTPN11 | 10 |
| 9/57 | 119/8111 | 1.16E-07 | 4.48E-07 | 1.40E-07 | AKT2/HRAS/MAP2K1/MAPK1/  MAPK14/MAPK8/PIK3R1/PTPN11/RAC1 | 9 |
| 11/57 | 202/8111 | 1.18E-07 | 4.48E-07 | 1.40E-07 | AKT2/CASP3/CCNA2/CDK2/JAK3/  MAPK14/MAPK8/MDM2/PIK3R1/  RAC1/STAT1 | 11 |
| 11/57 | 203/8111 | 1.24E-07 | 4.62E-07 | 1.45E-07 | AKT2/INSR/MAPK14/MAPK8/  MMP2/MMP9/PARP1/PIK3R1/  RAC1/TGFB2/TGFBR1 | 11 |
| 11/57 | 204/8111 | 1.30E-07 | 4.73E-07 | 1.48E-07 | CASP3/CCNA2/CDK2/CHEK1/  HRAS/JAK3/MAPK1/MDM2/  PIK3R1/RAC1/SRC | 11 |
| 7/57 | 58/8111 | 1.32E-07 | 4.73E-07 | 1.48E-07 | AKT2/CTNNA1/EGFR/HRAS/  MAP2K1/MAPK1/PIK3R1 | 7 |
| 9/57 | 121/8111 | 1.34E-07 | 4.73E-07 | 1.48E-07 | AKT2/ESR1/HRAS/MAP2K1/  MAPK1/MDM2/PIK3R1/SRC/STAT1 | 9 |
| 10/57 | 162/8111 | 1.47E-07 | 5.12E-07 | 1.60E-07 | AKT2/BCL2L1/EGFR/HRAS/  IL2/JAK2/JAK3/PIK3R1/PTPN11/  STAT1 | 10 |
| 8/57 | 89/8111 | 1.63E-07 | 5.56E-07 | 1.74E-07 | AKT2/HRAS/IGF1/IGF1R/INSR/  PIK3R1/PPARG/SOD2 | 8 |
| 11/57 | 212/8111 | 1.93E-07 | 6.48E-07 | 2.03E-07 | AKT2/BCL2L1/CASP3/CHEK1/  HRAS/MAP2K1/MAPK1/MAPK14/  MAPK8/PIK3R1/RAC1 | 11 |
| 7/57 | 62/8111 | 2.11E-07 | 6.98E-07 | 2.19E-07 | AKT2/HRAS/IGF1/IGF1R/INSR/  PIK3R1/SOD2 | 7 |
| 8/57 | 93/8111 | 2.30E-07 | 7.44E-07 | 2.33E-07 | EGFR/HRAS/MAP2K1/MAPK1/  MAPK14/MAPK8/MMP2/SRC | 8 |
| 9/57 | 129/8111 | 2.32E-07 | 7.44E-07 | 2.33E-07 | AR/AURKA/CDK2/IGF1/IGF1R/  MAP2K1/MAPK1/MAPK14/PGR | 9 |
| 8/57 | 98/8111 | 3.45E-07 | 1.09E-06 | 3.41E-07 | AKT2/EGFR/HRAS/MAP2K1/  MAPK1/MAPK8/PIK3R1/RAC1 | 8 |
| 11/57 | 225/8111 | 3.52E-07 | 1.09E-06 | 3.42E-07 | AKT2/CASP3/EGFR/HRAS/  MAP2K1/MAPK1/MAPK14/  MDM2/PIK3R1/RAC1/SRC | 11 |
| 9/57 | 137/8111 | 3.89E-07 | 1.19E-06 | 3.73E-07 | AKT2/IL2/MAP2K1/MAPK1/  MAPK14/MAPK8/PIK3R1/RAC1/SRC | 9 |
| 10/57 | 180/8111 | 3.95E-07 | 1.19E-06 | 3.73E-07 | AKT2/CASP3/JAK2/MAPK1/  MAPK14/MAPK8/SRC/STAT1/  TGFB2/VDR | 10 |
| 9/57 | 139/8111 | 4.40E-07 | 1.31E-06 | 4.10E-07 | AKT2/BCL2L1/CASP3/CDK2/  IL2/JAK3/MAPK8/PIK3R1/STAT1 | 9 |
| 8/57 | 102/8111 | 4.71E-07 | 1.38E-06 | 4.32E-07 | AKT2/IL2/MAPK1/MAPK14/  MAPK8/PIK3R1/TGFB2/TGFBR1 | 8 |
| 8/57 | 104/8111 | 5.47E-07 | 1.58E-06 | 4.95E-07 | AKT2/MAP2K1/MAPK1/MAPK14/  MAPK8/PIK3R1/RAC1/STAT1 | 8 |
| 10/57 | 192/8111 | 7.18E-07 | 2.04E-06 | 6.40E-07 | BCL2L1/CCNA2/IGF1/IGF1R/  MDM2/MET/MMP9/PLAU/PPARG/RARA | 10 |
| 8/57 | 109/8111 | 7.86E-07 | 2.21E-06 | 6.91E-07 | AKT2/EGFR/IGF1/IGF1R/INSR/  MAP2K1/MAPK1/PIK3R1 | 8 |
| 8/57 | 112/8111 | 9.68E-07 | 2.68E-06 | 8.40E-07 | AKT2/CASP3/MAP2K1/MAPK1/  MAPK14/MAPK8/MMP9/PIK3R1 | 8 |
| 8/57 | 114/8111 | 1.11E-06 | 3.03E-06 | 9.49E-07 | CTNNA1/ITK/MAPK14/MMP2/  MMP9/PIK3R1/PTPN11/RAC1 | 8 |
| 9/57 | 157/8111 | 1.23E-06 | 3.33E-06 | 1.04E-06 | AKT2/CASP3/CDK2/EGFR/HRAS/  MAP2K1/MAPK1/PIK3R1/STAT1 | 9 |
| 8/57 | 119/8111 | 1.54E-06 | 4.09E-06 | 1.28E-06 | AKT2/HRAS/MAP2K1/MAPK1/  MAPK14/MAPK8/PIK3R1/RAC1 | 8 |
| 7/57 | 92/8111 | 3.20E-06 | 8.40E-06 | 2.63E-06 | IL2/JAK2/JAK3/MAPK1/MAPK14/  MAPK8/STAT1 | 7 |
| 8/57 | 137/8111 | 4.44E-06 | 1.15E-05 | 3.61E-06 | AKT2/BCL2L1/HRAS/IGF1R/  MAP2K1/MAPK1/MAPK8/PIK3R1 | 8 |
| 6/57 | 64/8111 | 5.04E-06 | 1.29E-05 | 4.04E-06 | AKT2/ESR2/HRAS/MAP2K1/  MAPK1/PIK3R1 | 6 |
| 10/57 | 246/8111 | 6.75E-06 | 1.71E-05 | 5.35E-06 | AKT2/BCL2L1/EGFR/MAPK1/  MAPK14/MAPK8/MDM2/PIK3R1/  RAC1/SRC | 10 |
| 10/57 | 249/8111 | 7.52E-06 | 1.88E-05 | 5.89E-06 | AKT2/CASP3/HRAS/HSP90AA1/  MAP2K1/MAPK1/MAPK14/MAPK8/  PIK3CG/RAC1 | 10 |
| 11/57 | 310/8111 | 8.14E-06 | 2.01E-05 | 6.30E-06 | CASP3/EGFR/HRAS/MAP2K1/  MAPK1/MDM2/MET/MMP9/PIK3R1/  PLAU/TGFB2 | 11 |
| 6/57 | 73/8111 | 1.09E-05 | 2.64E-05 | 8.28E-06 | BCL2L1/CASP3/CDK2/CHEK1/  IGF1/MDM2 | 6 |
| 8/57 | 155/8111 | 1.11E-05 | 2.64E-05 | 8.28E-06 | AKT2/HRAS/IGF1/IGF1R/INSR/  MAP2K1/MAPK1/PIK3R1 | 8 |
| 8/57 | 155/8111 | 1.11E-05 | 2.64E-05 | 8.28E-06 | AKT2/CASP3/INSR/MAPK14/  MAPK8/PIK3R1/PPARG/RAC1 | 8 |
| 7/57 | 113/8111 | 1.26E-05 | 2.96E-05 | 9.28E-06 | AKT2/HRAS/JAK2/MAP2K1/  MAPK1/PIK3CG/PIK3R1 | 7 |
| 11/57 | 331/8111 | 1.51E-05 | 3.53E-05 | 1.11E-05 | AKT2/CASP3/CCNA2/CDK2/  EGFR/HRAS/MAP2K1/MAPK1/  MDM2/PIK3R1/STAT1 | 11 |
| 9/57 | 218/8111 | 1.82E-05 | 4.19E-05 | 1.31E-05 | EGFR/FGFR1/FGFR2/HRAS/  MAP2K1/MAPK1/PIK3R1/RAC1/SRC | 9 |
| 7/57 | 120/8111 | 1.87E-05 | 4.25E-05 | 1.33E-05 | AKT2/CCNA2/IGF1/IGF1R/INSR/  PIK3R1/PPARG | 7 |
| 6/57 | 82/8111 | 2.13E-05 | 4.81E-05 | 1.51E-05 | AKT2/HRAS/MAP2K1/MAPK1/  PIK3R1/RAC1 | 6 |
| 7/57 | 131/8111 | 3.30E-05 | 7.35E-05 | 2.30E-05 | CASP3/HRAS/MAP2K1/MAPK1/  PIK3R1/PTPN11/RAC1 | 7 |
| 6/57 | 92/8111 | 4.12E-05 | 9.08E-05 | 2.84E-05 | AKT2/BCL2L1/CASP3/CDK2/  PIK3R1/XIAP | 6 |
| 7/57 | 137/8111 | 4.40E-05 | 9.59E-05 | 3.00E-05 | AKT2/HRAS/INSR/MAP2K1/  MAPK1/MAPK8/PIK3R1 | 7 |
| 6/57 | 94/8111 | 4.65E-05 | 0.000100375 | 3.14E-05 | CASP3/HSP90AA1/MAPK1/  MAPK14/MAPK8/MMP9 | 6 |
| 5/57 | 60/8111 | 5.83E-05 | 0.000124509 | 3.90E-05 | HRAS/IGF1/IGF1R/MAP2K1/MAPK1 | 5 |
| 4/57 | 32/8111 | 6.81E-05 | 0.000143874 | 4.51E-05 | BCL2L1/CASP3/MAPK8/XIAP | 4 |
| 7/57 | 159/8111 | 0.000113147 | 0.000236686 | 7.41E-05 | HSP90AA1/JAK2/JAK3/MAPK8/  PARP1/STAT1/XIAP | 7 |
| 4/57 | 37/8111 | 0.000121808 | 0.000249706 | 7.82E-05 | IGF1/INSR/MAPK1/PIK3R1 | 4 |
| 4/57 | 37/8111 | 0.000121808 | 0.000249706 | 7.82E-05 | HRAS/MAP2K1/MAPK1/PPARG | 4 |
| 7/57 | 172/8111 | 0.000184665 | 0.000374816 | 0.000117401 | AKT2/CASP3/JAK2/MAP2K1/  MAPK1/PIK3R1/STAT1 | 7 |
| 5/57 | 77/8111 | 0.000192623 | 0.000383375 | 0.000120082 | CTNNA1/MET/PIK3R1/RAC1/SRC | 5 |
| 5/57 | 77/8111 | 0.000192623 | 0.000383375 | 0.000120082 | JAK2/MAPK1/MAPK14/STAT1/TGFB2 | 5 |
| 6/57 | 124/8111 | 0.000216951 | 0.000427643 | 0.000133947 | AKT2/MAPK1/MAPK14/  PIK3CG/PIK3R1/SRC | 6 |
| 7/57 | 181/8111 | 0.00025289 | 0.000493739 | 0.00015465 | BCL2L1/HSP90AA1/MAPK1/  MAPK14/MAPK8/STAT1/XIAP | 7 |
| 7/57 | 182/8111 | 0.000261583 | 0.000505892 | 0.000158456 | HRAS/MAPK1/MET/PIK3R1/  PTPN11/RAC1/SRC | 7 |
| 4/57 | 46/8111 | 0.000287153 | 0.000550152 | 0.00017232 | INSR/MAPK1/MAPK8/PIK3R1 | 4 |
| 7/57 | 190/8111 | 0.000340169 | 0.000645691 | 0.000202245 | AKT2/MAP2K1/MAPK1/MAPK14/  PIK3R1/RAC1/SRC | 7 |
| 5/57 | 88/8111 | 0.000360157 | 0.000677359 | 0.000212164 | EGFR/HRAS/MAP2K1/MAPK1/SRC | 5 |
| 6/57 | 137/8111 | 0.000372228 | 0.000693698 | 0.000217282 | AKT2/HRAS/MAP2K1/MAPK1/  PIK3CG/TGFBR1 | 6 |
| 7/57 | 197/8111 | 0.000423578 | 0.000782283 | 0.000245028 | CASP3/MAPK1/MAPK14/MAPK8/  PTPN11/RAC1/SRC | 7 |
| 5/57 | 97/8111 | 0.00056471 | 0.001033622 | 0.000323753 | AKT2/MAP2K1/MAPK1/PIK3R1/RAC1 | 5 |
| 5/57 | 98/8111 | 0.000591907 | 0.001073814 | 0.000336342 | IGF1/MAPK14/MAPK8/PIK3R1/SRC | 5 |
| 6/57 | 154/8111 | 0.000693682 | 0.001247411 | 0.000390717 | EGFR/HRAS/MAP2K1/MAPK1/  PIK3CG/SRC | 6 |
| 5/57 | 106/8111 | 0.000846414 | 0.001508825 | 0.000472597 | EGFR/FGFR1/MAP2K1/MAPK1/VDR | 5 |
| 5/57 | 108/8111 | 0.00092114 | 0.001627876 | 0.000509887 | AKT2/INSR/MAPK8/PIK3R1/PTPN11 | 5 |
| 7/57 | 232/8111 | 0.001118781 | 0.001960258 | 0.000613996 | EGFR/IL2/MAPK1/MAPK14/  MAPK8/PIK3R1/STAT1 | 7 |
| 4/57 | 68/8111 | 0.001277885 | 0.002220055 | 0.00069537 | BCL2L1/HRAS/MAPK8/SRC | 4 |
| 4/57 | 69/8111 | 0.001349487 | 0.002324747 | 0.000728162 | AKT2/JAK2/MAPK8/PTPN11 | 4 |
| 5/57 | 124/8111 | 0.001708632 | 0.002918913 | 0.000914268 | CCNA2/CDK2/CHEK1/MDM2/TGFB2 | 5 |
| 7/57 | 252/8111 | 0.00180381 | 0.003056042 | 0.00095722 | EGFR/FGFR2/HRAS/IGF1R/  MDM2/SRC/TGFBR1 | 7 |
| 4/57 | 76/8111 | 0.001931165 | 0.00324499 | 0.001016403 | CASP3/MAPK1/MAPK14/MAPK8 | 4 |
| 8/57 | 369/8111 | 0.003981296 | 0.006635493 | 0.002078383 | AKT2/CASP3/HRAS/INSR/  MAP2K1/MAPK1/MAPK8/PIK3R1 | 8 |
| 6/57 | 219/8111 | 0.004163117 | 0.006856671 | 0.002147661 | AKT2/MAP2K1/MAPK1/  MAPK8/PIK3R1/RAC1 | 6 |
| 4/57 | 94/8111 | 0.004180897 | 0.006856671 | 0.002147661 | BMP2/MAPK1/TGFB2/TGFBR1 | 4 |
| 3/57 | 51/8111 | 0.005397091 | 0.00871401 | 0.00272942 | IGF1/IGF1R/INSR | 3 |
| 4/57 | 101/8111 | 0.005398435 | 0.00871401 | 0.00272942 | HRAS/KIT/MAP2K1/MAPK1 | 4 |
| 4/57 | 104/8111 | 0.005985977 | 0.009586916 | 0.003002834 | BCL2L1/PARP1/PLAU/XIAP | 4 |
| 5/57 | 167/8111 | 0.006153888 | 0.009779434 | 0.003063135 | AKT2/INSR/MAP2K1/MAPK1/PIK3CG | 5 |
| 6/57 | 240/8111 | 0.00648059 | 0.010219392 | 0.003200939 | EGFR/ERBB4/FGFR1/FGFR2/KDR/MET | 6 |
| 3/57 | 57/8111 | 0.007361478 | 0.01151987 | 0.003608278 | AKT2/INSR/PIK3R1 | 3 |
| 4/57 | 115/8111 | 0.00850251 | 0.013204655 | 0.004135989 | CASP3/HRAS/MAP2K1/MAPK1 | 4 |
| 3/57 | 65/8111 | 0.010563964 | 0.016282802 | 0.005100133 | IL2/STAT1/TGFB2 | 3 |
| 3/57 | 67/8111 | 0.011472138 | 0.017550659 | 0.005497254 | HRAS/MAP2K1/MAPK1 | 3 |
| 6/57 | 273/8111 | 0.011848798 | 0.017992619 | 0.005635686 | CASP3/MAPK1/MAPK14/  MAPK8/PIK3R1/RAC1 | 6 |
| 8/57 | 475/8111 | 0.017150373 | 0.025851664 | 0.008097312 | BCL2L1/CASP3/HRAS/MAP2K1/  MAPK1/MAPK14/MAPK8/RAC1 | 8 |
| 4/57 | 150/8111 | 0.020807651 | 0.031135537 | 0.009752338 | AKT2/MAPK1/MAPK14/PIK3CG | 4 |
| 8/57 | 498/8111 | 0.022151947 | 0.032906879 | 0.010307161 | AKT2/BCL2L1/CASP3/JAK2/  PIK3R1/PTPN11/SRC/STAT1 | 8 |
| 4/57 | 155/8111 | 0.023153083 | 0.034146633 | 0.010695479 | CDK2/EGFR/MAP2K1/MAPK1 | 4 |
| 4/57 | 157/8111 | 0.024134748 | 0.035340167 | 0.01106932 | BMP2/CTNNA1/TGFB2/TGFBR1 | 4 |

**Table S6. Basic information of the top 20 pathways**

| **Table S6**. Basic information of the top 20 pathways | | | | | |
| --- | --- | --- | --- | --- | --- |
| Pathway ID | Pathway Description | Gene ID | Count | pvalue | Pathway Class |
| hsa05205 | Proteoglycans in cancer | AKT2/CASP3/EGFR/ERBB4/ESR1/FGFR1/HRAS/IGF1/IGF1R/KDR/MAP2K1/MAPK1/MAPK14/MDM2/MET/MMP2/MMP9/  PIK3R1/PLAU/PTPN11/RAC1/SRC/TGFB2 | 23 | 1.32E-22 | Cancer: overview |
| hsa05215 | Prostate cancer | AKT2/AR/CDK2/EGFR/FGFR1/FGFR2/GSTP1/HRAS/HSP90AA1/IGF1/IGF1R/  MAP2K1/MAPK1/MDM2/MMP9/PIK3R1/PLAU | 17 | 4.61E-20 | Cancer: specific types |
| hsa05207 | Chemical carcinogenesis-receptor activation | AKT2/AR/EGFR/ESR1/ESR2/GSTM1/HRAS/HSP90AA1/JAK2/MAP2K1/MAPK1/  PGR/PIK3R1/SRC/VDR/XIAP | 16 | 6.06E-13 | Cancer: overview |
| hsa05218 | Melanoma | AKT2/EGFR/FGFR1/HRAS/IGF1/IGF1R/MAP2K1/MAPK1/MDM2/MET/PIK3R1 | 11 | 1.63E-12 | Cancer: specific types |
| hsa05212 | Pancreatic cancer | AKT2/BCL2L1/EGFR/MAP2K1/MAPK1/MAPK8/PIK3R1/RAC1/STAT1/TGFB2/  TGFBR1 | 11 | 3.02E-12 | Cancer: specific types |
| hsa05210 | Colorectal cancer | AKT2/CASP3/EGFR/HRAS/MAP2K1/MAPK1/MAPK8/PIK3R1/RAC1/TGFB2/  TGFBR1 | 11 | 1.23E-11 | Cancer: specific types |
| hsa04151 | PI3K-Akt signaling pathway | AKT2/BCL2L1/CDK2/EGFR/ERBB4/FGFR1/FGFR2/HRAS/HSP90AA1/IGF1/  IGF1R/IL2/INSR/JAK2/JAK3/KDR/KIT/MAP2K1/MAPK1/MDM2/MET/PIK3CG/  PIK3R1/RAC1 | 24 | 2.14E-18 | Signal transduction |
| hsa04068 | FoxO signaling pathway | AKT2/CDK2/EGFR/HRAS/IGF1/IGF1R/INSR/MAP2K1/MAPK1/MAPK14/  MAPK8/MDM2/PIK3R1/SOD2/TGFB2/TGFBR1 | 16 | 2.79E-16 | Signal transduction |
| hsa04014 | Ras signaling pathway | AKT2/BCL2L1/EGFR/FGFR1/FGFR2/HRAS/IGF1/IGF1R/INSR/KDR/KIT/MAP2K1/MAPK1/MAPK8/MET/PIK3R1/PLA2G2A/PTPN11/RAC1 | 19 | 5.47E-16 | Signal transduction |
| hsa04010 | MAPK signaling pathway | AKT2/CASP3/EGFR/ERBB4/FGFR1/FGFR2/HRAS/IGF1/IGF1R/INSR/KDR/KIT/MAP2K1/MAPK1/MAPK14/MAPK8/MET/RAC1/  TGFB2/TGFBR1 | 20 | 2.91E-15 | Signal transduction |
| hsa04015 | Rap1 signaling pathway | AKT2/EGFR/FGFR1/FGFR2/HRAS/IGF1/IGF1R/INSR/KDR/KIT/MAP2K1/MAPK1/MAPK14/MET/PIK3R1/RAC1/SRC | 17 | 3.10E-14 | Signal transduction |
| hsa04917 | Prolactin signaling pathway | AKT2/ESR1/ESR2/HRAS/JAK2/MAP2K1/MAPK1/MAPK14/MAPK8/PIK3R1/SRC/STAT1 | 12 | 3.32E-14 | Endocrine system |
| hsa04914 | Progesterone-mediated oocyte maturation | AKT2/AURKA/CCNA2/CDK2/HSP90AA1/IGF1/IGF1R/MAP2K1/MAPK1/MAPK14/MAPK8/PGR/PIK3R1 | 13 | 1.07E-13 | Endocrine system |
| hsa04915 | Estrogen signaling pathway | AKT2/EGFR/ESR1/ESR2/HRAS/HSP90AA1/MAP2K1/MAPK1/  MMP2/MMP9/PGR/PIK3R1/RARA/SRC | 14 | 3.61E-13 | Endocrine system |
| hsa01522 | Endocrine resistance | AKT2/EGFR/ESR1/ESR2/HRAS/IGF1/IGF1R/MAP2K1/MAPK1/  MAPK14/MAPK8/MDM2/MMP2/MMP9/PIK3R1/SRC | 16 | 2.24E-18 | Drug resistance: antineoplastic |
| hsa01521 | EGFR tyrosine kinase inhibitor resistance | AKT2/BCL2L1/EGFR/FGFR2/HRAS/IGF1/IGF1R/JAK2/KDR/  MAP2K1/MAPK1/MET/PIK3R1/SRC | 14 | 1.14E-16 | Drug resistance: antineoplastic |
| hsa05161 | Hepatitis B | AKT2/CASP3/CCNA2/CDK2/HRAS/JAK2/JAK3/MAP2K1/  MAPK1/MAPK14/MAPK8/MMP9/PIK3R1/SRC/STAT1/TGFB2/  TGFBR1 | 17 | 3.85E-16 | Infectious disease: viral |
| hsa05166 | Human T-cell leukemia virus 1 infection | AKT2/BCL2L1/CCNA2/CDK2/CHEK1/HRAS/IL2/JAK3/MAP2K1/MAPK1/MAPK8/PIK3R1/TGFB2/TGFBR1/XIAP | 15 | 1.48E-11 | Infectious disease: viral |
| hsa04933 | AGE-RAGE signaling pathway in diabetic complications | AKT2/CASP3/HRAS/JAK2/MAPK1/MAPK14/MAPK8/MMP2/PIK3R1/RAC1/STAT1/TGFB2/TGFBR1 | 13 | 1.07E-13 | Endocrine and metabolic disease |
| hsa05417 | Lipid and atherosclerosis | AKT2/BCL2L1/CASP3/HRAS/HSP90AA1/JAK2/MAPK1/MAPK14/MAPK8/MMP9/PIK3R1/PPARG/RAC1/SOD2/SRC | 15 | 1.14E-11 | Cardiovascular disease |

**Table S7 The residues of proteins interacted with 4i**

| **Table S7** The residues of proteins interacted with **4i** | | | | | | | | | | | |
| --- | --- | --- | --- | --- | --- | --- | --- | --- | --- | --- | --- |
| EGFR | | ESR1 | | HRAS | | GIF1 | | MAPK1 | | AKT2 | |
| Residues | ΔASA,  Å2 | Residues | ΔASA,  Å2 | Residues | ΔASA, Å2 | Residues | ΔASA,  Å2 | Residues | ΔASA, Å2 | Residues | ΔASA, Å2 |
| Leu718 | 31.452 | Leu391 | 7.49 | Glu31 | 9.969 | Leu54O | 37.452 | Met106O | 17.159 | Lys160O | 17.159 |
| Gly796O | 23.946 | Glu353O | 3.65 | Arg789O | 11.641 | Leu57 | 5.788 | Asp104O | 1.693 | Phe163 | 17.28 |
| Cys797 | 13.385 | Arg394O | 3.881 | Thr785O | 13.699 | Val17O | 11.803 | Ala50O | 16.797 | Gly159O | 17.323 |
| Leu844O | 42.445 | Leu346H,O | 20.997 | Asn85H | 34.837 | Ala13O | 20.769 | Leu154O | 37.452 | Gly161O | 35.533 |
| Asp855H,O | 29.661 | Leu349O | 6.015 | Phe28O | 23.85 | Phe16O | 81.812 | Cys164 | 19.046 | Gly164 | 2.085 |
| Arg841 | 48.158 | Ala350O | 23.265 | Val29O | 8.18 | Asp12 | 40.755 | Lys52O | 26.653 | Leu183 | 6.81 |
| Asn842H | 10.789 | Met421 | 15.763 | Leu120 | 23.038 | Gln15 | 39.288 | Glu69 | 1.468 | Lys181O | 22.184 |
| Glu762 | 4.002 | Val534O | 31.835 | Thr786 | 21.881 | Glu9 | 19.001 | Asp165O | 22.869 | Asp293O | 48.293 |
| Thr790O | 17.596 | Leu525O | 45.607 | Glu783 | 36.297 | Leu5O | 34.614 | Gln103O | 20.096 | Leu158O | 7.848 |
| Thr854 | 15.494 | Thr347O | 29.897 | Asp30O | 13.164 |  |  | Ile29O | 46.499 | Met282O | 12.9 |
| Met766 | 9.255 | Met528 | 25.448 | Glu781 | 24.34 |  |  | Asp109H,O | 18.278 | Phe439O | 10.811 |
| Ala743O | 19.52 | Leu384O | 18.726 | Asp782 | 28.94 |  |  | Lys112O | 29.066 | Asp440H | 7.607 |
|  |  | Ile424O | 8.285 | Asp119O | 8.005 |  |  | Gly30 | 23.266 | Phe443 | 6.741 |
|  |  | Gly521O | 4.957 | Lys147 | 12.948 |  |  |  |  | Asn280 | 7.268 |
|  |  | Phe404O | 21.05 | Lys117H,O | 48.206 |  |  |  |  | Glu279O | 13.405 |
|  |  | Met388O | 10.782 | Ala146O | 3.653 |  |  |  |  | Glu236O | 24.75 |
|  |  | Leu428O | 4.199 | Ala18O | 11.576 |  |  |  |  | Arg6O | 27.006 |
|  |  |  |  | Gly15O | 8.625 |  |  |  |  | Lys277 | 9.627 |
|  |  |  |  |  |  |  |  |  |  | Ser9 | 26.49 |
|  |  |  |  |  |  |  |  |  |  | Thr162 | 8.874 |
| Average | 22.14 | Average | 16.6 | Average | 19 | Average | 32.4 | Average | 21.56 | Average | 16.5 |
| Note: OThe residues are overlapping with the interacting residues of native ligand; H The residues  interacted with 4i through hydrogen bond | | | | | | | | | | | |

**
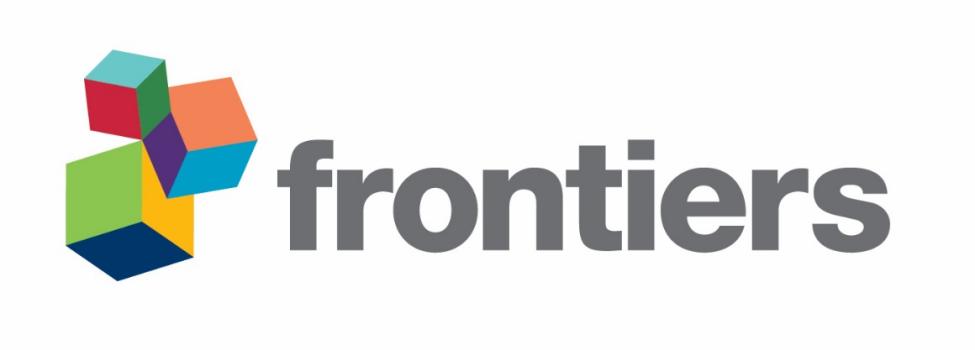
**

**Supplementary Figure 1.** The figure legends are required to have the same font as the main text, 12 point normal Times New Roman, single spaced. Please use a single paragraph for each legend and prepare the figures keeping in mind the PDF layout.
